# Supplementary material for: Radical asymmetric intramolecular α-cyclopropanation of aldehydes towards bicyclo[3.1.0]hexanes containing vicinal all-carbon quaternary stereocenters
Source: Nat Commun. 2018 Jan 15;9:227. doi: 10.1038/s41467-017-02231-7 (PMC5768789; doi:10.1038/s41467-017-02231-7)
Supplement: Supplementary file 2 — Supplementary Information [file 41467_2017_2231_MOESM2_ESM.pdf]

Supplementary Figures

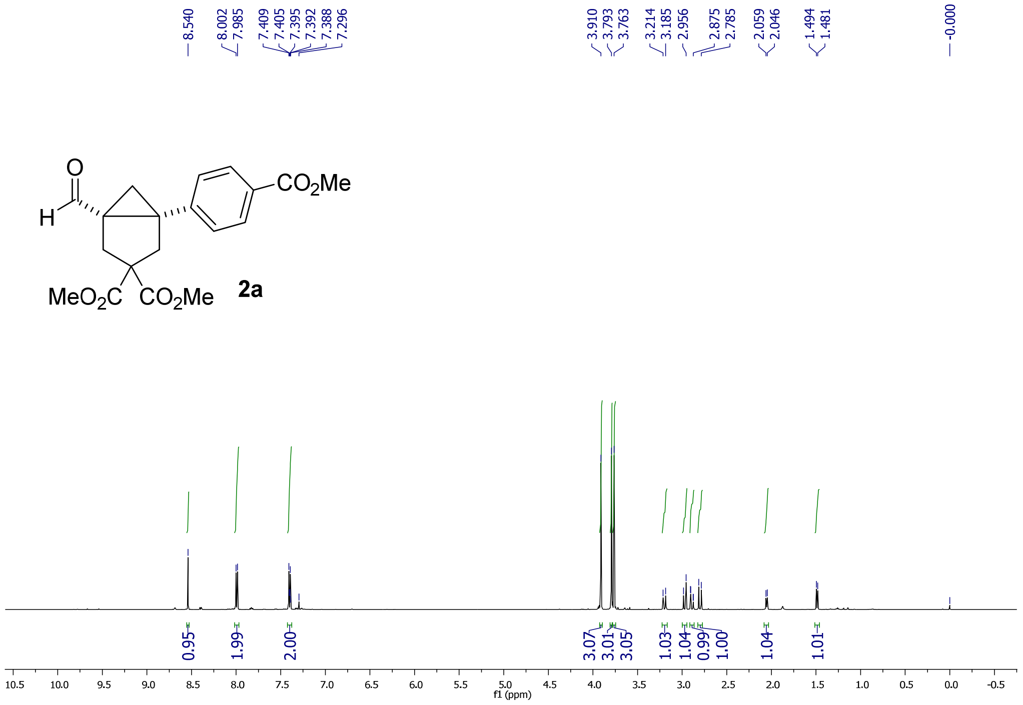

Supplementary Figure 1. ^{1}H NMR of **2a**

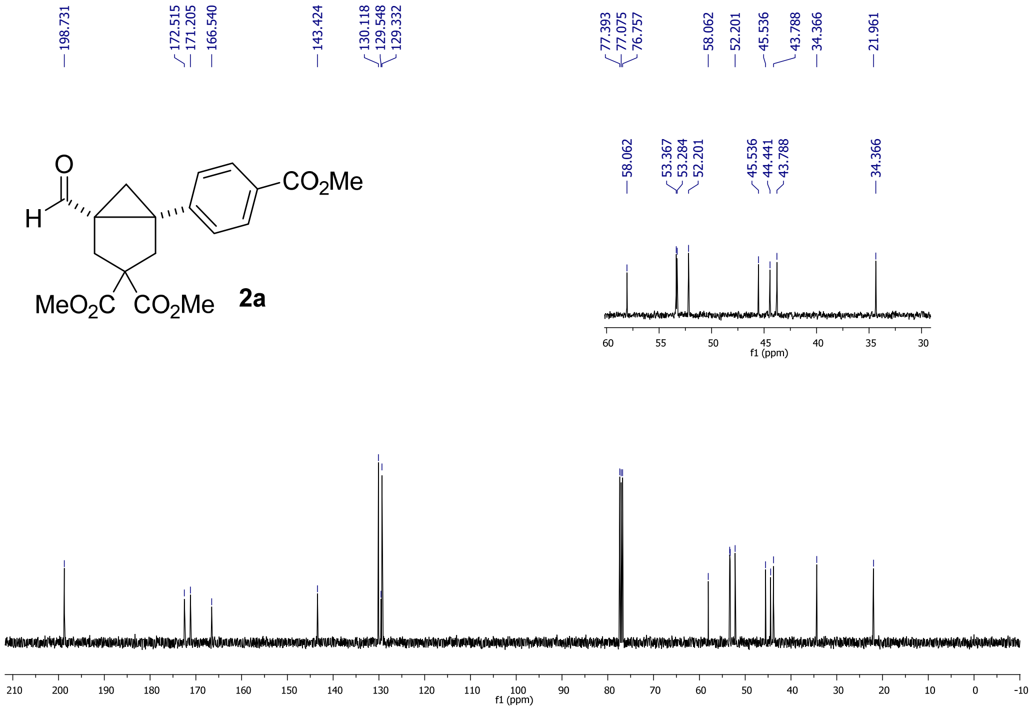

Supplementary Figure 2. ^{13}C NMR of **2a**

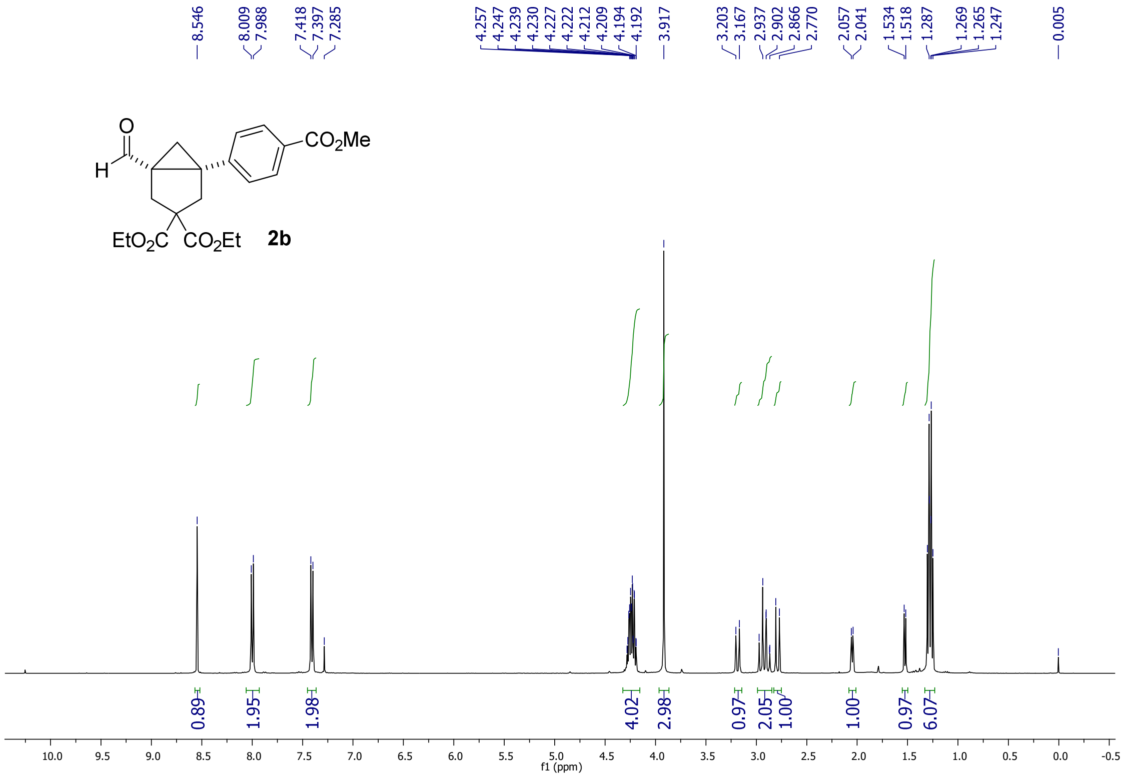

Supplementary Figure 3. ^{1}H NMR of **2b**

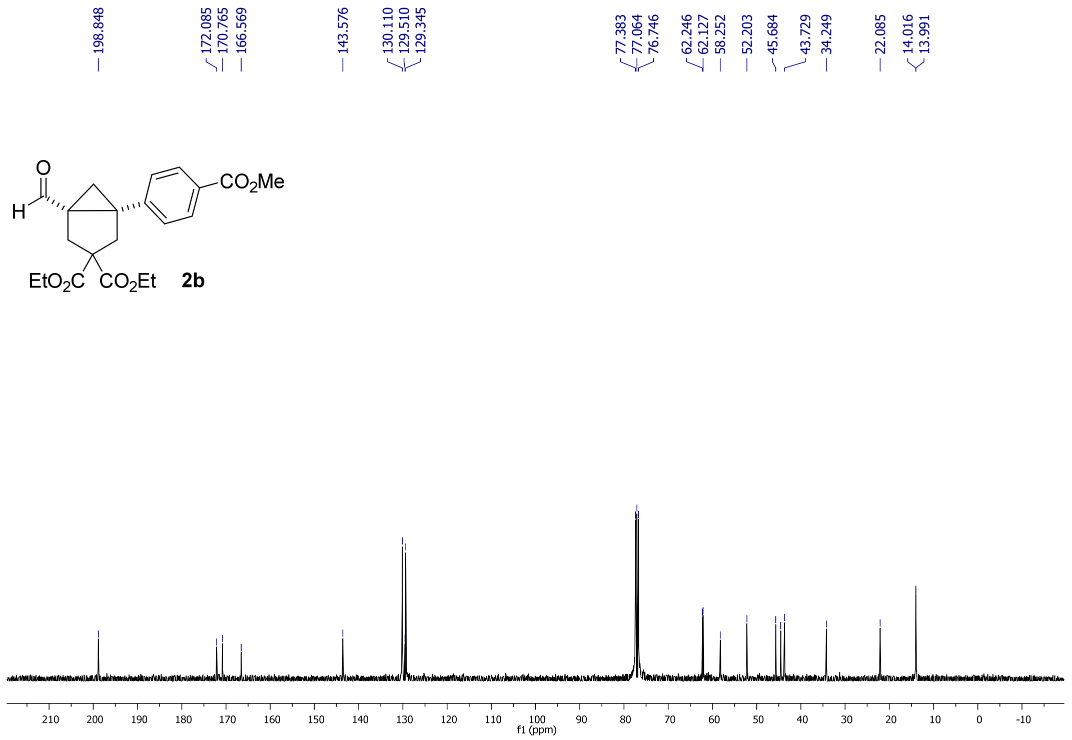

Supplementary Figure 4. ^{13}C NMR of **2b**

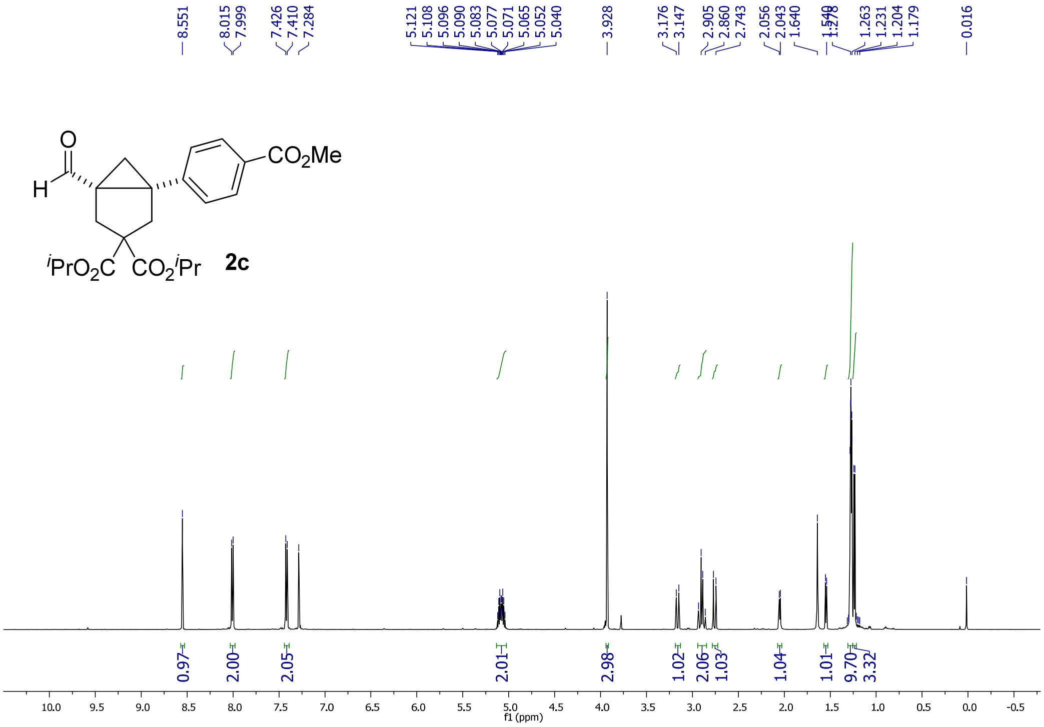

Supplementary Figure 5. ^{1}H NMR of **2c**

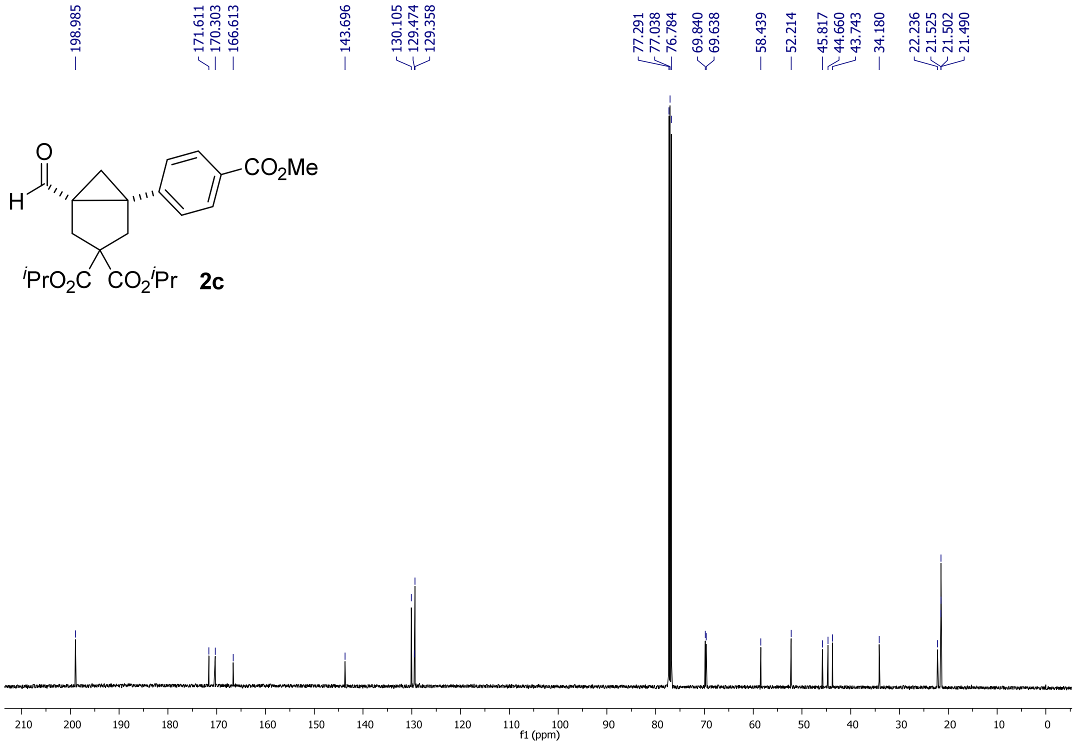

Supplementary Figure 6. ^{13}C NMR of **2c**

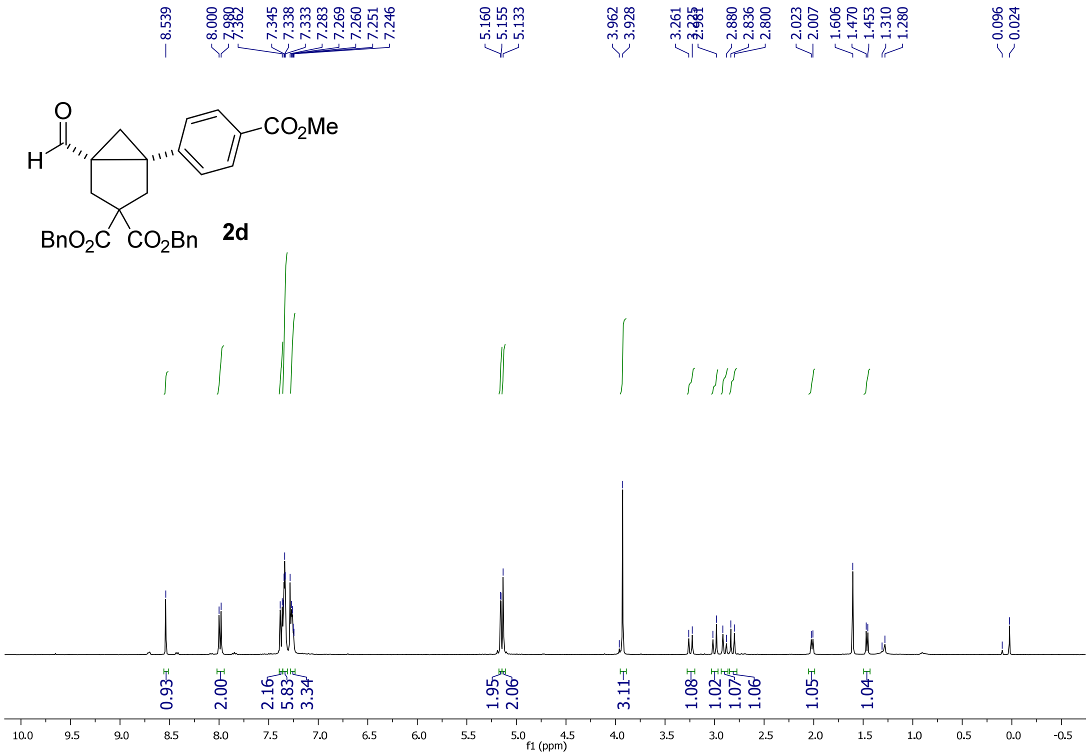

Supplementary Figure 7. ^{1}H NMR of **2d**

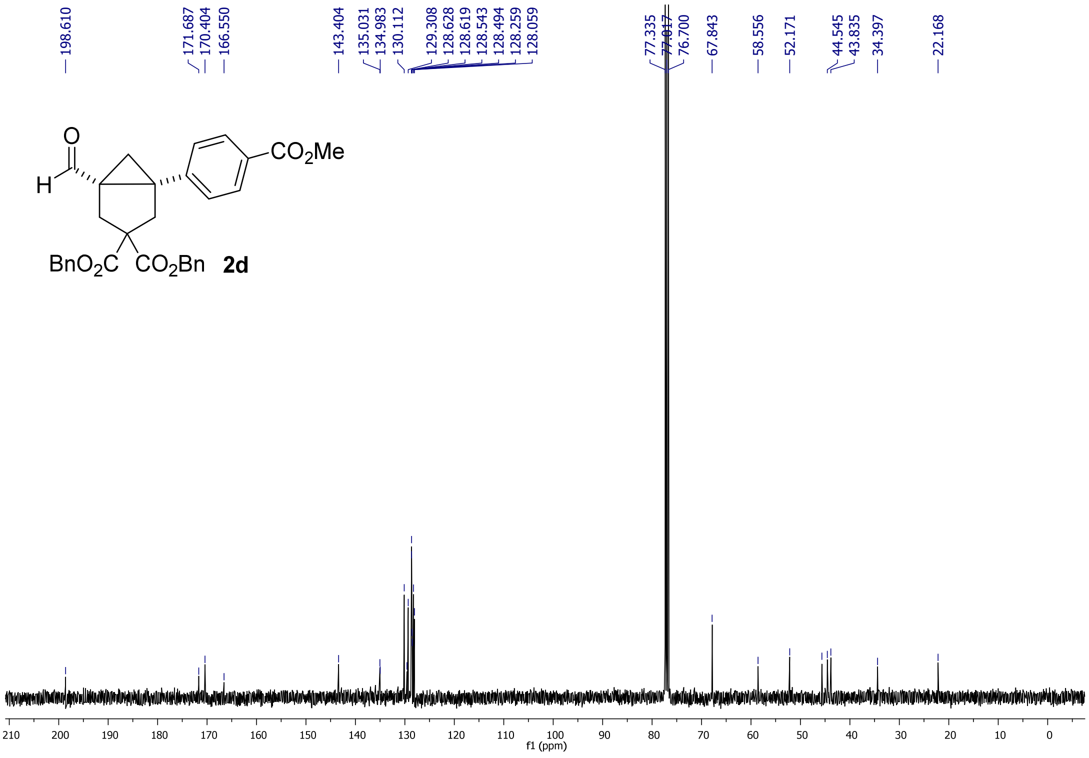

Supplementary Figure 8. ^{13}C NMR of **2d**

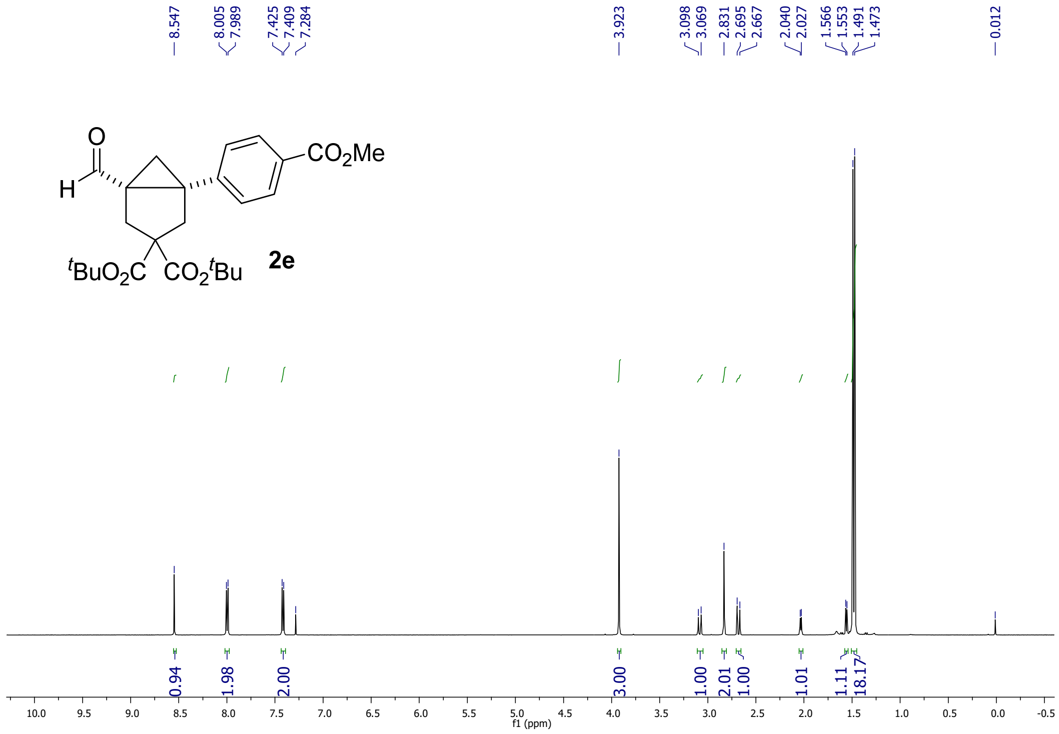

Supplementary Figure 9. ^{1}H NMR of **2e**

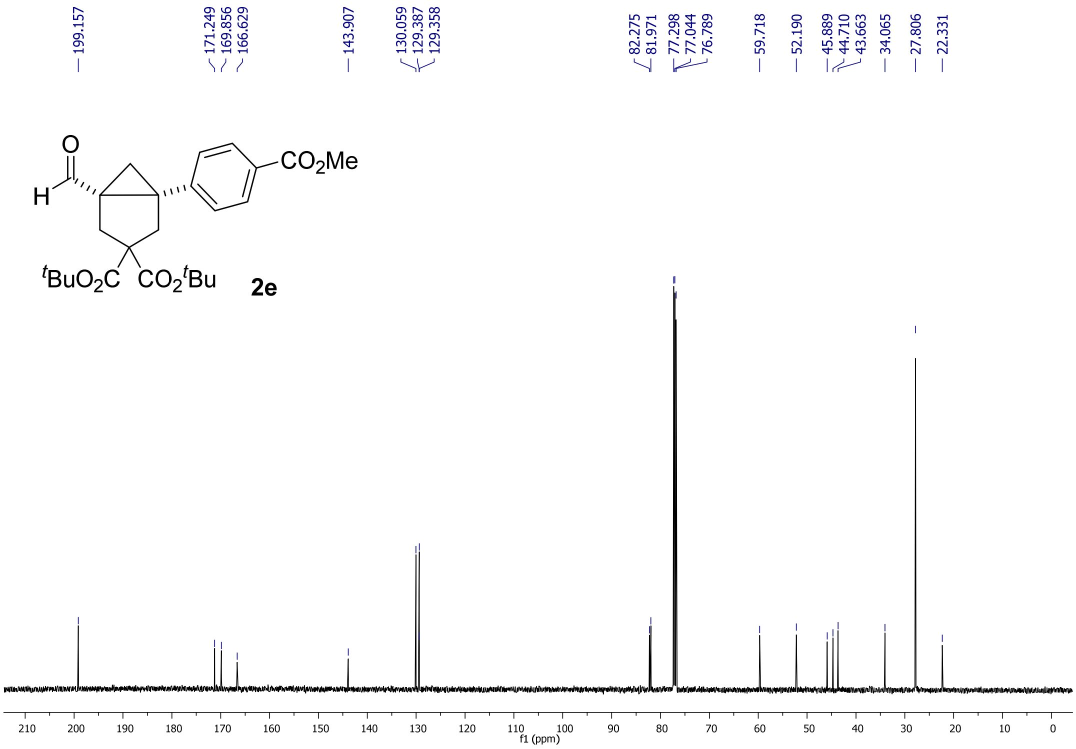

Supplementary Figure 10. ^{13}C NMR of **2e**

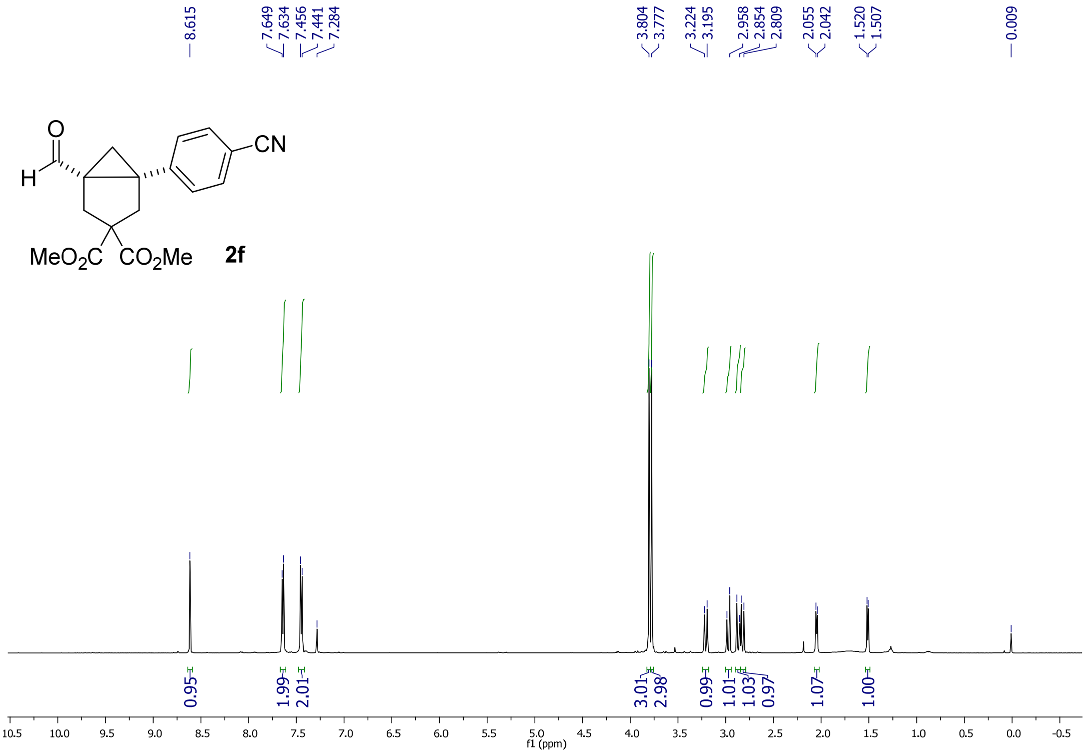

Supplementary Figure 11. ^{1}H NMR of **2f**

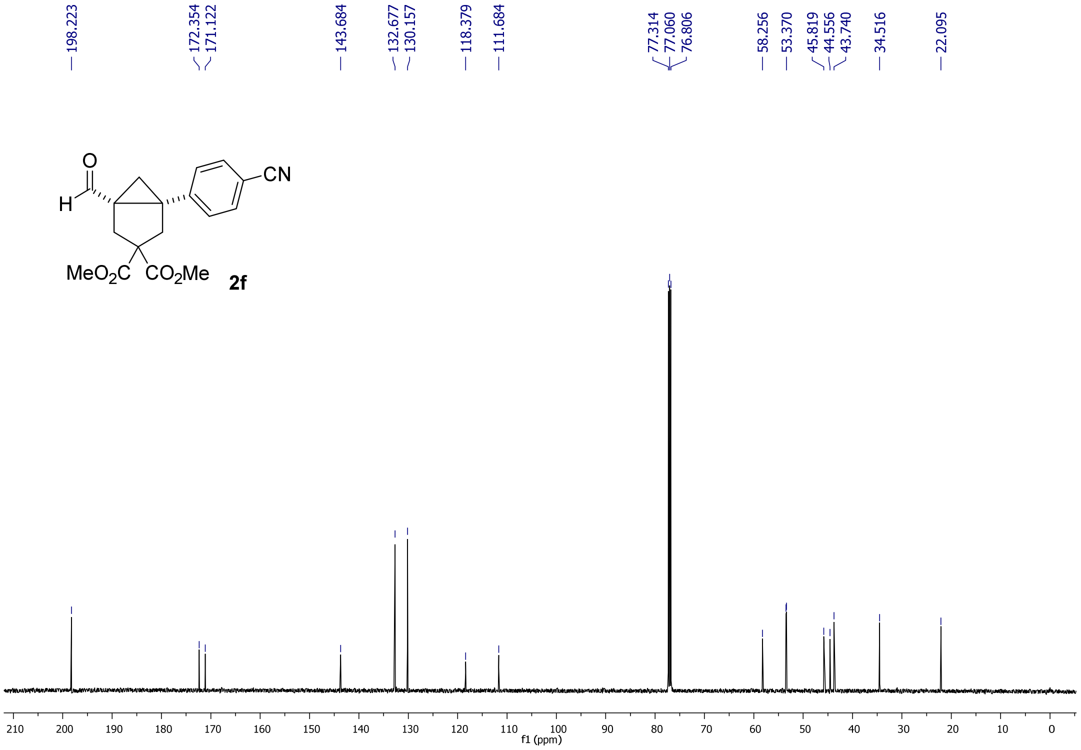

Supplementary Figure 12. ^{13}C NMR of **2f**

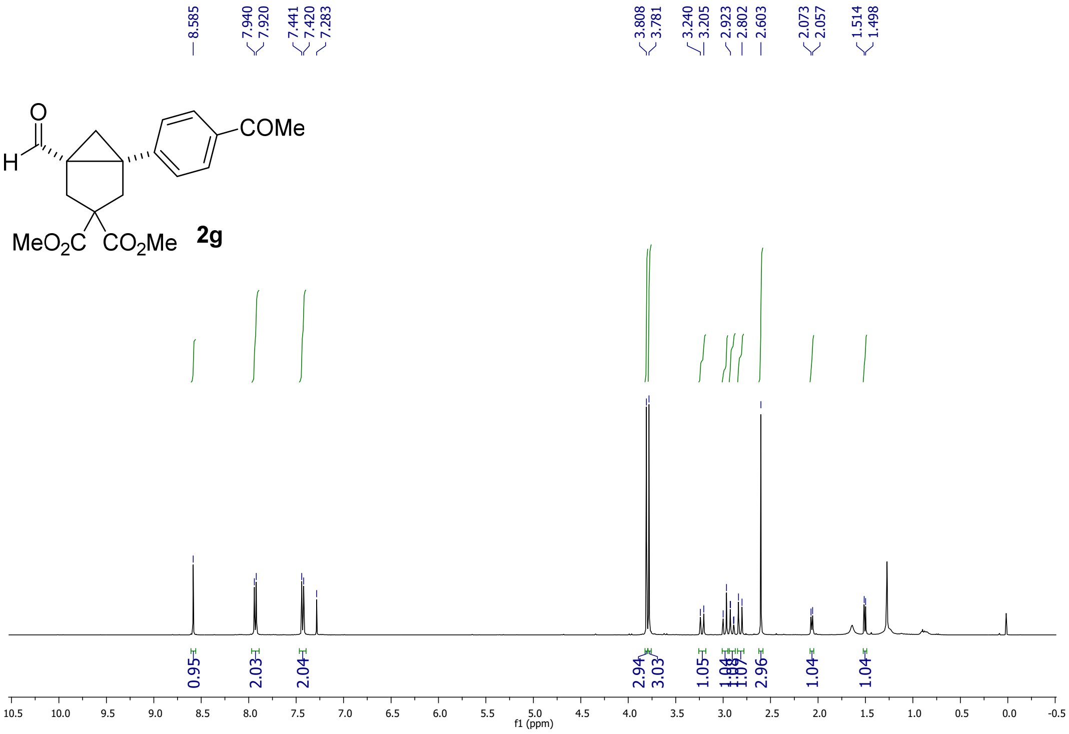

Supplementary Figure 13. ^{1}H NMR of **2g**

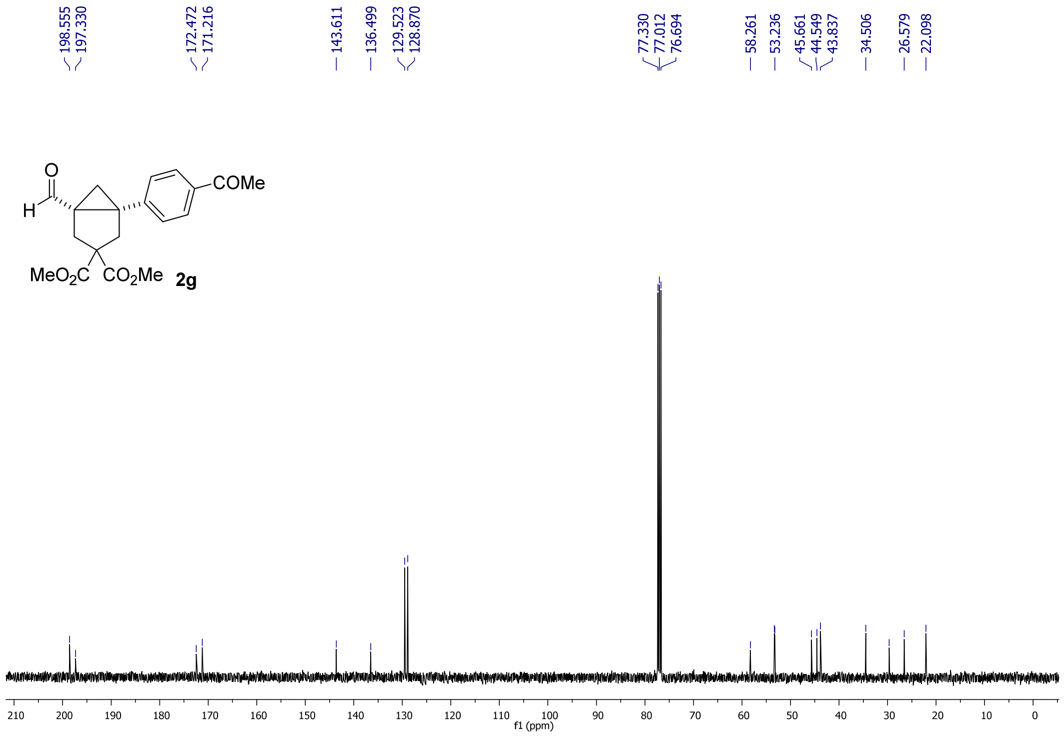

Supplementary Figure 14. ^{13}C NMR of **2g**

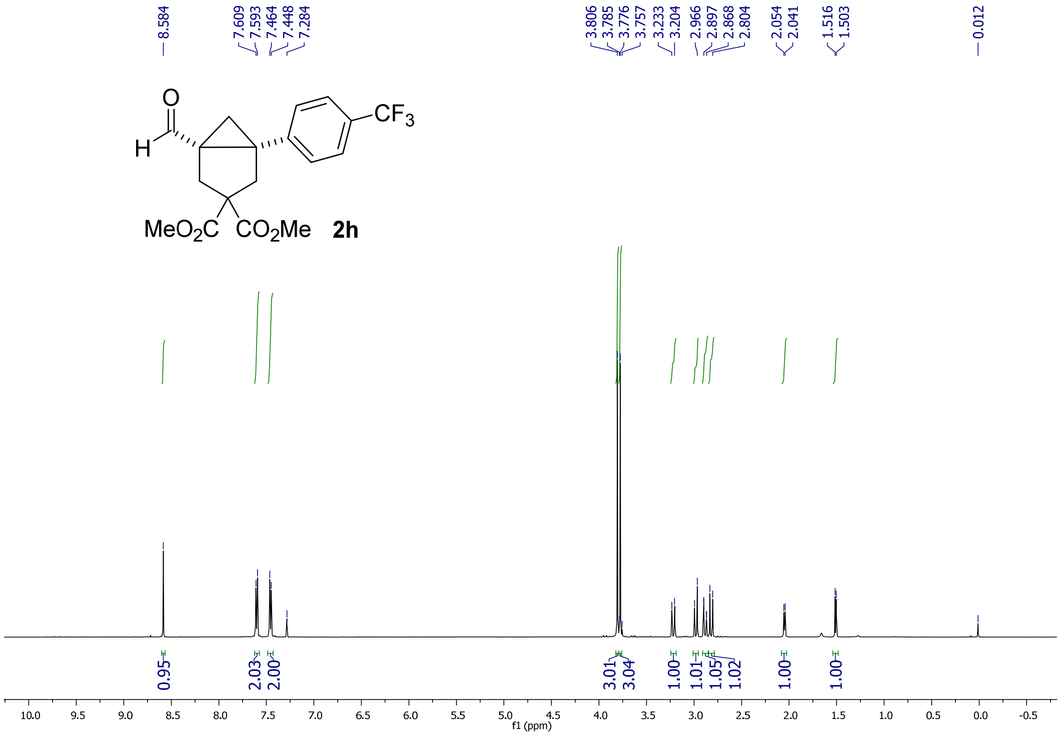

Supplementary Figure 15. ^{1}H NMR of **2h**

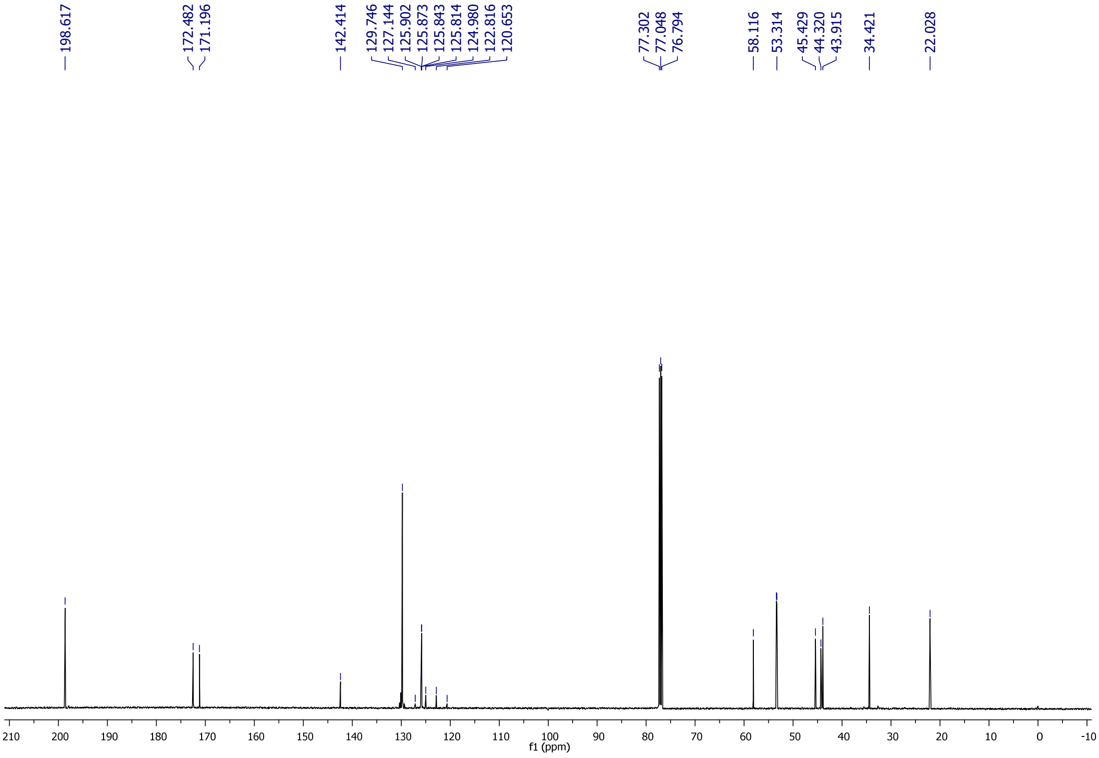

Supplementary Figure 16. ^{13}C NMR of **2h**

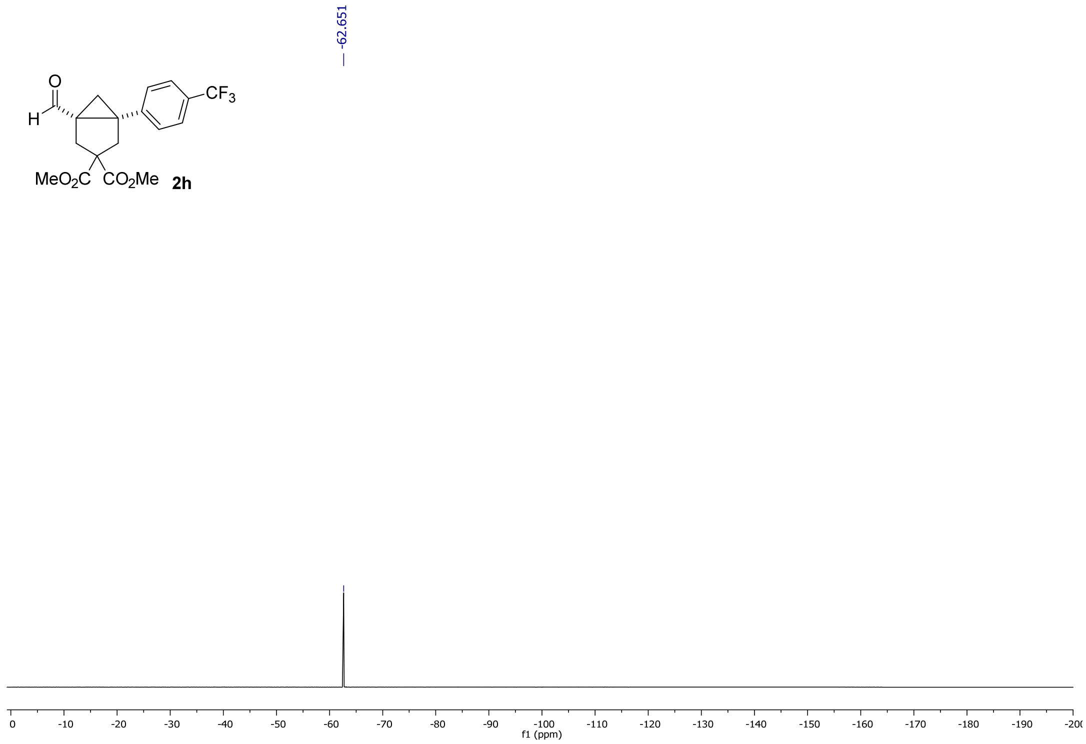

Supplementary Figure 17. ^{19}F NMR of **2h**

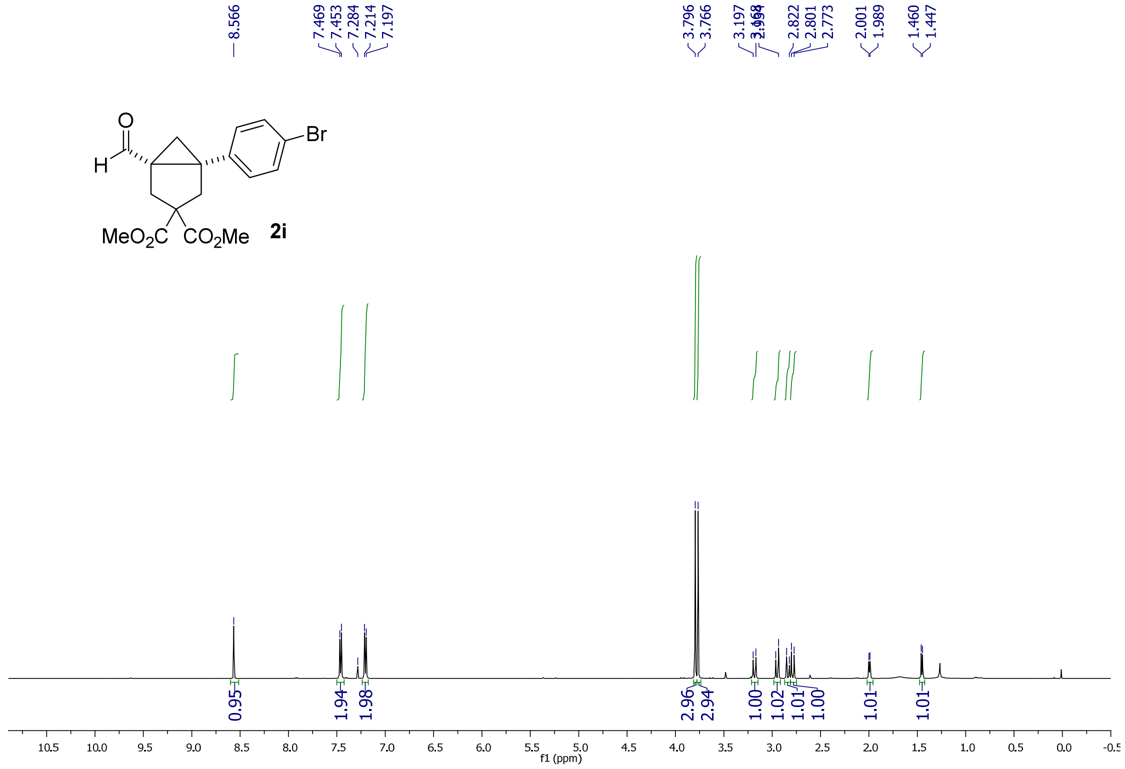

Supplementary Figure 18. ^{1}H NMR of **2i**

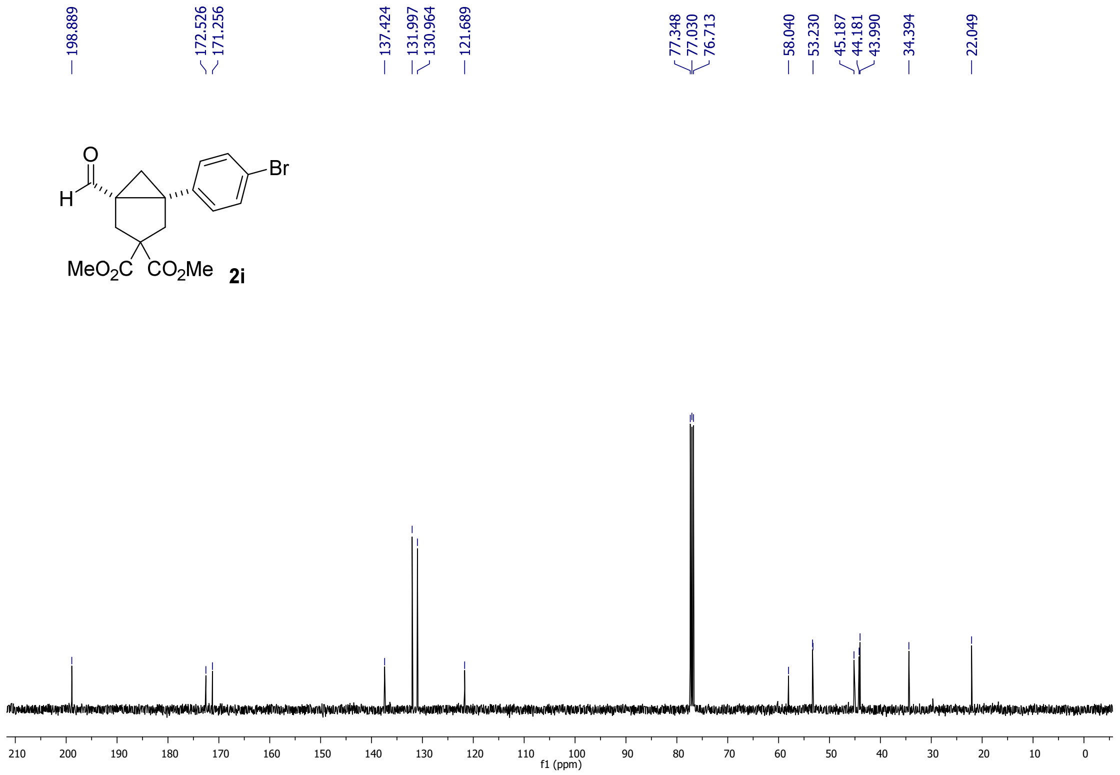

Supplementary Figure 19. ^{13}C NMR of **2i**

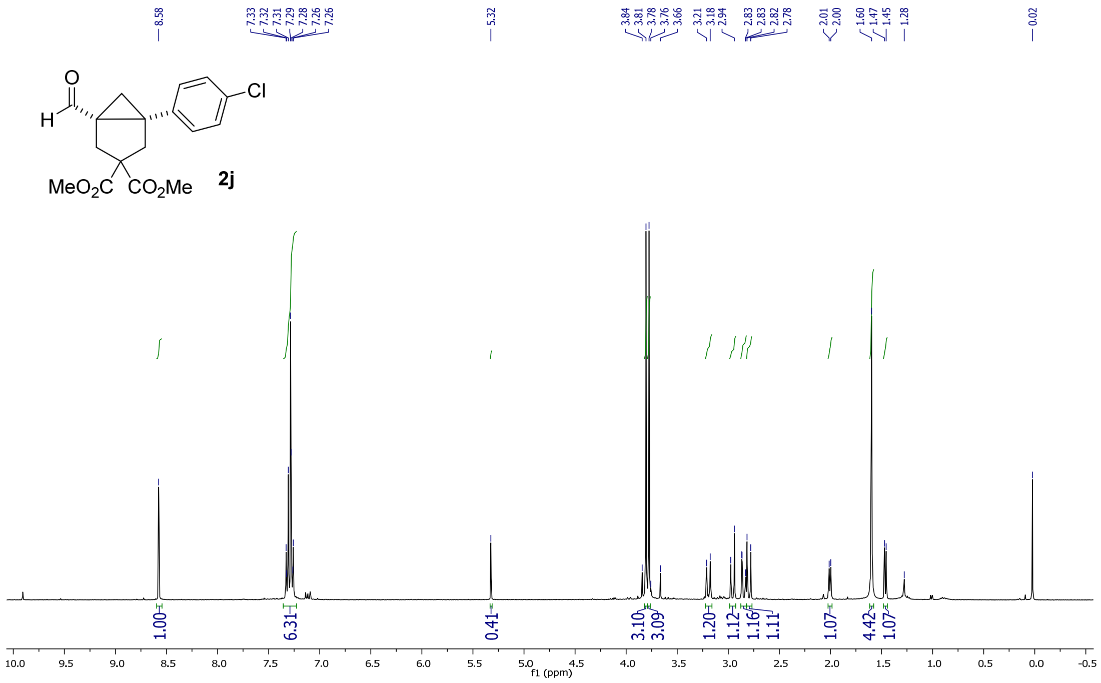

Supplementary Figure 20. ^{1}H NMR of **2j**

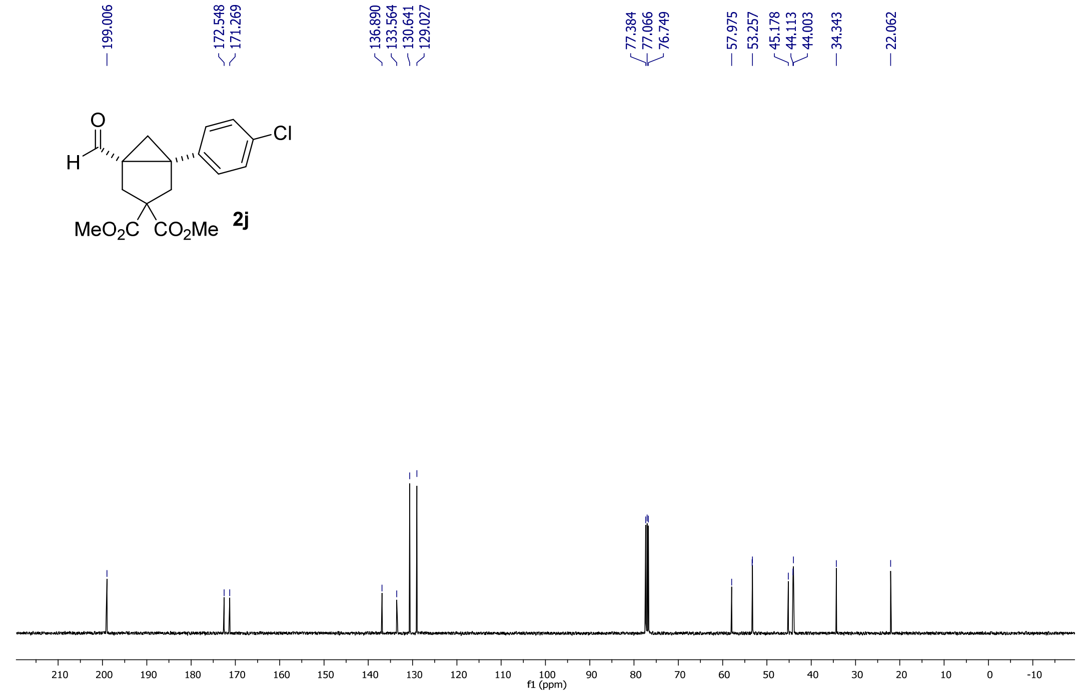

Supplementary Figure 21. ^{13}C NMR of **2j**

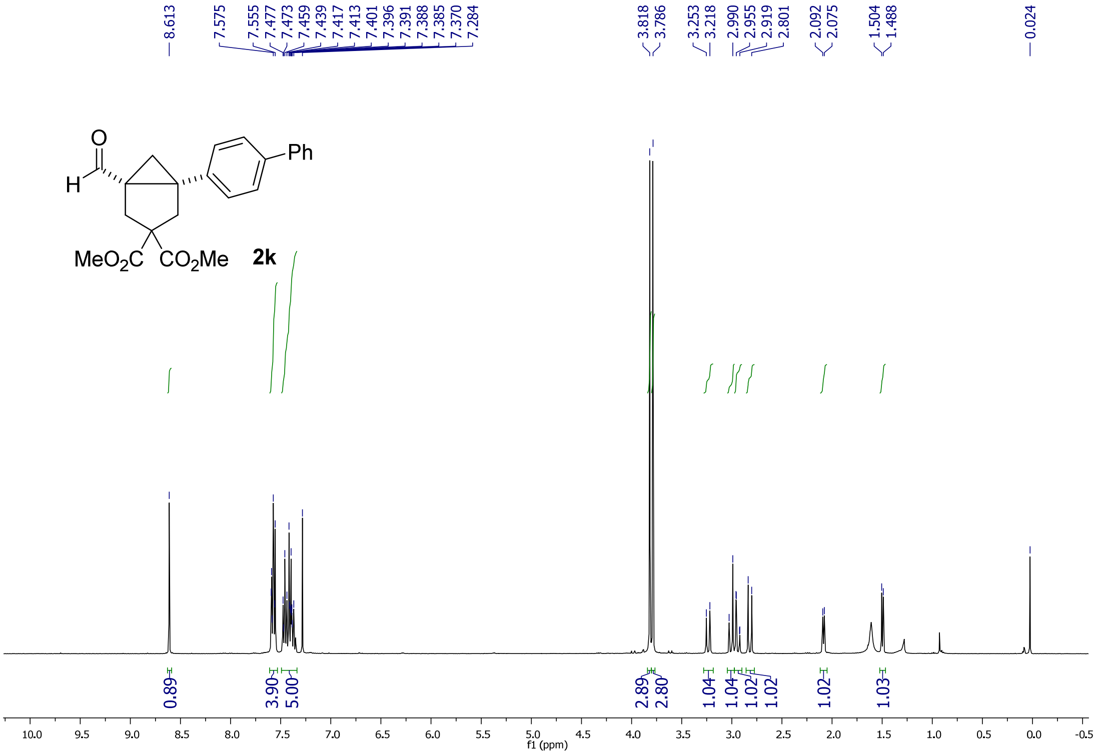

Supplementary Figure 22. ^{1}H NMR of **2k**

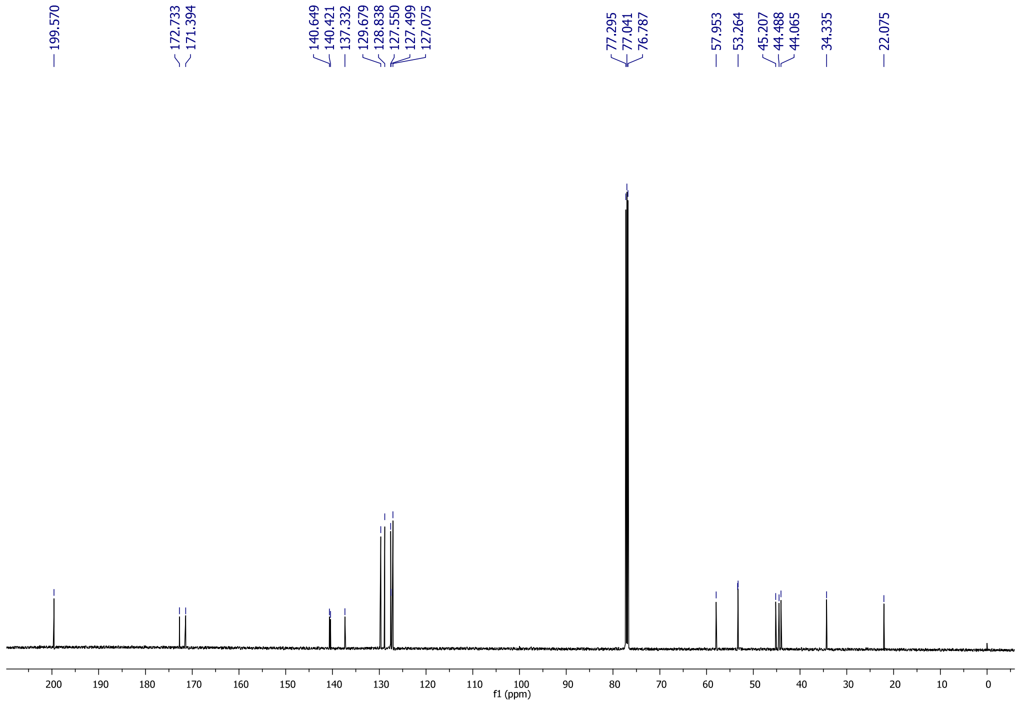

Supplementary Figure 23. ^{13}C NMR of **2k**

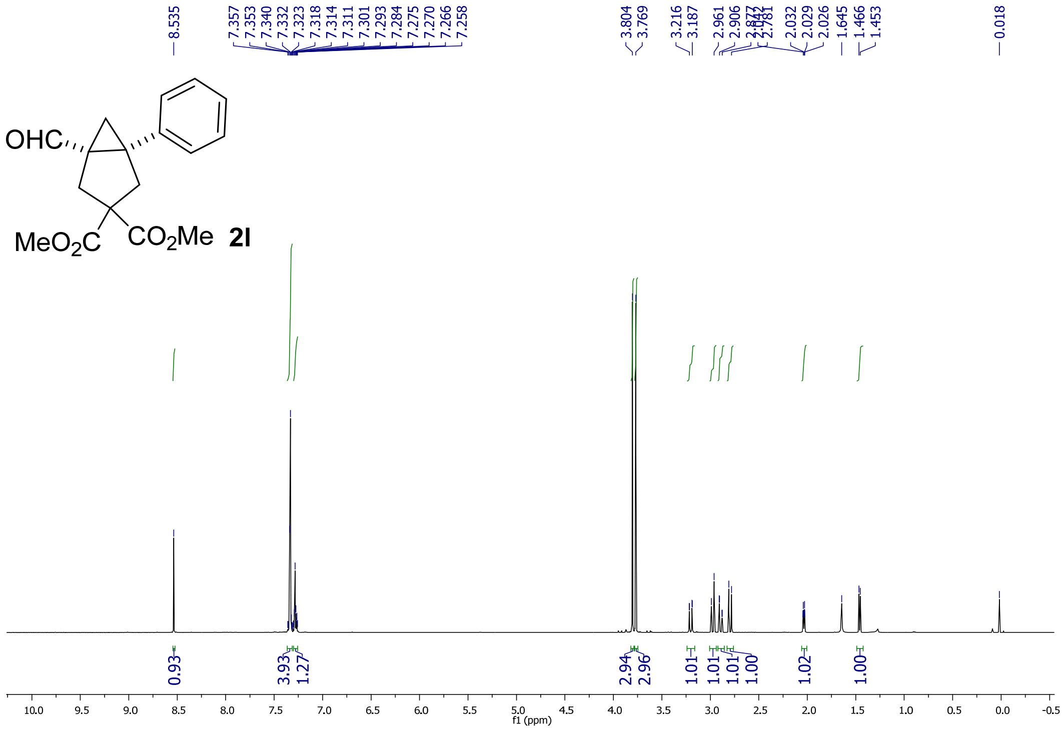

Supplementary Figure 24. ^{1}H NMR of **2l**

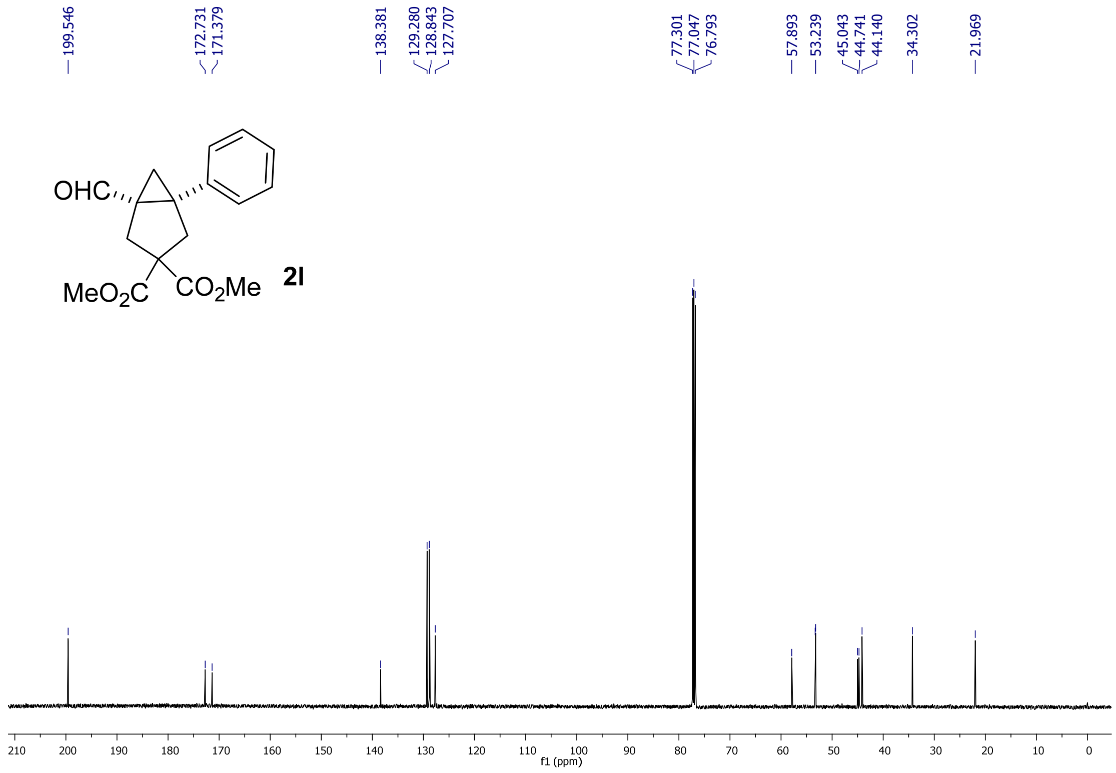

Supplementary Figure 25. ^{13}C NMR of **2l**

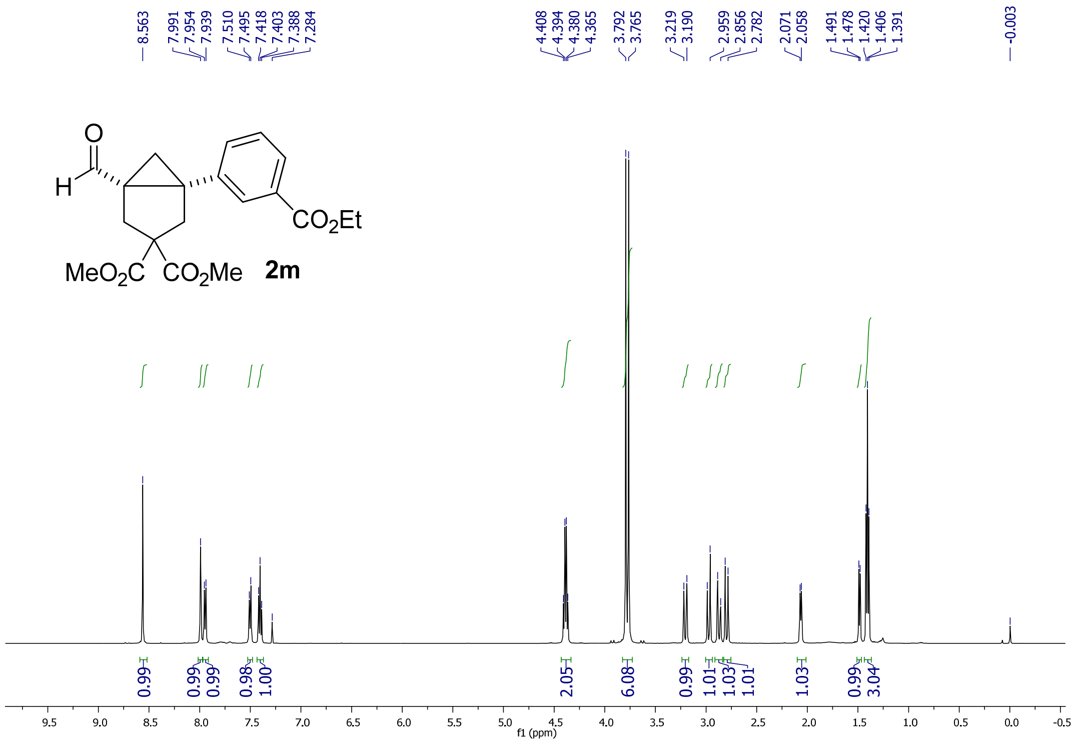

Supplementary Figure 26. ^{1}H NMR of **2m**

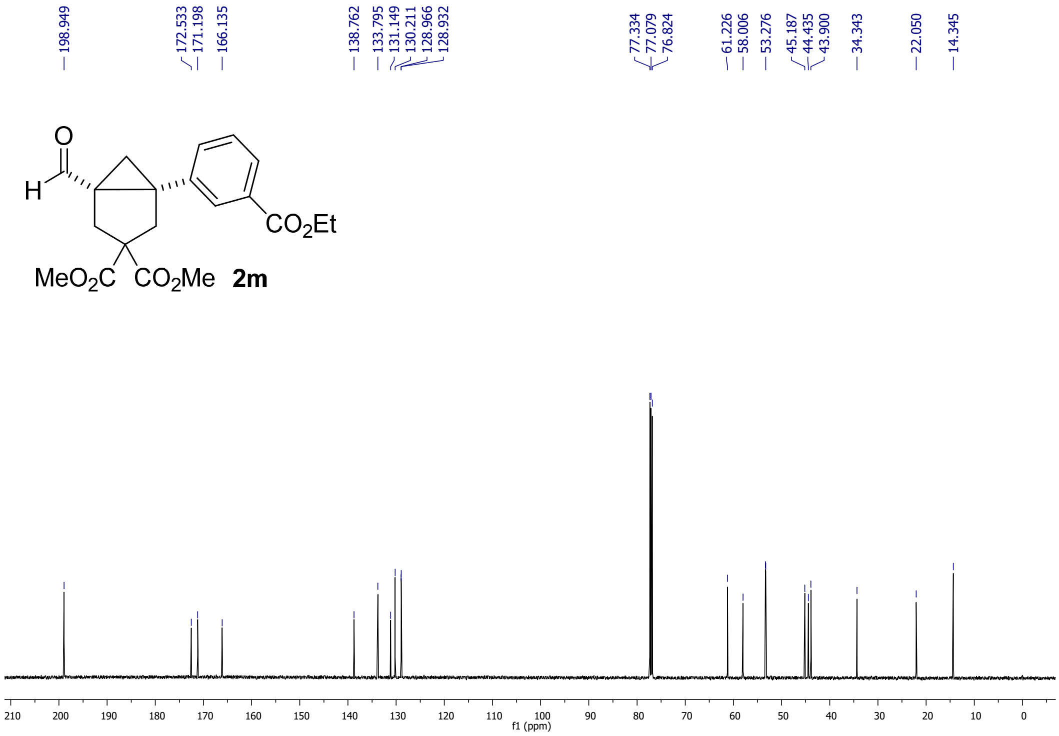

Supplementary Figure 27. ^{13}C NMR of **2m**

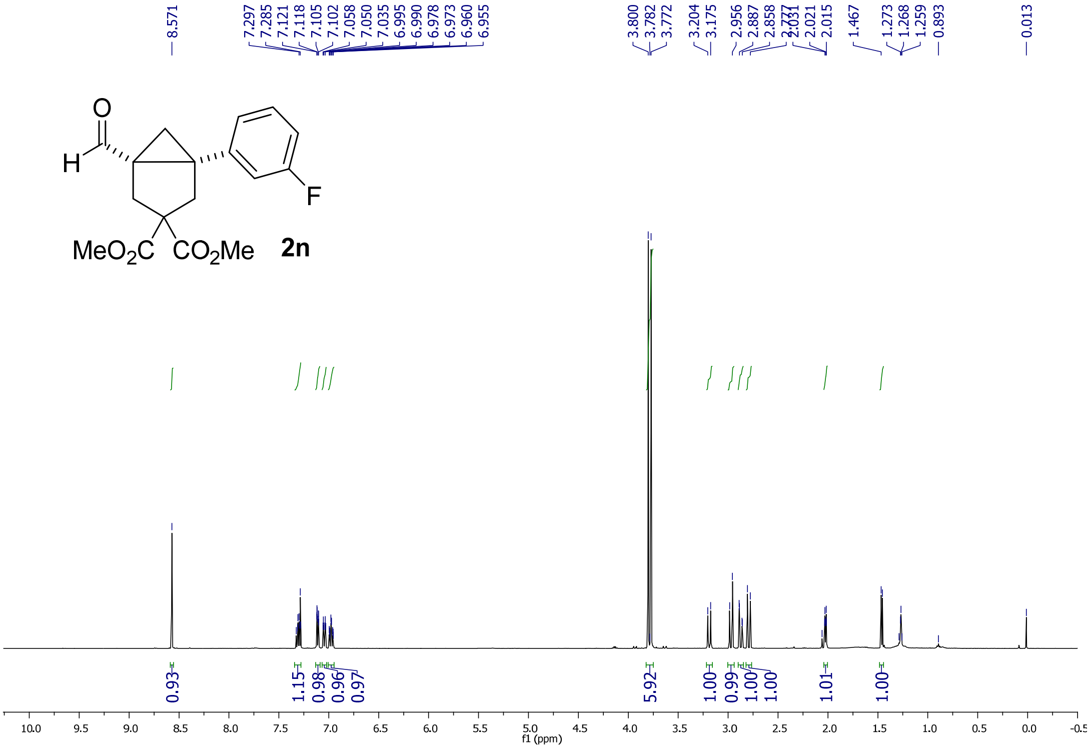

Supplementary Figure 28. ^{1}H NMR of **2n**

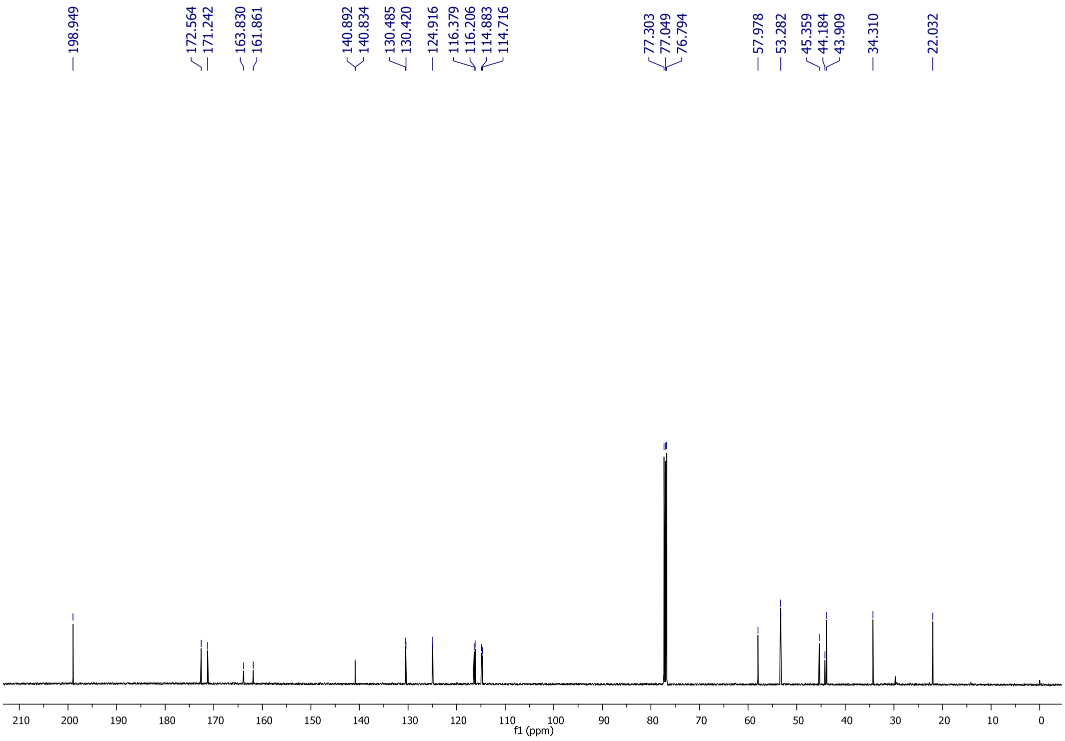

Supplementary Figure 29. ^{13}C NMR of **2n**

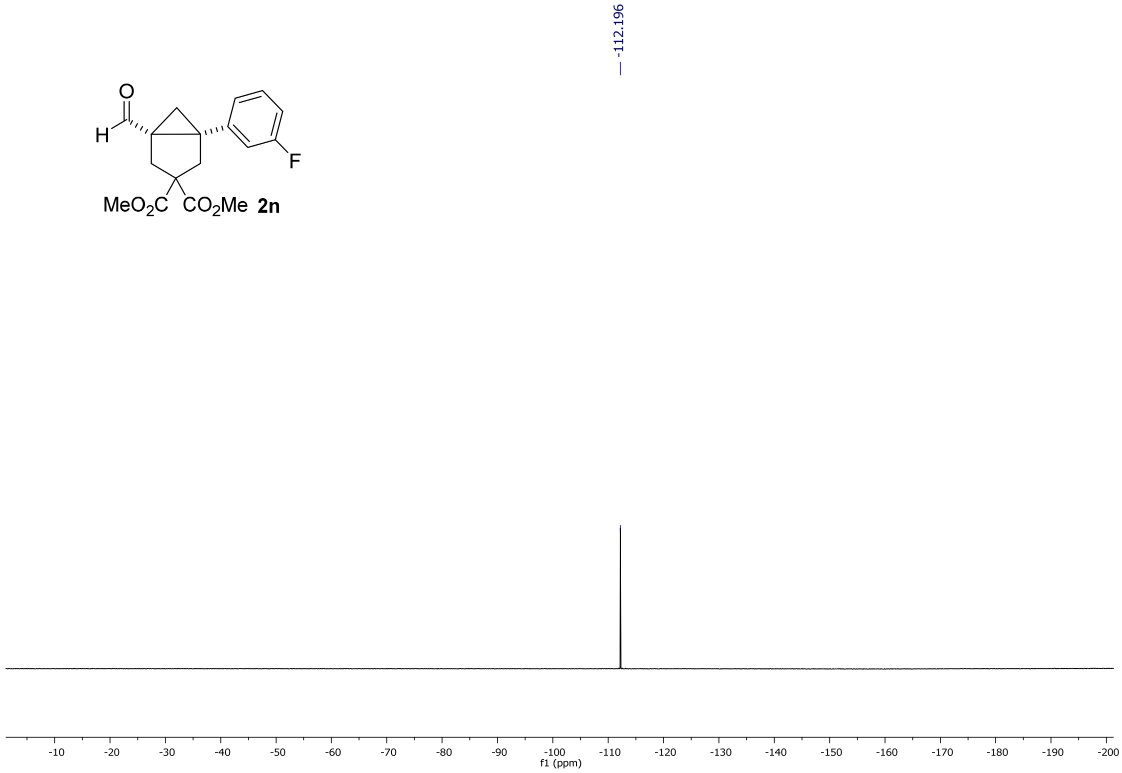

Supplementary Figure 30. ^{19}F NMR of **2n**

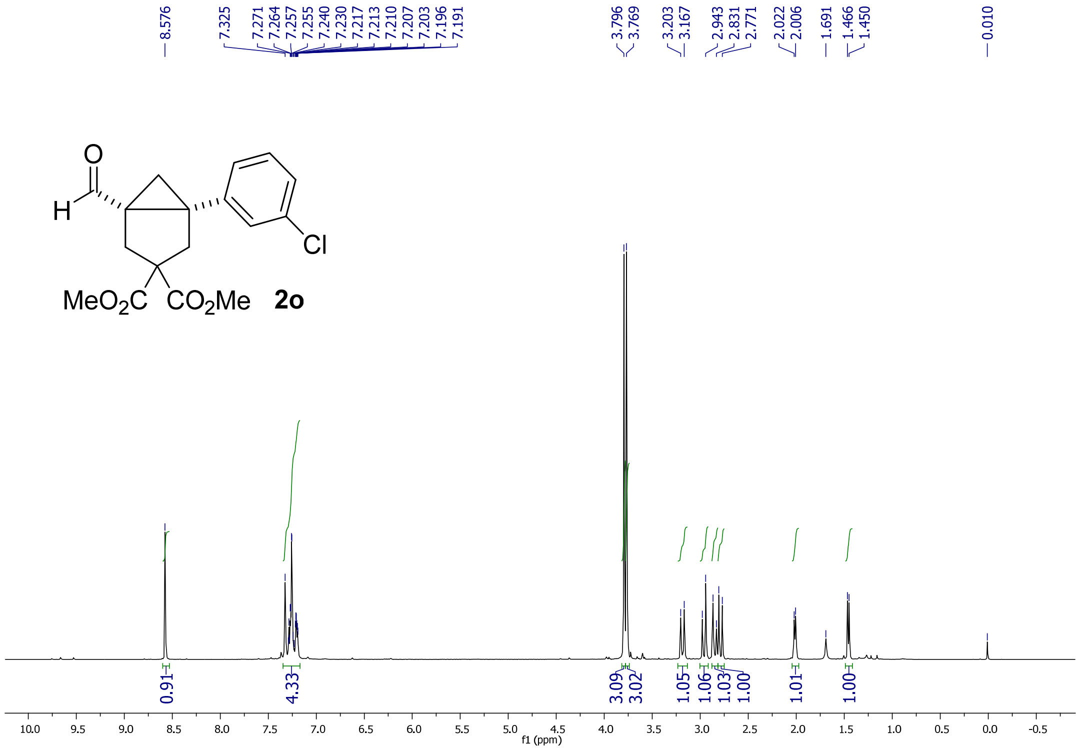

Supplementary Figure 31. ^{1}H NMR of **2o**

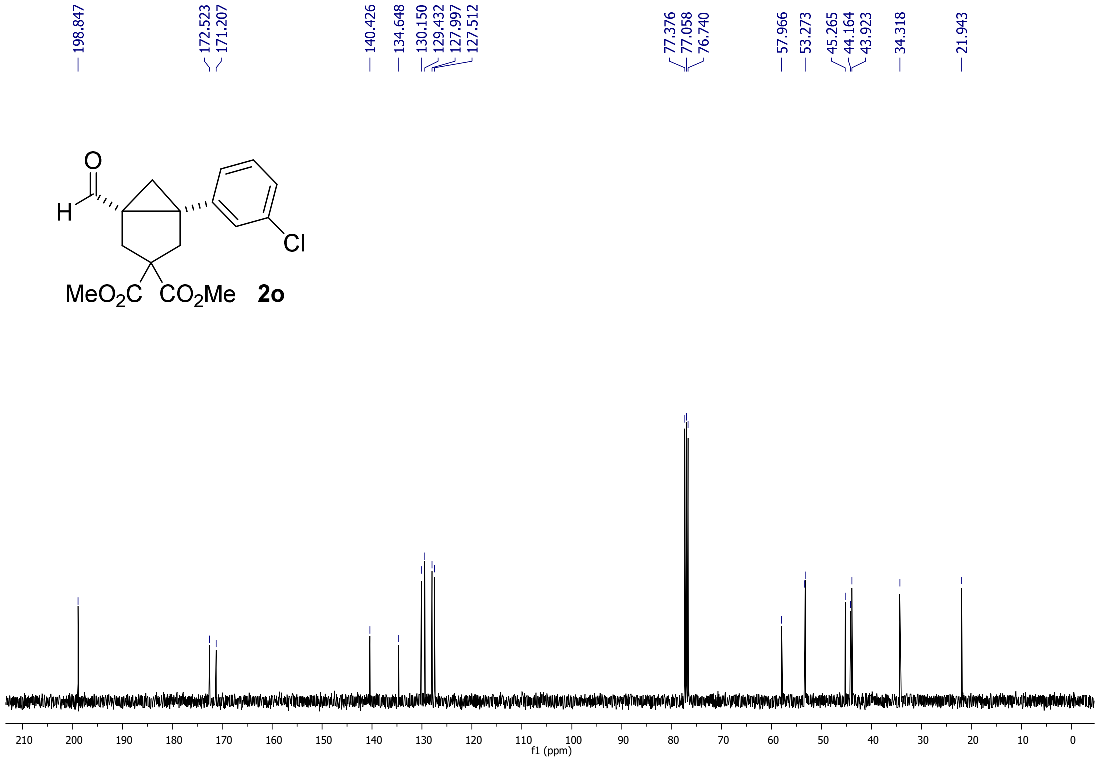

Supplementary Figure 32. ^{13}C NMR of **2o**

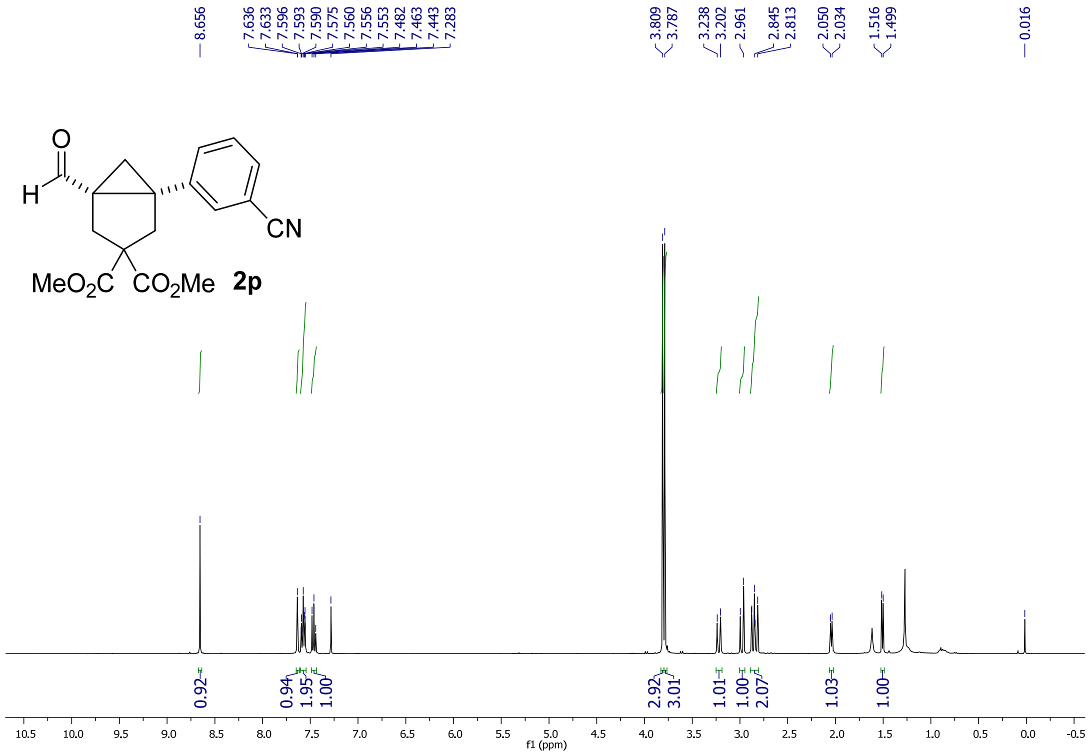

Supplementary Figure 33. ^{1}H NMR of **2p**

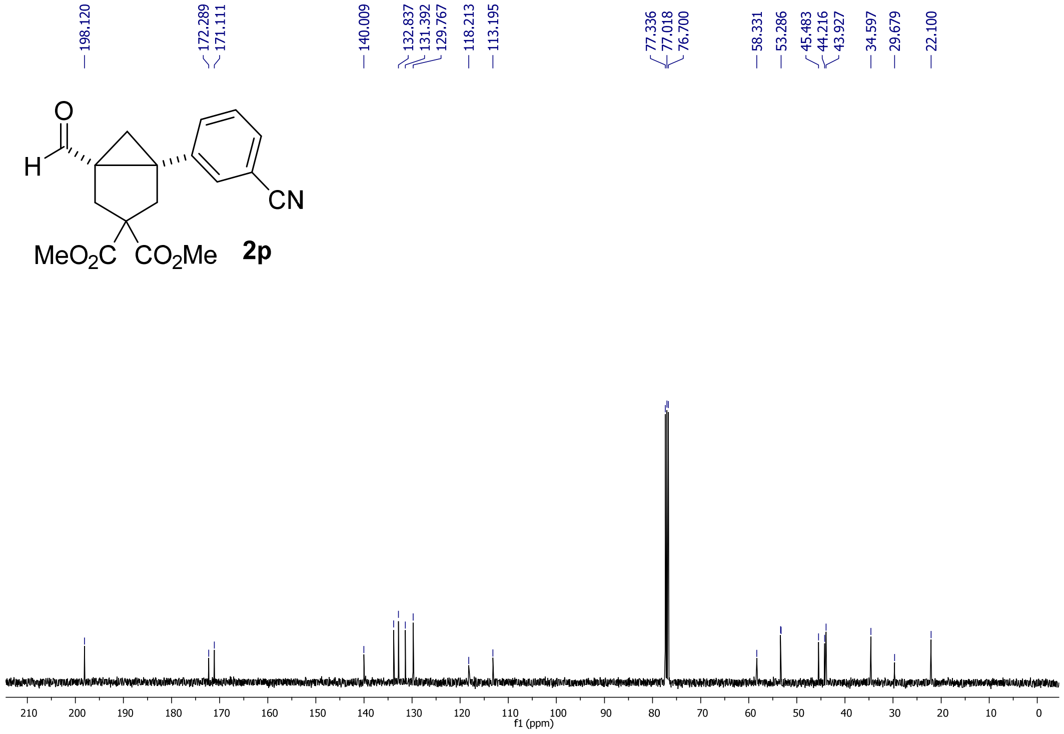

Supplementary Figure 34. ^{13}C NMR of **2p**

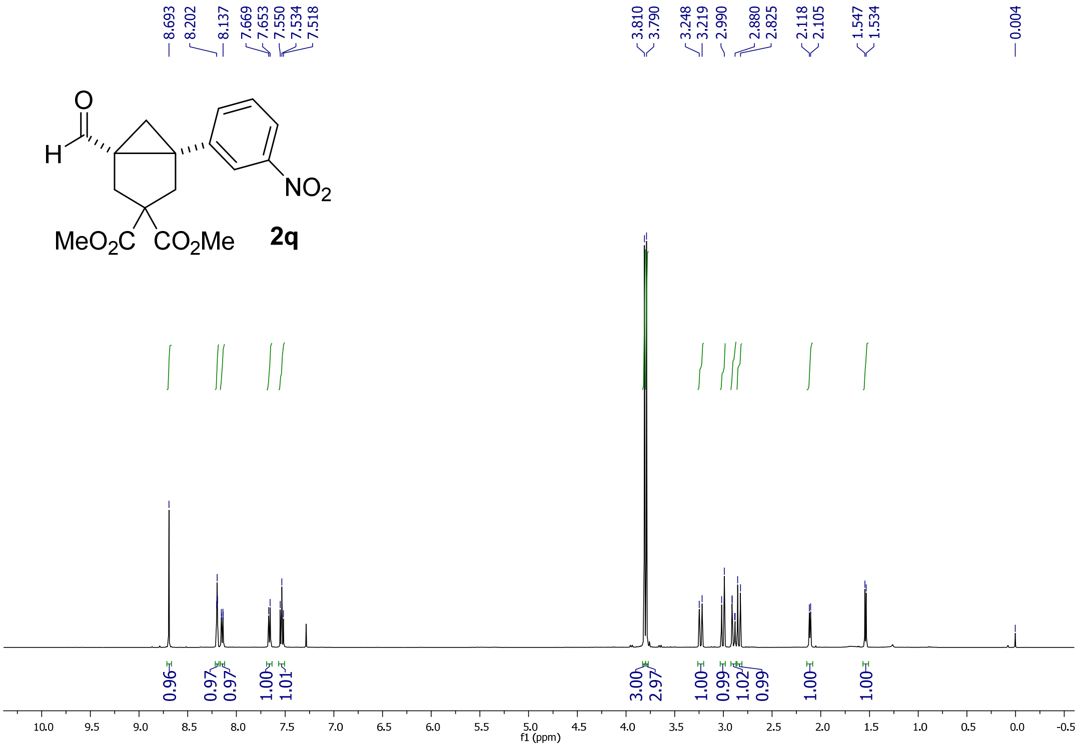

Supplementary Figure 35. ^{1}H NMR of **2q**

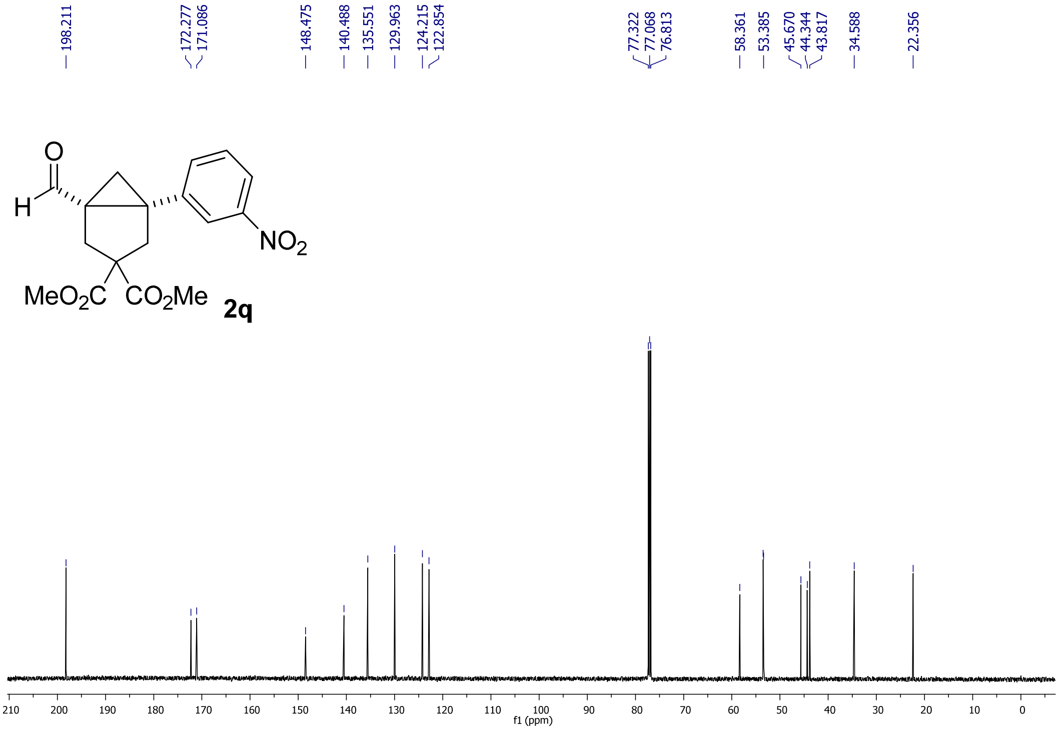

Supplementary Figure 36. ^{13}C NMR of **2q**

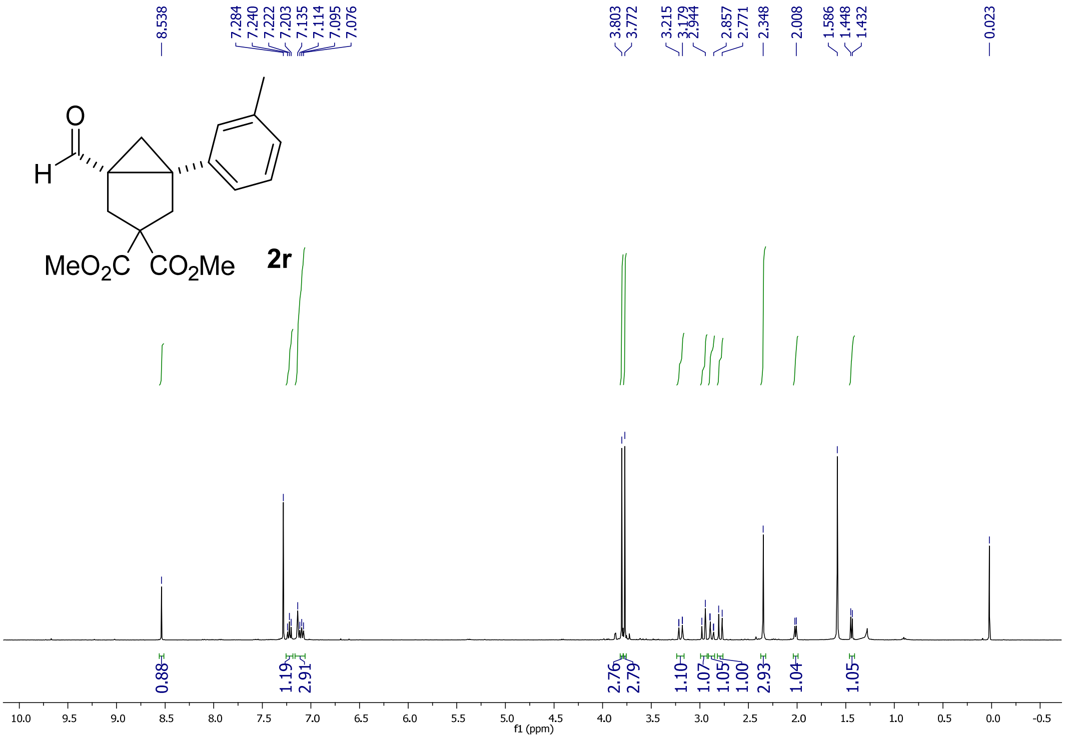

Supplementary Figure 37. ^{1}H NMR of **2r**

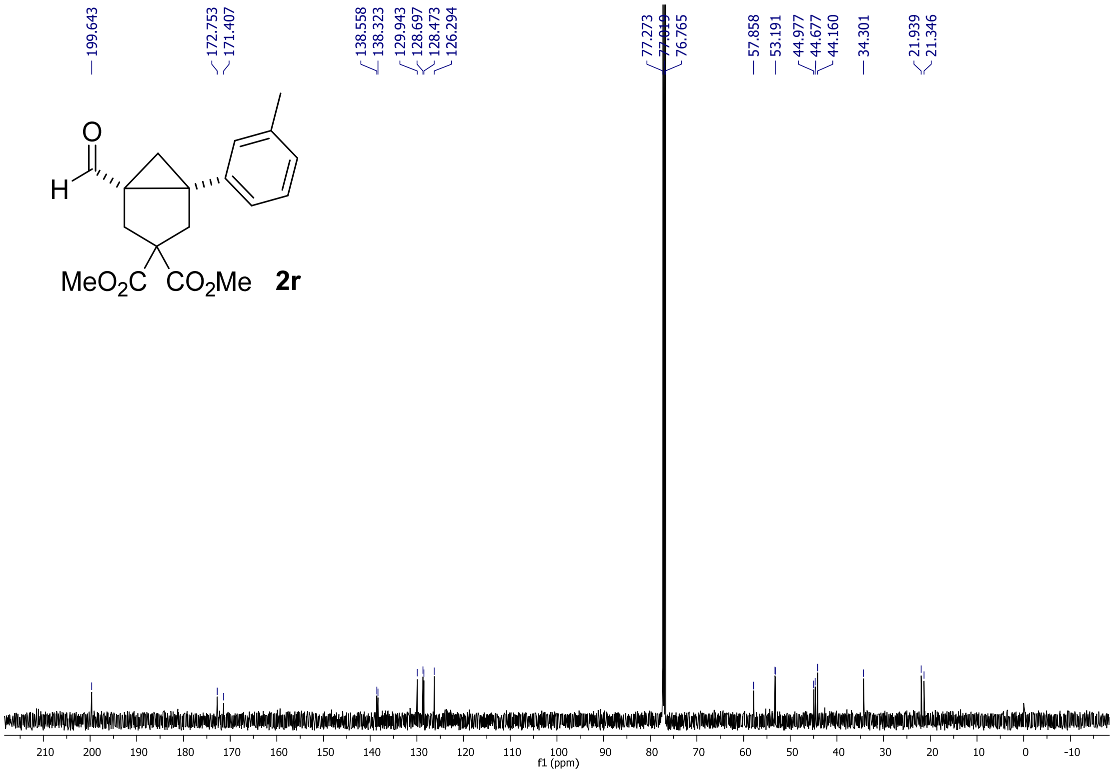

Supplementary Figure 38. ^{13}C NMR of **2r**

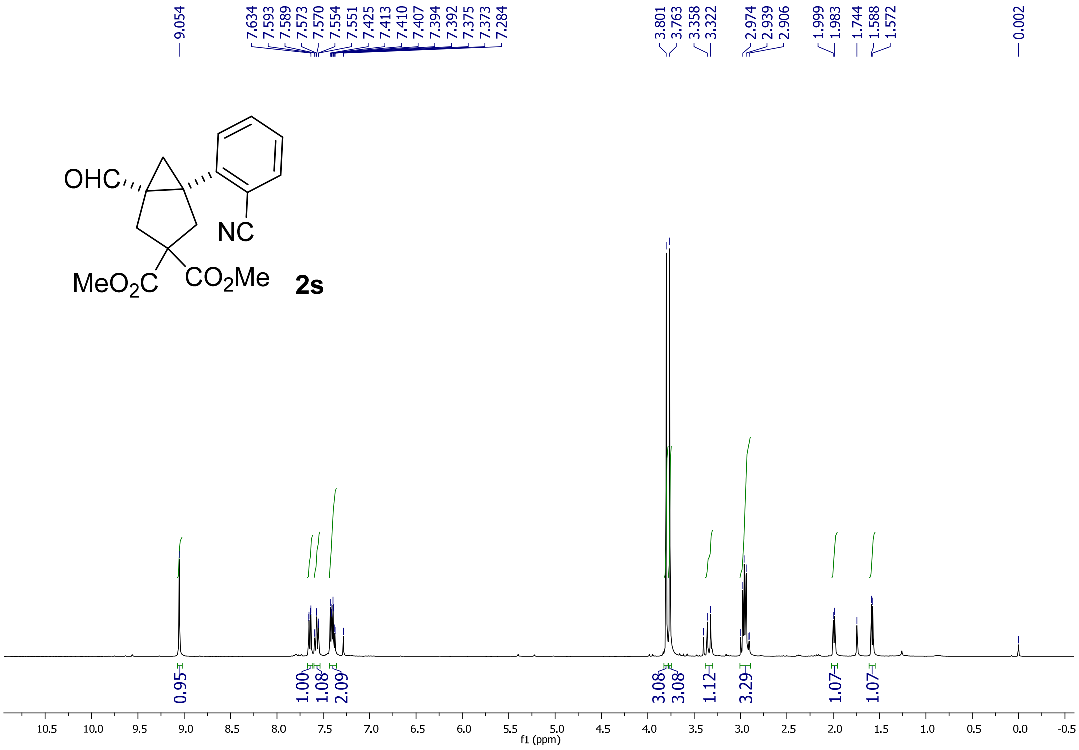

Supplementary Figure 39. ^{1}H NMR of **2s**

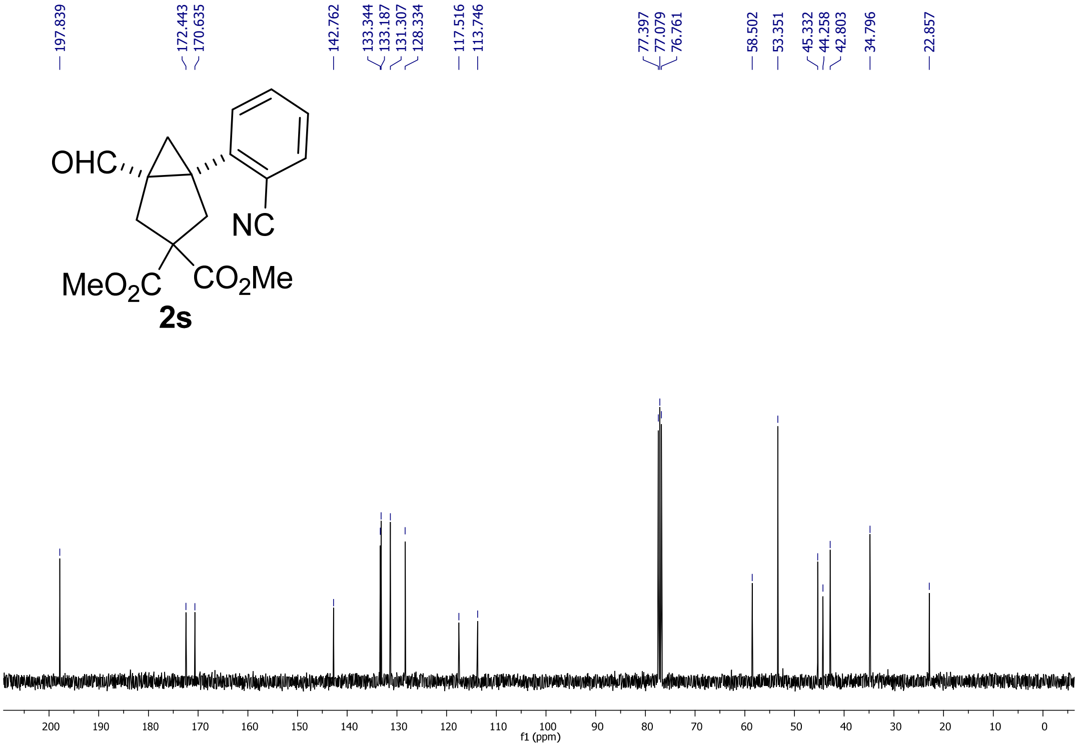

Supplementary Figure 40. ^{13}C NMR of **2s**

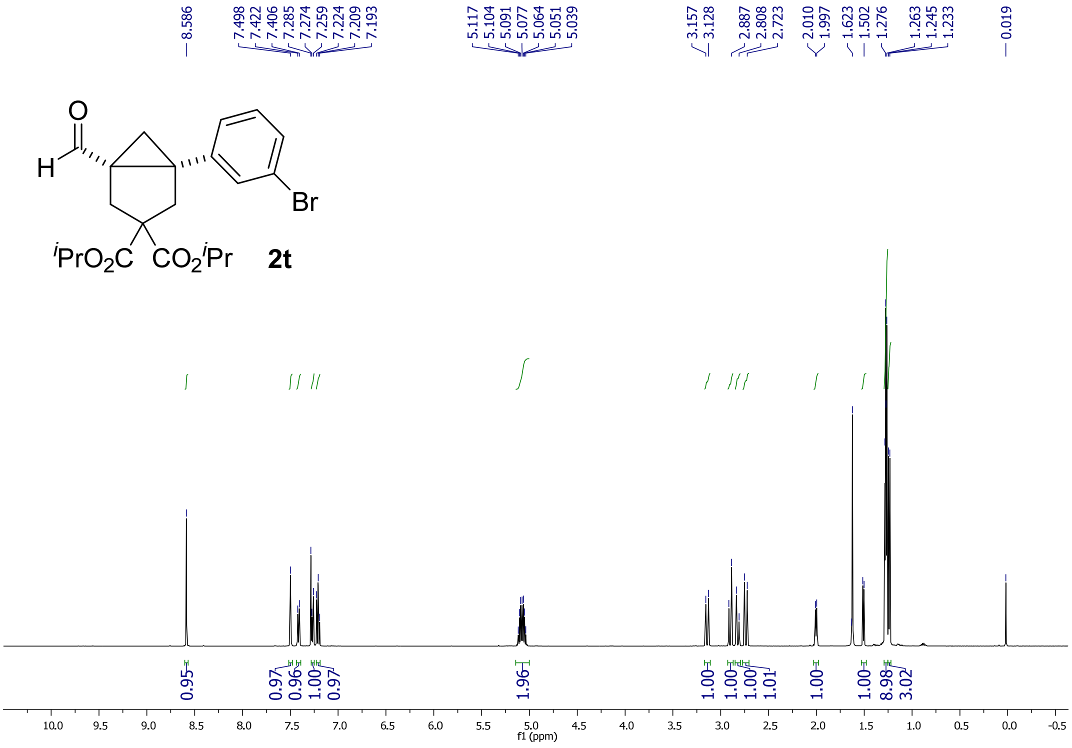

Supplementary Figure 41. ^{1}H NMR of **2t**

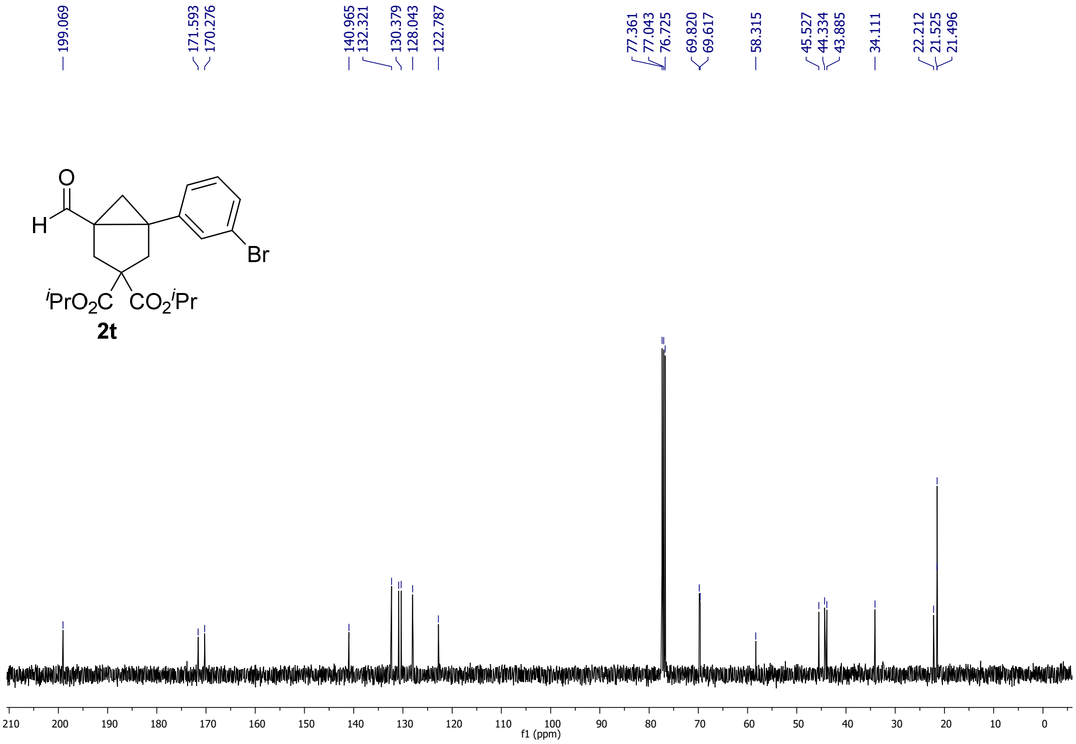

Supplementary Figure 42. ^{13}C NMR of **2t**

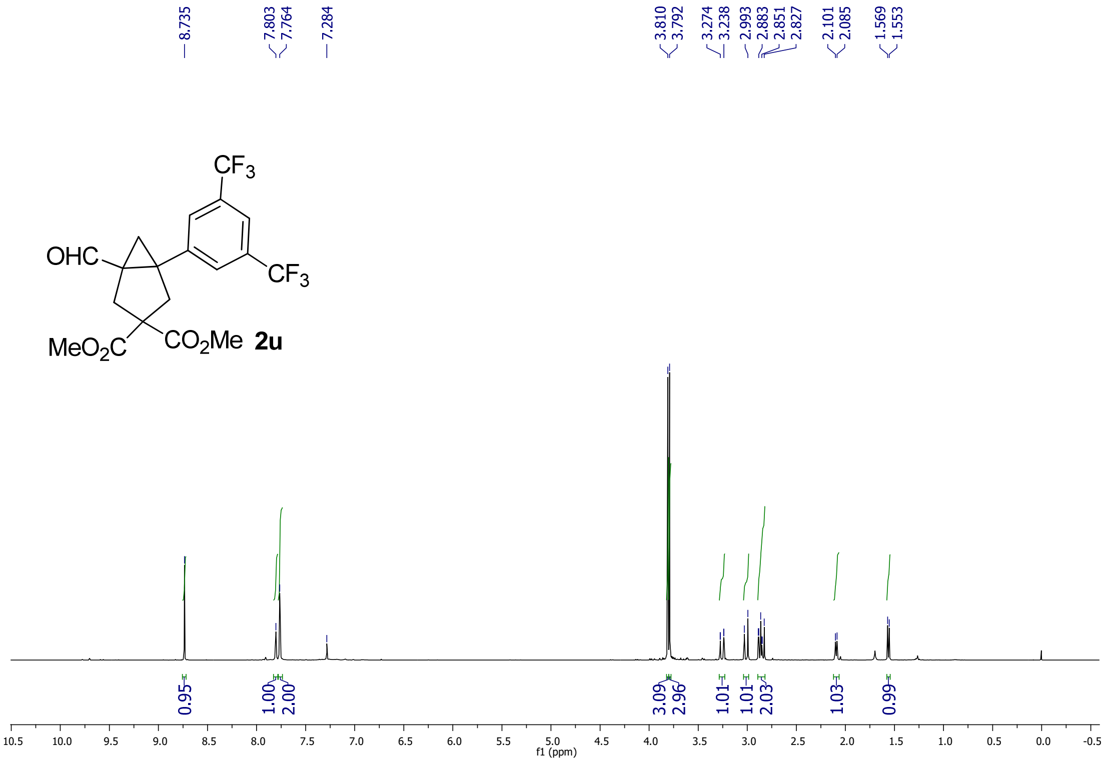

Supplementary Figure 43. ^{1}H NMR of **2u**

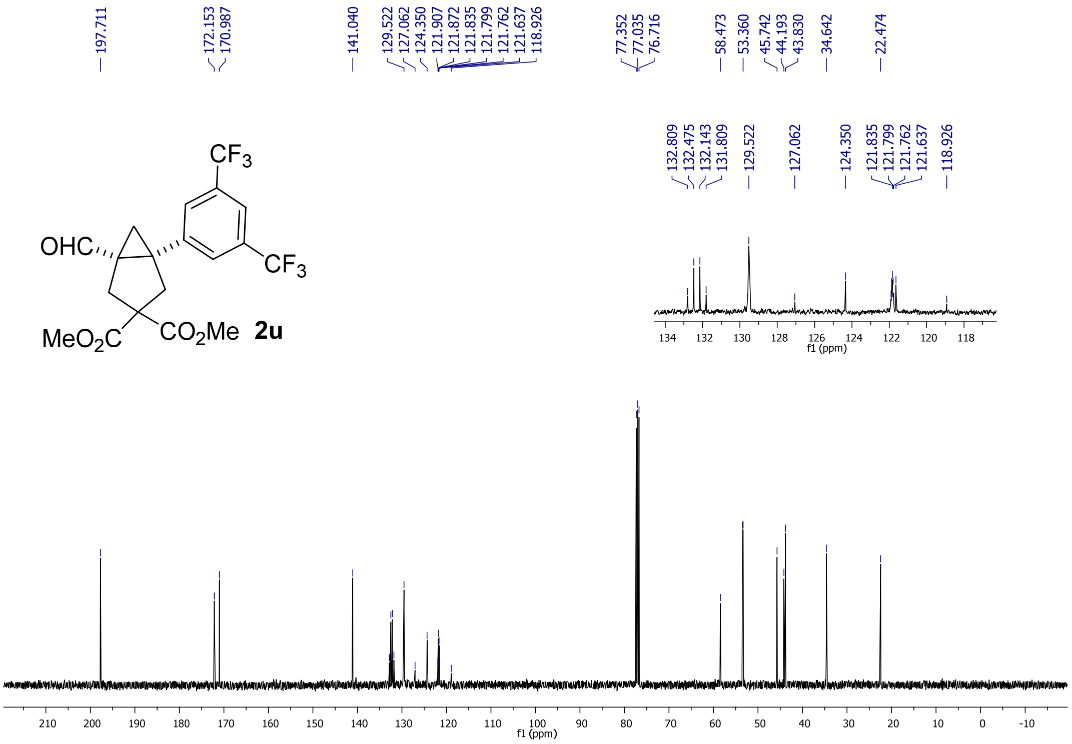

Supplementary Figure 44. ^{13}C NMR of **2t**

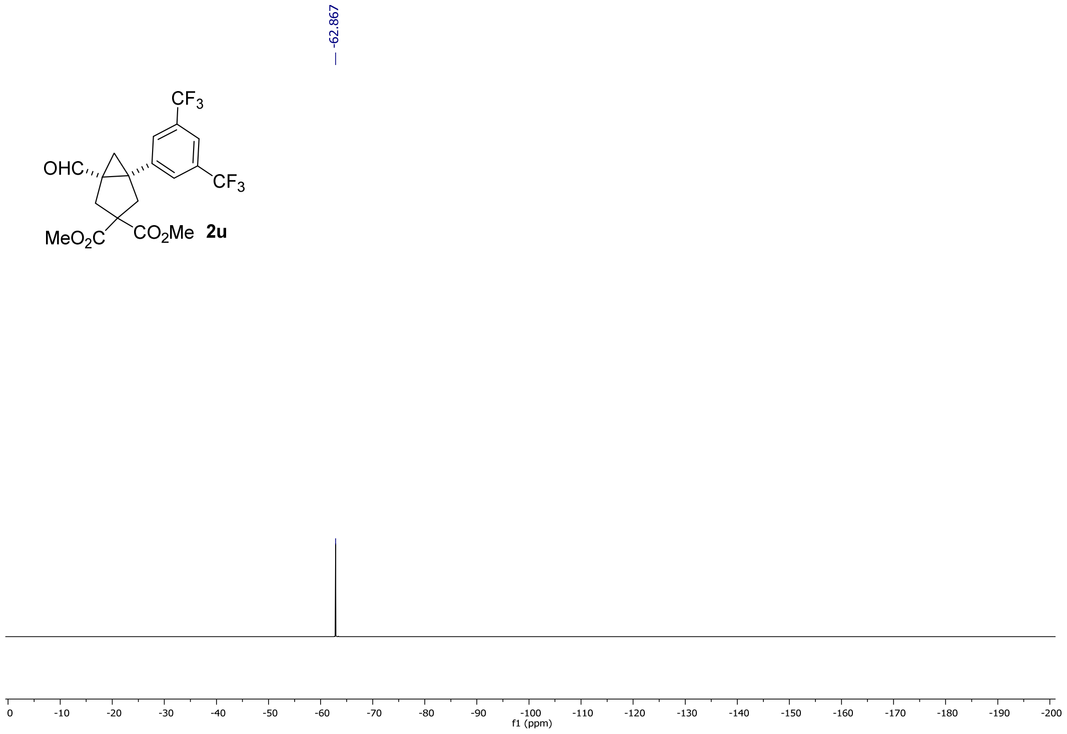

Supplementary Figure 45. ^{19}F NMR of **2u**

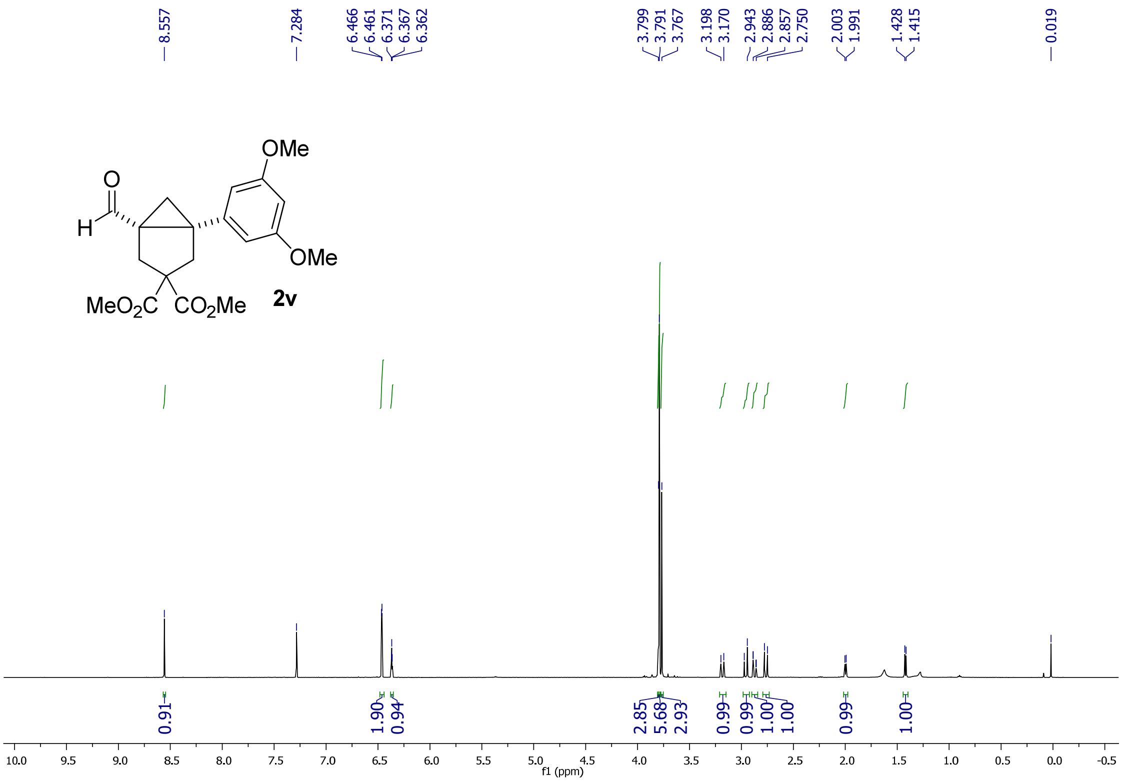

Supplementary Figure 46. ^{1}H NMR of **2v**

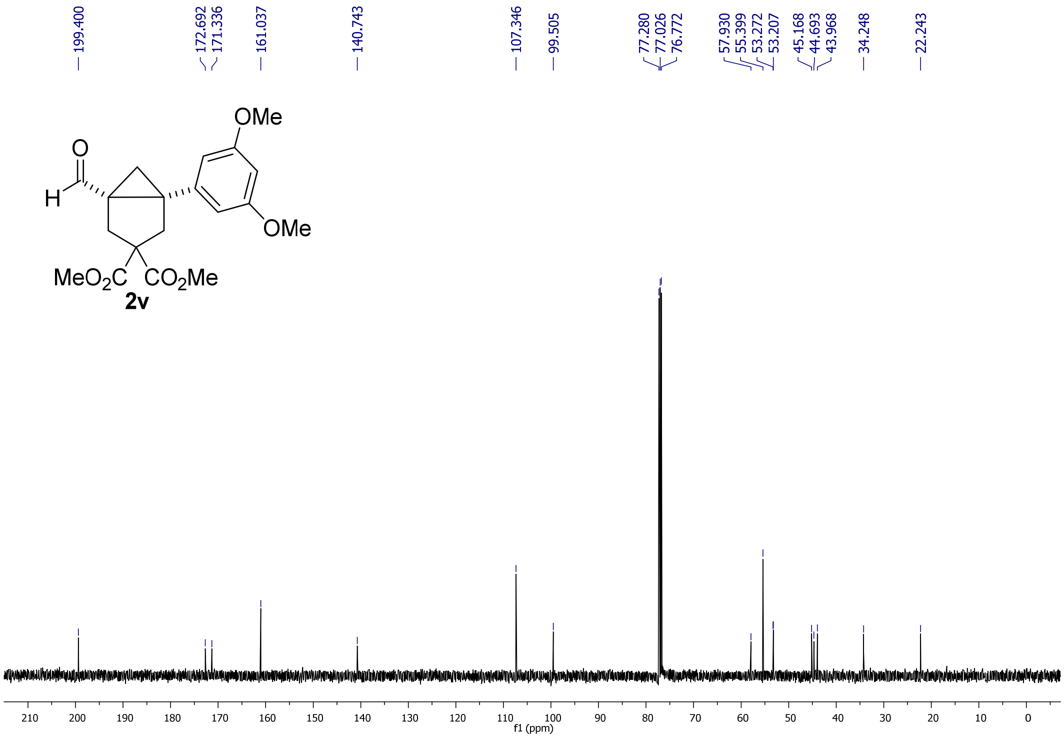

Supplementary Figure 47. ^{13}C NMR of **2v**

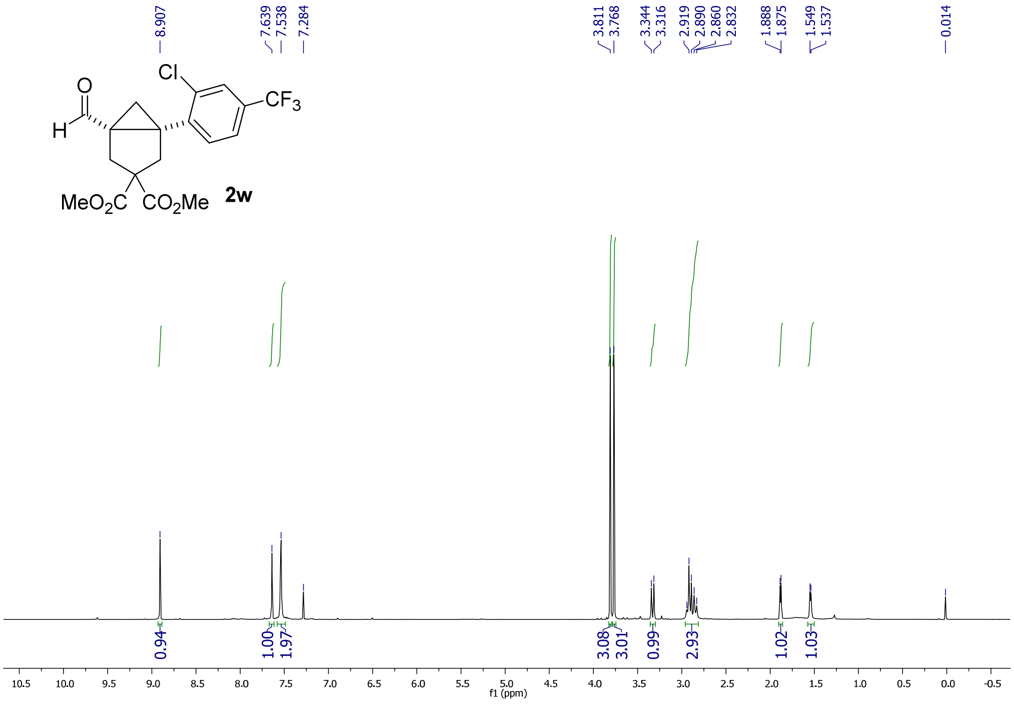

Supplementary Figure 48. ^{1}H NMR of **2w**

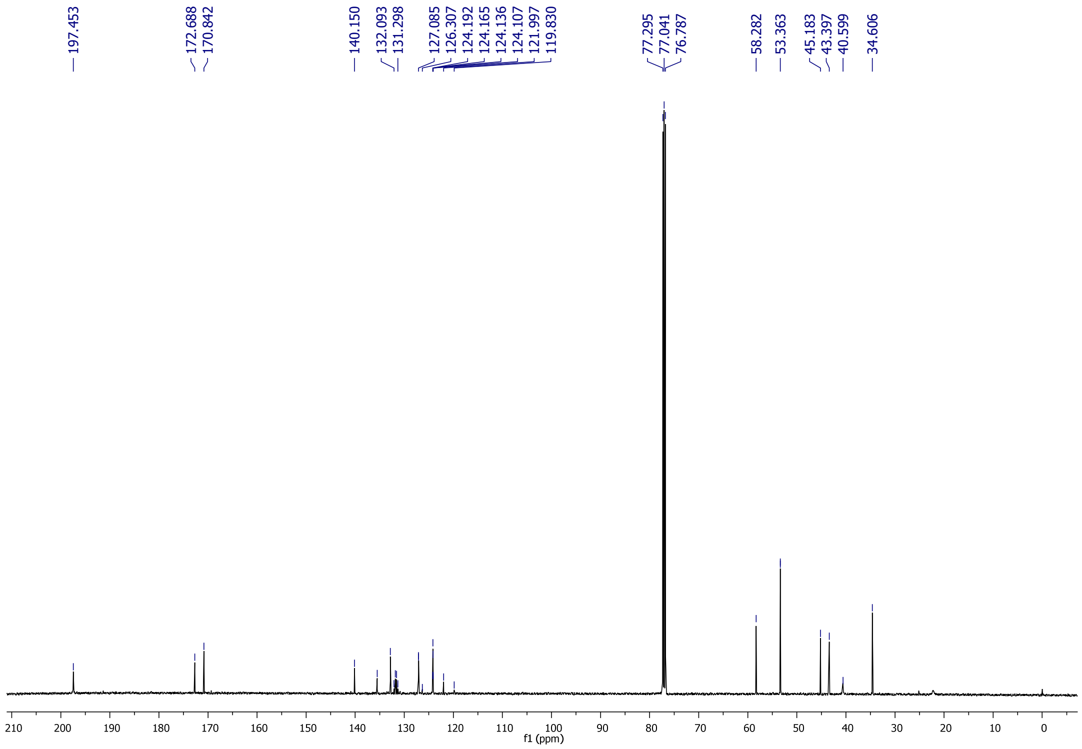

Supplementary Figure 49. ^{13}C NMR of **2w**

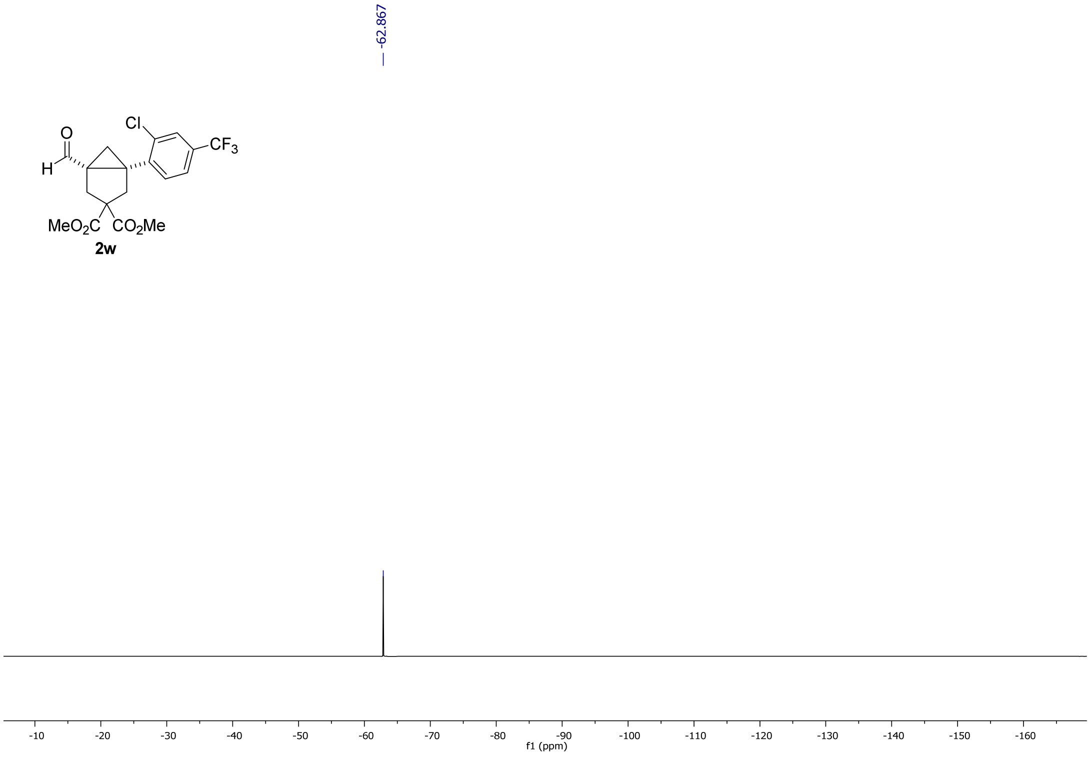

Supplementary Figure 50. ^{19}F NMR of **2w**

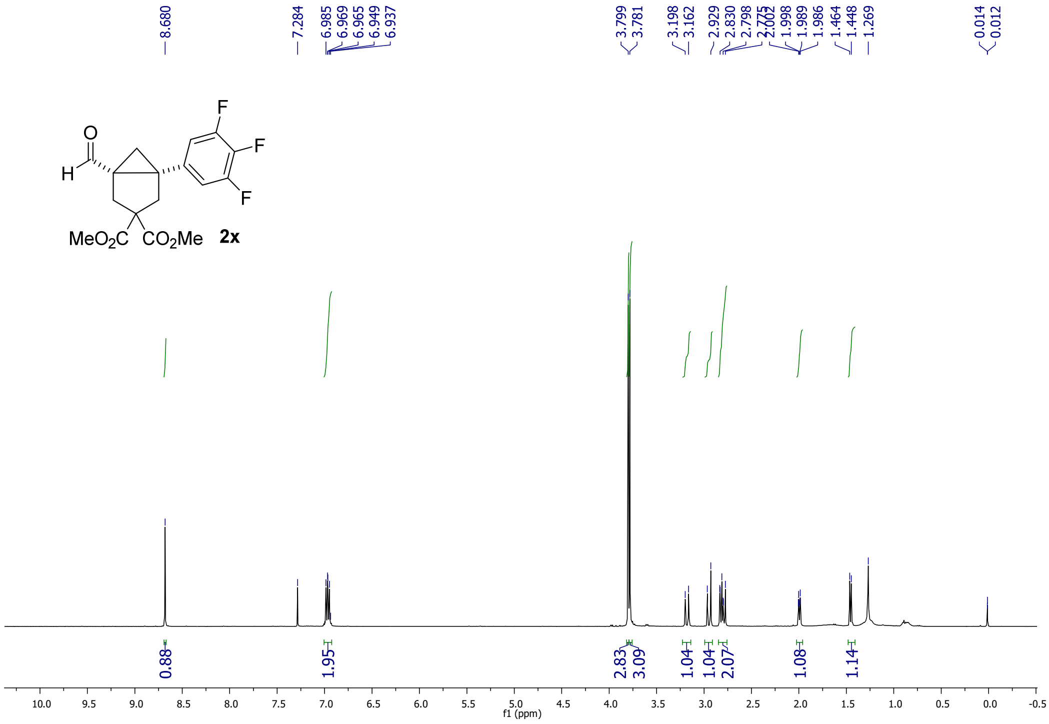

Supplementary Figure 48. ^{1}H NMR of **2x**

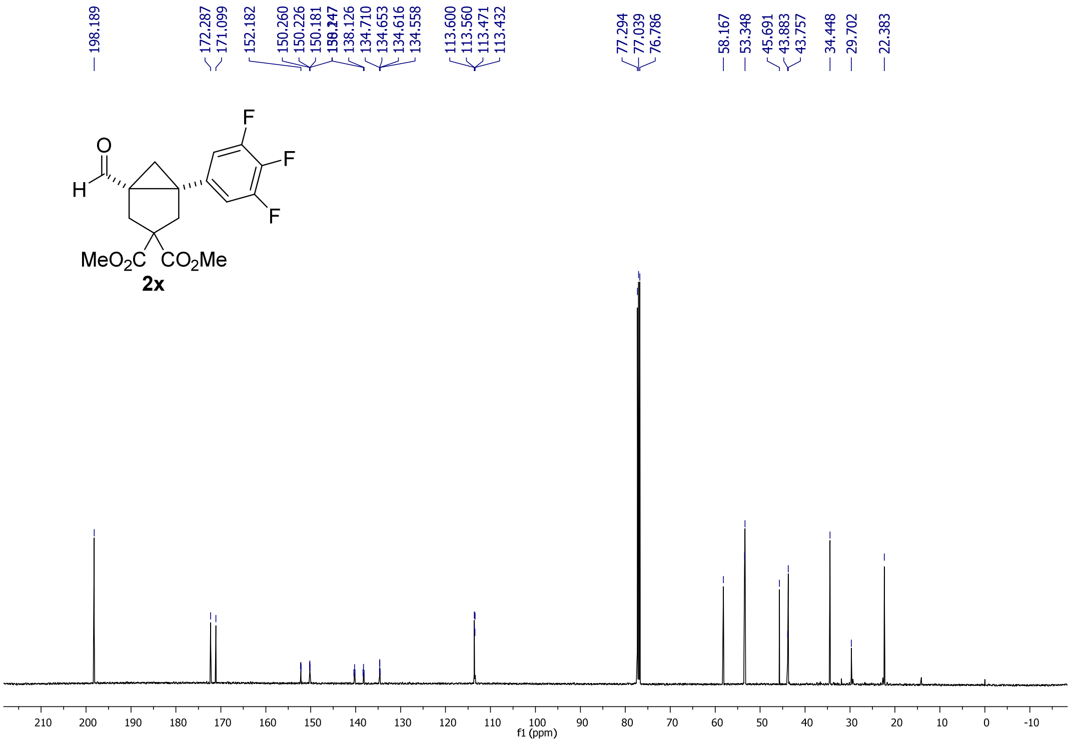

Supplementary Figure 52. ^{13}C NMR of **2x**

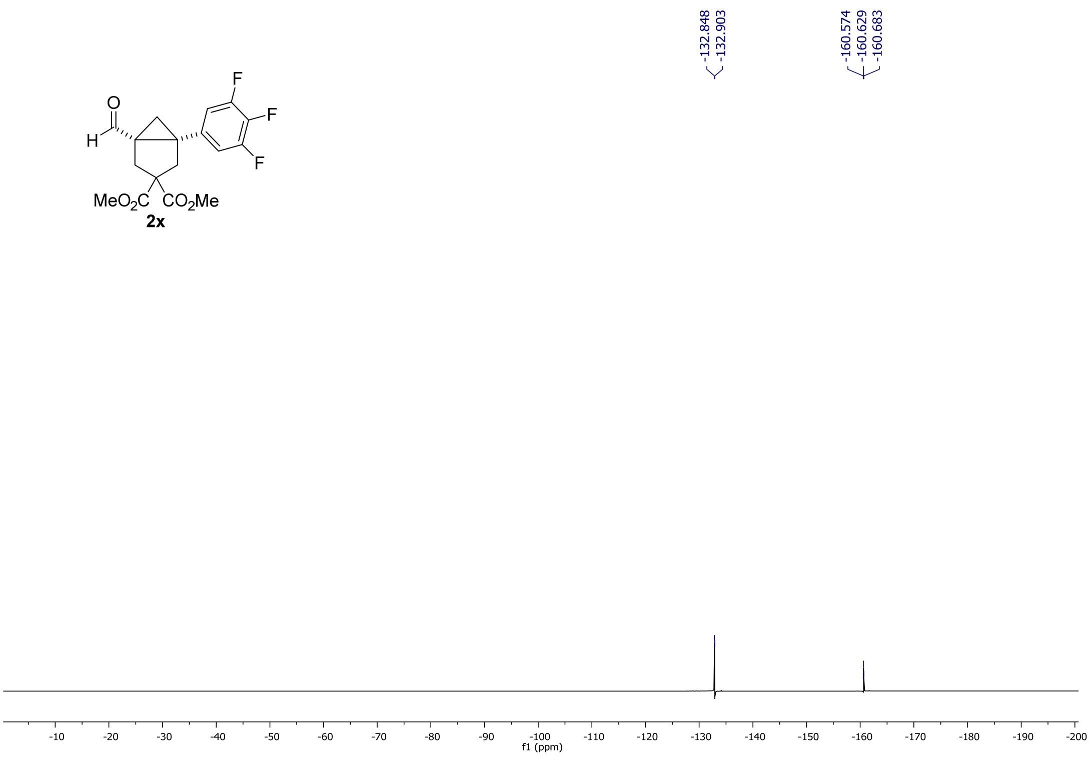

Supplementary Figure 53. ^{19}F NMR of **2x**

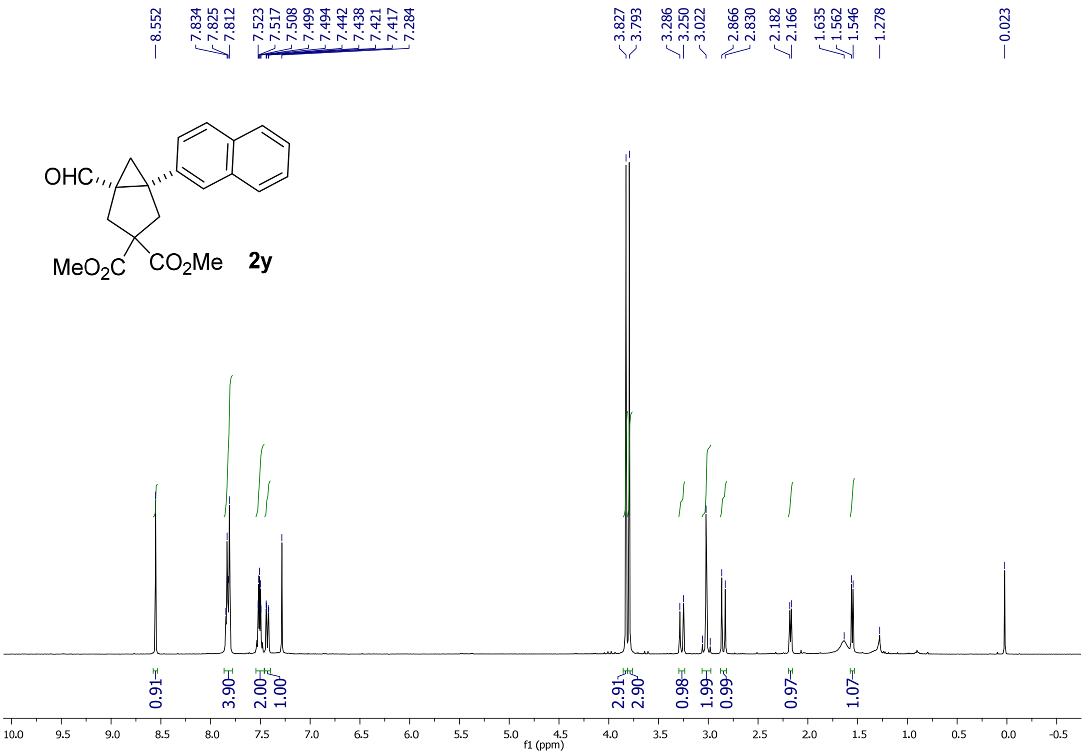

Supplementary Figure 54. ^{1}H NMR of **2y**

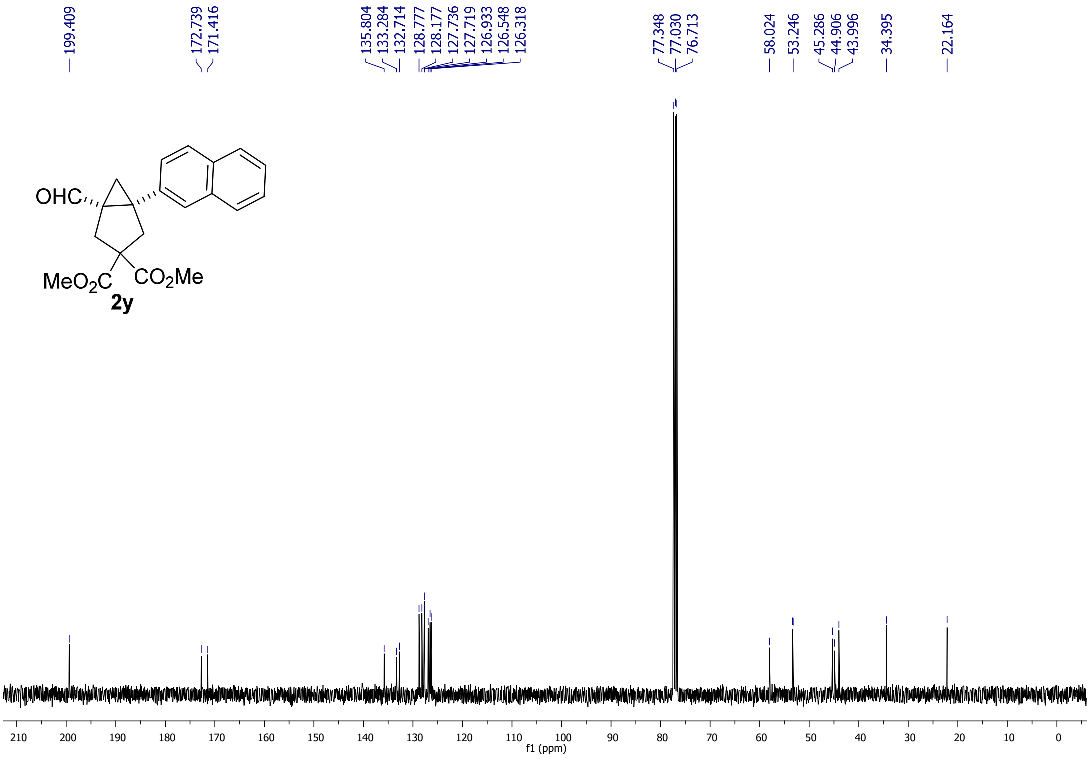

Supplementary Figure 55. ^{13}C NMR of **2y**

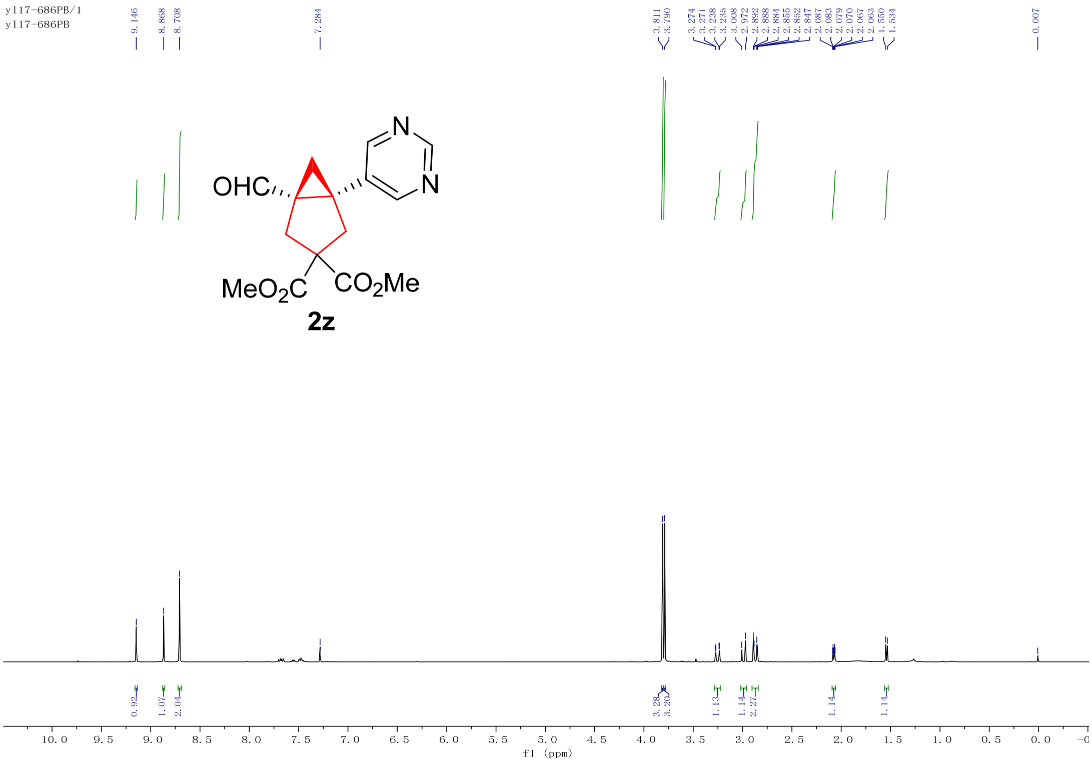

Supplementary Figure 56. ^{1}H NMR of **2z**

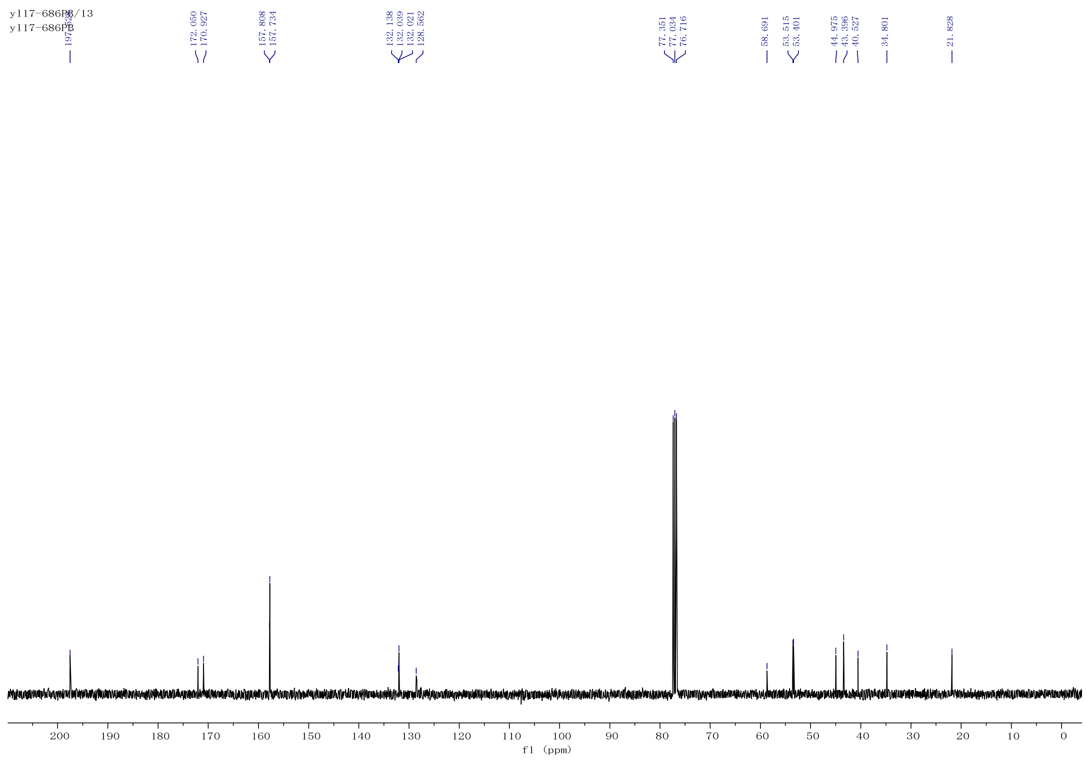

Supplementary Figure 57. ^{13}C NMR of **2z**

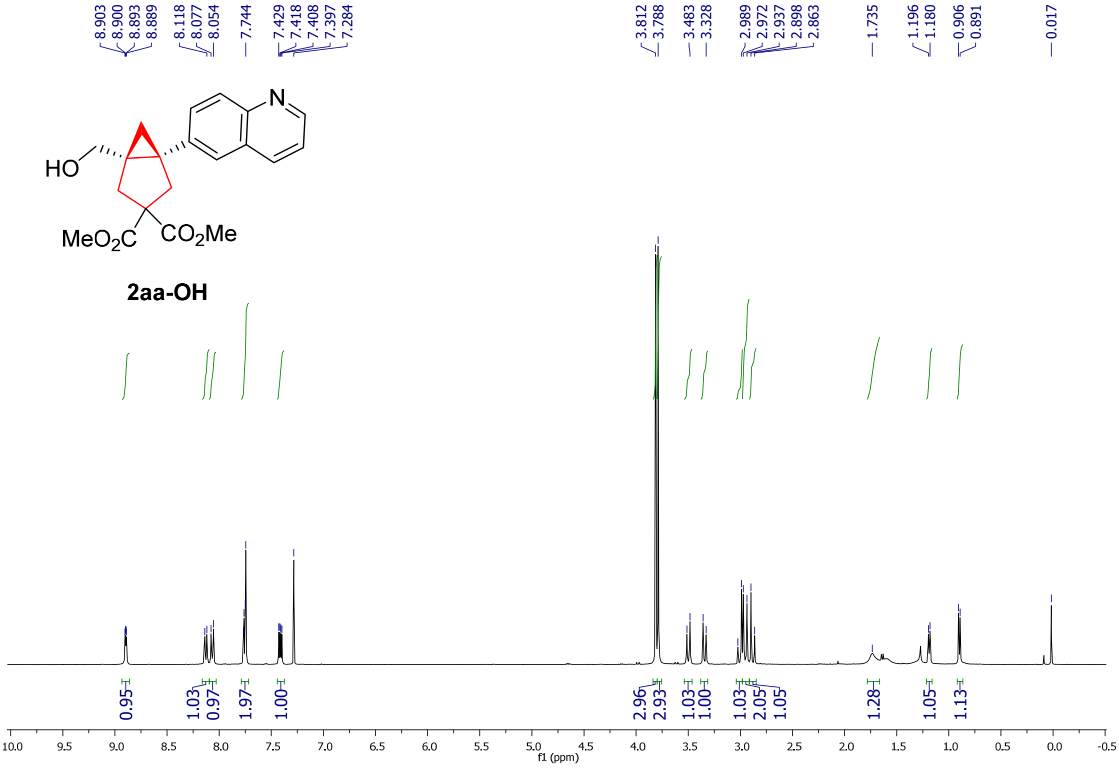

Supplementary Figure 58. ^{1}H NMR of **2aa-OH**

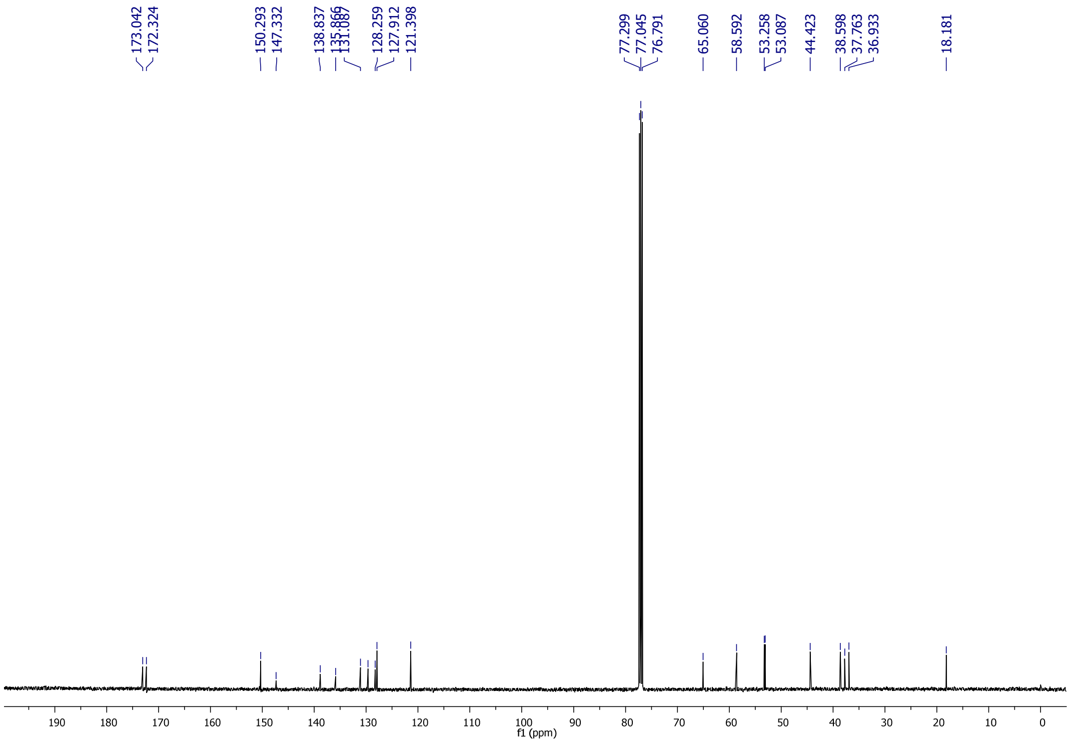

Supplementary Figure 59. ^{13}C NMR of **2aa-OH**

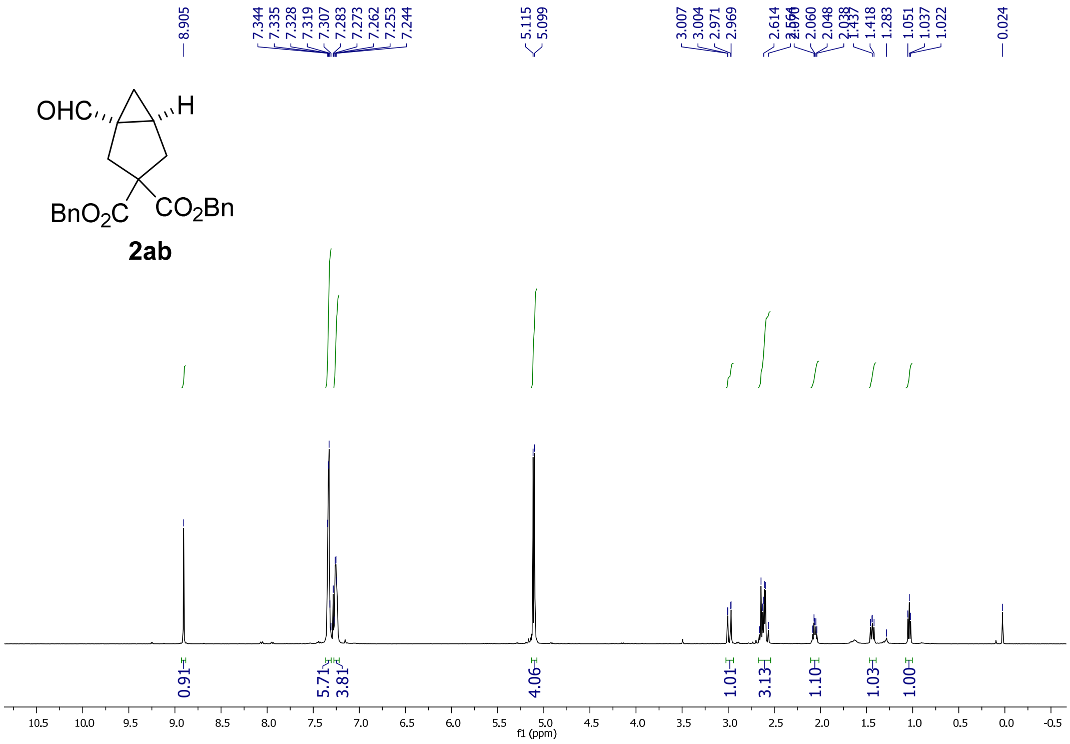

Supplementary Figure 60. ^{1}H NMR of **2ab**

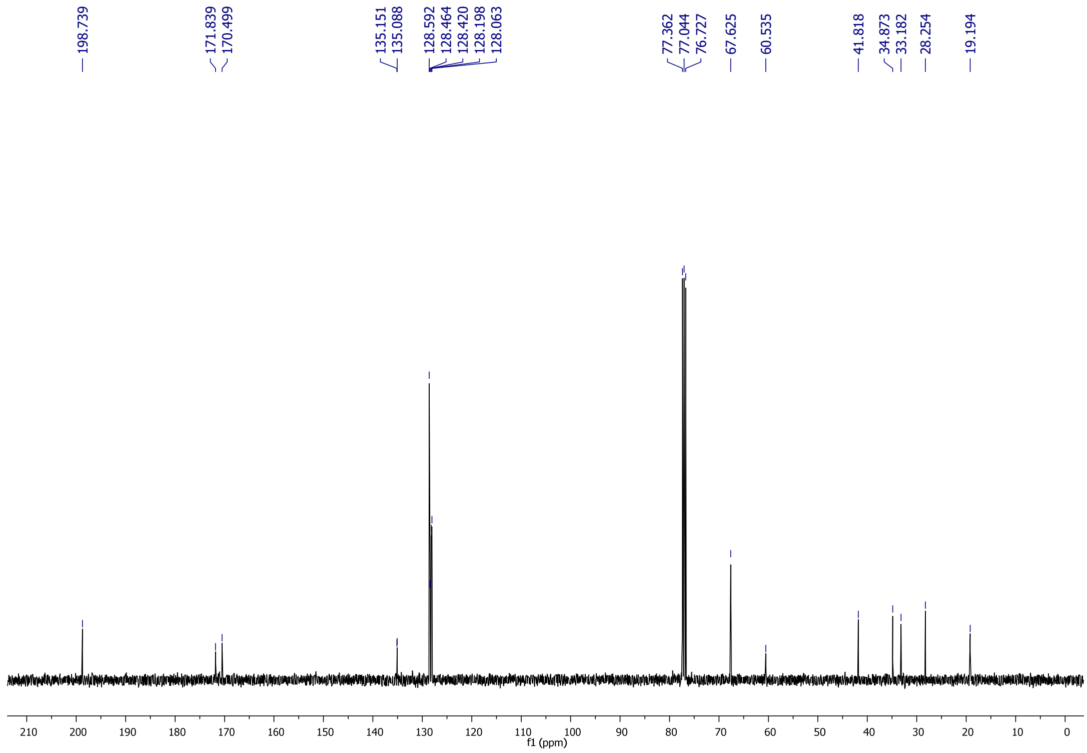

Supplementary Figure 61. ^{13}C NMR of **2ab**

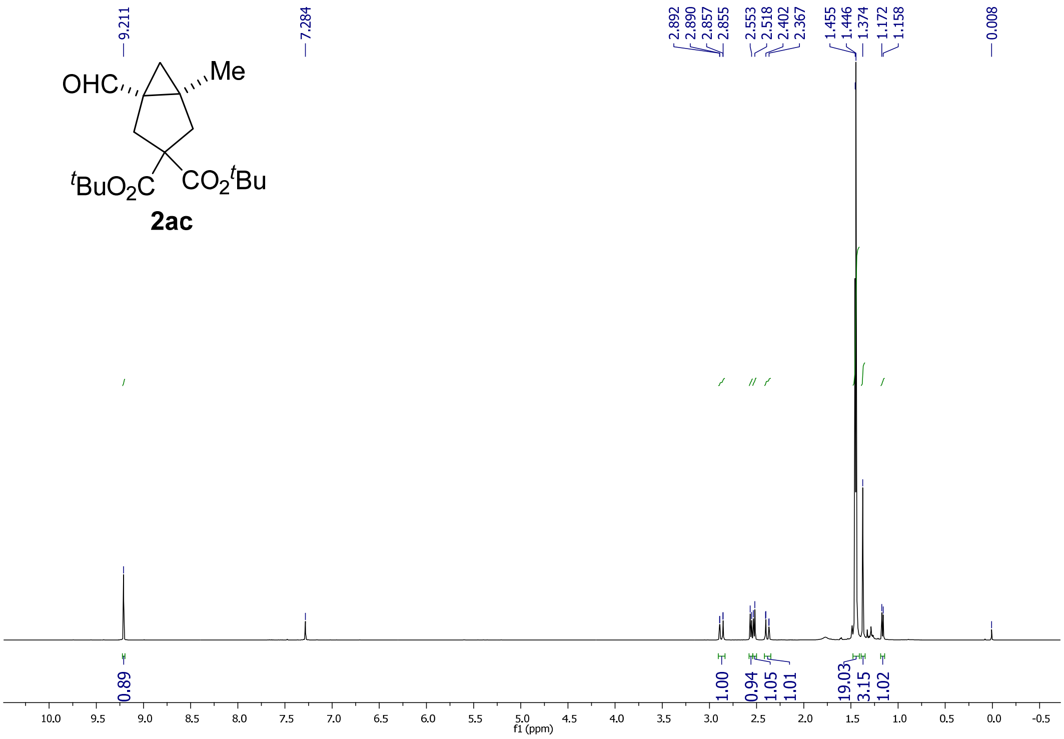

Supplementary Figure 62. ^{1}H NMR of **2ac**

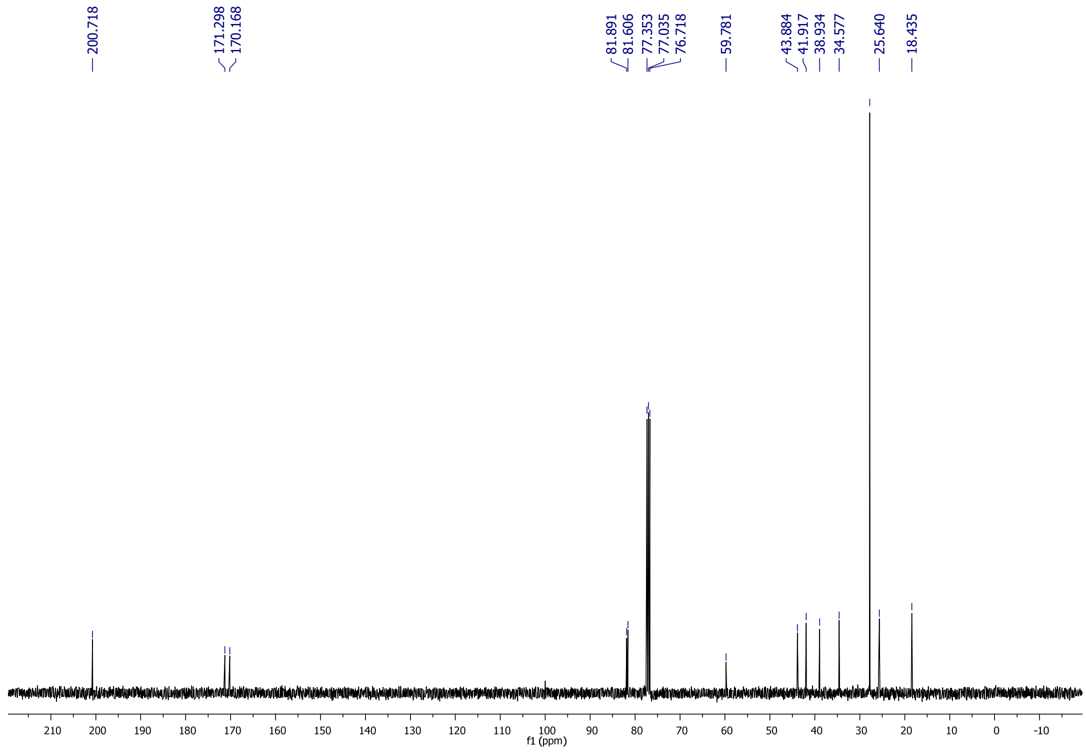

Supplementary Figure 63. ^{13}C NMR of **2ac**

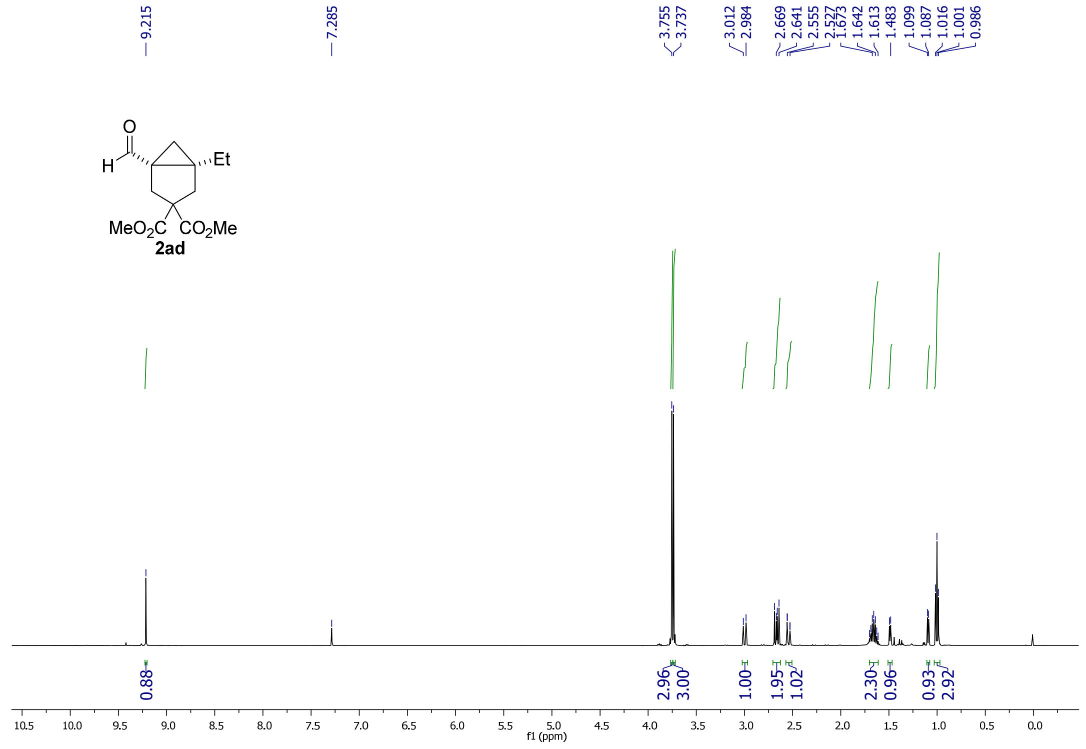

Supplementary Figure 64. ^{1}H NMR of **2ad**

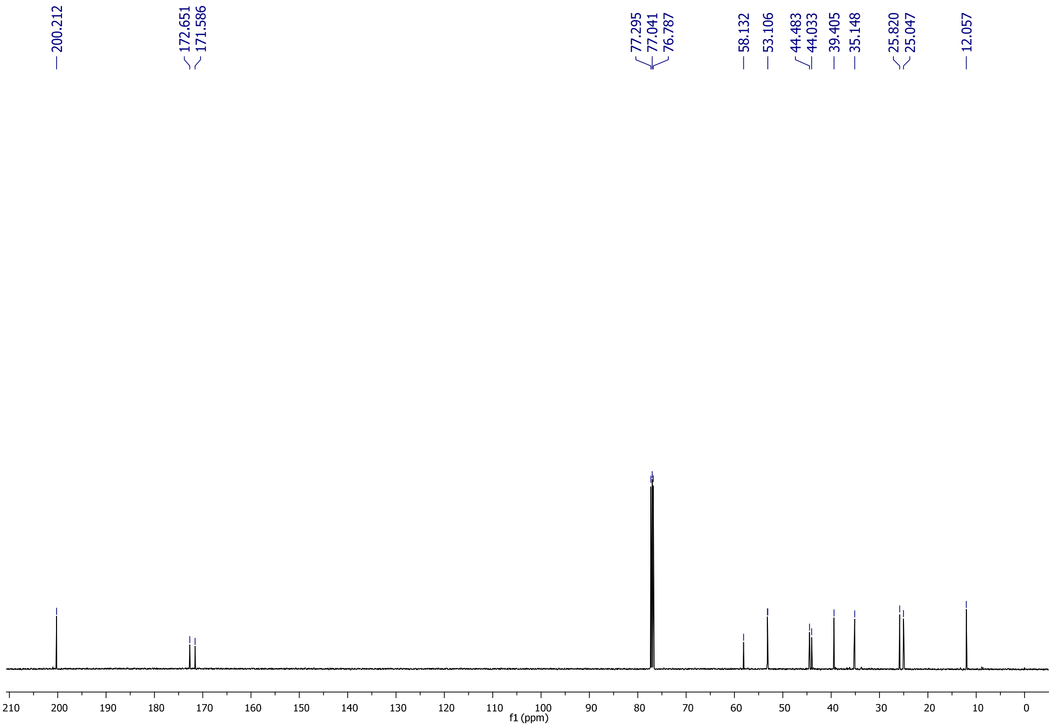

Supplementary Figure 65. ^{13}C NMR of **2ad**

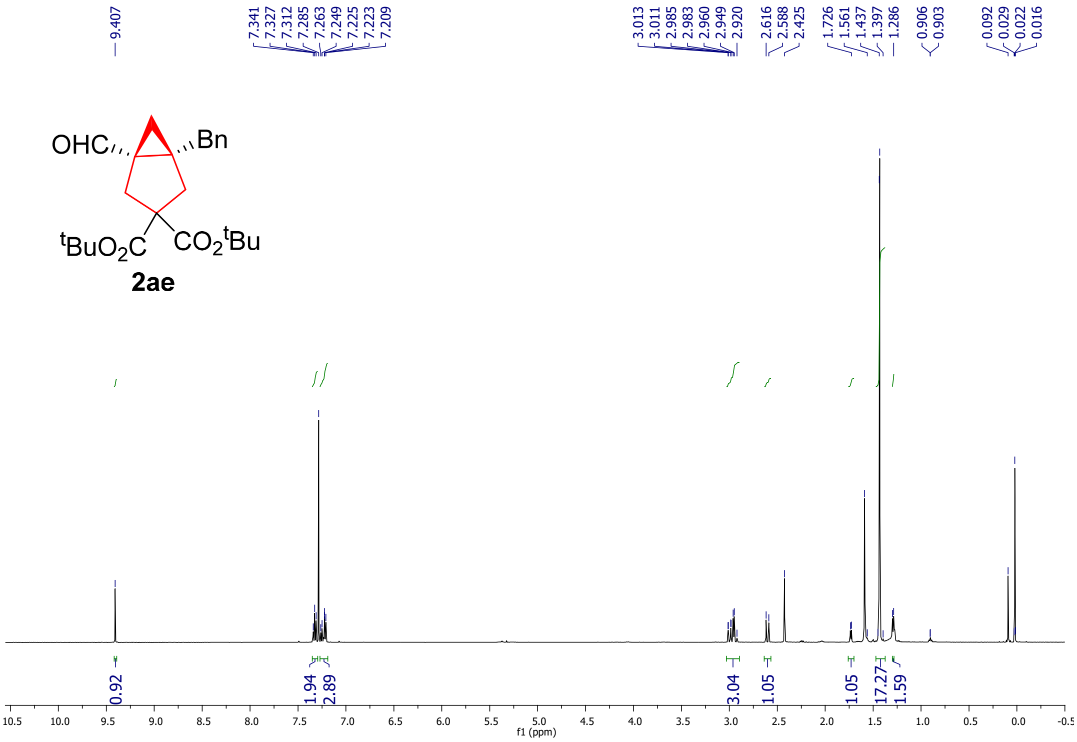

Supplementary Figure 66. ^{1}H NMR of **2ae**

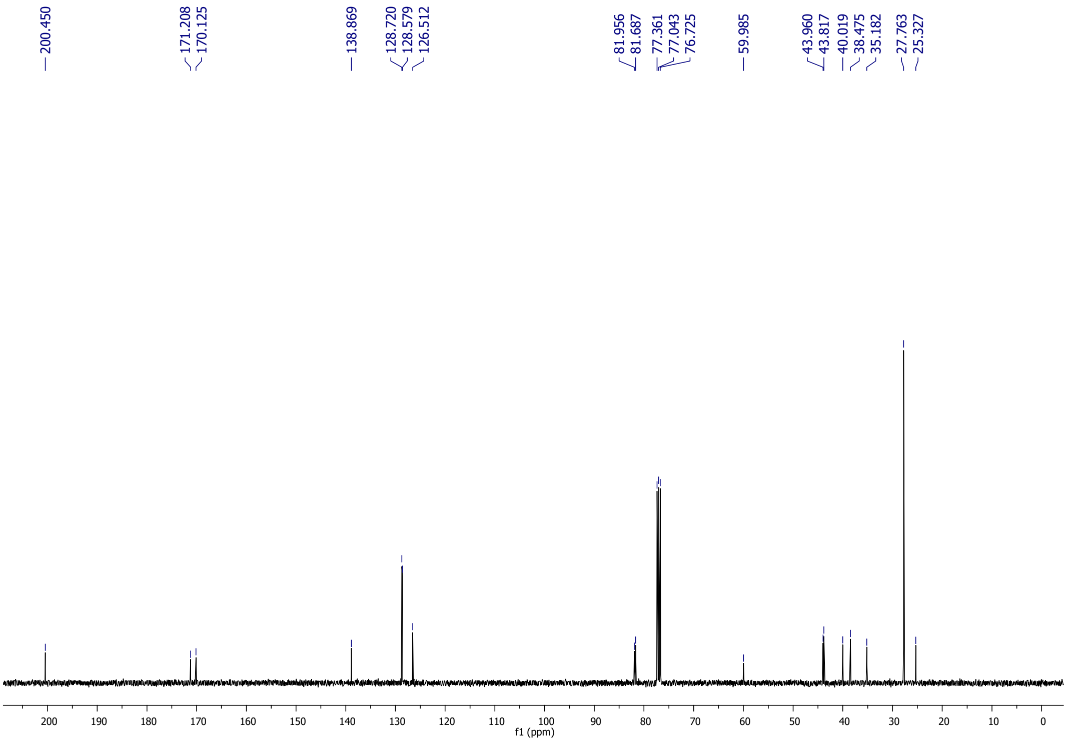

Supplementary Figure 67. ^{13}C NMR of **2ae**

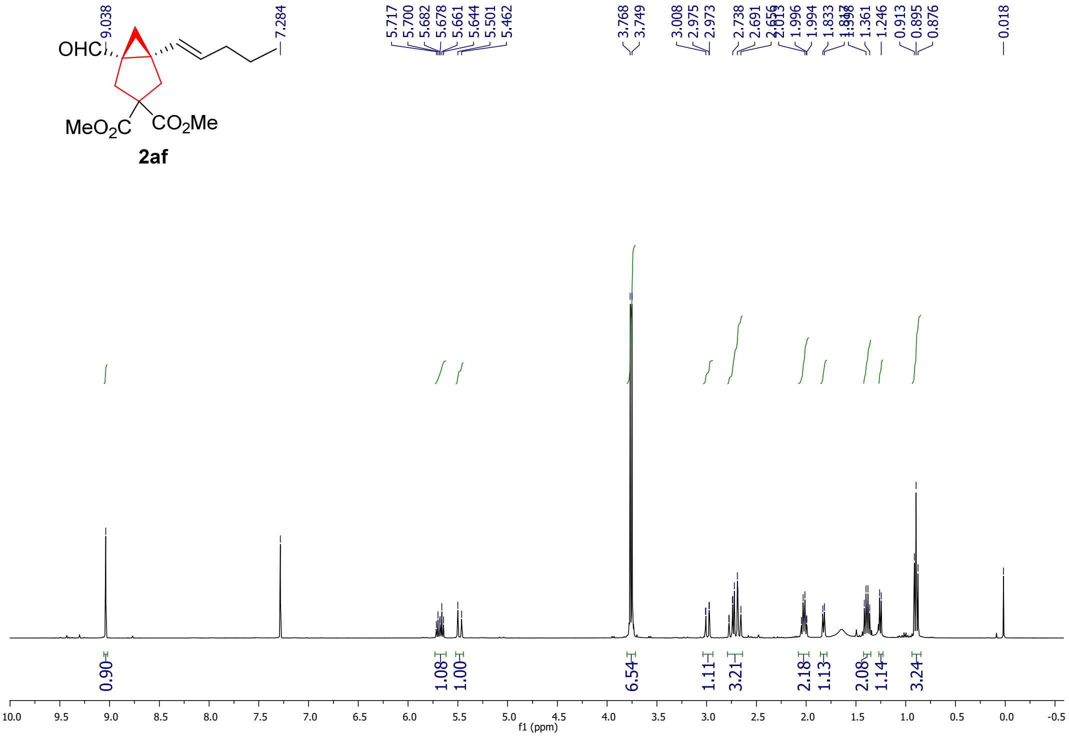

Supplementary Figure 68. ^{1}H NMR of **2af**

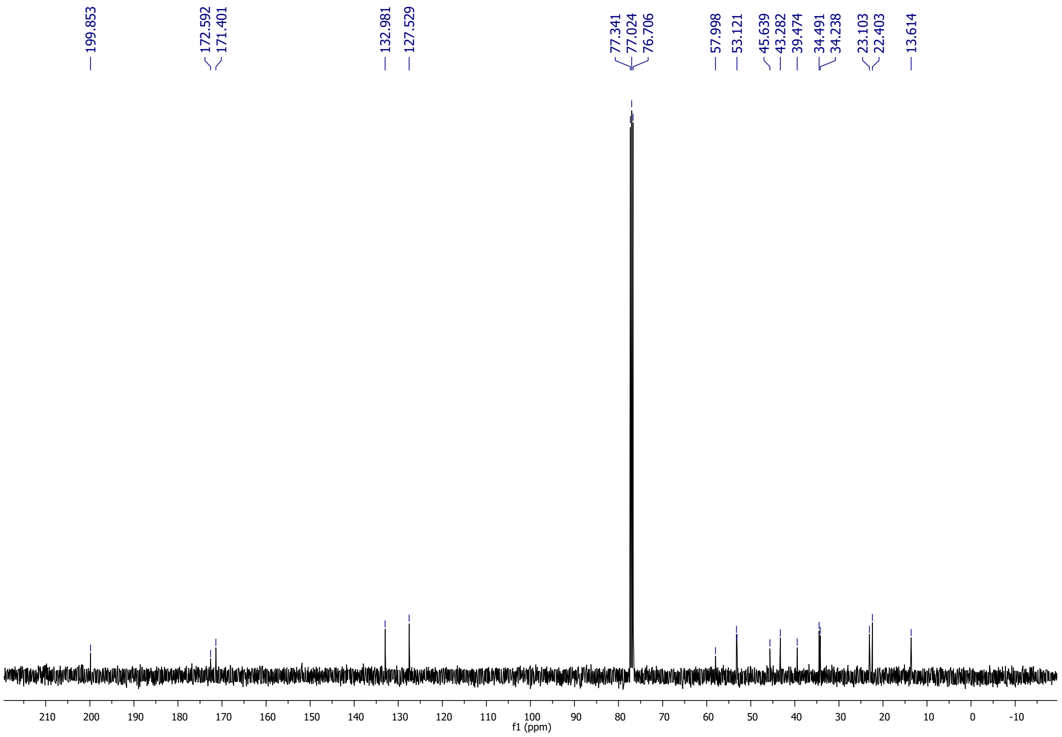

Supplementary Figure 69. ^{13}C NMR of **2af**

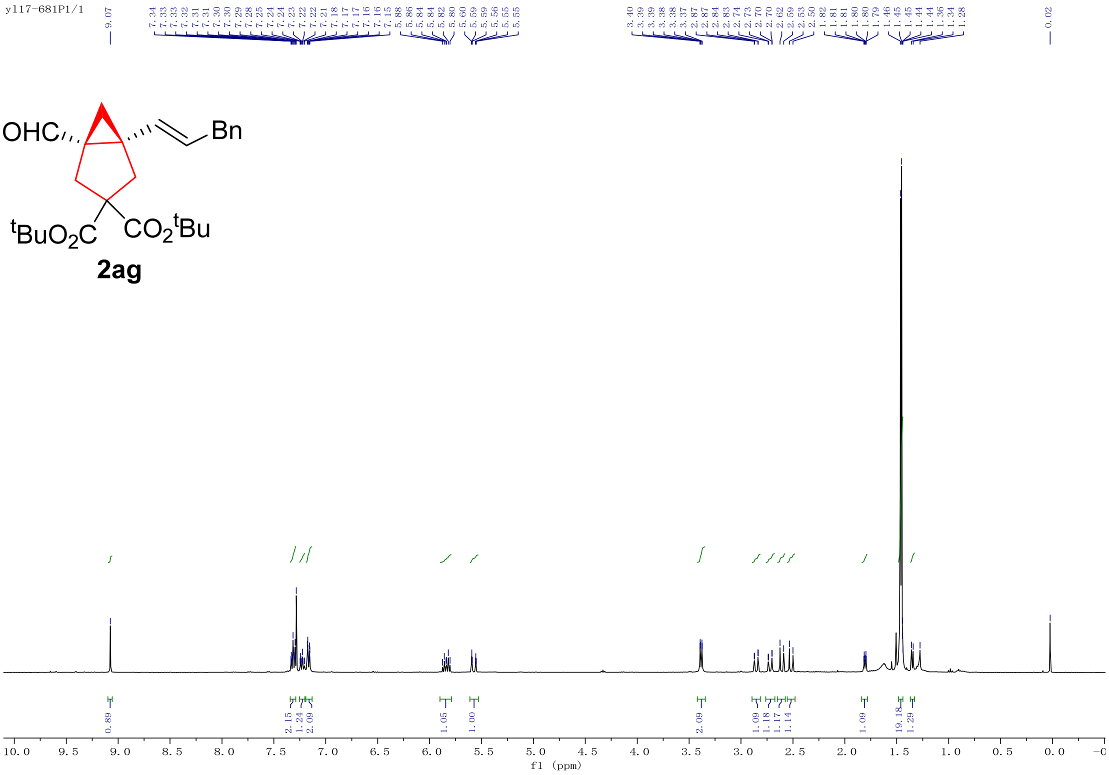

Supplementary Figure 70. ^{1}H NMR of **2ag**

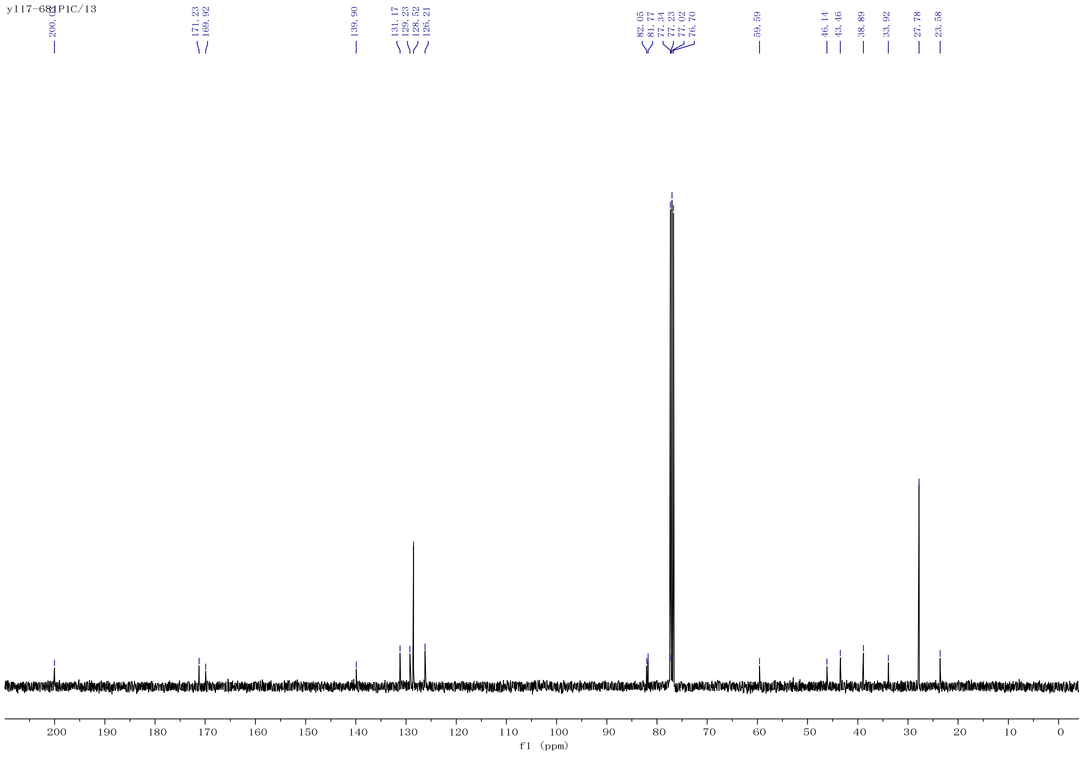

Supplementary Figure 71. ^{13}C NMR of **2ag**

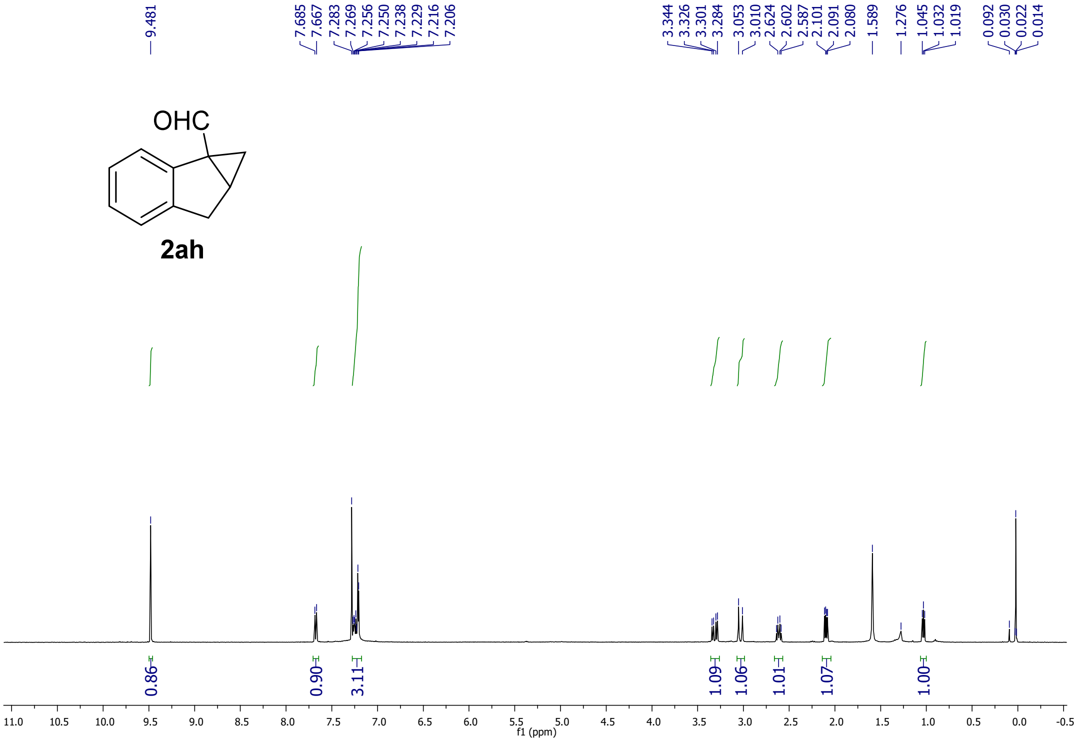

Supplementary Figure 72. ^{1}H NMR of **2ah**

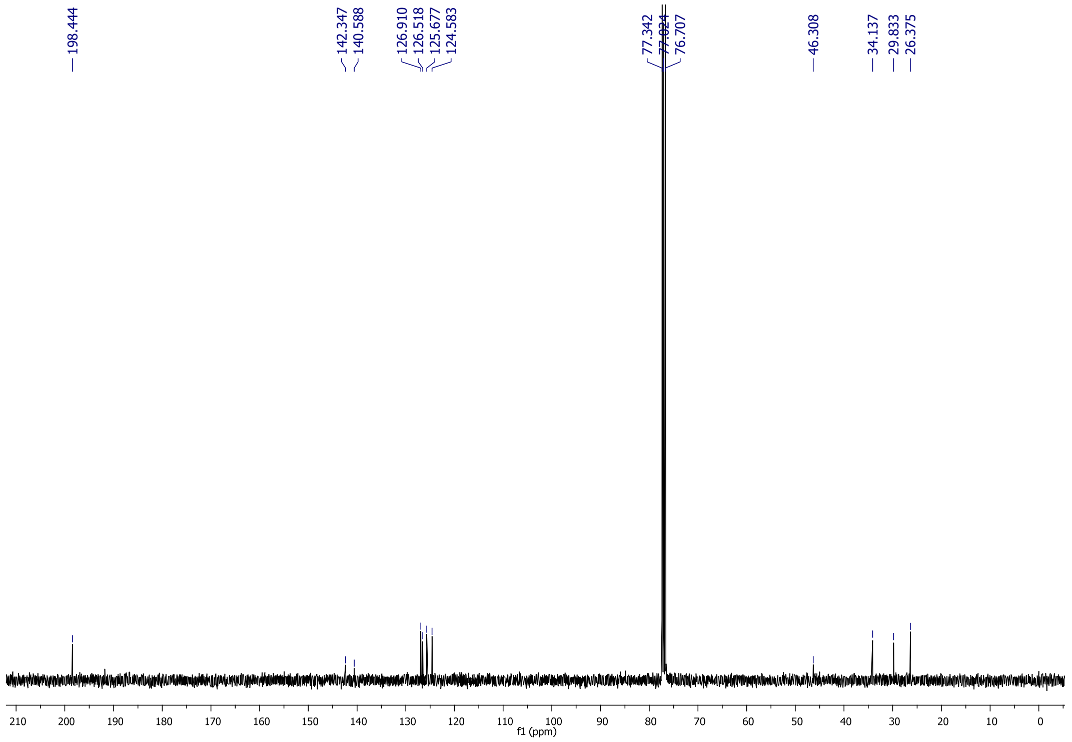

Supplementary Figure 73. ^{13}C NMR of **2ah**

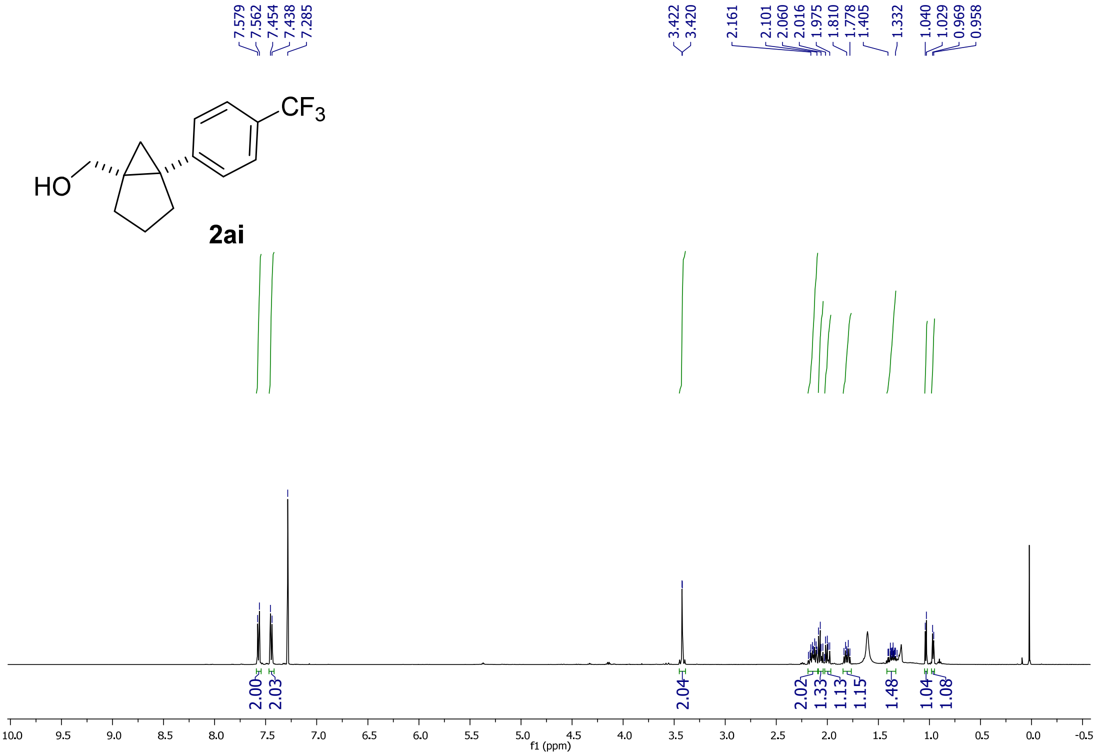

Supplementary Figure 74. ^{1}H NMR of **2ai**

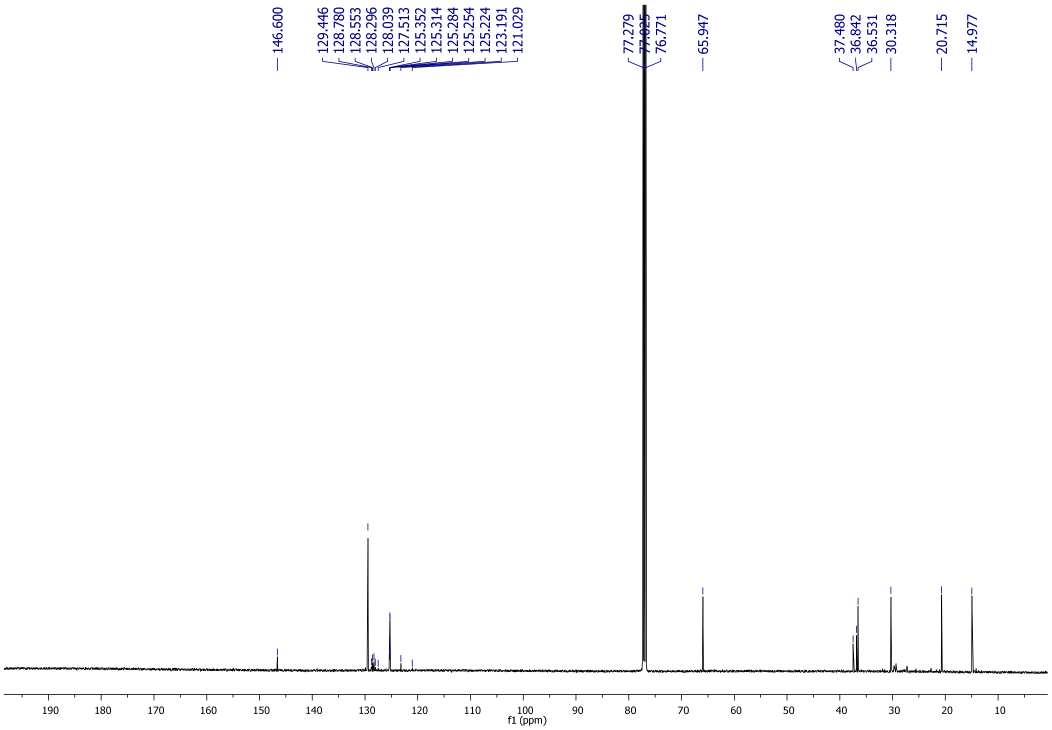

Supplementary Figure 75. ^{13}C NMR of **2ai**

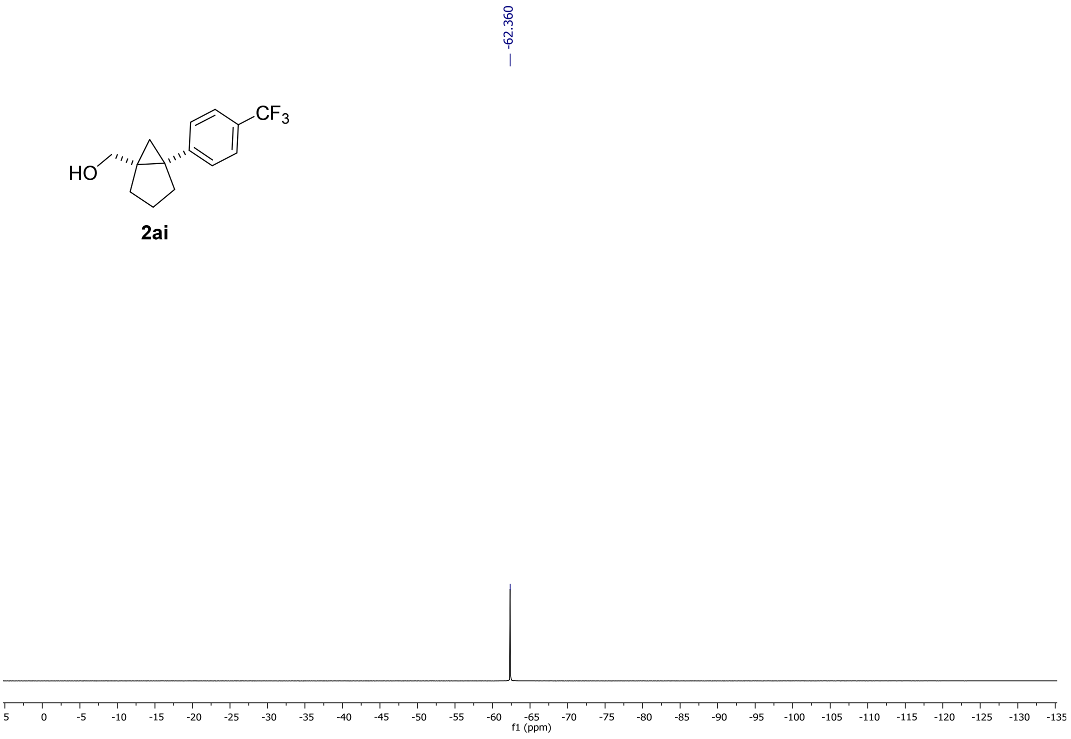

Supplementary Figure 76. ^{19}F NMR of **2ai**

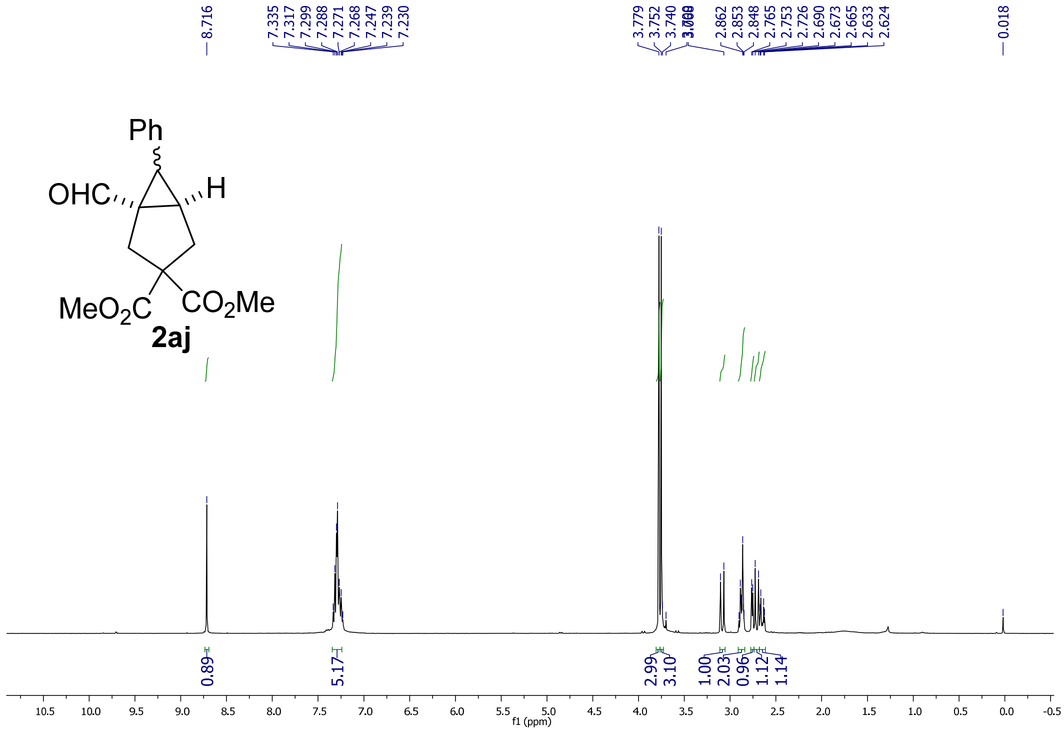

Supplementary Figure 74. ^{1}H NMR of **2aj**

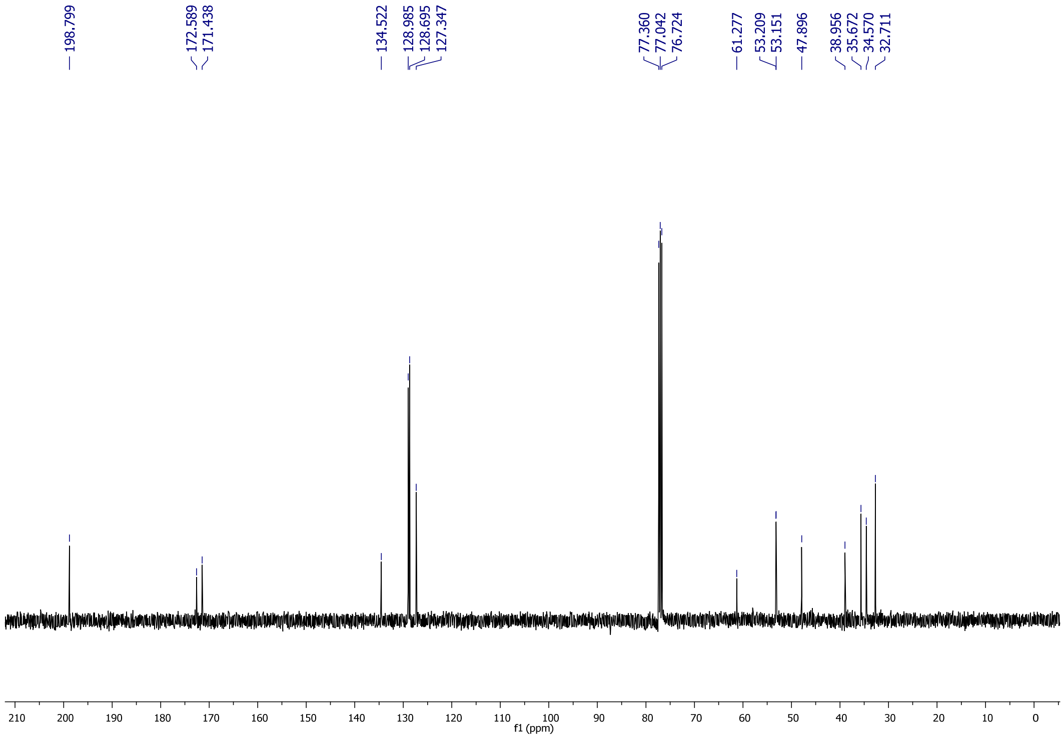

Supplementary Figure 78. ^{13}C NMR of **2aj**

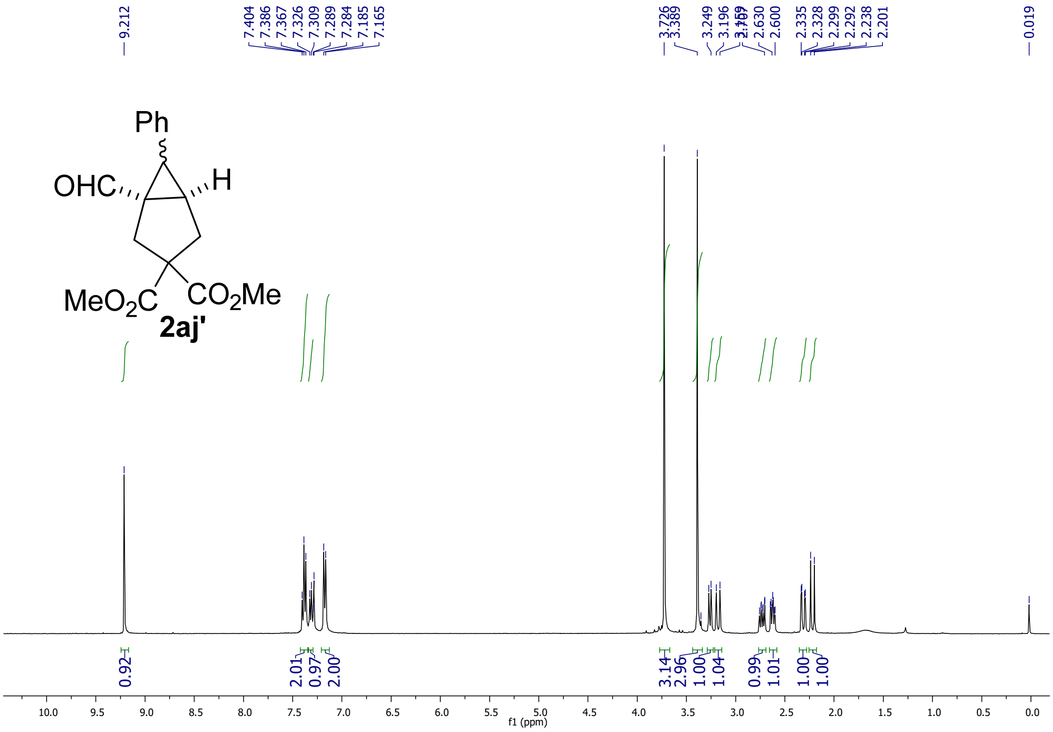

Supplementary Figure 79. ^{1}H NMR of **2aj’**

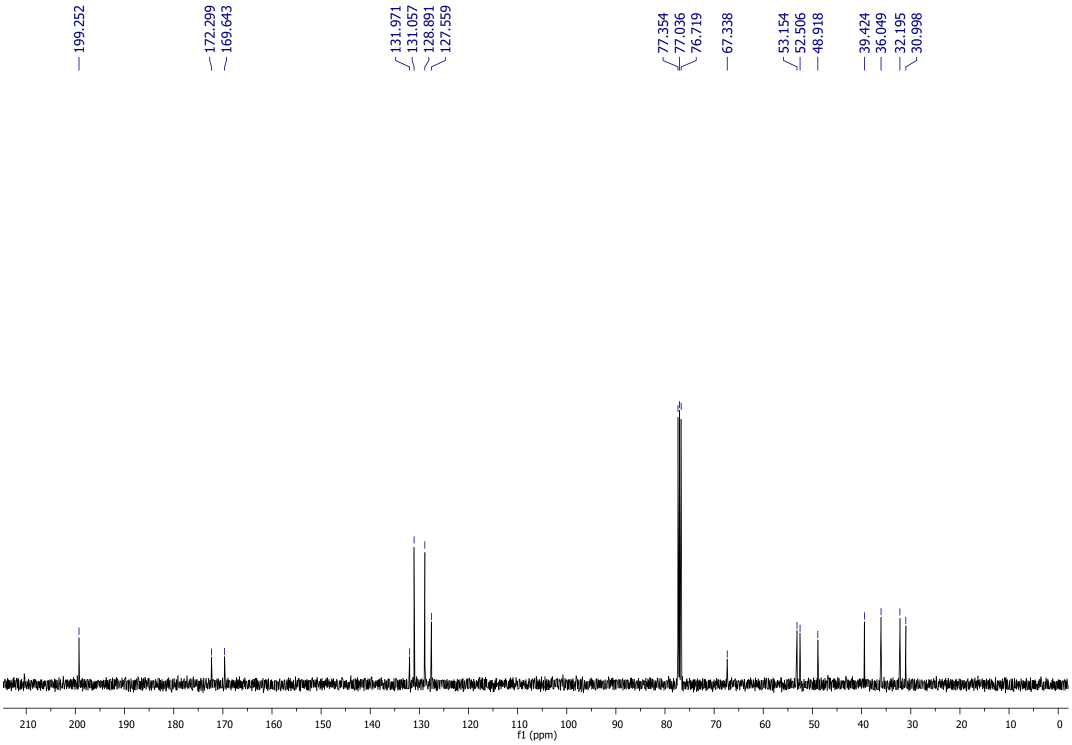

Supplementary Figure 80. ^{13}C NMR of **2aj’**

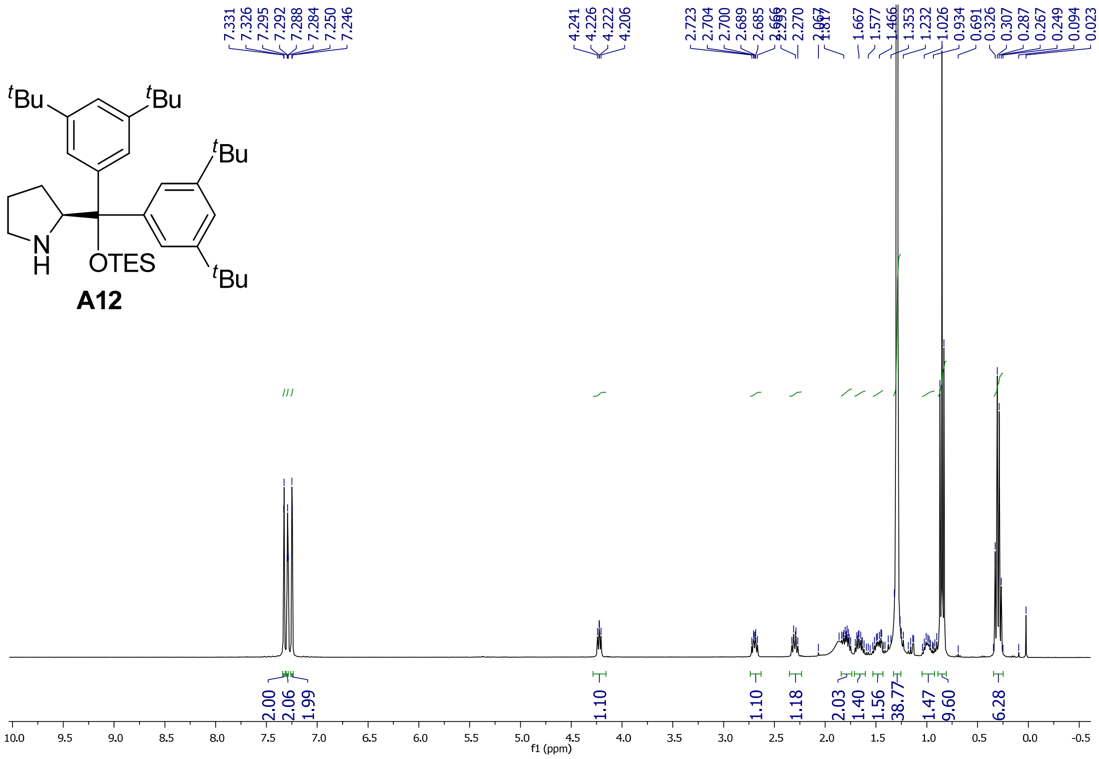

Supplementary Figure 81. ^{1}H NMR of **A12**

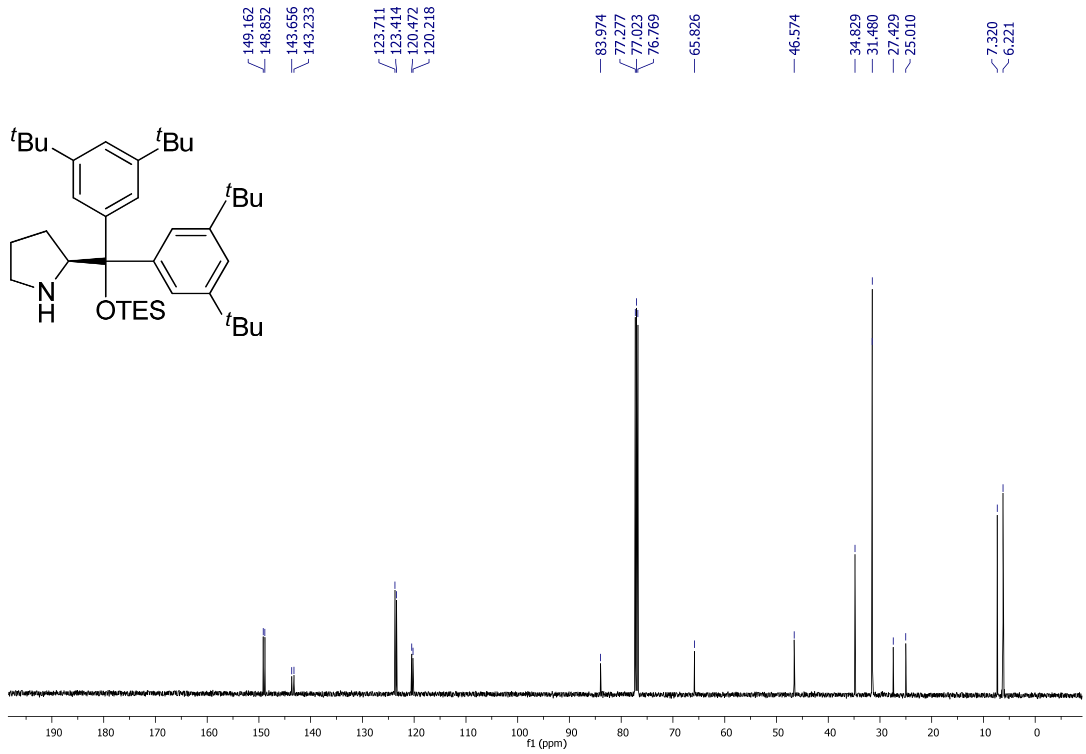

Supplementary Figure 82. ^{13}C NMR of **A12**

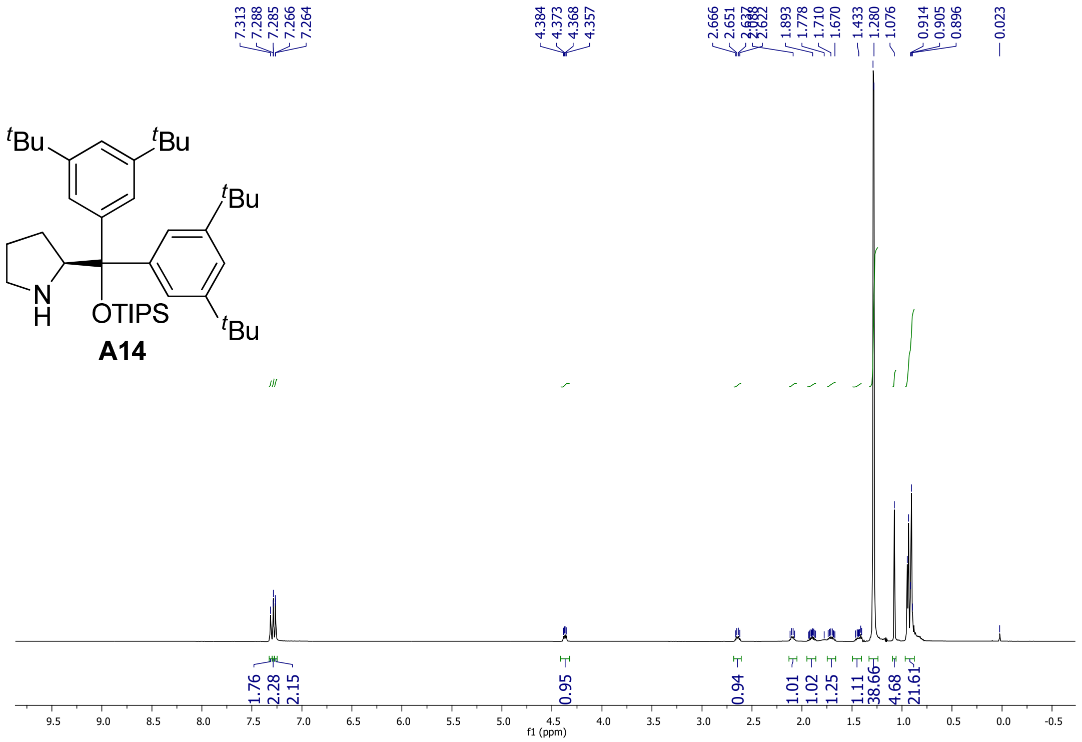

Supplementary Figure 83. ^{1}H NMR of **A14**

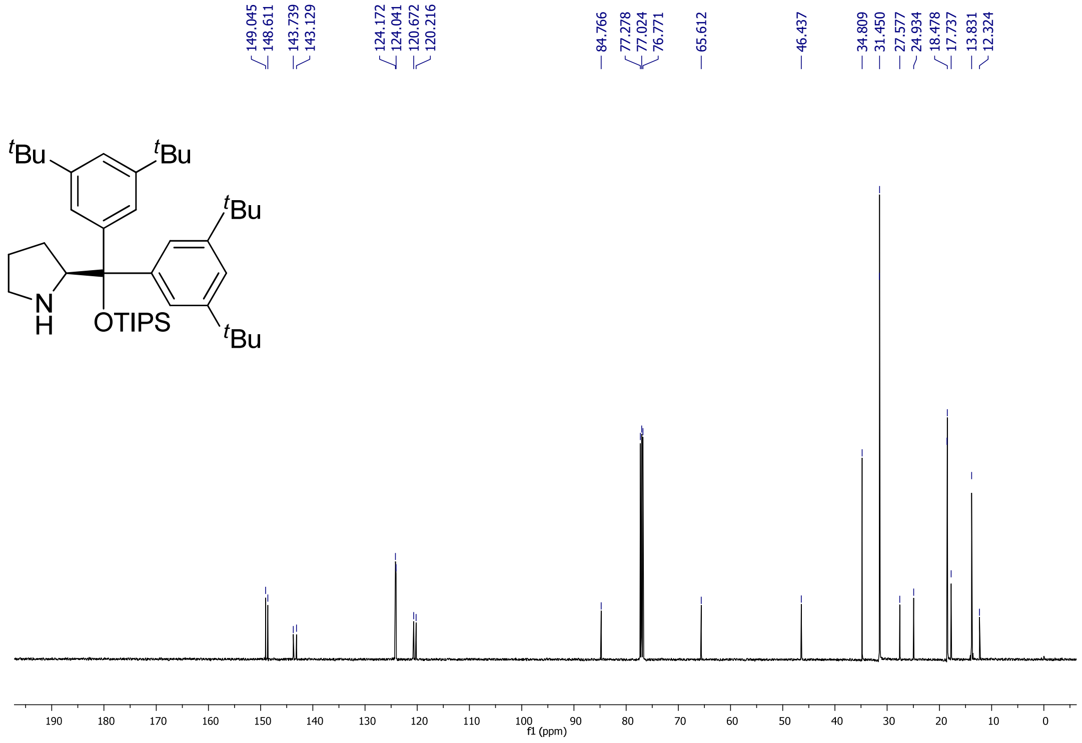

Supplementary Figure 84. ^{13}C NMR of **A14**

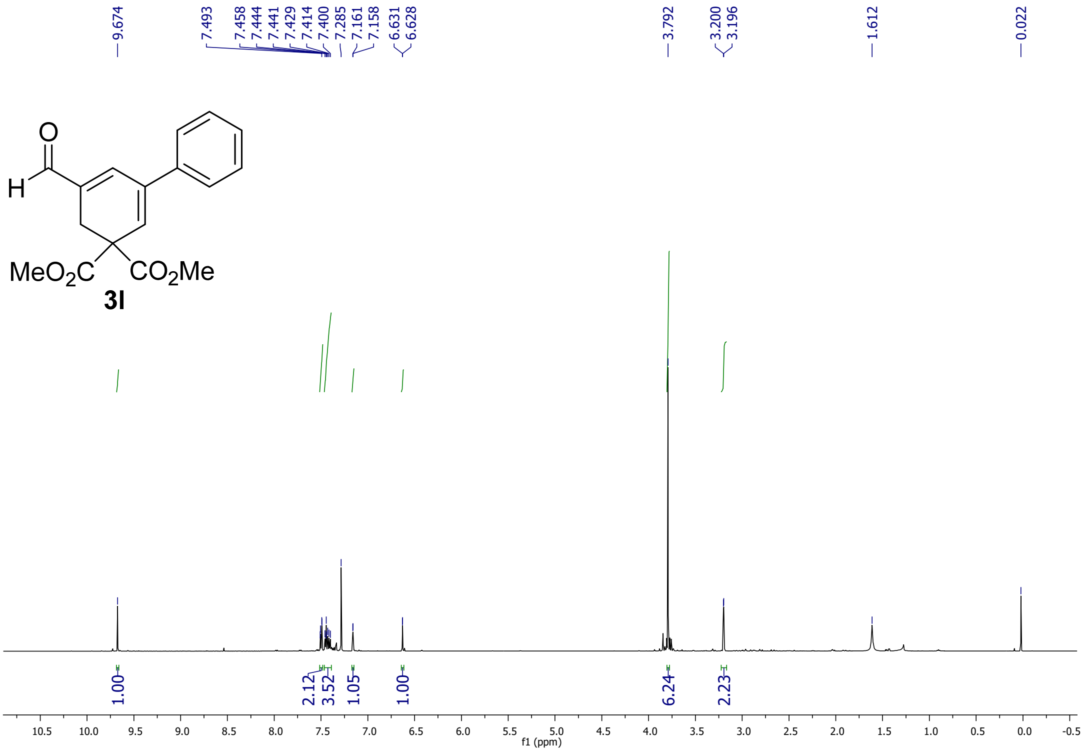

Supplementary Figure 85. ^{1}H NMR of **3l**

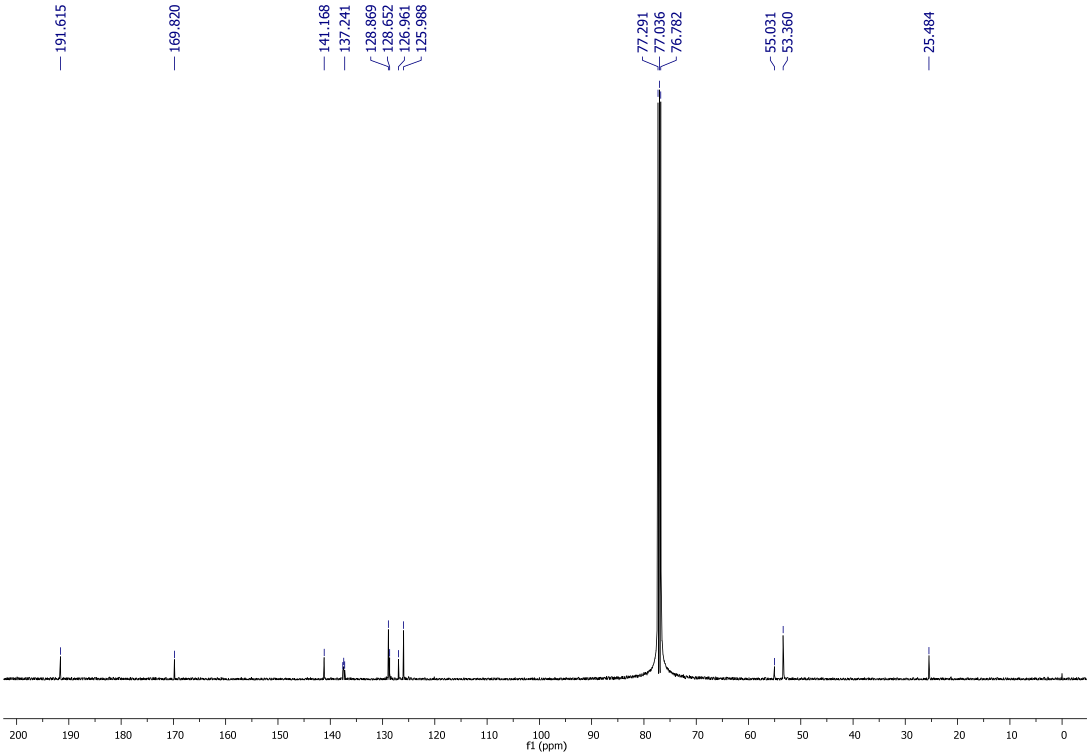

Supplementary Figure 86. ^{13}C NMR of **3l**

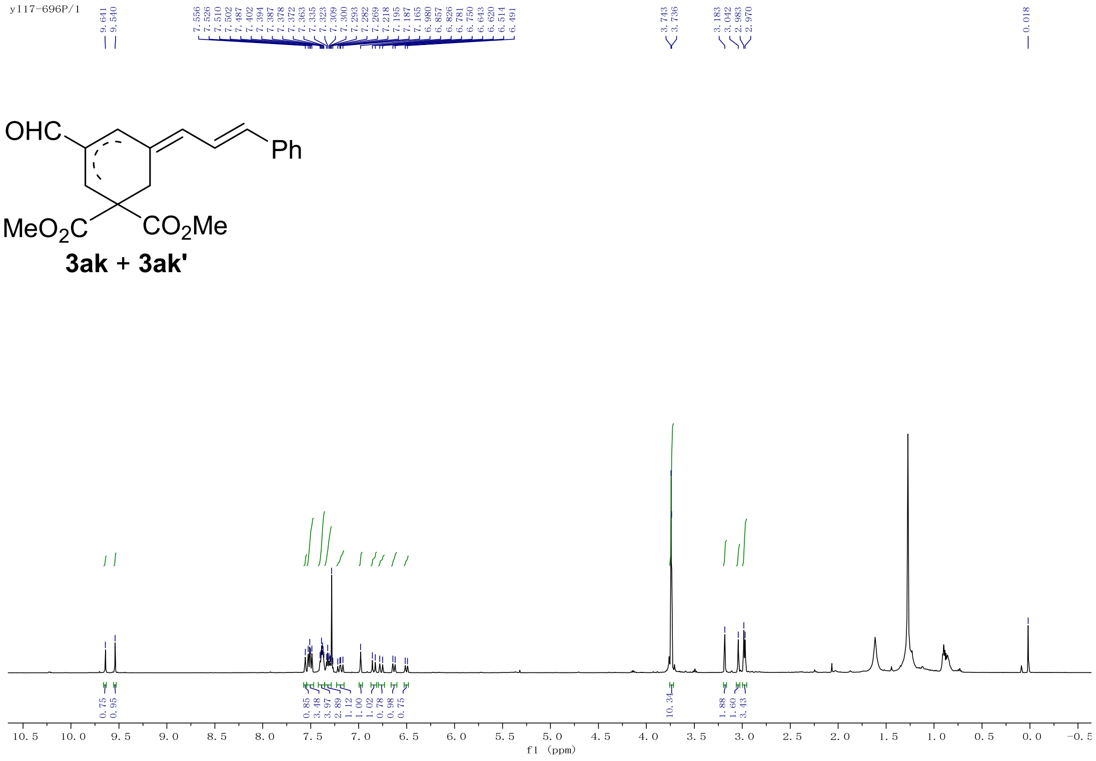

Supplementary Figure 87. ^{1}H NMR of **3ak+3ak’**

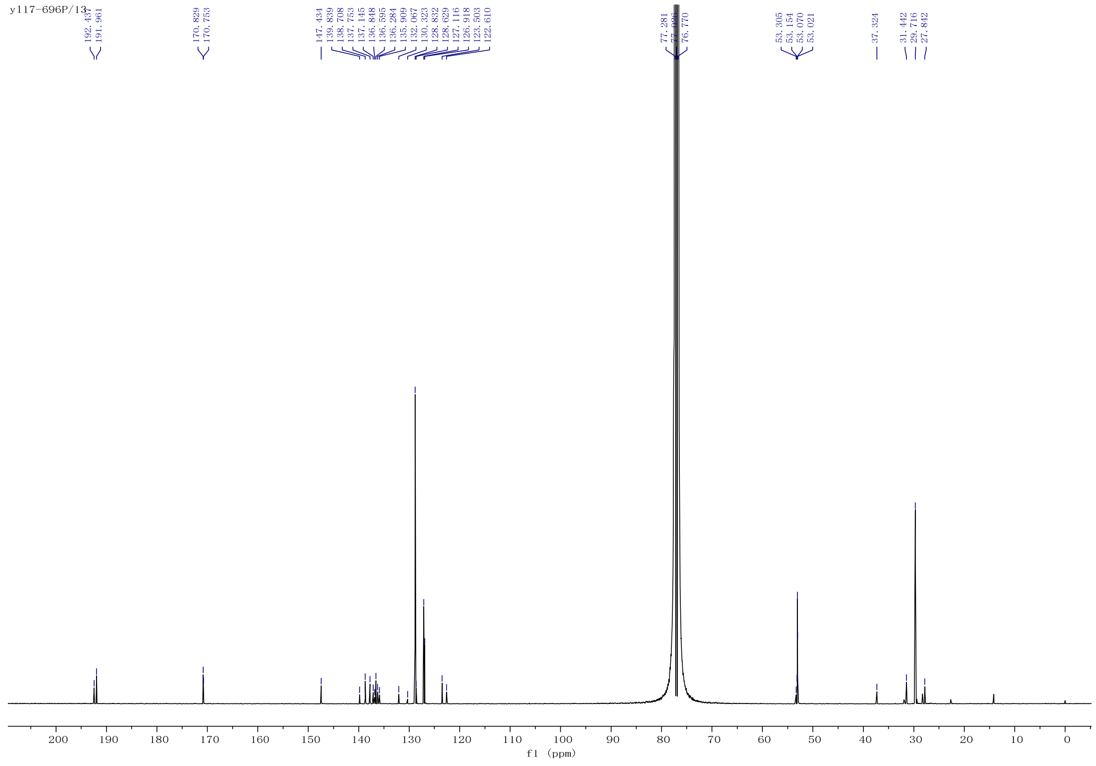

Supplementary Figure 88. ^{13}C NMR of **3ak+3ak’**

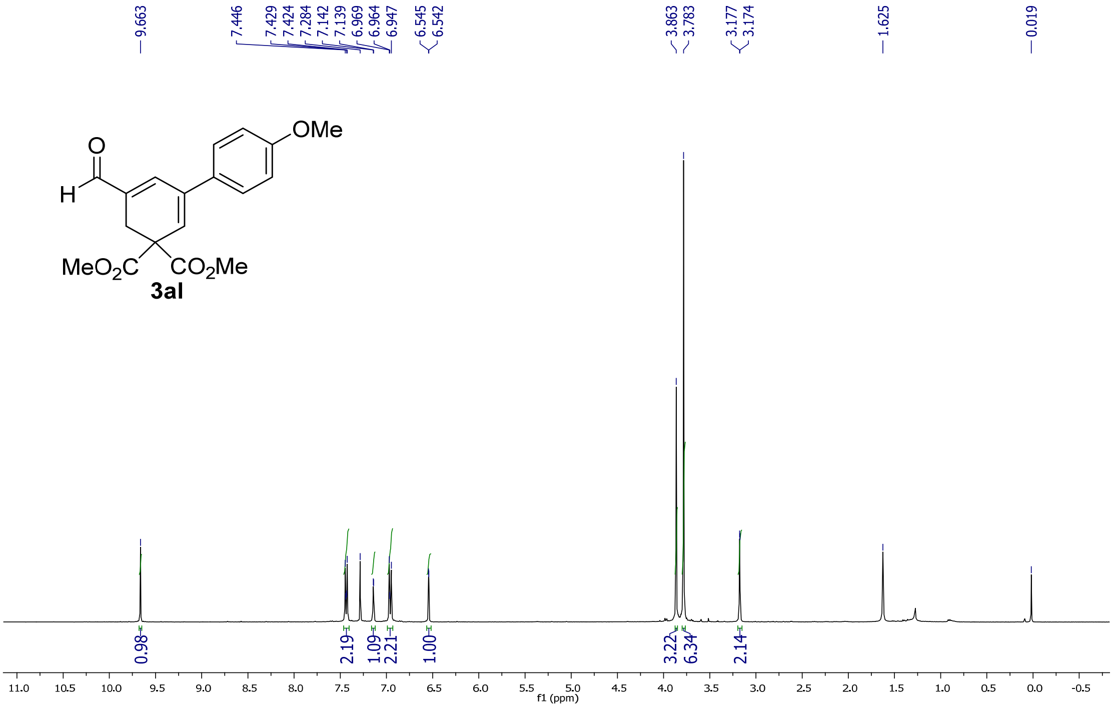

Supplementary Figure 89. ^{1}H NMR of **3al**

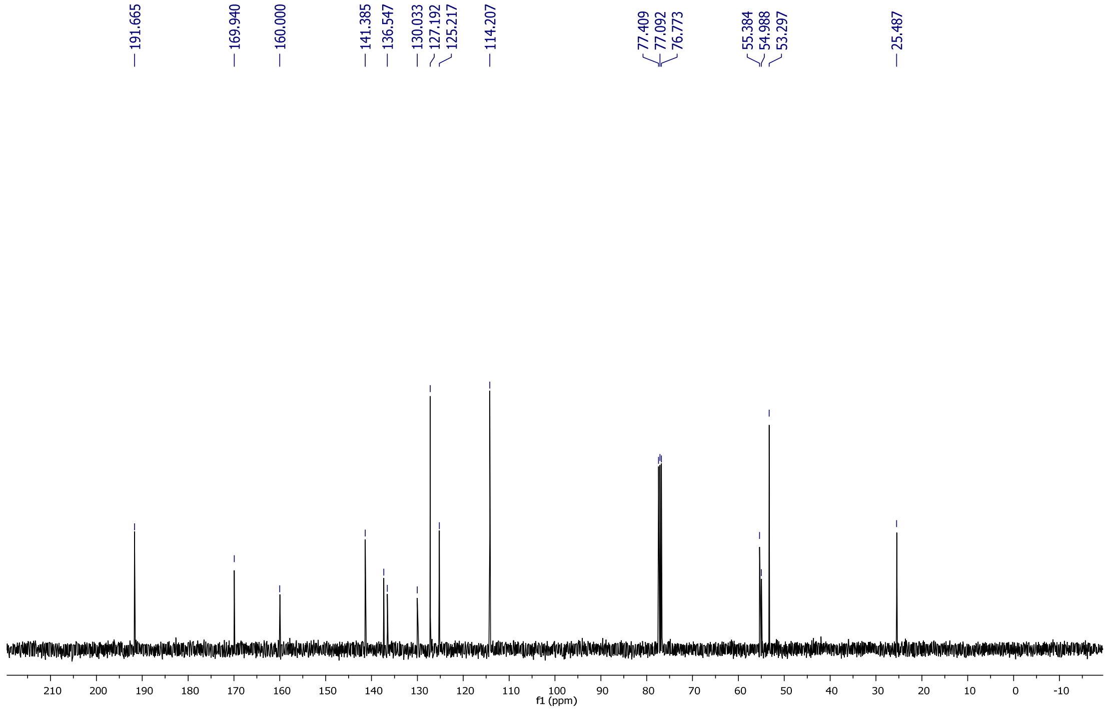

Supplementary Figure 90. ^{13}C NMR of **3al**

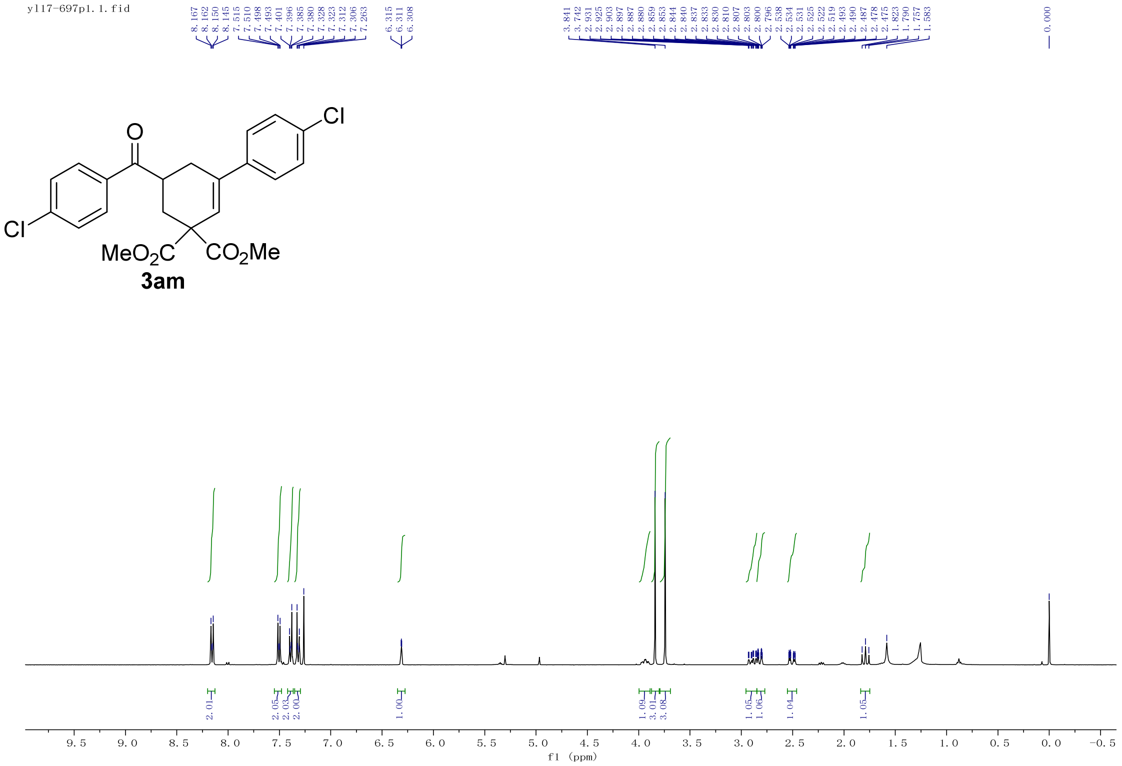

Supplementary Figure 91. ^{1}H NMR of **3am**

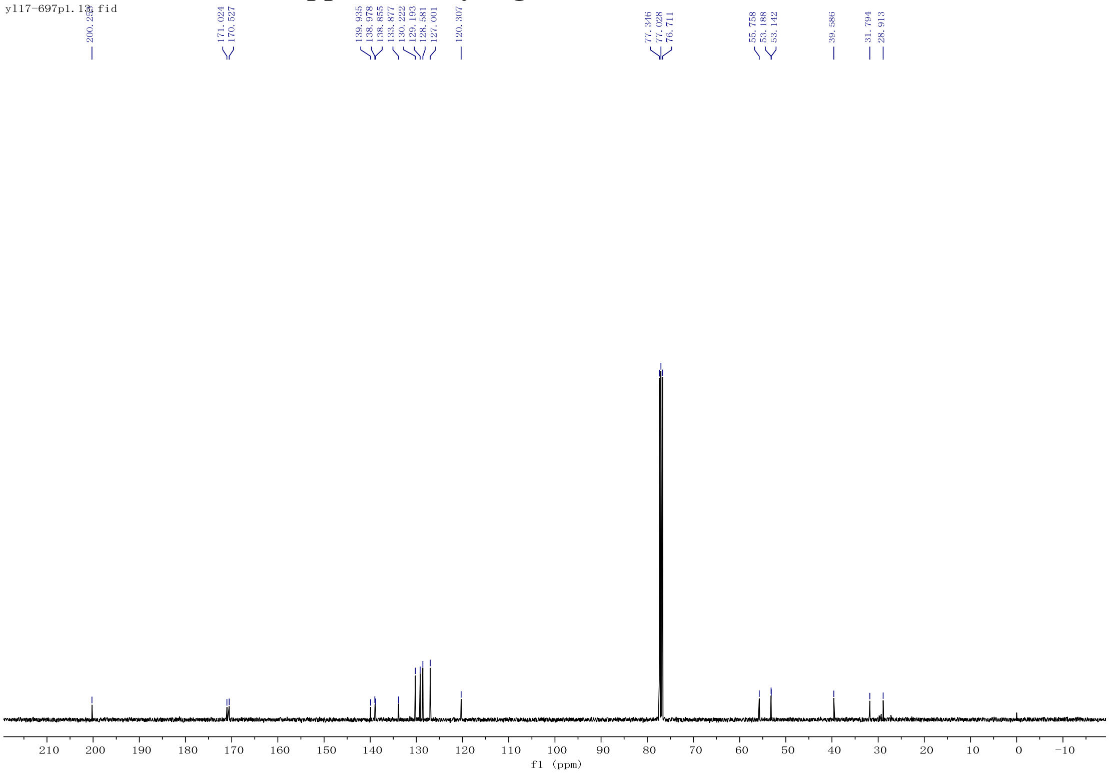

Supplementary Figure 92. ^{13}C NMR of **3am**

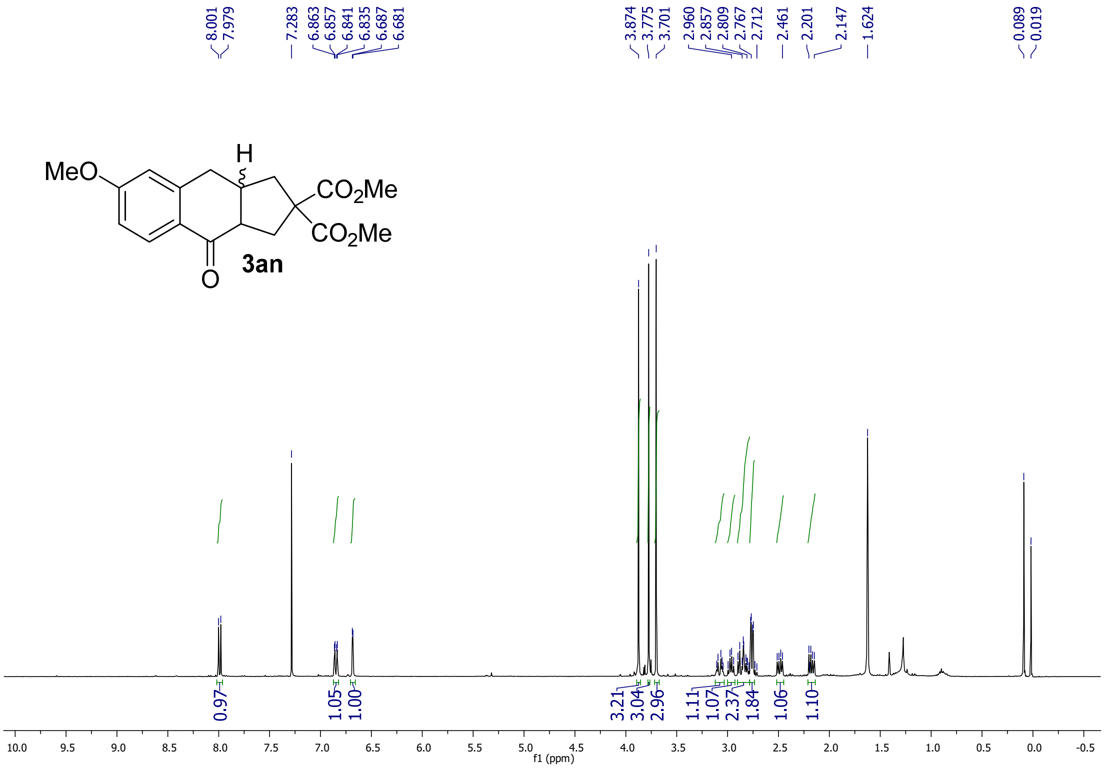

Supplementary Figure 93. ^{1}H NMR of **3an**

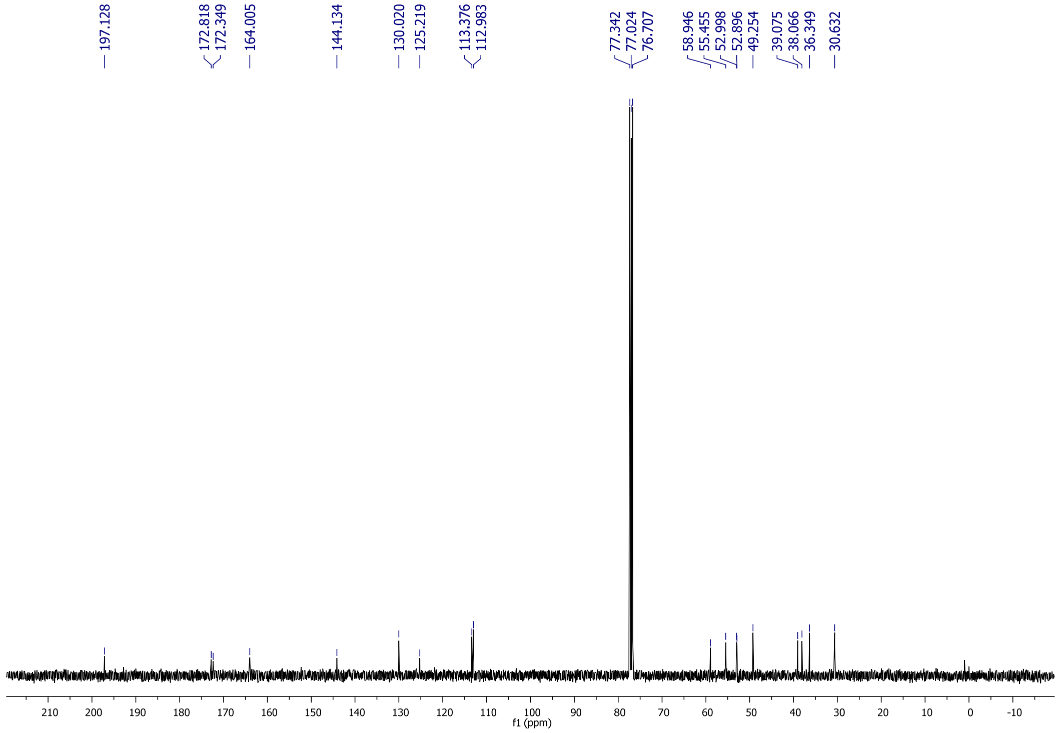

Supplementary Figure 94. ^{13}C NMR of **3an**

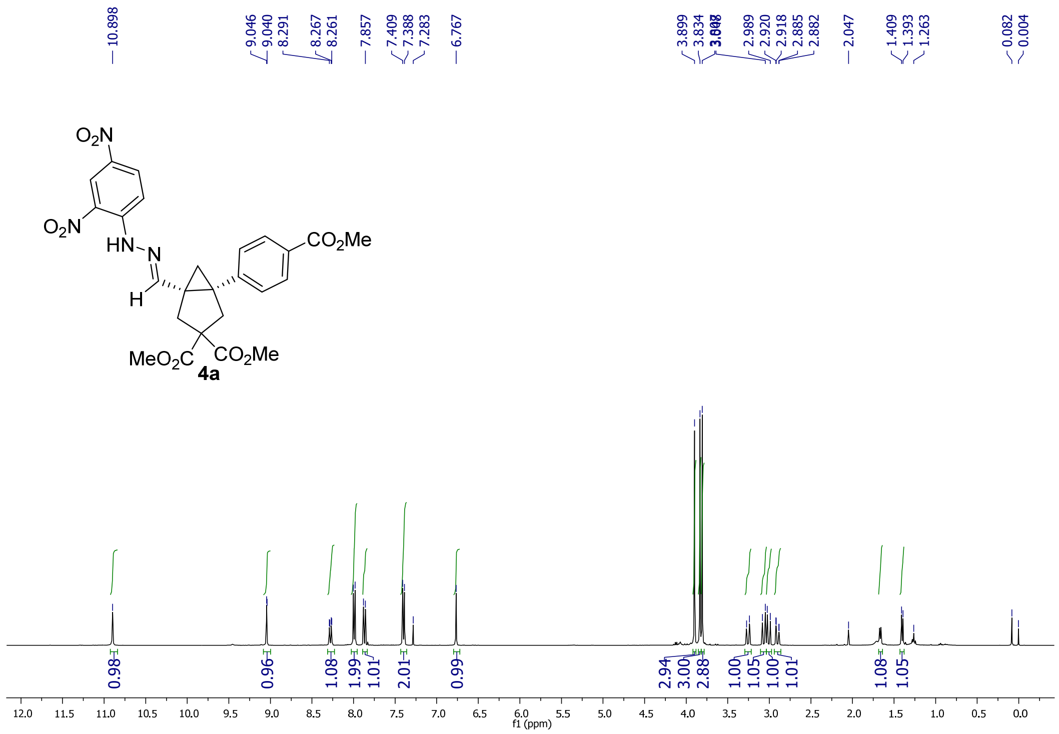

Supplementary Figure 95. ^{1}H NMR of **4a**

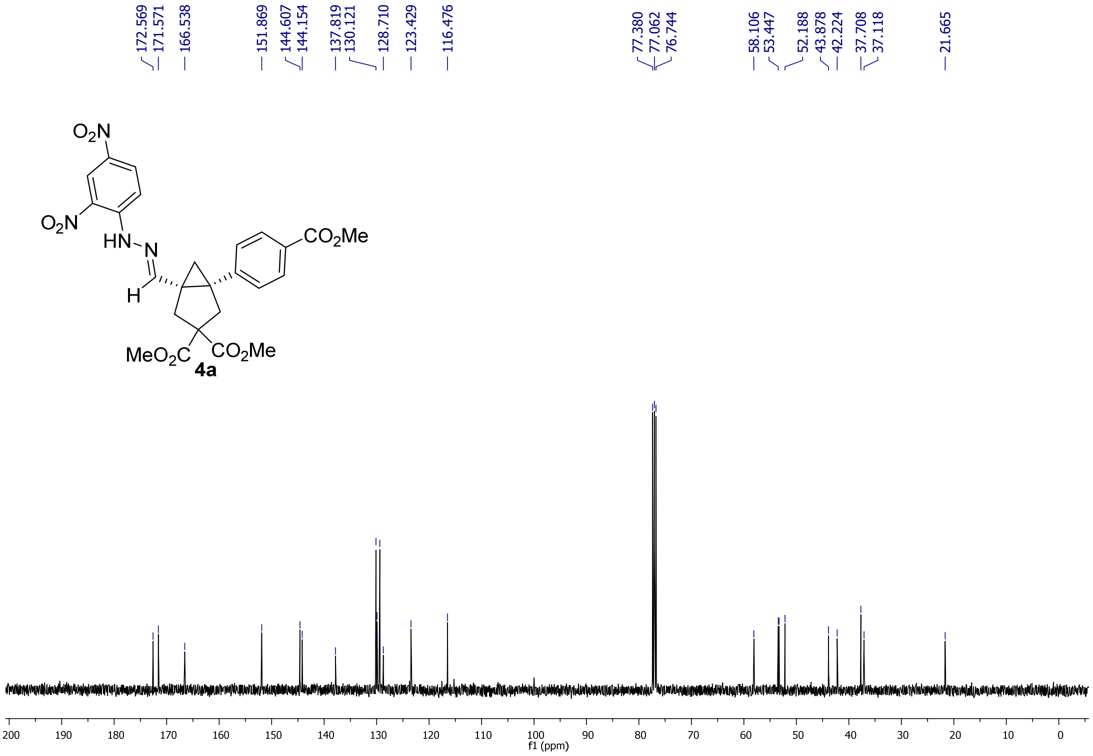

Supplementary Figure 96. ^{13}C NMR of **4a**

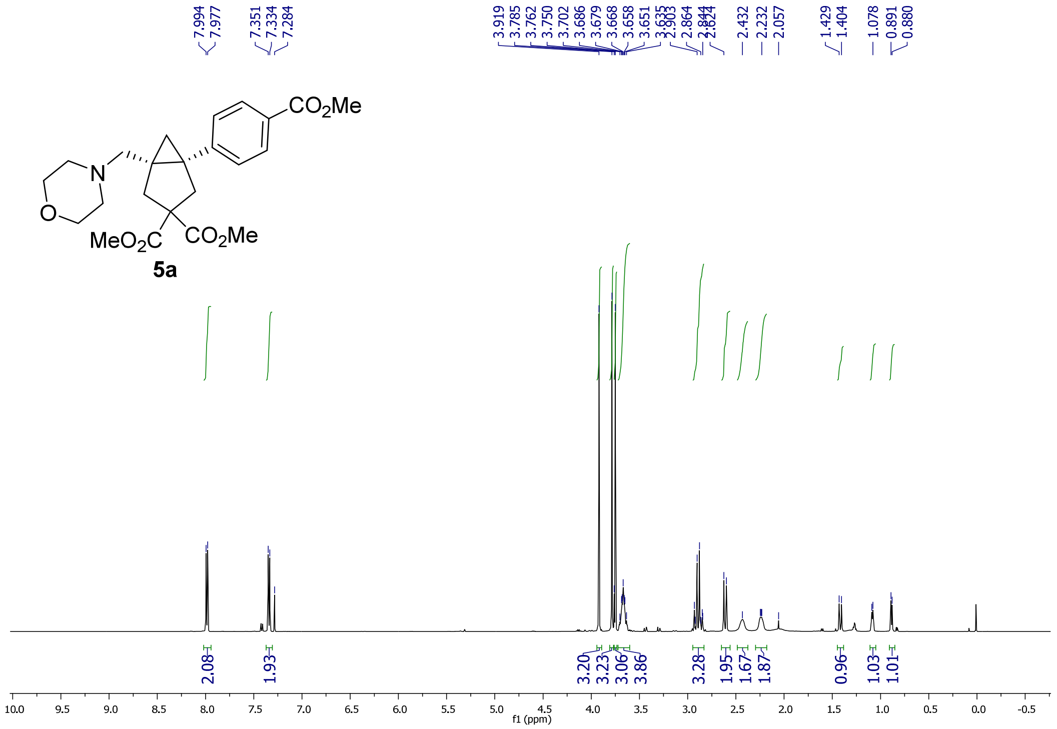

Supplementary Figure 97. ^{1}H NMR of **5a**

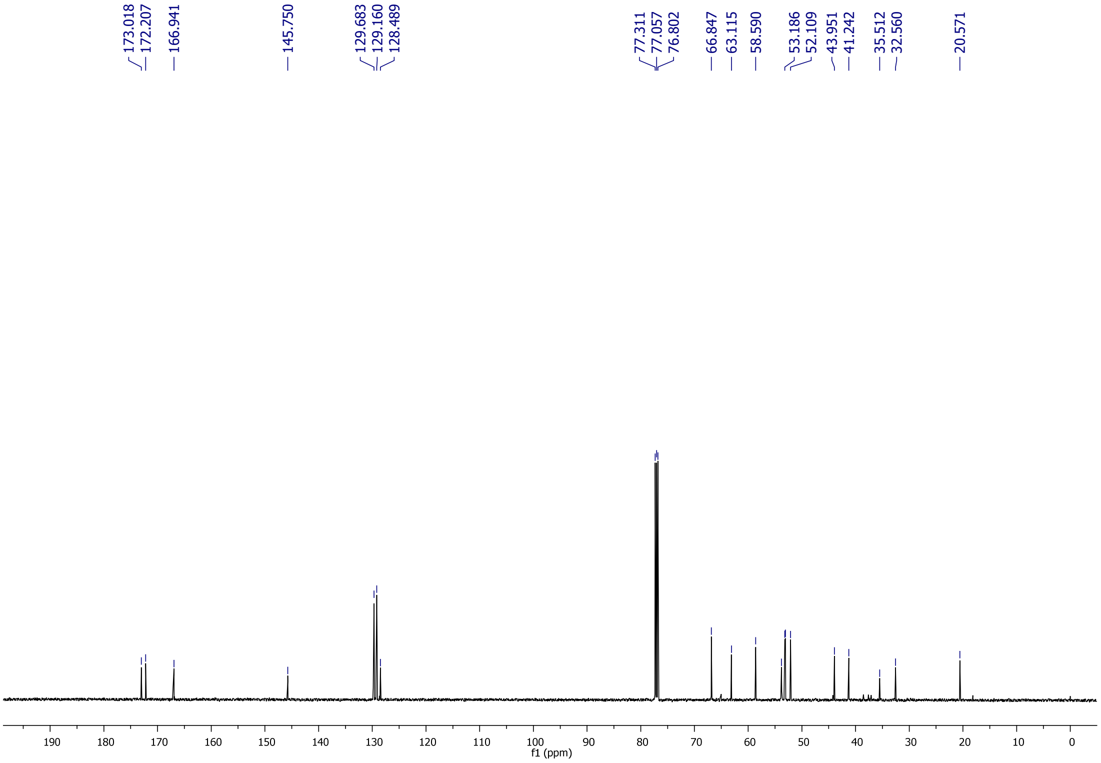

Supplementary Figure 98. ^{13}C NMR of **5a**

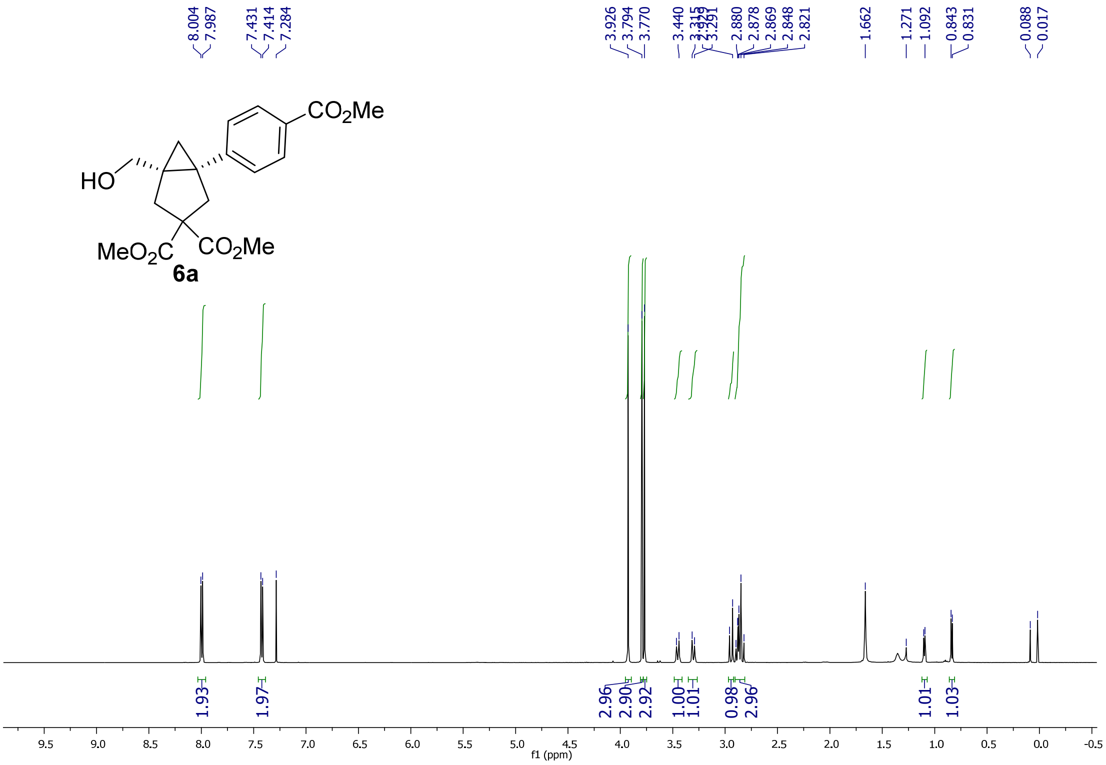

Supplementary Figure 99. ^{1}H NMR of **6a**

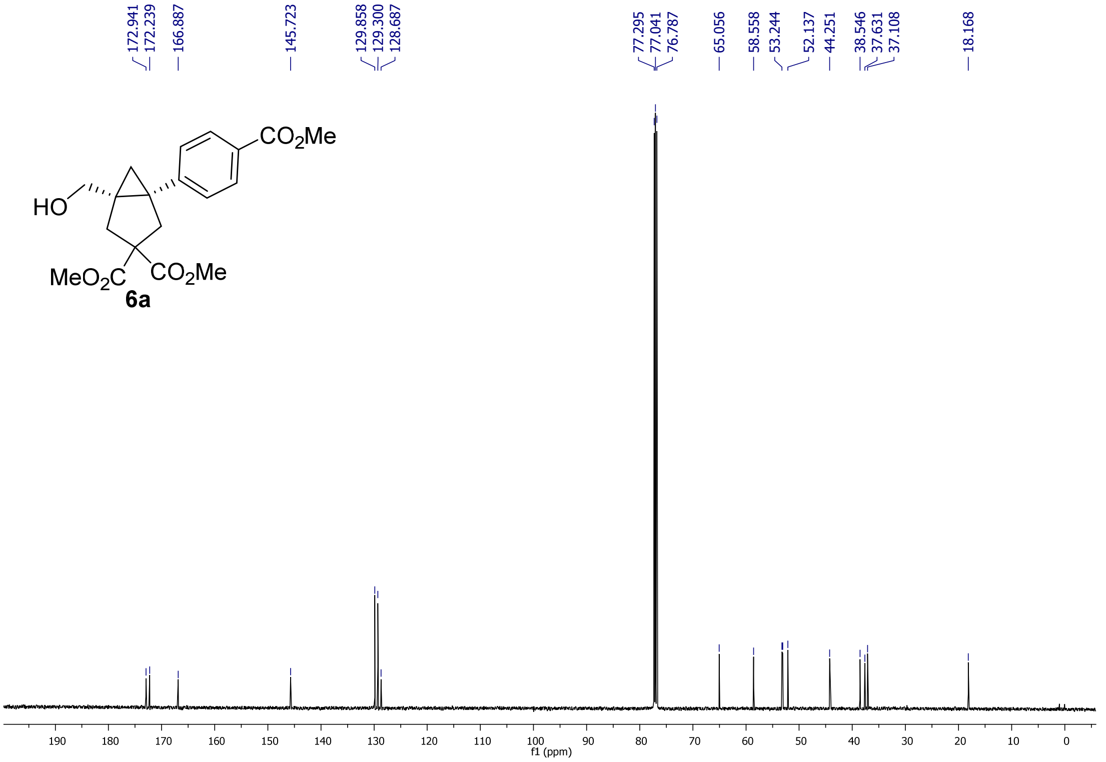

Supplementary Figure 100. ^{13}C NMR of **6a**

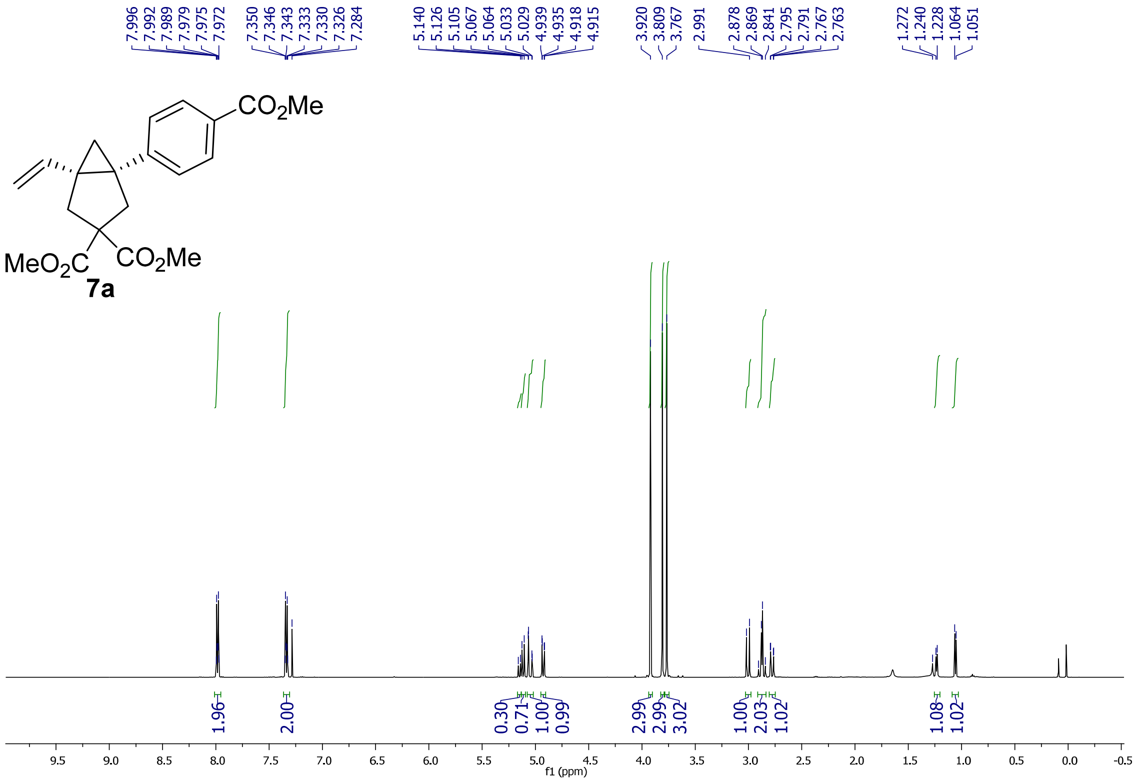

Supplementary Figure 101. ^{1}H NMR of **7a**

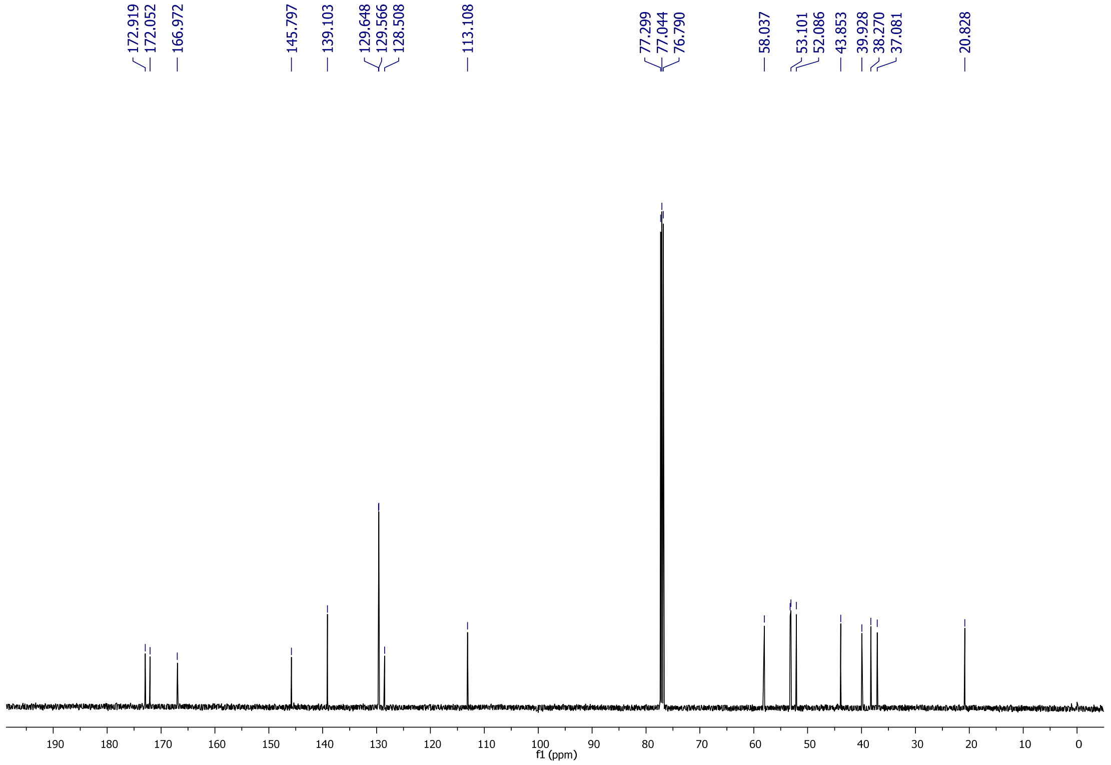

Supplementary Figure 102. ^{13}C NMR of **7a**

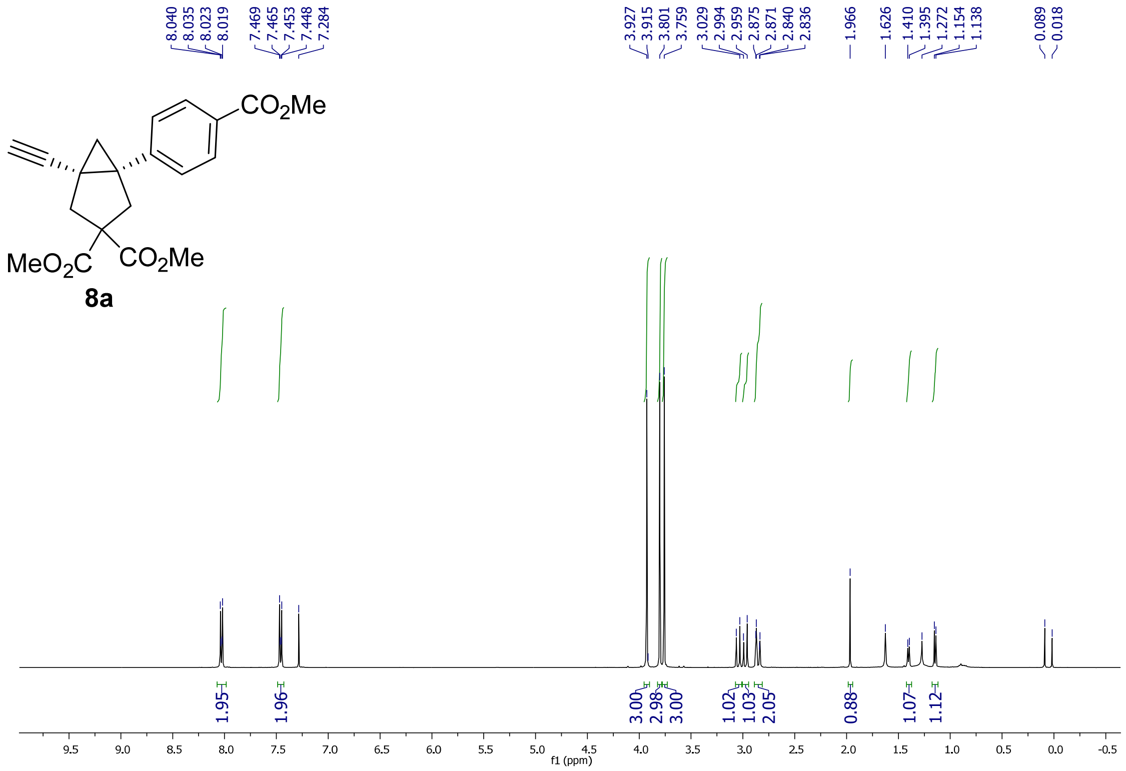

Supplementary Figure 103. ^{1}H NMR of **8a**

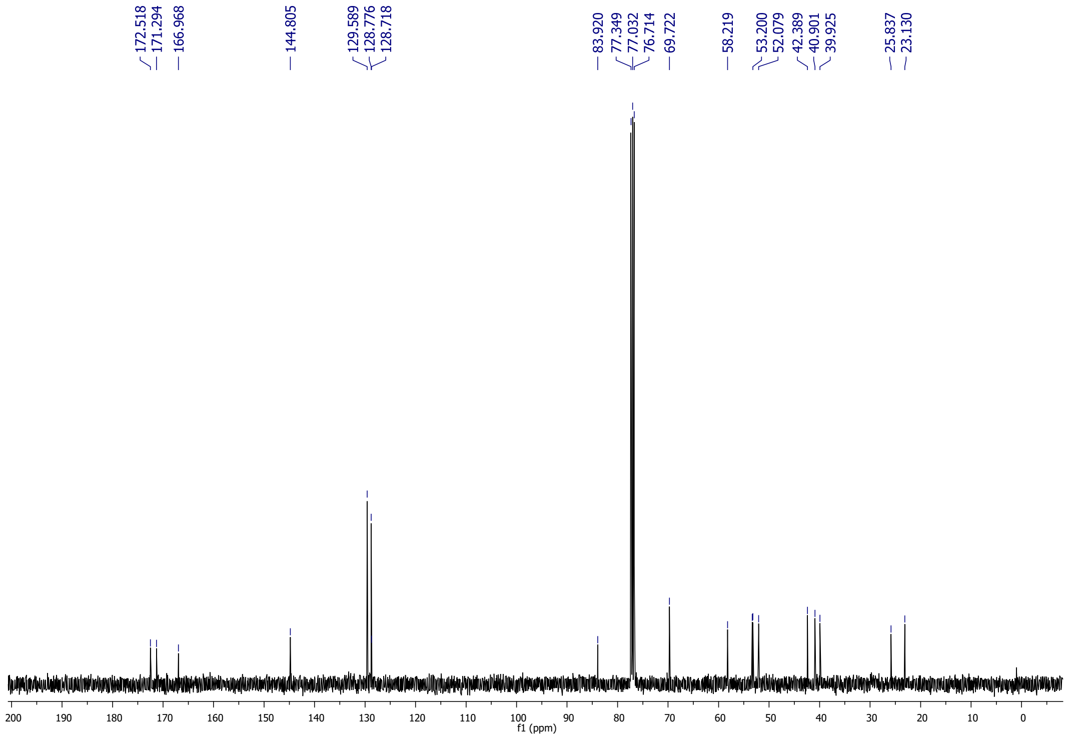

Supplementary Figure 104. ^{13}C NMR of **8a**

Supplementary Figures 105. HPLC spectra for racemic and chiral **2a**

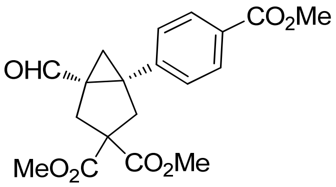

2a HPLC condition: Chiralcel IA, *i*-PrOH/*n*-hexane = 90/10, flow rate 1.0 mL/min. λ = 254 nm, t(minor) = 18.28 min, t(major) = 20.3 min, 95:5 er.

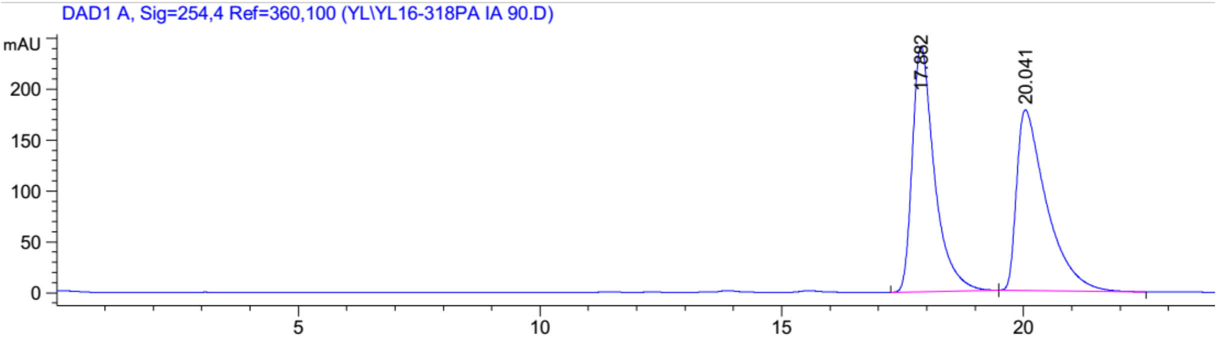

Peak #	RetTime [min]	Type	Width [min]	Area [mAU*s]	Height [mAU]	Area %
1	17.882	BB	0.4775	7675.08447	241.04300	50.2230
2	20.041	BB	0.6280	7606.92188	177.49545	49.7770

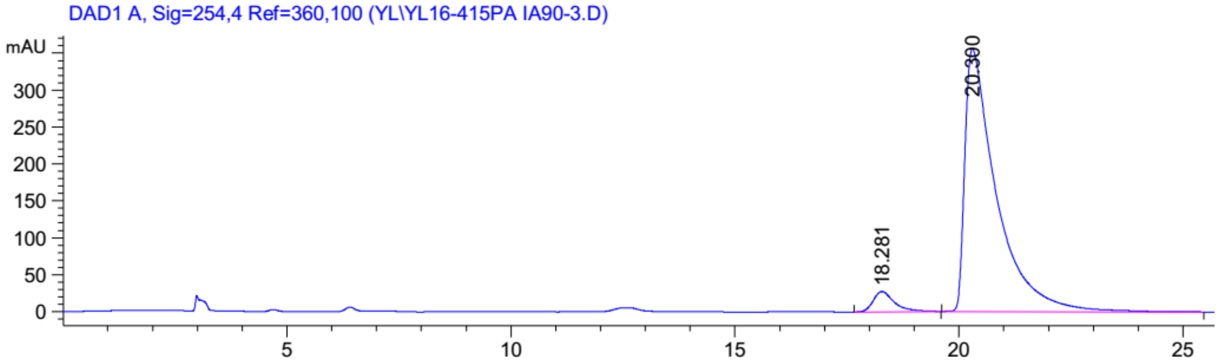

Peak #	RetTime [min]	Type	Width [min]	Area [mAU*s]	Height [mAU]	Area %
1	18.281	BB	0.4942	895.80353	27.34633	4.8999
2	20.300	BB	0.6918	1.73862e4	355.98419	95.1001

Supplementary Figures 106. HPLC spectra for racemic and chiral **2b**

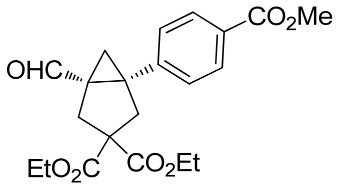

2b HPLC condition: Chiralcel IA, *i*-PrOH/*n*-hexane = 90/10, flow rate 1.0 mL/min. λ = 230 nm, t(minor) = 15.03 min, t(major) = 16.89 min, 92.5:7.5 er.

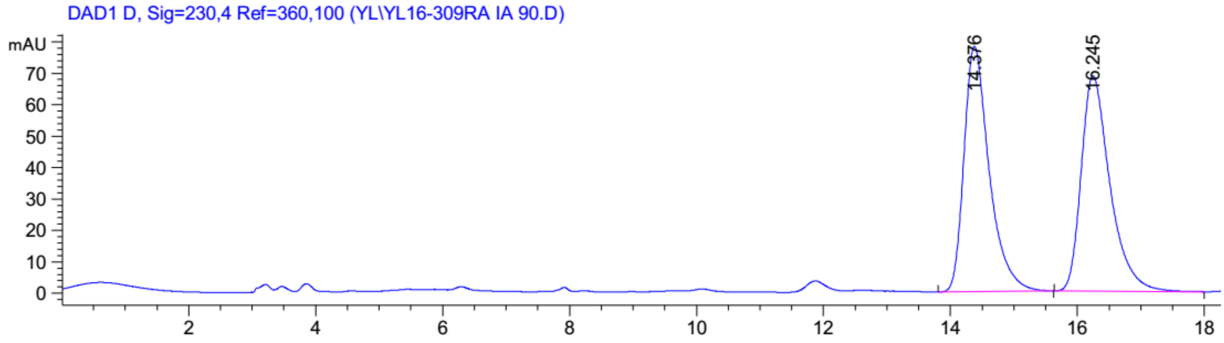

Peak #	RetTime [min]	Type	Width [min]	Area [mAU*s]	Height [mAU]	Area %
1	14.376	BB	0.4162	2154.93213	78.03257	49.9785
2	16.245	BB	0.4754	2156.78564	68.48622	50.0215

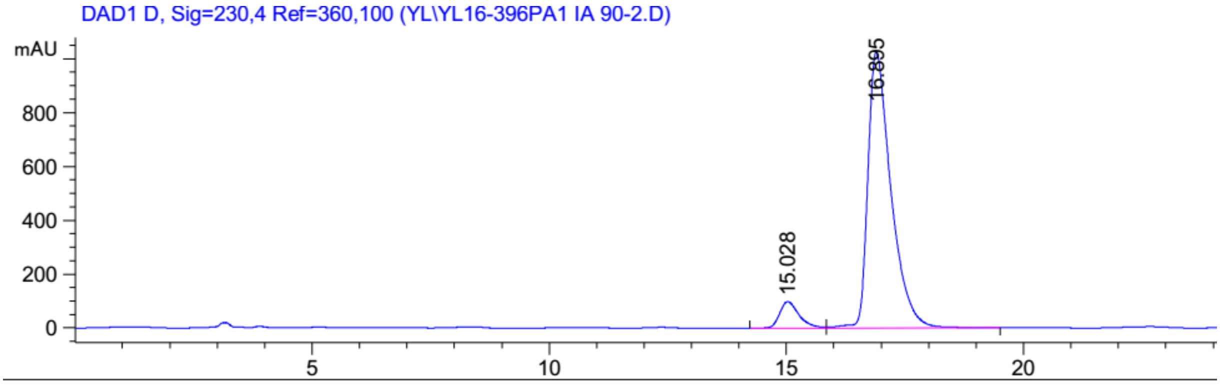

Peak #	RetTime [min]	Type	Width [min]	Area [mAU*s]	Height [mAU]	Area %
1	15.028	BV	0.4250	2772.84766	98.33008	7.4079
2	16.895	VB	0.5012	3.46579e4	1028.28650	92.5921

Supplementary Figures 107. HPLC spectra for racemic and chiral **2c**

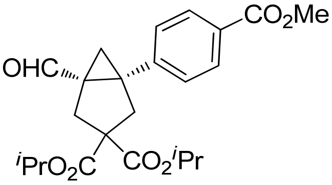

2c HPLC condition: Chiralcel OD-H, *i*-PrOH/*n*-hexane = 90/10, flow rate 1.0 mL/min. λ = 230 nm, t(minor) = 8.31 min, t(major) = 9.21 min, 92.5:7.5 er.

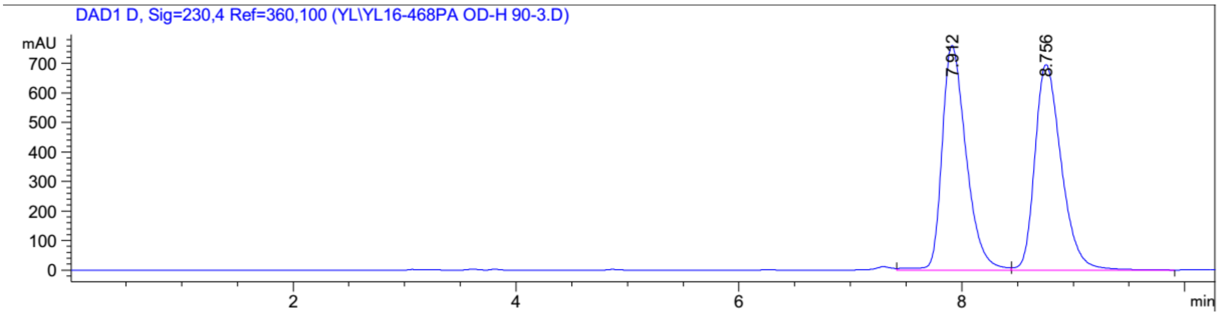

Signal 4: DAD1 D, Sig=230,4 Ref=360,100

Peak #	RetTime [min]	Type	Width [min]	Area [mAU*s]	Height [mAU]	Area %
1	7.912	VV	0.2310	1.14197e4	761.46997	49.8793
2	8.756	VB	0.2542	1.14750e4	696.51306	50.1207

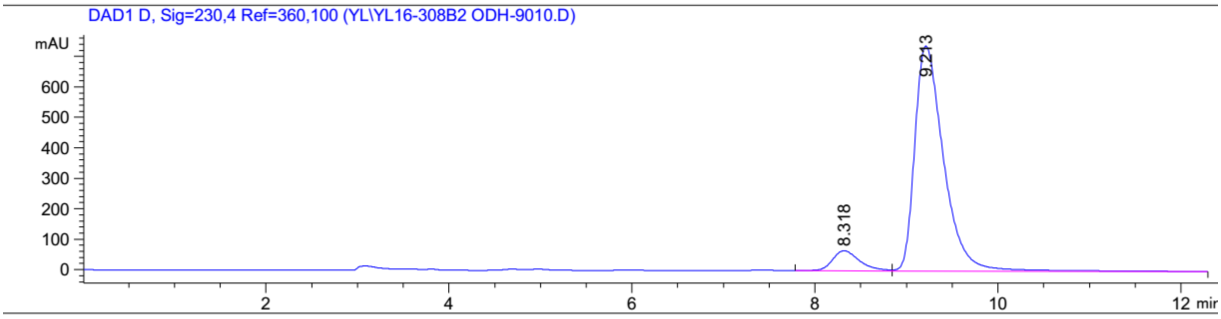

Signal 3: DAD1 D, Sig=230,4 Ref=360,100

Peak #	RetTime [min]	Type	Width [min]	Area [mAU*s]	Height [mAU]	Area %
1	8.318	BV	0.3093	1351.22937	65.88388	7.6945
2	9.213	VBA	0.3340	1.62097e4	738.82660	92.3055

Supplementary Figures 108. HPLC spectra for racemic and chiral **2d**

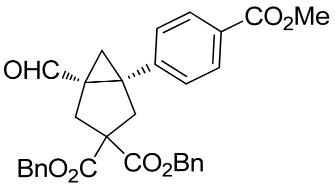

2d HPLC condition: Chiralcel IA, *i*-PrOH/*n*-hexane = 90/10, flow rate 1.0 mL/min. λ = 230 nm, t(minor) = 17.09 min, t(major) = 19.84 min, 93:7 er.

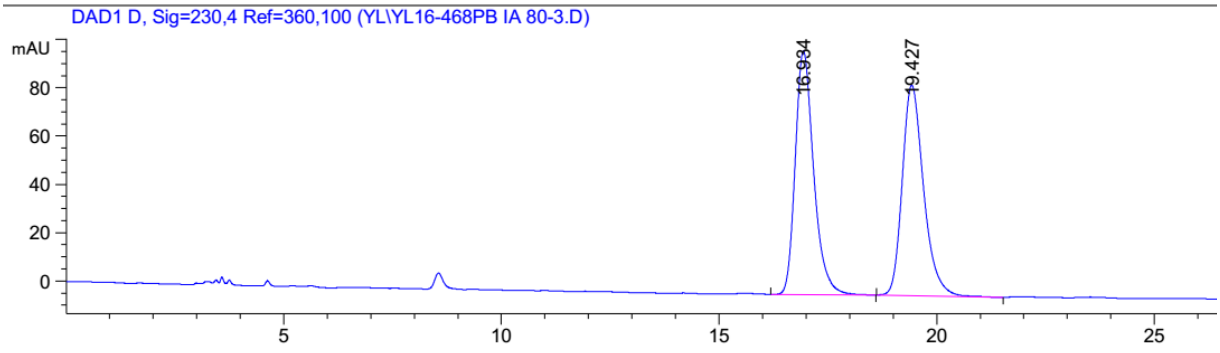

Signal 4: DAD1 D, Sig=230,4 Ref=360,100

Peak #	RetTime [min]	Type	Width [min]	Area [mAU*s]	Height [mAU]	Area %
1	16.934	BB	0.4471	2967.07739	100.89144	49.9873
2	19.427	BB	0.5191	2968.58838	87.22259	50.0127

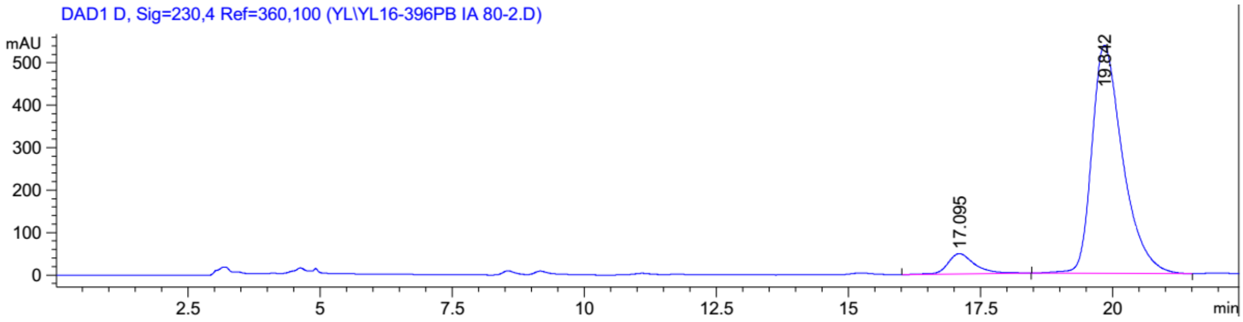

Signal 3: DAD1 D, Sig=230,4 Ref=360,100

Peak #	RetTime [min]	Type	Width [min]	Area [mAU*s]	Height [mAU]	Area %
1	17.095	BB	0.5276	1696.90051	47.86060	7.1979
2	19.842	BB	0.6149	2.18781e4	537.44922	92.8021

Supplementary Figures 109. HPLC spectra for racemic and chiral **2f**

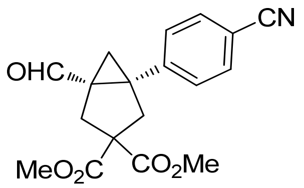

2f HPLC condition: Chiralcel AD-H, *i*-PrOH/*n*-hexane = 80/20, flow rate 1.0 mL/min. λ = 230 nm, t(major) = 12.58 min, t(minor) = 15.27 min, 91.5:8.5 er.

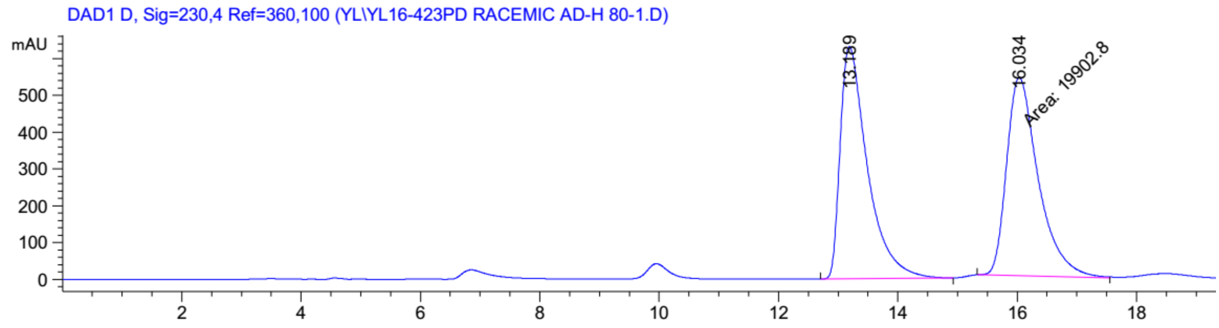

Peak #	RetTime [min]	Type	Width [min]	Area [mAU*s]	Height [mAU]	Area %
1	13.189	BB	0.4740	2.00149e4	631.02234	50.1404
2	16.034	MM	0.6152	1.99028e4	539.17731	49.8596

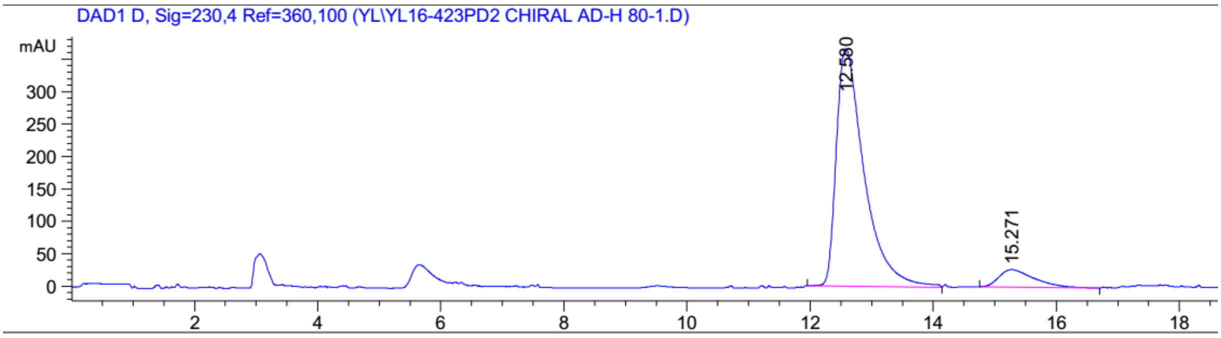

Peak #	RetTime [min]	Type	Width [min]	Area [mAU*s]	Height [mAU]	Area %
1	12.580	BV	0.4680	1.15030e4	366.64801	91.6525
2	15.271	BV	0.5830	1047.67078	26.74617	8.3475

Supplementary Figures 110. HPLC spectra for racemic and chiral **2g**

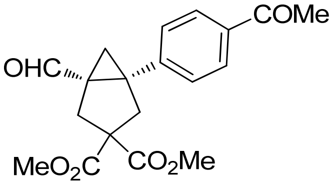

2g HPLC condition: Chiralcel OD-H, *i*-PrOH/*n*-hexane = 80/20, flow rate 1.0 mL/min. λ = 230 nm, t(major) = 16.17 min, t(minor) = 18.64 min, 93.5:6.5 er.

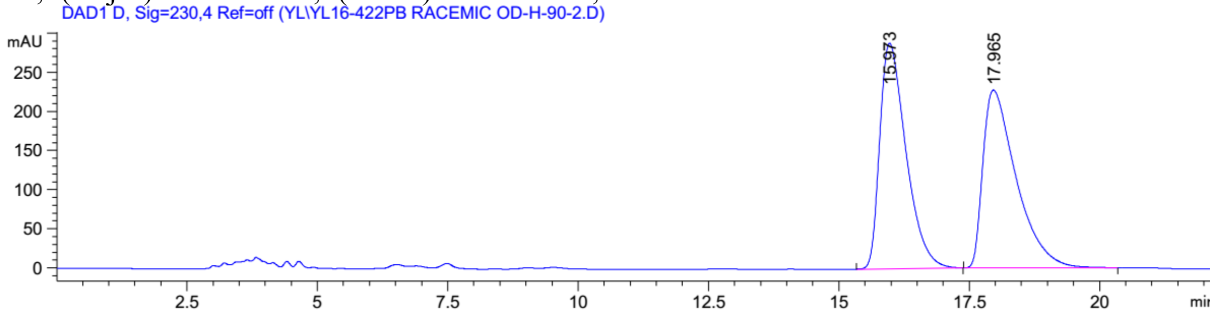

Peak #	RetTime [min]	Type	Width [min]	Area [mAU*s]	Height [mAU]	Area %
1	15.973	BB	0.5318	1.00748e4	288.25931	49.9537
2	17.965	BB	0.6604	1.00935e4	227.17387	50.0463

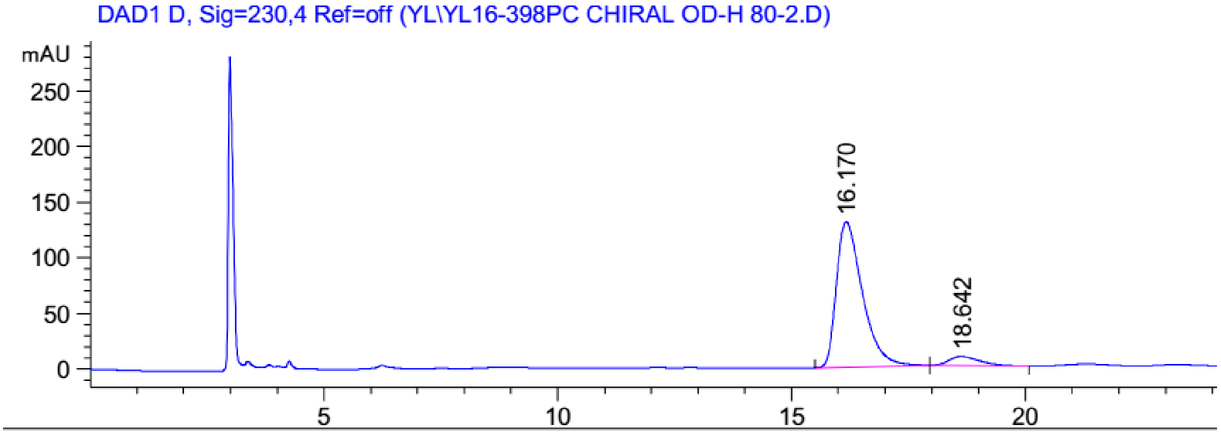

Peak #	RetTime [min]	Type	Width [min]	Area [mAU*s]	Height [mAU]	Area %
1	16.170	BB	0.5844	5043.82324	130.64757	93.2336
2	18.642	BB	0.6147	366.05283	8.04768	6.7664

Supplementary Figures 111. HPLC spectra for racemic and chiral **2h**

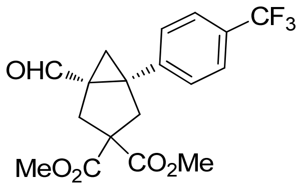

2h HPLC condition: Chiralcel OD-H, *i*-PrOH/*n*-hexane = 80/20, flow rate 1.0 mL/min. λ = 230 nm, t(major) = 6.23 min, t(minor) = 7.47 min, 94.5:5.5 er.

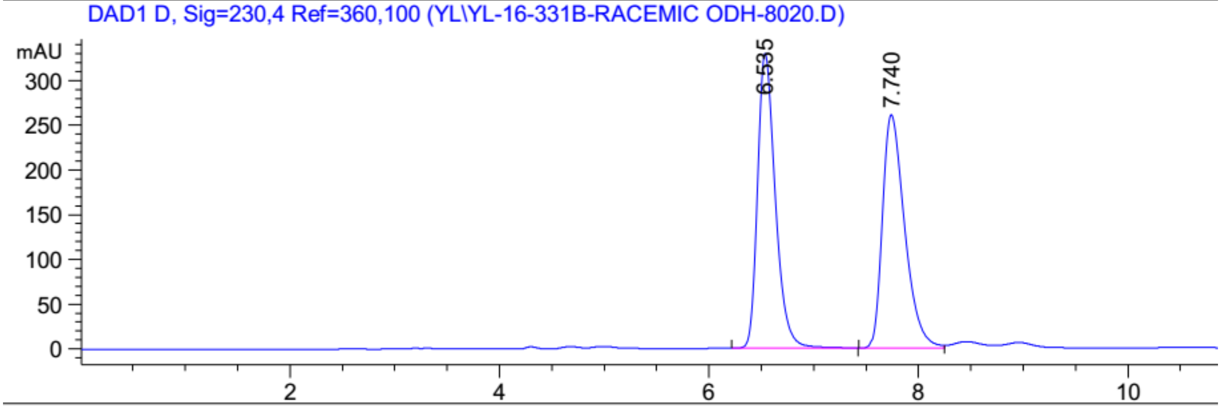

Peak #	RetTime [min]	Type	Width [min]	Area [mAU*s]	Height [mAU]	Area %
1	6.535	BB	0.1830	3929.28442	329.88565	50.2585
2	7.740	BV	0.2298	3888.86279	261.03091	49.7415

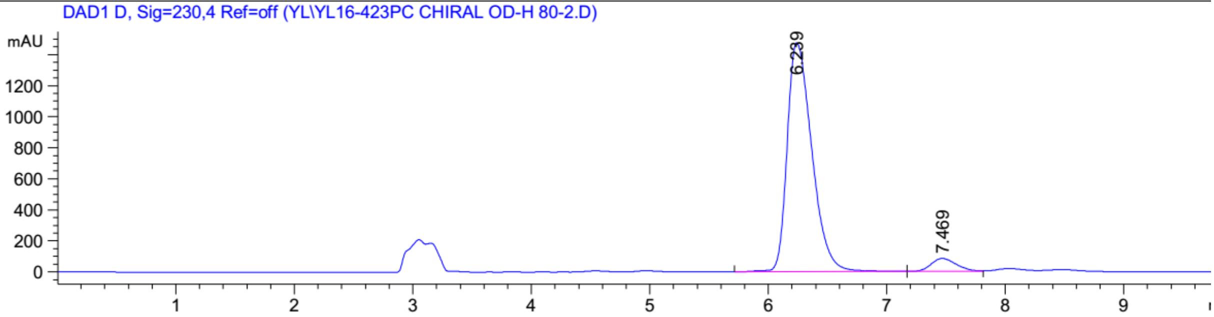

Peak #	RetTime [min]	Type	Width [min]	Area [mAU*s]	Height [mAU]	Area %
1	6.239	BB	0.2242	2.12565e4	1474.16748	94.4643
2	7.469	BV	0.2330	1245.66272	83.06308	5.5357

Supplementary Figures 112. HPLC spectra for racemic and chiral **2m**

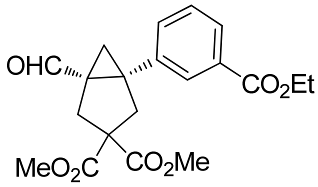

2m HPLC condition: Chiralcel AS-H, *i*-PrOH/*n*-hexane = 95/5, flow rate 0.8 mL/min. λ = 230 nm, t(major) = 40.79 min, t(minor) = 46.21 min, 93:7 er.

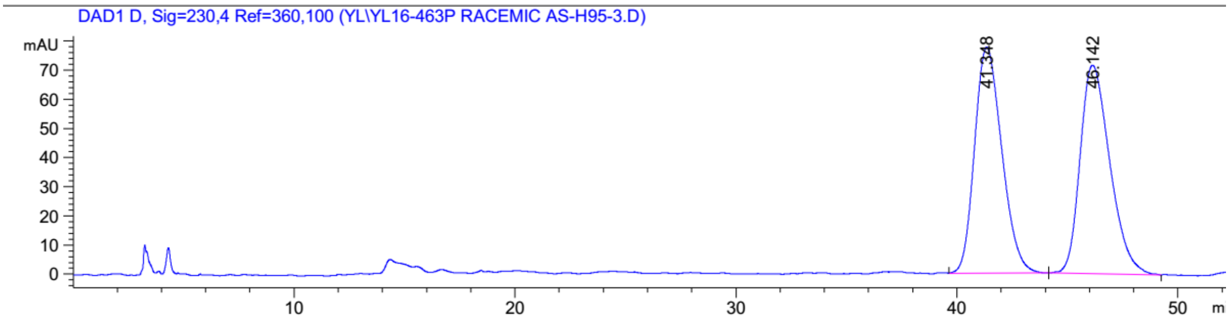

Signal 4: DAD1 D, Sig=230,4 Ref=360,100

Peak #	RetTime [min]	Type	Width [min]	Area [mAU*s]	Height [mAU]	Area %
1	41.348	BB	1.3309	6637.16064	77.51757	49.7809
2	46.142	BB	1.4491	6695.58252	71.59274	50.2191

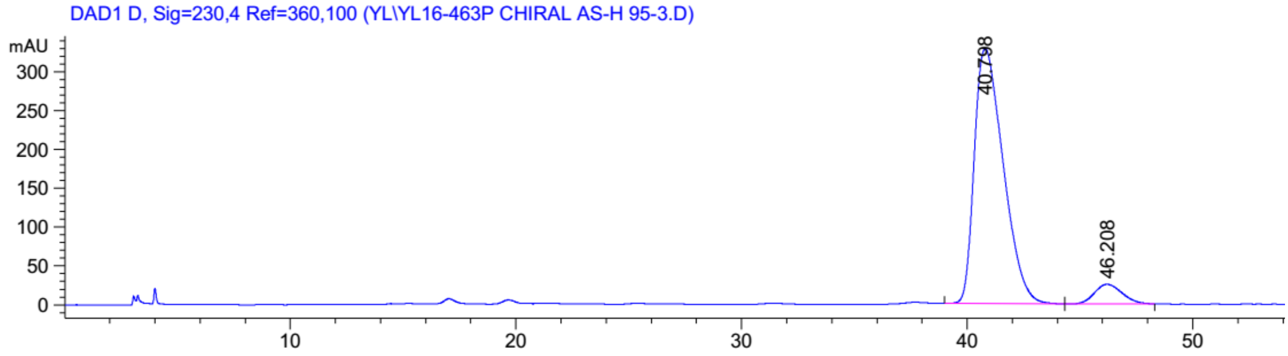

Signal 4: DAD1 D, Sig=230,4 Ref=360,100

Peak #	RetTime [min]	Type	Width [min]	Area [mAU*s]	Height [mAU]	Area %
1	40.798	BB	1.3969	2.92403e4	328.35239	93.1198
2	46.208	BB	1.2885	2160.45166	25.29346	6.8802

Supplementary Figures 113. HPLC spectra for racemic and chiral **2n**

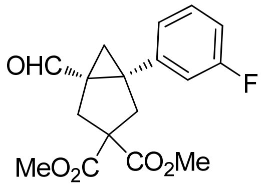

2n HPLC condition: Chiralcel AS-H, *i*-PrOH/*n*-hexane = 80/20, flow rate 1.0 mL/min. λ = 230 nm, t(major) = 10.79 min, t(minor) = 13.02 min, 93.5:6.5 er.

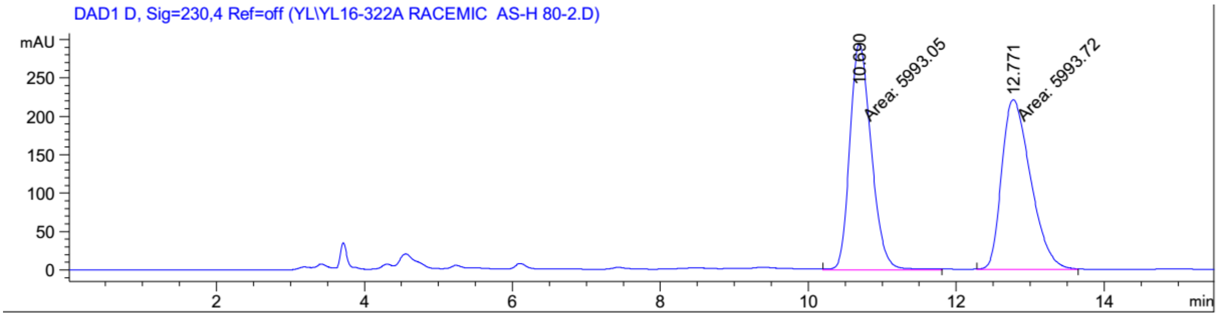

Peak #	RetTime [min]	Type	Width [min]	Area [mAU*s]	Height [mAU]	Area %
1	10.690	MM	0.3393	5993.04736	294.33997	49.9972
2	12.771	MM	0.4525	5993.72021	220.74911	50.0028

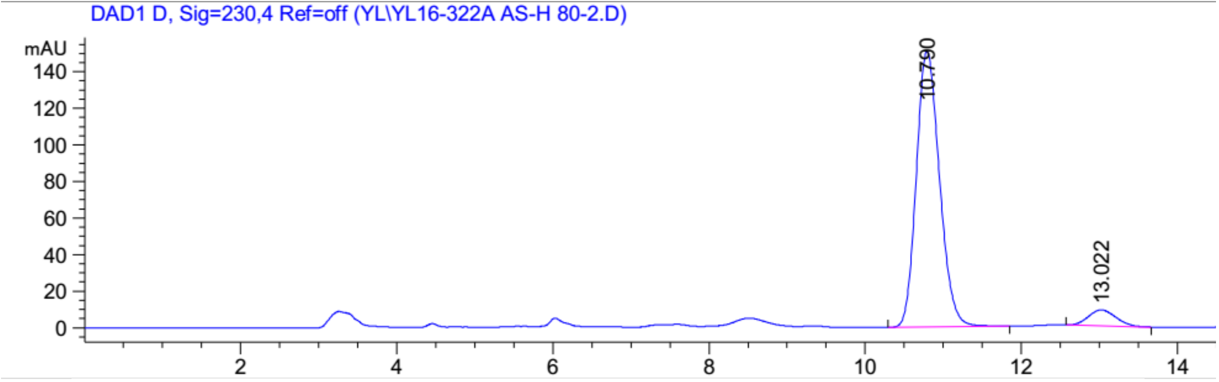

Peak #	RetTime [min]	Type	Width [min]	Area [mAU*s]	Height [mAU]	Area %
1	10.790	BB	0.3206	3112.76196	150.93016	93.6101
2	13.022	BB	0.3780	212.47960	8.66735	6.3899

Supplementary Figures 114. HPLC spectra for racemic and chiral **2o**

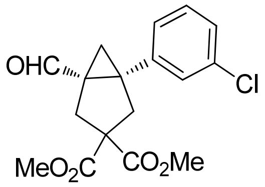

2o HPLC condition: Chiralcel IA, *i*-PrOH/*n*-hexane = 95/5, flow rate 0.8 mL/min. λ = 214 nm, t(minor) = 13.51 min, t(major) = 16.32 min, 90.5:9.5 er.

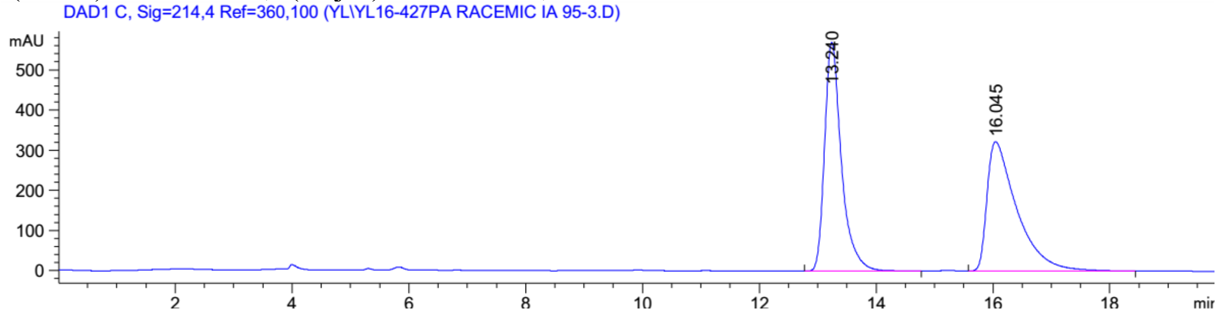

Signal 3: DAD1 C, Sig=214,4 Ref=360,100

Peak #	RetTime [min]	Type	Width [min]	Area [mAU*s]	Height [mAU]	Area %
1	13.240	BB	0.3018	1.14098e4	569.38721	49.8665
2	16.045	BB	0.5172	1.14709e4	322.34048	50.1335

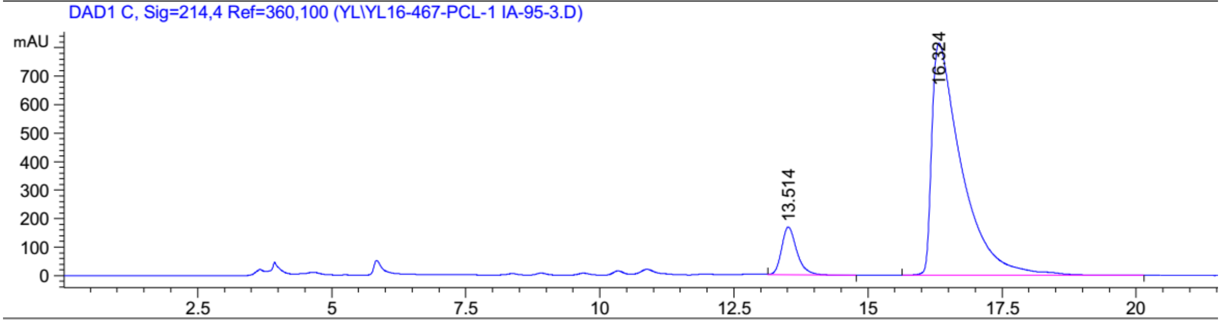

Signal 3: DAD1 C, Sig=214,4 Ref=360,100

Peak #	RetTime [min]	Type	Width [min]	Area [mAU*s]	Height [mAU]	Area %
1	13.514	VB	0.3014	3341.49756	168.43681	9.5724
2	16.324	BB	0.5539	3.15660e4	814.90356	90.4276

Supplementary Figures 115. HPLC spectra for racemic and chiral **2p**

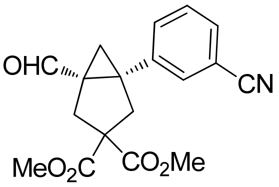

2p HPLC condition: Chiralcel AD-H, *i*-PrOH/*n*-hexane = 80/20, flow rate 1.0 mL/min. λ = 214 nm, t(minor) = 10.2 min, t(major) = 11.93 min, 95:5 er.

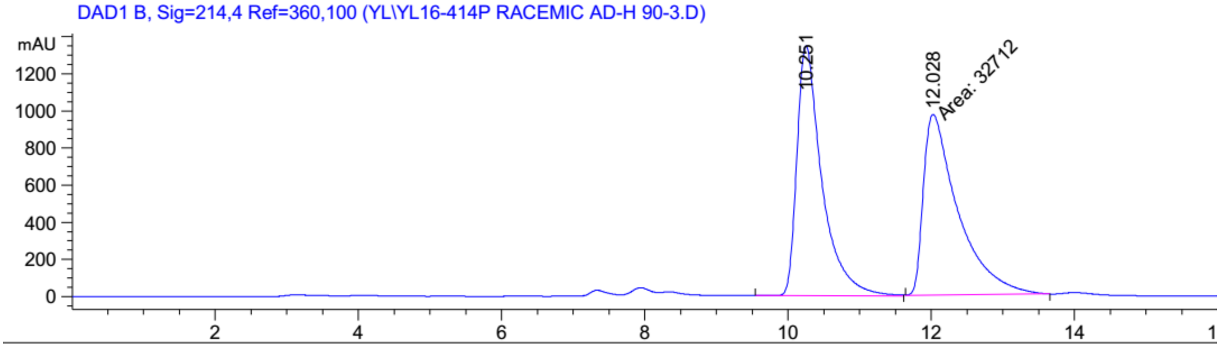

Peak #	RetTime [min]	Type	Width [min]	Area [mAU*s]	Height [mAU]	Area %
1	10.251	BV	0.3575	3.27224e4	1346.55505	50.0080
2	12.028	MM	0.5600	3.27120e4	973.65198	49.9920

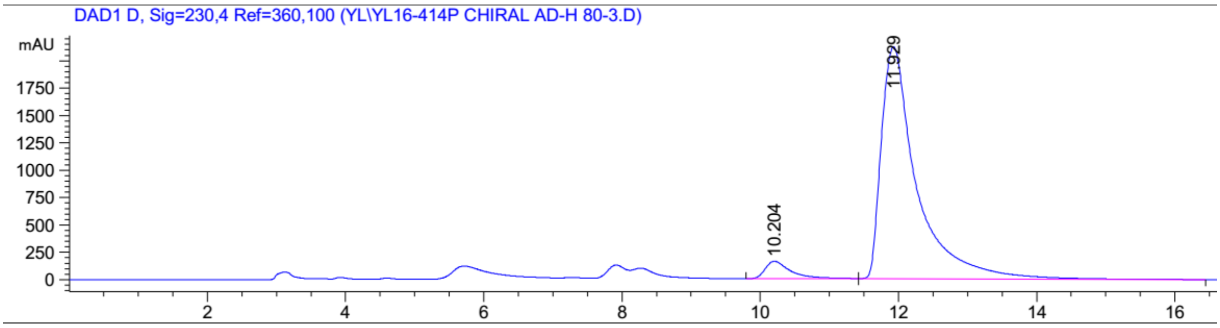

Peak #	RetTime [min]	Type	Width [min]	Area [mAU*s]	Height [mAU]	Area %
1	10.204	BV	0.3830	4186.06348	159.07127	5.2270
2	11.929	VBA	0.5277	7.58990e4	2119.86475	94.7730

Supplementary Figures 116. HPLC spectra for racemic and chiral **2q**

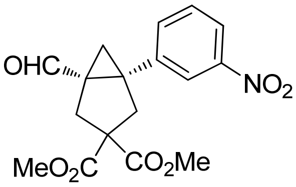

2q HPLC condition: Chiralcel OD-H, *i*-PrOH/*n*-hexane = 80/20, flow rate 1.0 mL/min. λ = 254 nm, t(major) = 16.26 min, t(minor) = 18.21 min, 92:8 er.

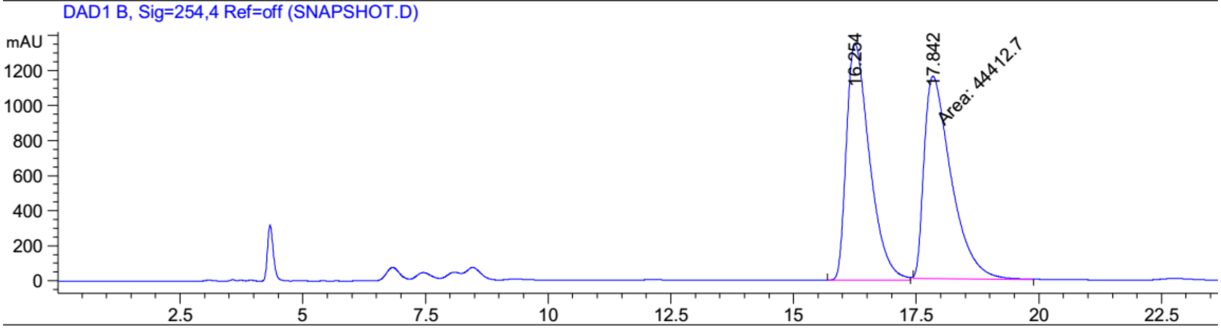

Peak #	RetTime [min]	Type	Width [min]	Area [mAU*s]	Height [mAU]	Area %
1	16.254	VV	0.5015	4.44082e4	1350.84949	49.9975
2	17.842	MM	0.6402	4.44127e4	1156.27332	50.0025

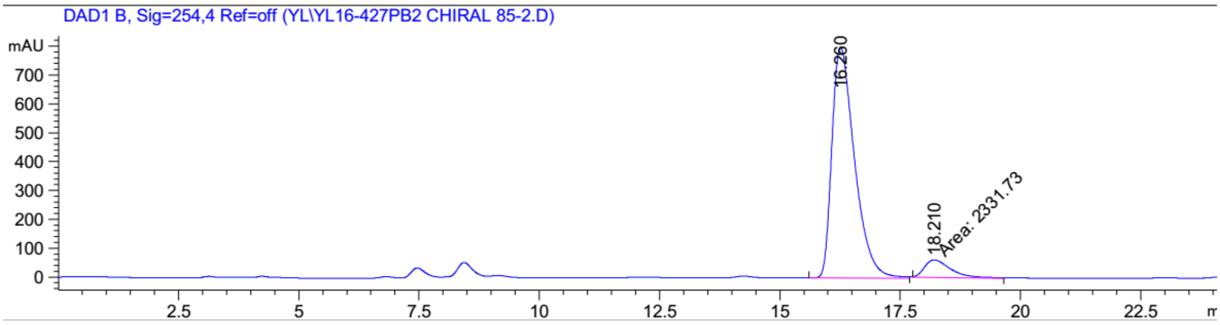

Peak #	RetTime [min]	Type	Width [min]	Area [mAU*s]	Height [mAU]	Area %
1	16.260	BV	0.5011	2.61372e4	800.08374	91.8096
2	18.210	MM	0.6345	2331.72729	61.24922	8.1904

Supplementary Figures 117. HPLC spectra for racemic and chiral **2s**

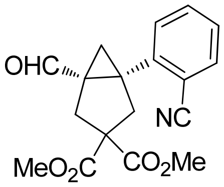

2s HPLC condition: Chiralcel AD-H, *i*-PrOH/*n*-hexane = 80/20, flow rate 1.0 mL/min. λ = 210 nm, t(minor) = 10.28 min, t(major) = 15.0 min, 93:7 er.

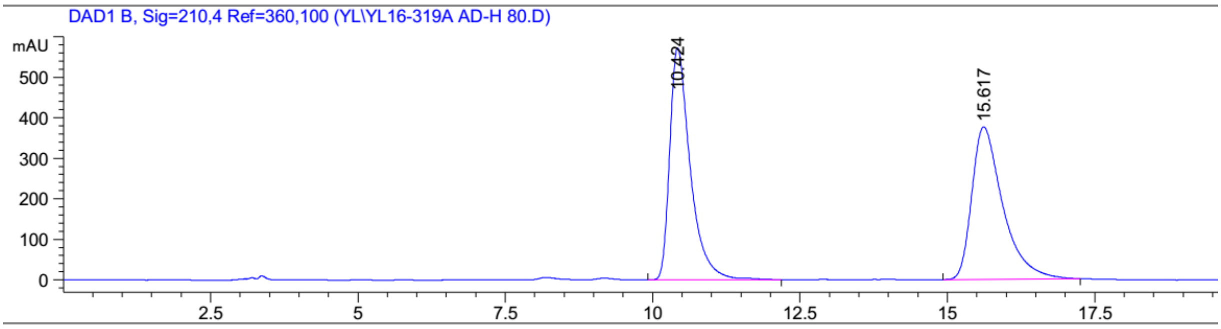

Peak #	RetTime [min]	Type	Width [min]	Area [mAU*s]	Height [mAU]	Area %
1	10.424	BB	0.3627	1.38542e4	571.75824	50.2009
2	15.617	BB	0.5483	1.37434e4	376.07709	49.7991

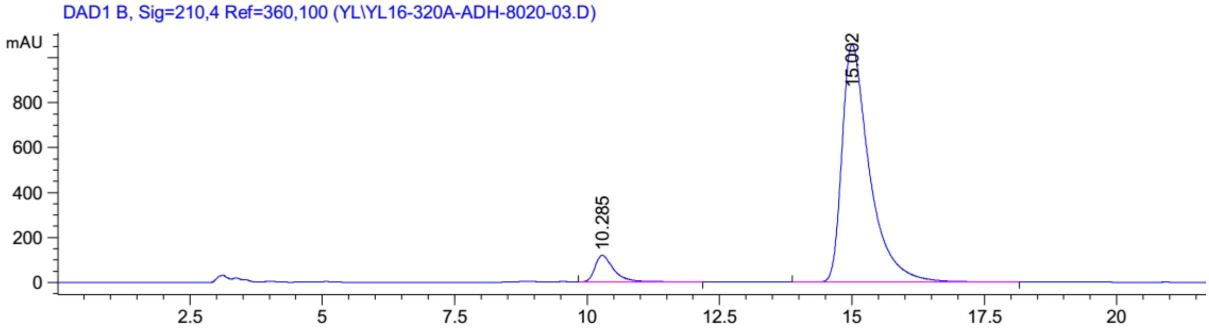

Peak #	RetTime [min]	Type	Width [min]	Area [mAU*s]	Height [mAU]	Area %
1	10.285	VB	0.3760	2998.97241	118.99097	7.2917
2	15.002	BB	0.5327	3.81298e4	1057.47034	92.7083

Supplementary Figures 118. HPLC spectra for racemic and chiral **2u**

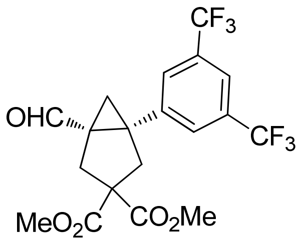

2u HPLC condition: Chiralcel AS-H, *i*-PrOH/*n*-hexane = 95/5, flow rate 0.8 mL/min. λ = 230 nm, t(minor) = 7.29 min, t(major) = 8.27 min, 89.5:10.5 er.

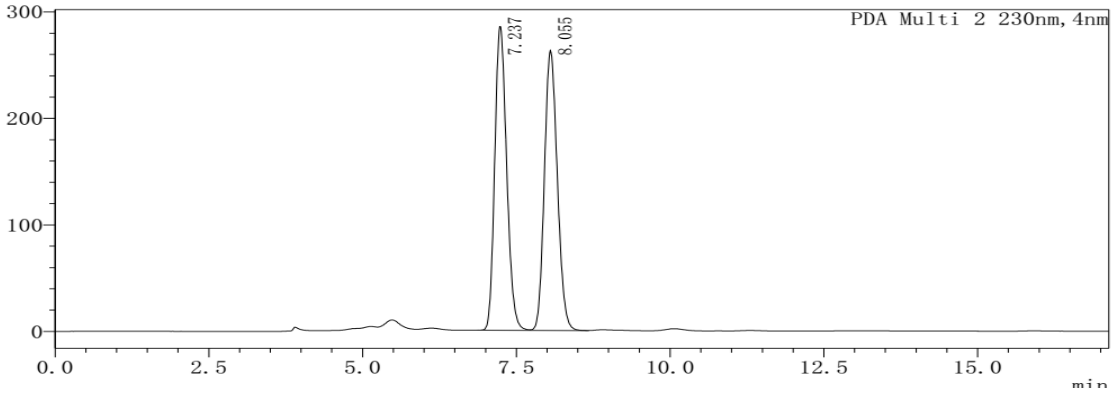

PDA Ch2 230nm

峰号	保留时间	面积	高度	面积%
1	7.237	3842671	285119	49.948
2	8.055	3850661	262638	50.052
总计		7693332	547757	100.000

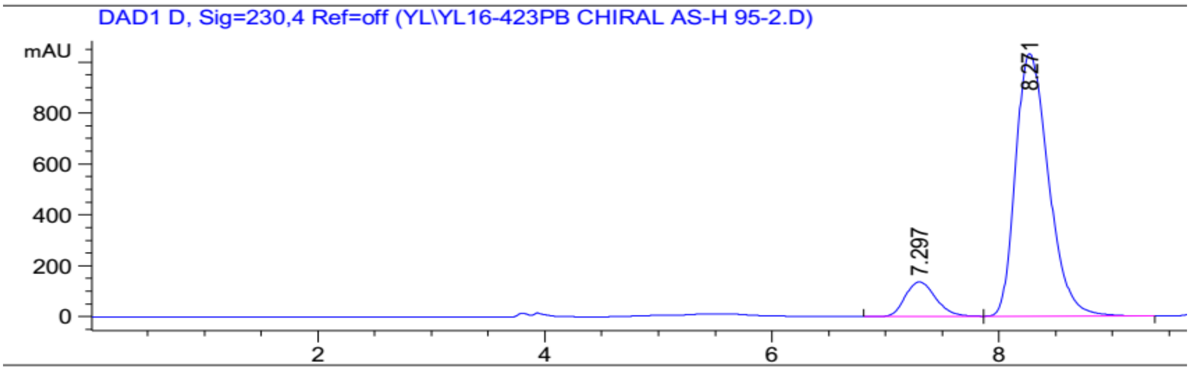

Signal 4: DAD1 D, Sig=230,4 Ref=off

Peak #	RetTime [min]	Type	Width [min]	Area [mAU*s]	Height [mAU]	Area %
1	7.297	BV	0.2947	2546.72729	135.87863	10.8838
2	8.271	VB	0.3157	2.08526e4	1032.02454	89.1162

Supplementary Figures 119. HPLC spectra for racemic and chiral **2x**

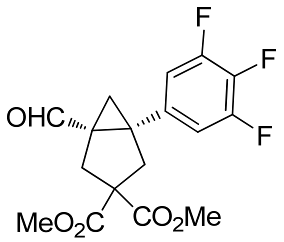

2x HPLC condition: Chiralcel AS-H, *i*-PrOH/*n*-hexane = 80/20, flow rate 1.0 mL/min. λ = 230 nm,

t(minor) = 7.34 min, t(major) = 8.47 min, 92.5:7.5 er.

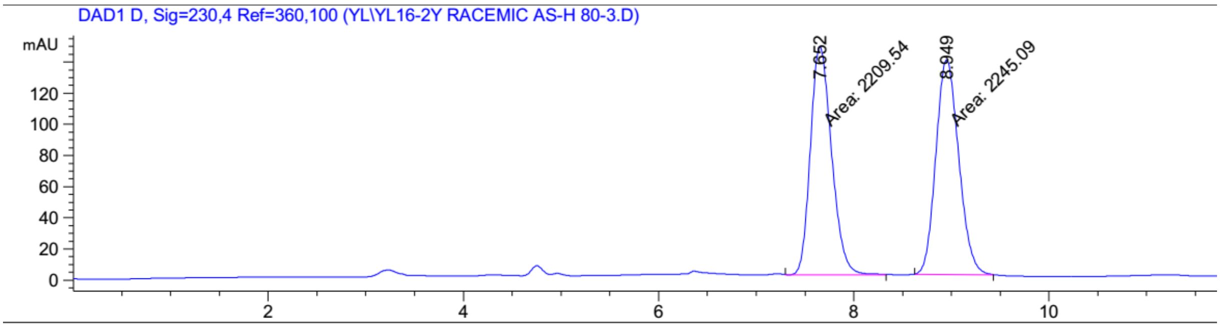

Signal 4: DAD1 D, Sig=230,4 Ref=360,100

Peak #	RetTime [min]	Type	Width [min]	Area [mAU*s]	Height [mAU]	Area %
1	7.652	MM	0.2513	2209.54028	146.55133	49.6010
2	8.949	MM	0.2713	2245.08594	137.89690	50.3990

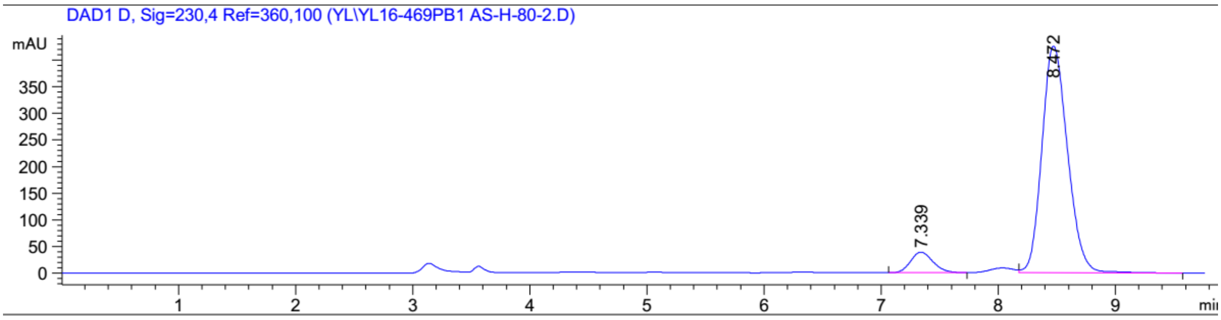

Signal 4: DAD1 D, Sig=230,4 Ref=360,100

Peak #	RetTime [min]	Type	Width [min]	Area [mAU*s]	Height [mAU]	Area %
1	7.339	BV	0.2080	513.36127	38.40315	7.3410
2	8.472	VB	0.2377	6479.69580	425.51111	92.6590

Supplementary Figures 120. HPLC spectra for racemic and chiral **2z**

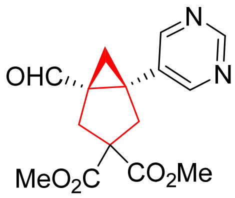

2z, HPLC condition: Chiralcel OD-H, *i*-PrOH/*n*-hexane = 80/20, flow rate 1.0 mL/min. λ = 210 nm, t(major) = 36.43 min, t(minor) = 39.51 min, 94.5:5.5 er.

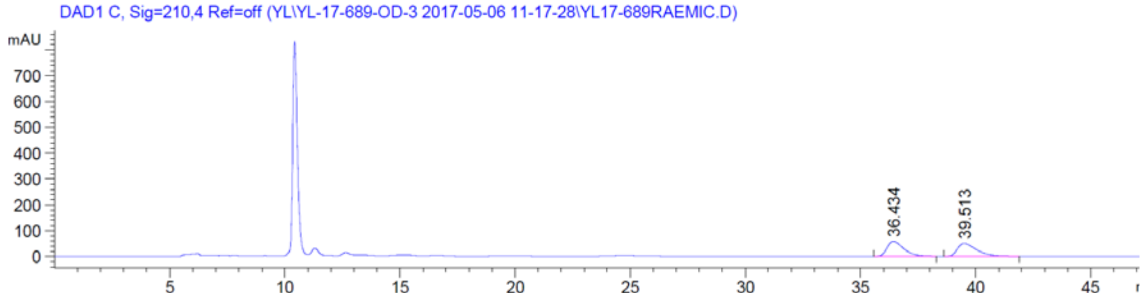

Signal 3: DAD1 C, Sig=210,4 Ref=off

Peak #	RetTime [min]	Type	Width [min]	Area [mAU*s]	Height [mAU]	Area %
1	36.434	BB	0.7285	2964.59399	58.77155	50.2346
2	39.513	BB	0.8151	2936.89868	50.30997	49.7654

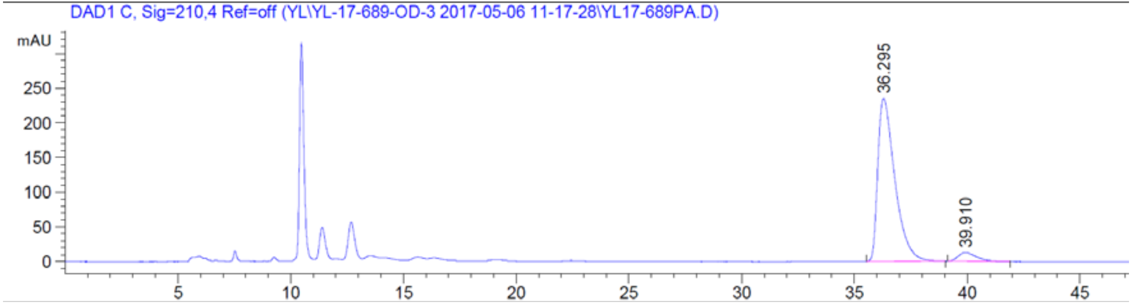

Signal 3: DAD1 C, Sig=210,4 Ref=off

Peak #	RetTime [min]	Type	Width [min]	Area [mAU*s]	Height [mAU]	Area %
1	36.295	BB	0.7668	1.22855e4	234.67386	94.4641
2	39.910	BB	0.6687	719.97455	12.67535	5.5359

Supplementary Figures 121. HPLC spectra for racemic and chiral **2aa**

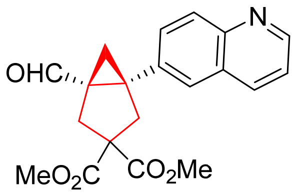

2aa, HPLC condition: Chiralcel AS-H, *i*-PrOH/*n*-hexane = 80/20, flow rate 1.0 mL/min. λ = 230 nm, t(major) = 23.94 min, t(minor) = 28.15 min, 94.9:5.1 er.

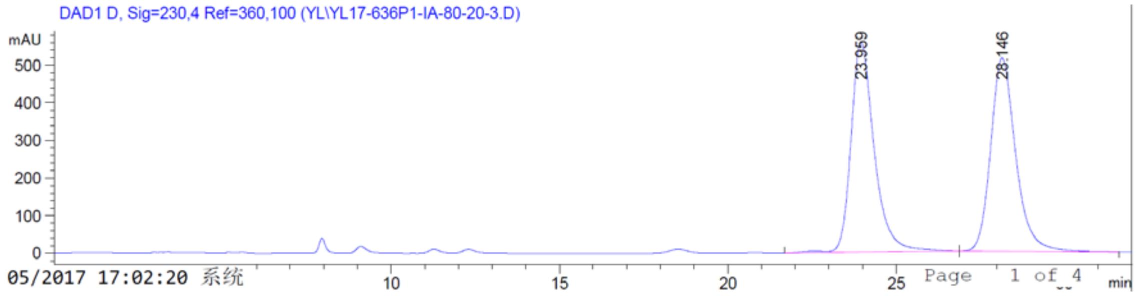

Signal 4: DAD1 D, Sig=230,4 Ref=360,100

Peak #	RetTime [min]	Type	Width [min]	Area [mAU*s]	Height [mAU]	Area %
1	23.959	BB	0.7236	2.69352e4	559.57062	50.3046
2	28.146	BBA	0.7843	2.66090e4	513.75757	49.6954

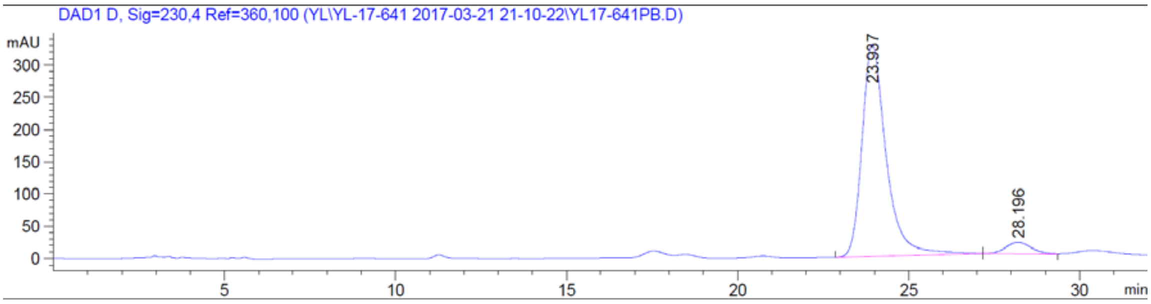

Signal 4: DAD1 D, Sig=230,4 Ref=360,100

Peak #	RetTime [min]	Type	Width [min]	Area [mAU*s]	Height [mAU]	Area %
1	23.937	BB	0.7335	1.61472e4	329.61832	94.8771
2	28.196	BB	0.7585	871.86316	17.52355	5.1229

Supplementary Figures 122. HPLC spectra for racemic and chiral **2ae**

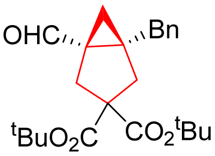

2ae, HPLC condition: Chiralcel AS-H, *i*-PrOH/*n*-hexane = 97/3, flow rate 0.6 mL/min. λ = 214 nm, t(major) = 11.07 min, t(minor) = 13.54 min, 91.6:8.4 er.

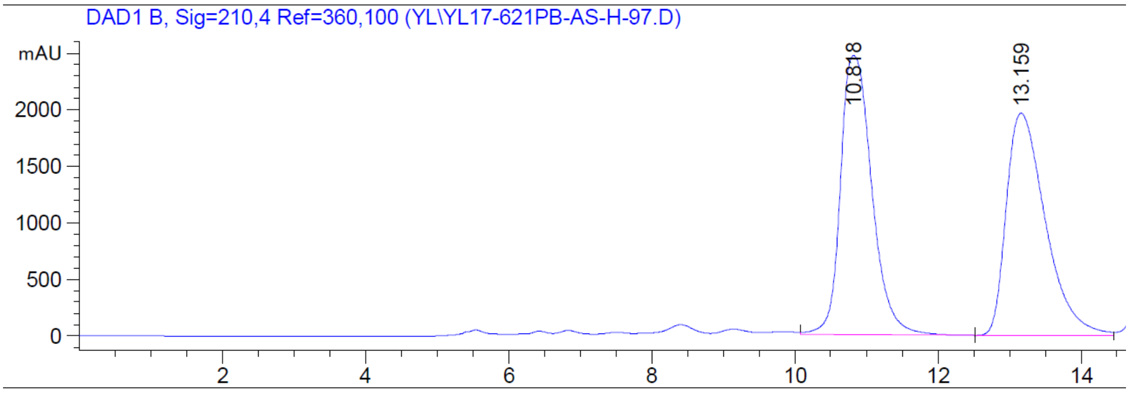

Signal 2: DAD1 B, Sig=210,4 Ref=360,100

Peak #	RetTime [min]	Type	Width [min]	Area [mAU*s]	Height [mAU]	Area %
1	10.818	VB	0.4693	7.51588e4	2468.10718	50.1180
2	13.159	BV	0.5876	7.48050e4	1967.29749	49.8820

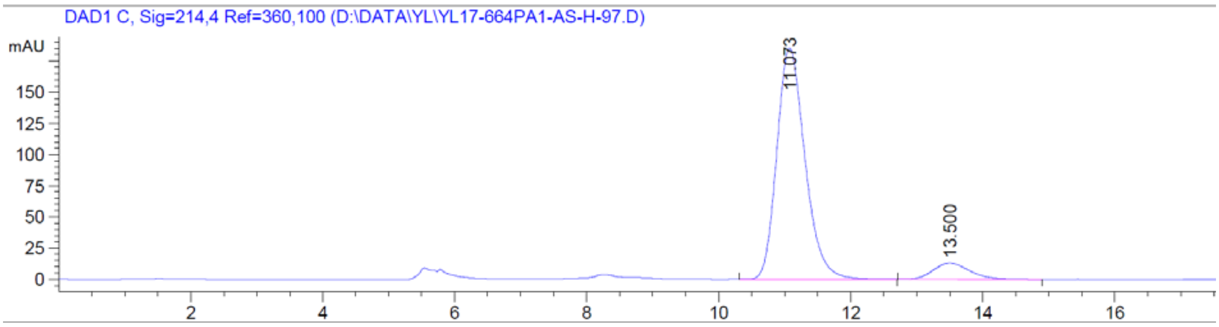

Signal 2: DAD1 C, Sig=214,4 Ref=360,100

Peak #	RetTime [min]	Type	Width [min]	Area [mAU*s]	Height [mAU]	Area %
1	11.073	BB	0.4580	5498.94482	185.40727	91.6019
2	13.500	BB	0.5801	504.14539	13.12451	8.3981

Supplementary Figures 123. HPLC spectra for racemic and chiral **2af**

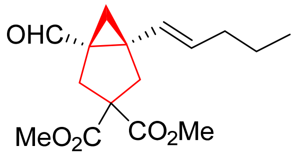

2af, HPLC condition: Chiralcel IA, *i*-PrOH/*n*-hexane = 95/5, flow rate 1.0 mL/min. λ = 254 nm, t(major) = 7.22 min, t(minor) = 7.98 min, 77.5:22.5 er.

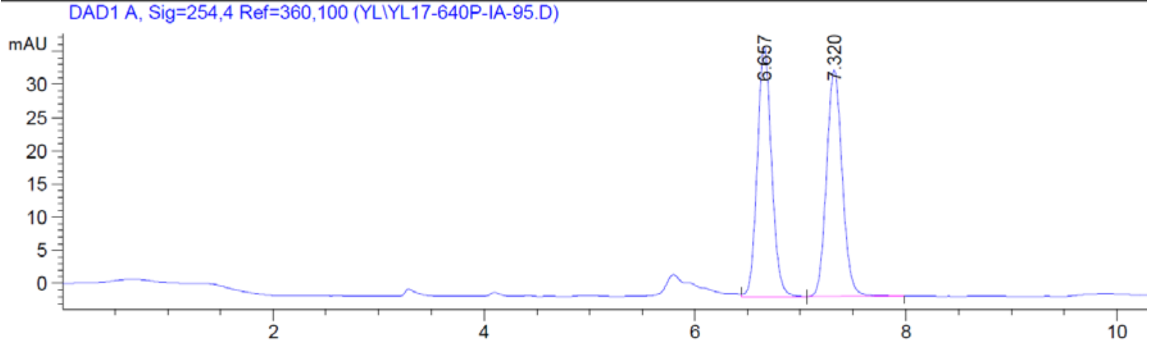

Signal 1: DAD1 A, Sig=254,4 Ref=360,100

Peak #	RetTime [min]	Type	Width [min]	Area [mAU*s]	Height [mAU]	Area %
1	6.657	VB	0.1457	354.33582	37.67252	49.9834
2	7.320	BB	0.1612	354.57169	34.12046	50.0166

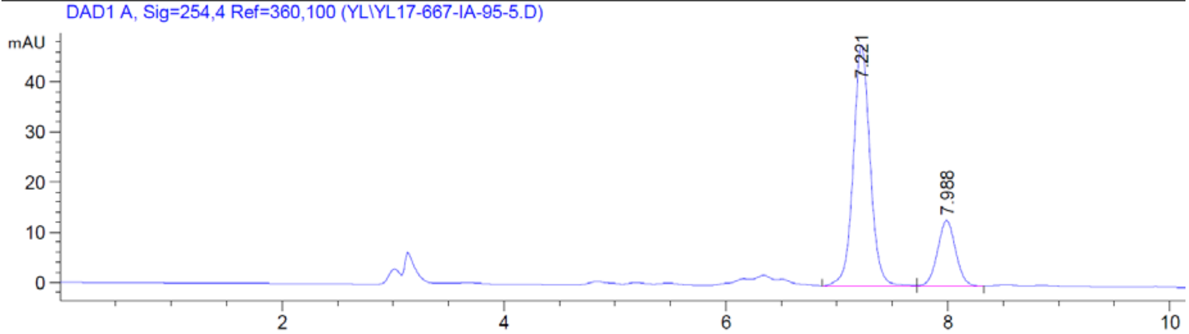

Signal 1: DAD1 A, Sig=254,4 Ref=360,100

Peak #	RetTime [min]	Type	Width [min]	Area [mAU*s]	Height [mAU]	Area %
1	7.221	BV	0.1586	505.10983	48.06079	77.4621
2	7.988	VB	0.1731	146.96349	13.07631	22.5379

Supplementary Figures 124. HPLC spectra for racemic and chiral **4a**

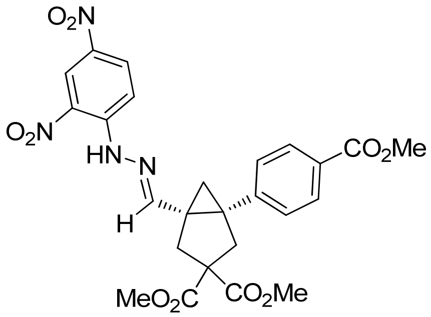

4a HPLC condition: Chiralcel AD-H, *i*-PrOH/*n*-hexane = 80/20, flow rate 1.0 mL/min. λ = 230 nm, t(minor) = 20.07 min, t(major) = 26.41 min, 95.2:4.8 er.

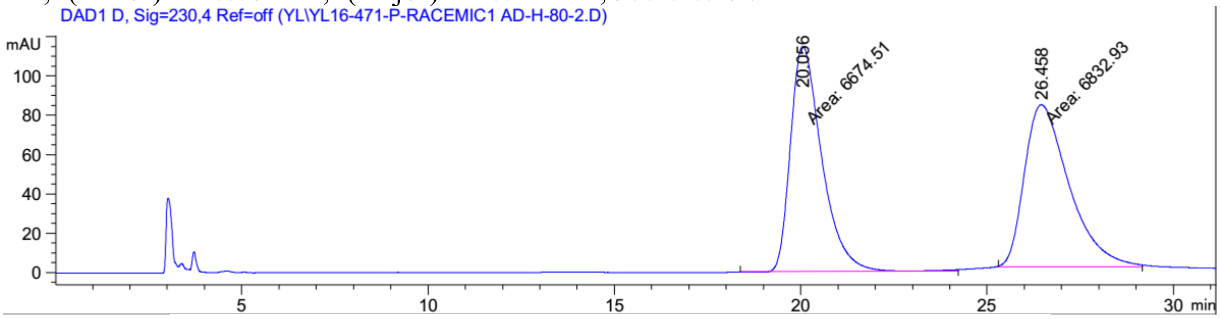

Signal 4: DAD1 D, Sig=230,4 Ref=off

Peak #	RetTime [min]	Type	Width [min]	Area [mAU*s]	Height [mAU]	Area %
1	20.056	MM	0.9725	6674.51123	114.38651	49.4136
2	26.458	MM	1.3834	6832.93115	82.31929	50.5864

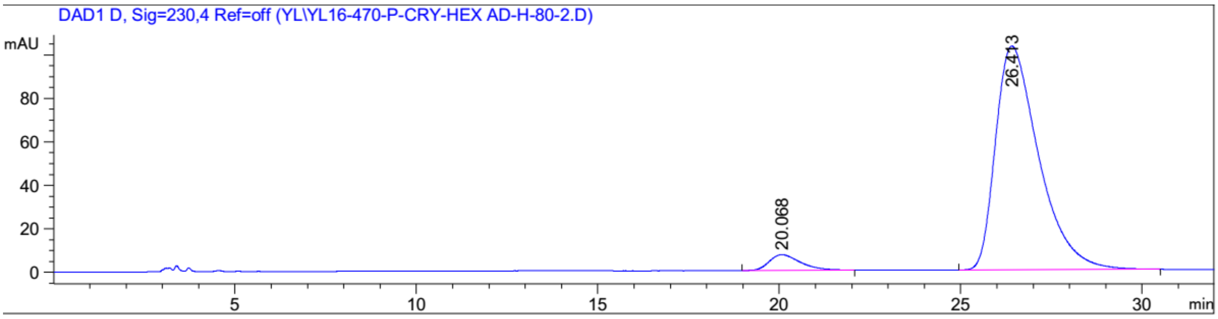

Signal 4: DAD1 D, Sig=230,4 Ref=off

Peak #	RetTime [min]	Type	Width [min]	Area [mAU*s]	Height [mAU]	Area %
1	20.068	BB	0.8320	428.77414	7.20753	4.7912
2	26.413	BB	1.2395	8520.42871	102.95412	95.2088

Supplementary Figures 125. HPLC spectra for racemic and chiral **5a**

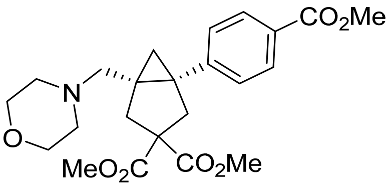

5a, Chiralcel OD-3, *i*-PrOH/*n*-hexane = 95/5, flow rate 0.6 mL/min. λ = 254 nm, t(minor) = 20.87 min, t(major) = 23.42 min, 94:6 er.

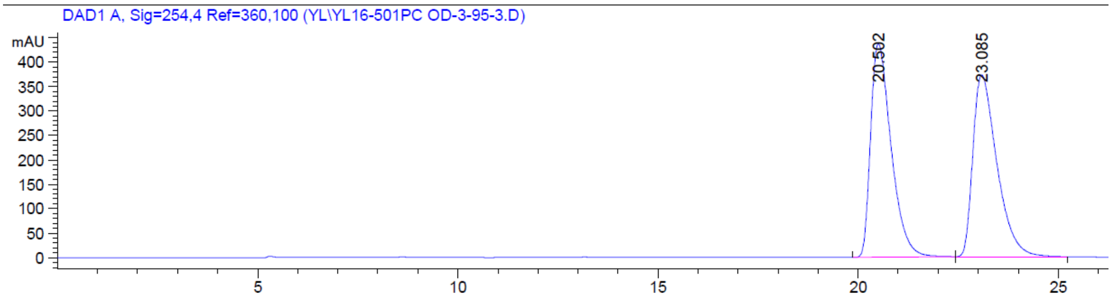

Signal 1: DAD1 A, Sig=254,4 Ref=360,100

Peak #	RetTime [min]	Type	Width [min]	Area [mAU*s]	Height [mAU]	Area %
1	20.502	BB	0.5458	1.55784e4	437.12106	50.1727
2	23.085	BB	0.6352	1.54712e4	371.98883	49.8273

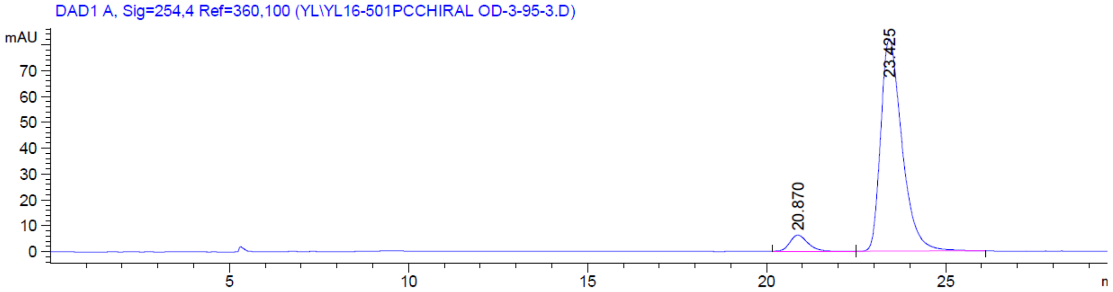

Signal 1: DAD1 A, Sig=254,4 Ref=360,100

Peak #	RetTime [min]	Type	Width [min]	Area [mAU*s]	Height [mAU]	Area %
1	20.870	BB	0.5285	212.21820	6.15144	5.9002
2	23.425	BB	0.6340	3384.58667	81.93044	94.0998

Supplementary Figures 126. HPLC spectra for racemic and chiral **6a**

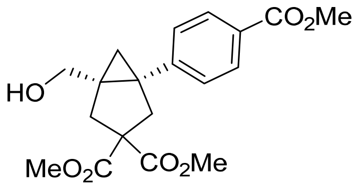

6a, Chiralcel AD-3, *i*-PrOH/*n*-hexane = 80/20, flow rate 0.8 mL/min. λ = 254 nm, t(minor) = 13.89 min, t(major) = 20.35 min, 94:6 er.

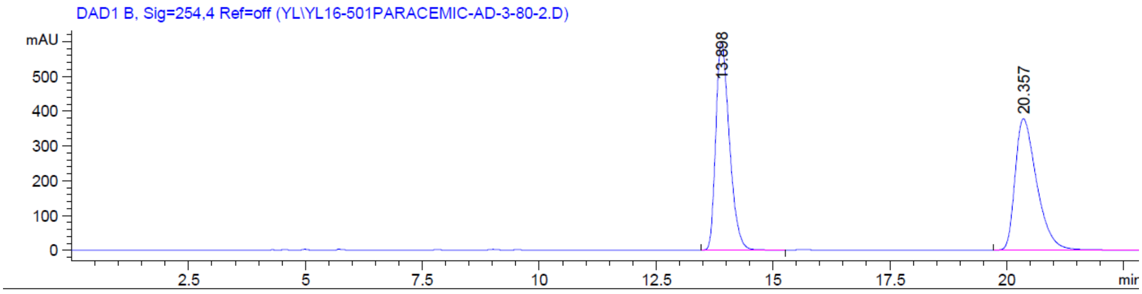

Signal 2: DAD1 B, Sig=254,4 Ref=off

Peak #	RetTime [min]	Type	Width [min]	Area [mAU*s]	Height [mAU]	Area %
1	13.898	BB	0.3098	1.21225e4	599.76166	49.9953
2	20.357	BBA	0.4905	1.21247e4	377.70642	50.0047

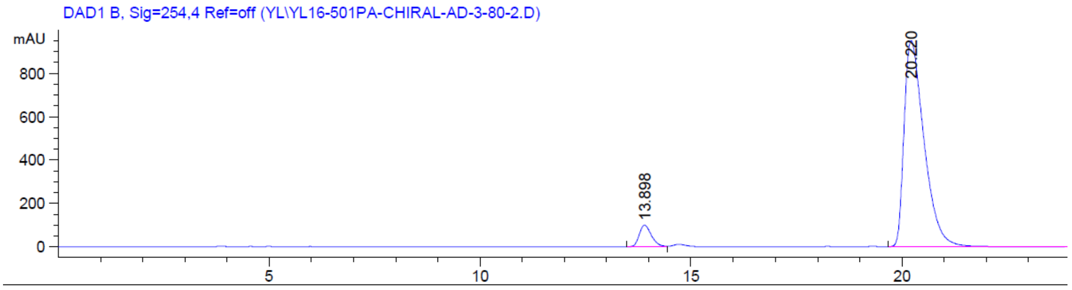

Peak #	RetTime [min]	Type	Width [min]	Area [mAU*s]	Height [mAU]	Area %
1	13.898	BV	0.3085	2003.59302	99.68087	6.0296
2	20.220	BB	0.5012	3.12259e4	950.64594	93.9704

Supplementary Figures 127. HPLC spectra for racemic and chiral **7a**

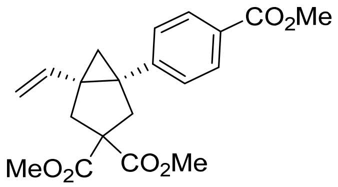

7a, Chiralcel OD-3, *i*-PrOH/*n*-hexane = 98/2, flow rate 0.3 mL/min. λ = 254 nm, t(major)= 33.99 min, t(minor) = 38.12 min, 94.5:5.5 er.

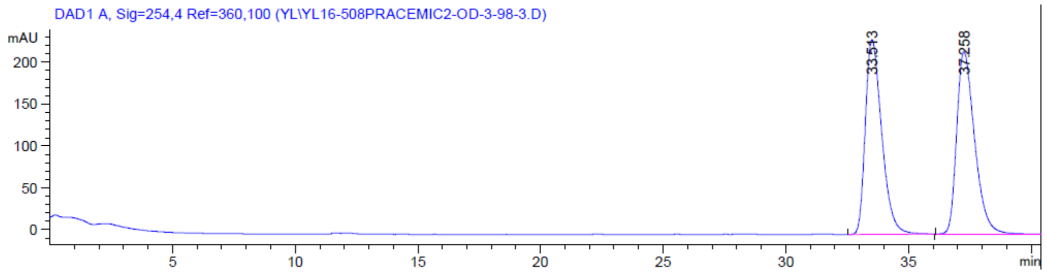

Signal 1: DAD1 A, Sig=254,4 Ref=360,100

Peak #	RetTime [min]	Type	Width [min]	Area [mAU*s]	Height [mAU]	Area %
1	33.513	BB	0.7162	1.07923e4	232.20825	49.3730
2	37.258	BBA	0.7853	1.10664e4	217.61761	50.6270

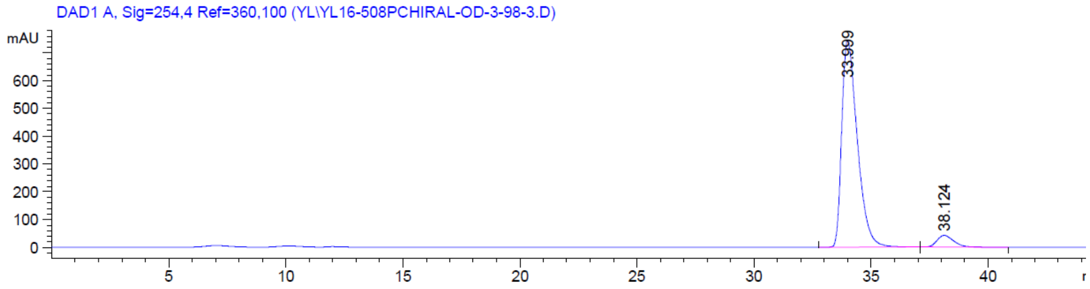

Signal 1: DAD1 A, Sig=254,4 Ref=360,100

Peak #	RetTime [min]	Type	Width [min]	Area [mAU*s]	Height [mAU]	Area %
1	33.999	BB	0.7167	3.44089e4	745.18292	94.2289
2	38.124	BB	0.7715	2107.38745	42.13735	5.7711

Supplementary Figures 128. HPLC spectra for racemic and chiral **8a**

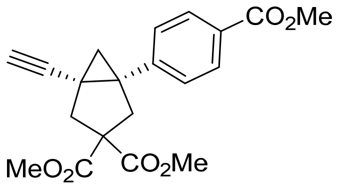

8a, Chiralcel AD-3, *i*-PrOH/*n*-hexane = 90/10, flow rate 0.8 mL/min. λ = 254 nm, t(minor) = 15.96 min, t(major) = 17.79 min, 94.5:5.5 er.

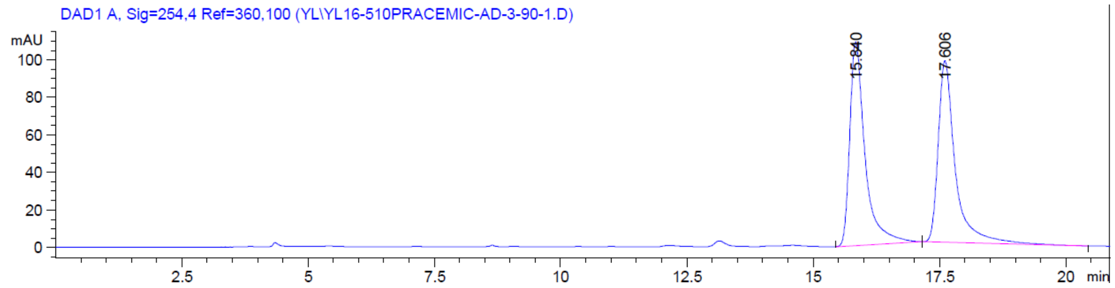

Signal 1: DAD1 A, Sig=254,4 Ref=360,100

Peak #	RetTime [min]	Type	Width [min]	Area [mAU*s]	Height [mAU]	Area %
1	15.840	BB	0.3120	2290.02539	108.62689	50.0688
2	17.606	BBA	0.3471	2283.73633	96.84109	49.9312

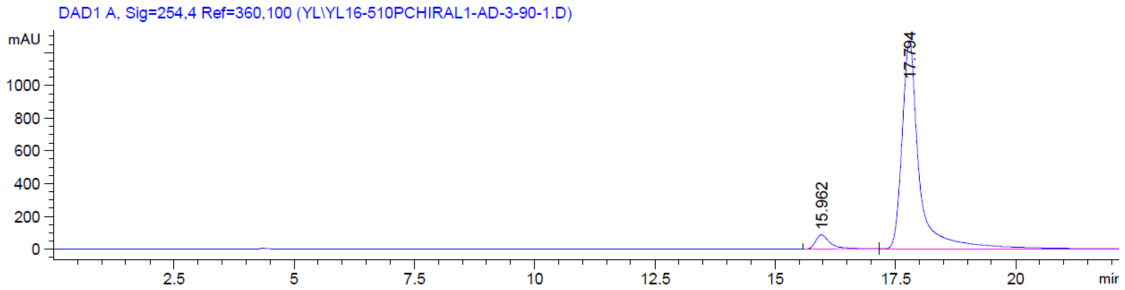

Signal 1: DAD1 A, Sig=254,4 Ref=360,100

Peak #	RetTime [min]	Type	Width [min]	Area [mAU*s]	Height [mAU]	Area %
1	15.962	BB	0.3157	1856.25769	87.41593	5.5127
2	17.794	BBA	0.3663	3.18159e4	1269.95276	94.4873

Supplementary Tables

Supplementary Table 1 Screening of effect of amine catalyst*

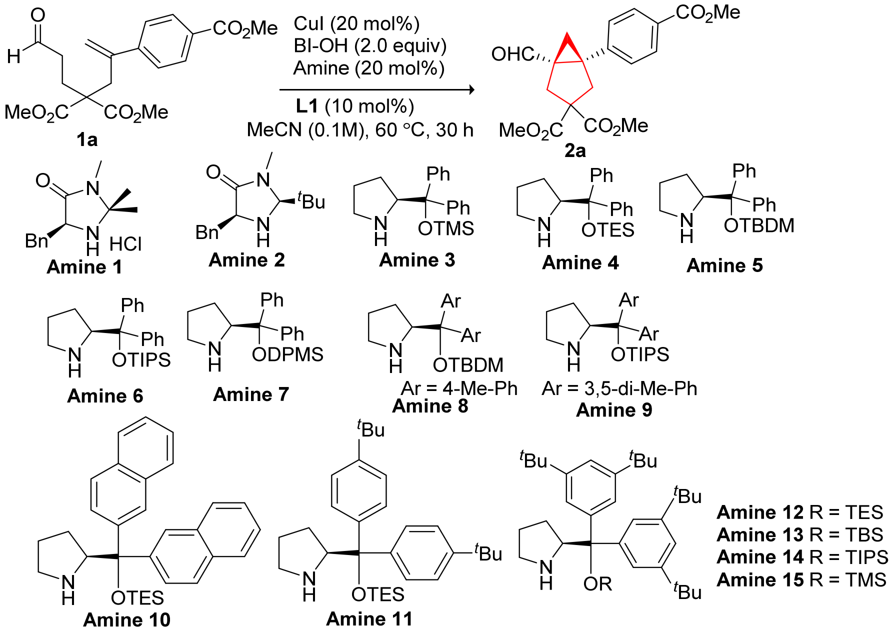

entry	Amine catalyst	conv. (%)	er
1	**Amine 1**	40	56:44
2	**Amine 2**	10	N.D.
3	**Amine 3**	60	69:31
4	**Amine 4**	80	70:30
5	**Amine 5**	90	66.5:33.5
6	**Amine 6**	90	77:23
7	**Amine 7**	90	66.5:33.5
8	**Amine 8**	95	70:30
9	**Amine 9**	95	73.5:26.5
10	**Amine 10**	90	76.5:23.5
11	**Amine 11**	90	57.5:42.5
**12**	**Amine 12**	**95**	**82.5:17.5**
13	**Amine 13**	95	81.5:18.5
14	**Amine 14**	95	70:30
15	**Amine 15**	90	52.5:47.5

* The reaction was run on 0.05 mmol scale.

Supplementary Table 2 Screening of effect of solvent*

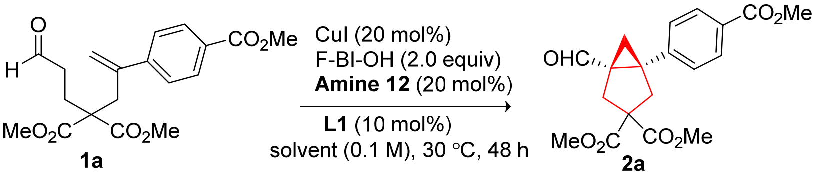

entry	solvent	conv. (%)	er
1	MeCN/DMF = 4/1	100	83.5:16.5
2	MeCN/MeOH = 4/1	80	68.5:31.5
3	MeCN/Dioxane = 4/1	100	86.5:13.5
4§	MeCN/Dioxane = 2/1	90	80.5:19.5
5	MeCN	90	88:12
6§	MeCN	90	89:11

* The reaction was run on 0.05 mmol scale. § Reaction concentration was 0.2 M.

Supplementary Table 3. Screening of effect of ligand*

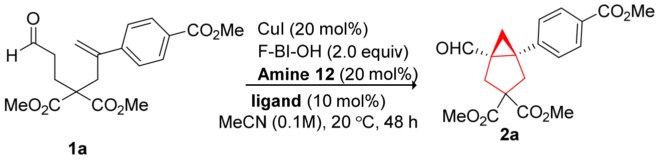

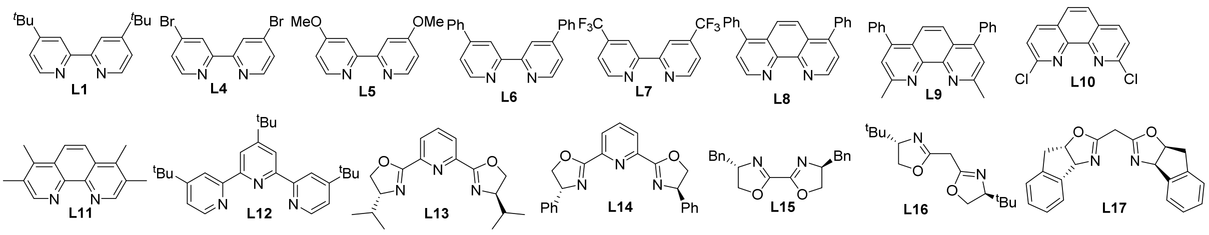

entry	ligand	conv. (%)	ee (%)
1	**L1**	**ca.85**	91.5:8.5
2	**L4**	100 (trace product)	N.D.
3	**L5**	100	85:15
4	**L6**	60	86.5:13.5
5	**L7**	100	90:10
5	**L8**	50	90:10
6	**L9**	60	N.D.
7	**L10**	100 (trace product)	N.D.
8	**L11**	100	86.5:13.5
9	**L12**	100	70:30
10	**L13**	100	90.5:9.5
11	**L14**	100	78.5:11.5
12	**L15**	100	91:9
13	**L16**	40	N.D.
14	**L17**	100	89.5:10.5

*The reaction was run on 0.05 mmol scale.

Supplementary Table 4. Screening of effect of temperature

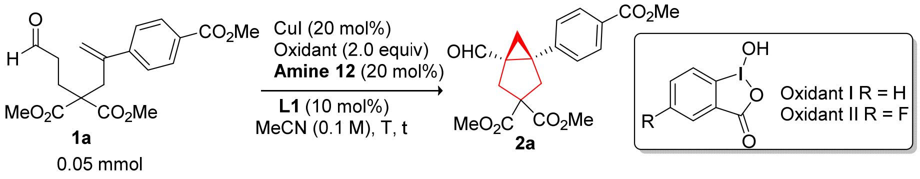

entry	oxidant	T (ºC)	t (h)	conv. (%)	er
1	I	60	30	95	82.5:17.5
2	I	50	34	95	84.5:15.5
3	I	40	46	90	84.5:15.5
4	I	30	48	80	85:15
5	I	20	72	50	88:12
6	II	30	48	100	88:12
**7**	**II**	**20**	**48**	**ca. 85**	**91.5:8.5**
8	II	5	96	ca. 60	92.5:7.5

Supplementary Table 5. Screening of effect of oxidant.

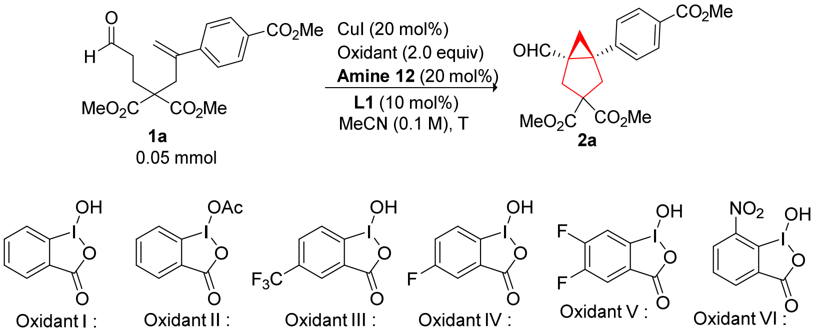

entry	oxidant	T (°C)	t (h)	conv. (%)	er
1	I	30	50	90	85:15
2	II	30	48	100	72.5:27.5
3	III	30	48	100	87:13
4	IV	30	48	100	88:12
5	IV (2.5 equiv)	30	48	100	90:10
6	IV (1.5 equiv)	30	48	90	86.5:13.5
**7**	**IV**	**20**	**48**	**90**	**91.5:8.5**
8	V	20	48	100 (65% yield)	90:10
**9**	**V**	**10**	**96**	**ca. 50**	**92.5:7.5**
10	VI	20	48	100	70:30

Supplementary Table 6. Screening of effect of additive*

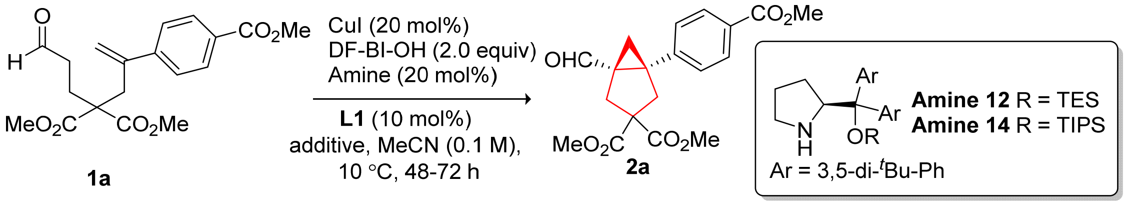

entry	amine	additive	yield (%)	er
1	**Amine 12**	$BnEt_3NCl$ (20 mol%)	67	92:8
2	**Amine 12**	$BnEt_3NBr$ (20 mol%)	-§	90:10
3	**Amine 12**	*n*-Bu_4NBr (20 mol%)	-§	85:15
**4**	**Amine 12**	***n*-Bu_4NI (20 mol%)**	**70**	**92.5:7.5**
5	**Amine 12**	*n*-Bu_4NCl (20 mol%)	66	**92.5:7.5**
6	**Amine 12**	4,5-difluoro-2-iodobenzoic acid (20 mol%)	60	88:12
7	**Amine 14**	$BnEt_3NCl$ (20 mol%)	-§	93:7
8	**Amine 14**	*n*-Bu_4NCl (20 mol%)	-§	90:10
9	**Amine 14**	*n*-Bu_4NBF_4 (20 mol%)	-§	94:6
10	**Amine 14**	*n*-Et_4NI (20 mol%)	-§	94.5:5.5
**11**	**Amine 14**	***n*-Bu_4NI (20 mol%)**	**60**	**95:5**
12¶	**Amine 14**	*n*-Bu_4NI (20 mol%)	-§	90:10
13	**Amine 14**	*n*-Bu_4NI (10 mol%)	-§	93.5:6.5

* The reaction was run on 0.05 mmol scale. §Yield was not detected. ¶ Reaction was run at 5 °C and reaction time was 4 days.

Supplementary Methods

All reactions were carried out under argon (Ar) atmosphere using Schlenk techniques with magnetic stirring. Reagents were purchased at the highest commercial quality and used without further purification, unless otherwise stated. Acetonitrile was purchased anhydrous from commercial sources, degassed before usage and transferred under an argon atmosphere. Analytical thin layer chromatography (TLC) was performed on precoated silica gel 60 F254 plates. Flash column chromatography was performed using Tsingdao silica gel (60, particle size 0.040-0.063 mm). Visualization on TLC was achieved by use of UV light (254 nm) or iodine. NMR spectra were recorded on a Bruker DPX 400 spectrometer at 400 MHz/500 MHz for ^{1}H NMR, 100 MHz /125 MHz for ^{13}C NMR and 376 MHz for ^{19}F NMR in $CDCl_3$ with tetramethylsilane (TMS) as internal standard. Chemical shifts are reported in ppm and coupling constants are given in Hz. Data for ^{1}H NMR are recorded as follows: chemical shift (ppm), multiplicity (s, singlet; d, doublet; t, triplet; q, quartet; m, multiplet), coupling constant (Hz), integration. Data for ^{13}C NMR are reported in terms of chemical shift (δ, ppm). ^{19}F NMR spectra were recorded on a Bruker DPX 400 MHz spectrometer. HMRS were obtained on a Bruker Apex IV RTMS.

Catalysts **A12-A14** were prepared from L-proline according to the reported procedures.[1,2]

Cyclic hypervalent iodine(III) oxidants (BI-OH, F-BI-OH and DF-BI-OH, *etc.*) were prepared according to the reported procedures.[3]

General procedure for the synthesis of substrate 1-2a

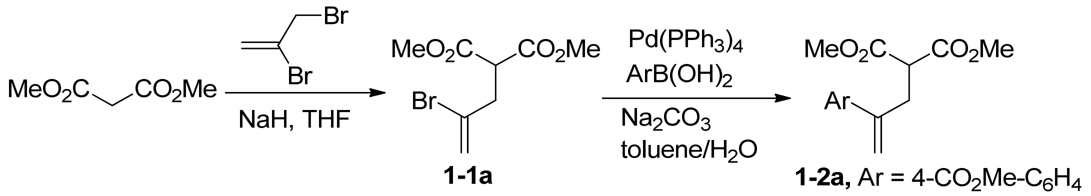

To a suspension of NaH (1.2 g, 30 mmol, 60% in mineral oil) in THF (40 mL) was added dimethyl malonate (46 mmol) in THF (5.0 mL) slowly in ice-bath over 30 min. The suspension was stirred for 30 min and a light-yellow solution was obtained. 2,3-Dibromoprop-1-ene (33 mmol) was then added and the reaction mixture was continued to stir at rt for 1.5 h. The reaction mixture was quenched by slow addition of water, and then extracted with ethyl acetate. The organic layer was washed with brine, dried with $MgSO_4$, filtered and concentrated. Flash chromatography (petroleum ether/ethyl acetate =20/1) gave the desired product **1-1a** (5.8 g) as a pale yellow oil in 70% yield.

To a flame-dried Schlenk tube equipped with a magnetic stir bar were added **1-1a** (2.5 g, 10 mmol), $Pd(PPh_3)_4$ (0.58 g, 5 mol%), Na_2CO_3 (4.24 g, 40 mmol) and (4-(methoxycarbonyl)phenyl)boronic acid (2.16 g, 12 mmol). The tube was evacuated and backfilled with argon for three times, freshly degassed toluene (15 mL) and water (15 mL) were added *via* syringe. The tube was stirred at 70 °C for 10 hours. After completion, solvent was removed under reduced pressure, and the residue was diluted with saturated water, then extracted with ethyl acetate. The organic layer was washed with brine, dried with $MgSO_4$, filtered and concentrated. Flash chromatography (petroleum ether/ethyl acetate =15/1-10/1) gave the corresponding product **1-2a** (2.45 g) as a yellow oil in 80% yield.

1-2b--1-2y (except for **1-2i** and **1-2t)**, **1-2aa**, **1-2ag**, **1-2al** and **1-2am** were prepared according to the similar procedure for **1-2a**.

1-2af was prepared according to reference.[4]

Synthesis of 1-2i and 1-2t.[5,6]

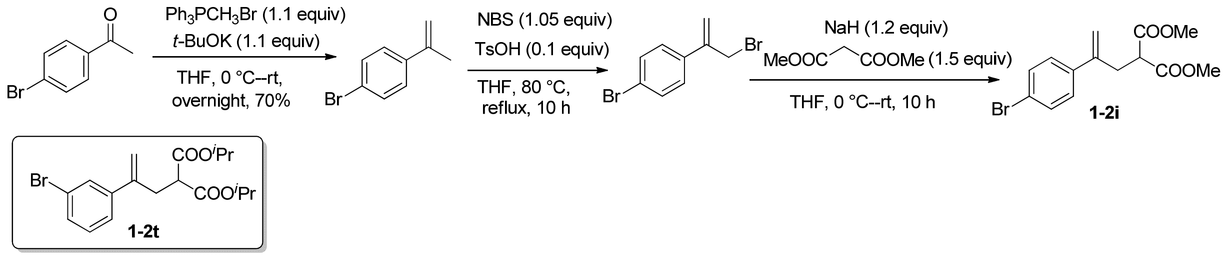

To a solution of Ph_3PCH_3Br (19.7 g, 55 mmol) in THF (60 mL) was added potassium-butoxide (6.2 g, 55 mmol) in ice-bath. The resulting mixture was stirred for 30 min. Then 4-bromoacetophenone (6.9 mL, 50 mmol) was added slowly. The resulting mixture was stirred at rt overnight until monitored full completion of starting material. After completion, solvent was

removed under reduced pressure, and the residue was diluted with ethyl acetate and washed with brine, dried with $MgSO_4$, filtered and concentrated. Flash chromatography (petroleum ether/ethyl acetate =20/1-15/1) gave the corresponding product 1-bromo-4-(prop-1-en-2-yl)benzene (6.87 g) as a yellow oil in 70% yield.

To a solution of 1-bromo-4-(prop-1-en-2-yl)benzene (3.94 g, 20 mmol) in THF (30 mL) was added NBS (3.74 g, 21 mmol) and TsOH (0.34 g, 2 mmol), and the mixture was heated to reflux for 4 h. After cooling the reaction mixture to room temperature, insoluble succinimide was removed by filtration. The filtrate was concentrated and purified by flash column chromatography on silica gel (petroleum ether) to afford the desired compound in 70% yield (ca. 90% purity).

1-2i was prepared in 65% yield from 1-bromo-4-(3-bromoprop-1-en-2-yl)benzene according to the similar procedure for the synthesis of **1-2a**.

1-2t was prepared from 3-bromoacetophenone according to the similar procedure for the synthesis of **1-2i**.

Synthesis of substrate 1-2z

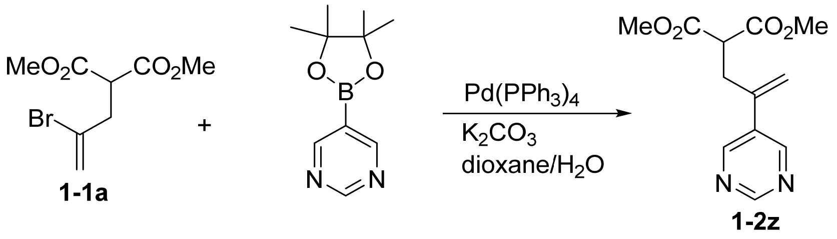

To a flame-dried Schlenk tube equipped with a magnetic stir bar were added **1-1a** (0.83 g, 3.3 mmol), $Pd(PPh_3)_4$ (0.38 g, 10 mol%), K_2CO_3 (1.14 g, 8.25 mmol) and (4-(methoxycarbonyl)phenyl)boronic acid (750 mg, 3.64 mmol). The tube was evacuated and backfilled with argon for three times, dioxane (15 mL) and water (1.5 mL) were added *via* syringe. The tube was stirred at 80 °C for 8 hours. After completion, solvent was removed under reduced pressure, and the residue was diluted with saturated water, then extracted with ethyl acetate. The organic layer was washed with brine, dried with $MgSO_4$, filtered and concentrated. Flash chromatography (petroleum ether/ethyl acetate =15/1-10/1) gave the corresponding product **1-2z** (0.62 g) as a yellow oil in 75% yield.

Synthesis of 1-2ad and 1-2ae.[7,8]

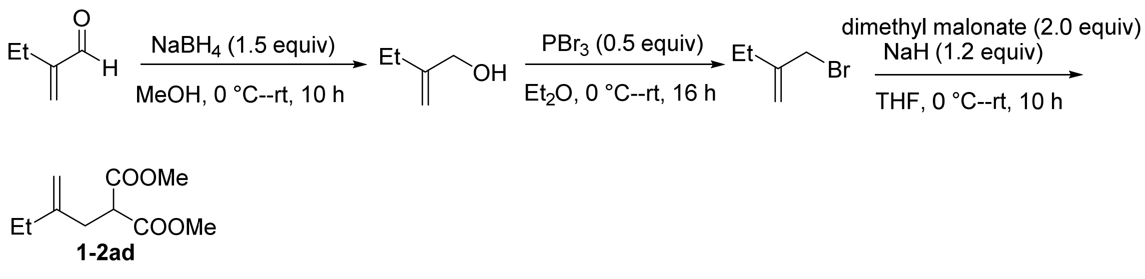

A solution of 2-methylenebutanal (2.45 mL, 25 mmol) in methanol (30 mL) was cooled to 0 ºC. Sodium borohydride (1.4 g, 37.5 mmol) was added portionwise, and the reaction mixture was stirred at 0 ºC for 1 h and then stirred at room temperature overnight until TLC showed the disappearance of starting material. The reaction mixture was evaporated to remove solvent and purified by flash column chromatography on silica gel (petroleum ether/ethyl acetate = 10/1-3/1) to give the substituted allyl alcohol (1.72 g) as a pale yellow oil in 80% yield.

To a solution of 2-methylenebutan-1-ol (1.72 g, 20 mmol) in diethyl ether (40 mL) was added phosphorus tribromide (0.94 mL, 10 mmol) dropwise at 0 ºC. Then the reaction mixture was stirred at room temperature for 16 h. The reaction mixture was cooled to 0 ºC and quenched with ice water. The organic layer was then washed sequentially with water, saturated sodium bicarbonate, and brine solution. Extracted with Et_2O, the combined organic layer was dried over sodium sulfate, filtered, and evaporated to 1/4 volume to yield substituted allyl bromide. This crude product was used for the next step without further purification.

1-2ad was prepared from 2-(bromomethyl)but-1-ene and dimethyl malonate following the previous method (52% yield for two steps).

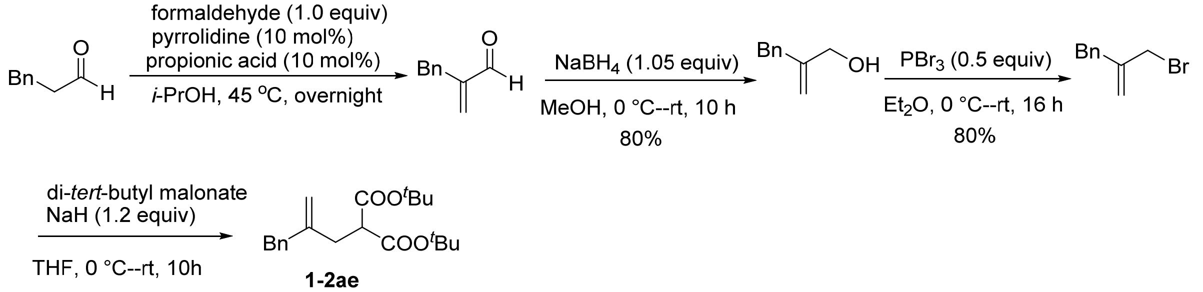

1-2ae

To a mixture of aqueous formaldehyde solution (37% formaldehyde in water, 30 mmol, 1.0 equiv) and 3-phenylpropanal (4.0 mL, 30 mmol) in *i*-PrOH (30 mL) were added propionic acid (0.23 mL, 3.0 mmol, 10 mol%) and pyrrolidine (0.25 mL, 3.0 mmol, 10 mol%). The reaction mixture was stirred at 45 °C overnight. The reaction mixture was quenched by the addition of saturated $NaHCO_3$ solution, and the mixture was then extracted with CH_2Cl_2 (3 × 20 mL). The combined extracts were washed with brine, dried over (Na_2SO_4), and concentrated in vacuum. The residue was purified by flash column chromatography on silica gel (petroleum ether/ethyl acetate = 20/1) to give the desired product (3.51 g) as a pale yellow oil in 80% yield.
The conversion of 2-benzylacrylaldehyde to **1-2ae** was followed by the similar procedure for the synthesis of **1-2ad**.

Representative Procedure for the synthesis of substrate 1.[9,10]

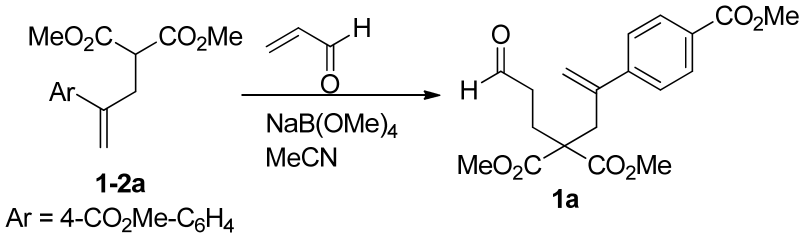

To a solution of **1-2a** (1.53 g, 5.0 mmol) and NaB(OMe)$_4$ (80 mg, 0.5 mmol, 10 mol%) in acetonitrile (10 mL) was added acrylaldehyde (6.0 mmol, 1.2 equiv) at room temperature. The resulting solution was stirred at room temperature under argon atmosphere and monitored by TLC. Upon completion, solvent was removed under reduced pressure, and the residue was purified by flash column chromatography on silica gel (petroleum ether/ethyl acetate = 10/1-3/1) to give the desired product **1a** (1.3 g) as a white solid in 72% yield.

Procedure for the Synthesis of 1ab and 1ac

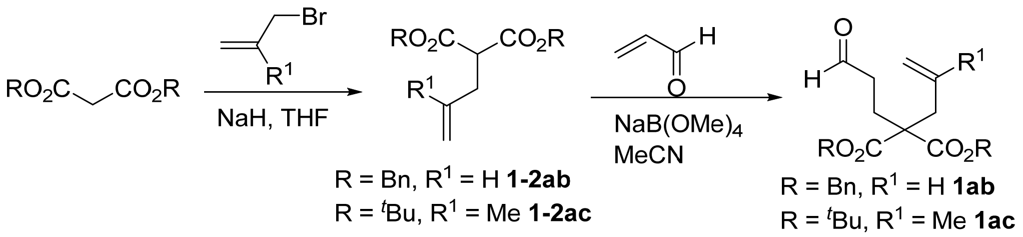

To a suspension of NaH (0.8 g, 20 mmol, 60% in mineral oil) in THF (30 mL) was added di-tert-butyl malonate (30 mmol) in THF (5 mL) slowly in ice-bath over 30 min. The suspension was stirred for 30 min and a light-yellow solution was obtained. 3-Bromo-2-methylprop-1-ene (2.2 mL, 22 mmol) was then added and the reaction mixture was continued to stir at rt for 1.5 h. The reaction mixture was quenched by slow addition of water, and then extracted with ethyl acetate. The organic layer was washed with brine, dried with $MgSO_4$, filtered and concentrated. Flash chromatography (petroleum ether/ ethyl acetate =20/1) gave the desired product **1-2ac** (4.08 g) as a pale yellow oil in 75% yield.

To a solution of **1-2ac** (2.6 g, 8.0 mmol) and NaB(OMe)$_4$ (64 mg, 0.4 mmol, 5 mol%) in acetonitrile (15 mL) was added acrylaldehyde (9.6 mmol, 1.2 equiv) at room temperature. The resulting solution was stirred at room temperature under argon atmosphere and monitored by TLC. Upon completion (ca. 5 h), solvent was removed under reduced pressure, and the residue was purified by flash column chromatography on silica gel (petroleum ether / ethyl acetate = 10/1-5/1) to give the desired product **1ac** (1.56 g) as a colorless oil in 60% yield.

1ab and **1aj** were prepared according to the similar procedure for the synthesis of **1ac**.

Procedure for the Synthesis of 1ah.[11]

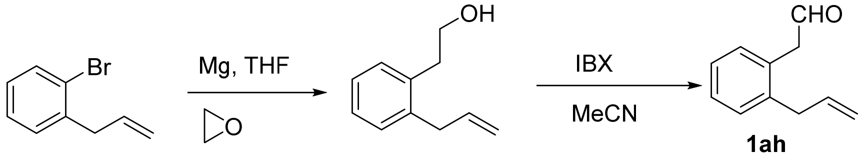

A dry round bottom flask was charged with a stir bar and Mg (0.27 g, 1.2 equiv) under an argon atmosphere. Part of the solution of 1-allyl-2-bromobenzene (1.77 g, 9 mmol) in THF (5.0 mL) was added to the flask and stirred. After the color of the mixture suddenly faded, the rest solution was added dropwise *via* dropping funnel. Then the mixture was stirred at reflux for 1 h. After that, oxirane (9 mL, 27 mmol, 2.5-3.3 M in THF 3 mL) was added dropwise in ice bath. The reaction was allowed to stir at rt for 1 h, and then quenched with NH_4Cl. After extracted with ethyl acetate, dried over Na_2SO_4 and concentrated, the crude product was applied to flash column chromatography to afford the product (1.31 g, 90% yield).

To a solution of 2-(2-allylphenyl)ethanol (0.77 g, 4.8 mmol) in MeCN (25 mL) was added IBX (4.2 g, 15 mmol). The resulting mixture was stirred at 80 °C for 2 h. The reaction mixture was filtered through a pad of celite and the filtrate was evaporated. The residue was purified by column chromatography on silica gel (petroleum ether / ethyl acetate = 10/1) to afford the **1ah** (0.61 g) as a colorless oil in 80% yield.

Synthesis of 1ai.[12]

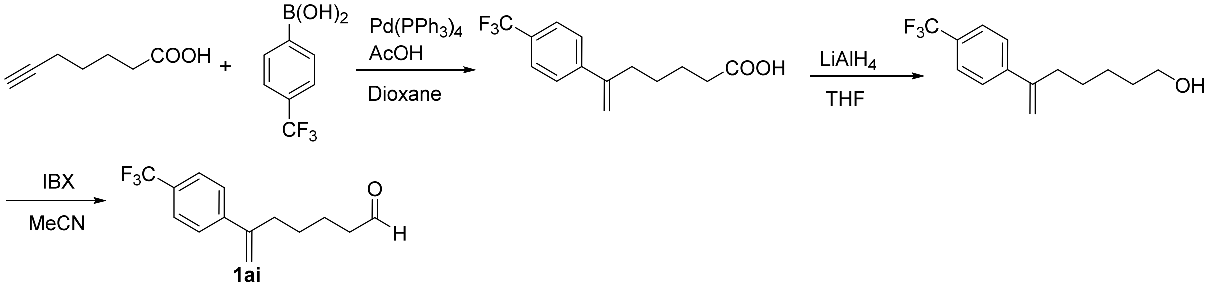

To a solution of 6-heptynoic acid (2.53 mL, 20 mmol) in dioxane (60 mL) were added $Pd(PPh_3)_4$ (0.7 g, 3 mol%), (4-(trifluoromethyl)phenyl)boronic acid (4.56g, 24 mmol) and AcOH (114 μL, 10 mol%) under an argon atmosphere. The mixture was stirred for 15 min at rt and then at 80 °C for 15 h. The reaction mixture was concentrated in vacuum and the crude product purified by column chromatography on silica gel (petroleum ether/ethyl acetate = 10/1-8/1) to afford the desired product as a white solid (3.44 g) in 63% yield.

To a solution of 6-(4-(trifluoromethyl)phenyl)hept-6-enoic acid (3.44 g, 12.6 mmol, 1 equiv) in THF (25 mL) was added $LiAlH_4$ (960 mg, 25.2 mmol, 2 equiv) at 0 °C. The mixture was stirred for 1 h at 0 °C then an aqueous solution of NaOH 3 M was added. The reaction mixture was filtered through a pad of celite and the aqueous layer was extracted with Et_2O. The organic extracts were dried over $MgSO_4$, filtered and concentrated in vacuum and the crude product was

purified by column chromatography on silica gel (petroleum ether/ethyl acetate = 6/1) to afford the alcohol product (2.08 g) as a colorless oil in 64% yield.

To a solution of 6-(4-(trifluoromethyl)phenyl)hept-6-en-1-ol (0.86 g, 3.33 mmol) in MeCN (15 mL) was added IBX (1.87 g, 6.66 mmol). The resulting mixture was stirred at 80 °C for 2 h. The reaction mixture was filtered through a pad of celite and the filtrate was evaporated. The residue was purified by column chromatography on silica gel (petroleum ether/ethyl acetate = 8/1) to afford the **1ai** (0.68 g) as a colorless oil in 80% yield.

Procedure for the Synthesis of 1ak[13]

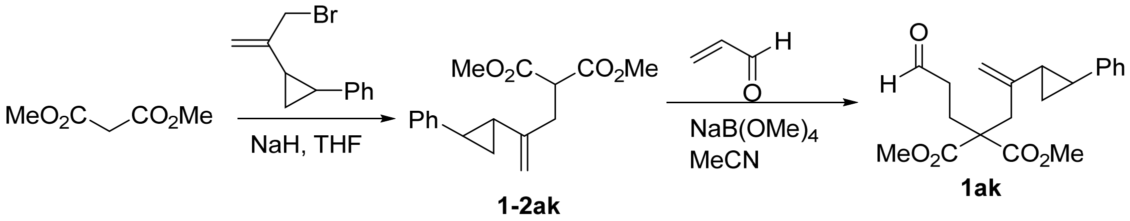

To a suspension of NaH (108 mg, 2.68 mmol, 60% in mineral oil) in THF (5.0 mL) was added di-methyl malonate (355 mg, 2.68 mmol) in THF (2 mL) slowly in ice-bath over 5 min. The suspension was stirred for 30 min and a light-yellow solution was obtained. (2-(3-bromoprop-1-en-2-yl)cyclopropyl)benzene (530 mg, 2.23 mmol) (which was prepared according to precedent) was then added and the reaction mixture was continued to stir at rt for 3 h. The reaction mixture was quenched by slow addition of water, and then extracted with ethyl acetate. The organic layer was washed with brine, dried with $MgSO_4$, filtered and concentrated. Flash chromatography (petroleum ether/ ethyl acetate =20/1) gave the desired product **1-2ak** (4.08 g) as a pale yellow oil in 65% yield. **^{1}H NMR** (400 MHz, $CDCl_3$) δ 7.30 – 7.24 (m, 2H), 7.19 – 7.13 (m, 1H), 7.11 – 7.07 (m, 2H), 4.78 (t, *J* = 1.2 Hz, 1H), 4.76 (q, *J* = 1.2 Hz, 1H), 3.72 (s, 3H), 3.69 (t, *J* = 7.6 Hz, 1H), 3.68 (s, 3H), 2.73 (m, dd, *J* = 8.4, 1.2 Hz, 2H), 1.90 (ddd, *J* = 8.8, 5.6, 4.8 Hz, 1H), 1.56 – 1.51(m, 1H), 1.23 (ddd, *J* = 8.8, 6.1, 5.0 Hz, 1H), 1.13 (ddd, *J* = 8.6, 5.8, 5.0 Hz, 1H). **^{13}C NMR** (100 MHz, $CDCl_3$) δ 169.43, 169.41, 145.65, 142.39, 128.41, 125.75, 125.70, 109.29, 52.58, 52.55, 50.63, 35.73, 28.10, 25.62, 15.35.

To a solution of **1-2ak** (288 g, 1.0 mmol) and $NaB(OMe)_4$ (16 mg, 0.1 mmol, 10 mol%) in acetonitrile (3.0 mL) was added acrylaldehyde (1.2 mmol, 1.2 equiv) at room temperature. The resulting solution was stirred at room temperature under argon atmosphere overnight. Upon completion, solvent was removed under reduced pressure, and the residue was purified by flash column chromatography on silica gel (petroleum ether / ethyl acetate = 10/1-5/1) to give the desired product **1ak** (190 mg) as a colorless oil in 56% yield.

Procedure for the Synthesis of 1am

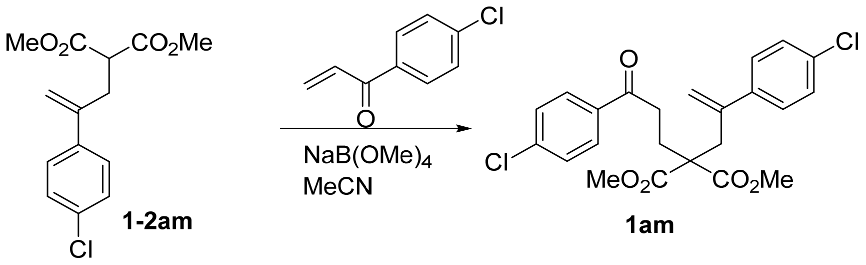

To a solution of **1-2am** (1.41 g, 5.0 mmol) and $NaB(OMe)_4$ (40 mg, 0.25 mmol, 5 mol%) in acetonitrile (15.0 mL) was added 1-(4-chlorophenyl)prop-2-en-1-one (6 mmol, 1.2 equiv) in acetonitrile (3.0 mL) in ice bath, and the resultant reaction mixture was stirred at room temperature upon completion, solvent was removed under reduced pressure, and the residue was purified by flash column chromatography on silica gel (petroleum ether / ethyl acetate = 10/1-5/1) to give the desired product **1am** (1.35 g) as a colorless oil in 60% yield.

General procedure for the preparation of cyclic hypervalent iodine(III) oxidant.

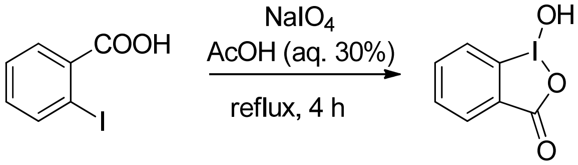

The mixture of $NaIO_4$ (7.24 g, 33.8 mmol, 1.05 equiv) and 2-iodobenzoic acid (8.00 g, 32.2 mmol, 1.00 equiv) were suspended in 30% (v/v) aq. AcOH (45 mL) and vigorously stirred under reflux for 4 h. The reaction mixture was allowed to cool to rt and then diluted with cold water (160 mL) and protecting it from light. After 30 min, the crude product was collected by filtration, washed on the filter with ice water (3 × 20 mL) and acetone (3 × 20 mL), and air-dried in the dark to give the pure product BI-OH (8.2 g, 31 mmol, 97%) as a colorless solid.

F-BI-OH and DF-BI-OH were prepared according to the similar procedure.

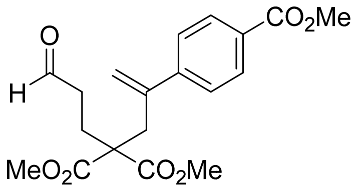

Dimethyl 2-(2-(4-(methoxycarbonyl)phenyl)allyl)-2-(3-oxopropyl)malonate (1a)
^{1}H NMR (400 MHz, $CDCl_3$) δ 9.59 (s, 1H), 7.96 – 7.94 (m, 2H), 7.39 – 7.32 (m, 2H), 5.33 (d, *J* = 1.1 Hz, 1H), 5.20 (s, 1H), 3.89 (s, 3H), 3.44 (s, 6H), 3.19 (s, 2H), 2.37 – 2.33 (m, 2H), 2.14 – 2.06 (m, 2H). **^{13}C NMR** (100 MHz, $CDCl_3$) δ 200.35, 170.78, 166.66, 145.88, 143.39, 129.48, 129.27, 126.83, 120.43, 56.38, 52.33, 52.10, 39.01, 38.41, 24.57. **HRMS** (ESI) m/z calcd. for $C_{19}H_{22}O_7Na$ $[M+Na]^+$ 385.1252, found 385.1257.

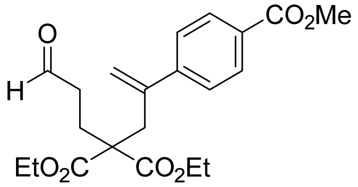

Diethyl 2-(2-(4-(methoxycarbonyl)phenyl)allyl)-2-(3-oxopropyl)malonate (1b)
^{1}H NMR (500 MHz, $CDCl_3$) δ 9.42 (s, 1H), 7.79 (d, *J* = 8.4 Hz, 2H), 7.22 (d, *J* = 8.4 Hz, 2H), 5.17 (s, 1H), 5.07 (s, 1H), 3.78 (m, 2H), 3.74 – 3.65 (m, 5H), 3.04 (s, 2H), 2.20 (t, *J* = 7.5 Hz, 2H), 1.92 (t, *J* = 7.5 Hz, 2H), 0.99 (t, *J* = 7.2 Hz, 6H). **^{13}C NMR** (125 MHz, $CDCl_3$) δ 200.16, 170.16, 166.35, 145.94, 143.49, 129.23, 129.09, 126.69, 120.05, 61.11, 56.13, 51.82, 38.71, 37.76, 24.11, 13.63. **HRMS** (ESI) m/z calcd. for $C_{21}H_{26}O_7Na$ $[M+Na]^+$ 413.1564, found 413.1571.

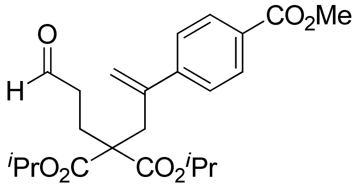

Diisopropyl 2-(2-(4-(methoxycarbonyl)phenyl)allyl)-2-(3-oxopropyl)malonate (1c)
^{1}H NMR (500 MHz, $CDCl_3$) δ 9.53 (s, 1H), 7.96 (d, *J* = 8.4 Hz, 2H), 7.38 (d, *J* = 8.4 Hz, 2H), 5.32 (d, *J* = 1.0 Hz, 1H), 5.20 (s, 1H), 4.80 (m, 2H), 3.90 (s, 3H), 3.17 (s, 2H), 2.29–2.26 (m, 2H), 2.06 – 2.01 (m, 2H), 1.16 (dd, *J* = 6.5, 3.6 Hz, 12H). **^{13}C NMR** (125 MHz, $CDCl_3$) δ 200.41, 169.99, 166.68, 146.50, 143.70, 129.53, 129.26, 126.75, 120.00, 69.27, 56.59, 52.08, 38.85, 37.52, 24.11, 21.46, 21.43. **HRMS** (ESI) m/z calcd. for $C_{23}H_{30}O_7Na$ $[M+Na]^+$ 441.1877, found 441.1884.

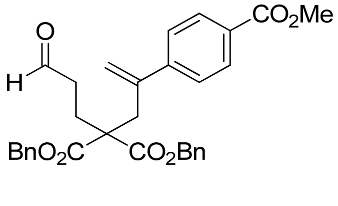

Dibenzyl 2-(2-(4-(methoxycarbonyl)phenyl)allyl)-2-(3-oxopropyl)malonate (1d)
^{1}H NMR (500 MHz, CDCl3) δ 9.49 (s, 1H), 7.99 – 7.93 (m, 2H), 7.38 – 7.34 (m, 2H), 7.33 – 7.30 (m, 6H), 7.22 – 7.17 (m, 4H), 5.31 (d, *J* = 1.1 Hz, 1H), 5.17 (s, 1H), 4.94 (d, *J* = 12.2 Hz, 2H), 4.77 (d, *J* = 12.2 Hz, 2H), 3.93 (s, 3H), 3.25 (s, 2H), 2.26 (m, 2H), 2.16 – 2.12 (m, 2H). **^{13}C NMR** (125 MHz, CDCl3) δ 200.23, 170.12, 166.69, 146.02, 143.29, 134.98, 129.58, 129.33, 128.57, 128.45, 128.30, 126.86, 120.50, 67.19, 56.64, 52.16, 38.84, 38.18, 24.47. **HRMS** (ESI) m/z calcd. for $C_{31}H_{30}O_7Na$ $[M+Na]^+$ 537.1878, found 537.1884.

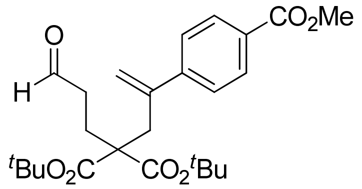

Di-tert-butyl 2-(2-(4-(methoxycarbonyl)phenyl)allyl)-2-(3-oxopropyl)malonate (1e)
^{1}H NMR (400 MHz, CDCl3) δ 9.46 (t, *J* = 1.4 Hz, 1H), 7.96 (d, *J* = 8.4 Hz, 2H), 7.38 (d, *J* = 8.4 Hz, 2H), 5.32 (d, *J* = 1.1 Hz, 1H), 5.21 (s, 1H), 3.90 (s, 3H), 3.10 (s, 2H), 2.26 – 2.15 (m, 2H), 1.99 – 1.90 (m, 2H), 1.37 (s, 18H). **^{13}C NMR** (100 MHz, CDCl3) δ 200.60, 169.75, 166.72, 147.07, 143.78, 129.69, 129.22, 126.69, 119.92, 81.99, 57.88, 52.09, 38.92, 36.96, 27.77, 24.13. **HRMS** (ESI) m/z calcd. for $C_{25}H_{34}O_7Na$ $[M+Na]^+$ 469.2193, found 469.2197.

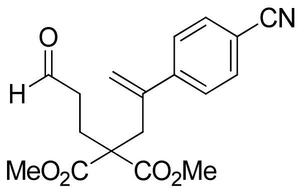

Dimethyl 2-(2-(4-cyanophenyl)allyl)-2-(3-oxopropyl)malonate (1f)
^{1}H NMR (400 MHz, CDCl3) δ 9.64 (s, 1H), 7.60 (d, *J* = 8.4 Hz, 2H), 7.40 (d, *J* = 8.4 Hz, 2H), 5.35 (s, 1H), 5.27 (s, 1H), 3.47 (s, 6H), 3.18 (s, 2H), 2.39 (t, *J* = 7.6 Hz, 2H), 2.12 – 2.06 (m, 2H). **^{13}C NMR** (100 MHz, CDCl3) δ 200.17, 170.67, 145.95, 142.79, 131.99, 127.57, 121.41, 118.58, 111.32, 56.30, 52.37, 39.03, 38.41, 24.70. **HRMS** (ESI) m/z calcd. for $C_{18}H_{19}NO_5Na$ $[M+Na]^+$ 352.1150, found 352.1155.

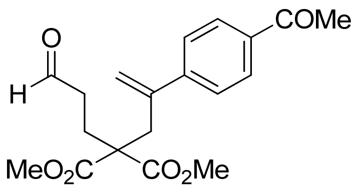

Dimethyl 2-(2-(4-acetylphenyl)allyl)-2-(3-oxopropyl)malonate (1g)
^{1}H NMR (400 MHz, CDCl3) δ 9.55 (s, 1H), 7.84 (d, *J* = 8.3 Hz, 2H), 7.34 (d, *J* = 8.3 Hz, 2H), 5.30 (s, 1H), 5.18 (s, 1H), 3.41 (s, 6H), 3.15 (s, 2H), 2.53 (s, 3H), 2.32 (t, *J* = 7.7 Hz, 2H), 2.05 (dd, *J* = 8.1, 7.2 Hz, 2H). **[3]C NMR** (100 MHz, CDCl3) δ 200.31, 197.47, 170.80, 146.09, 143.36, 136.25, 128.29, 127.02, 120.53, 56.48, 52.35, 39.06, 38.46, 26.59, 24.66.
HRMS (ESI) m/z calcd. for $C_{19}H_{22}O_6Na$ $[M+Na]^+$ 369.1305, found 369.1309.

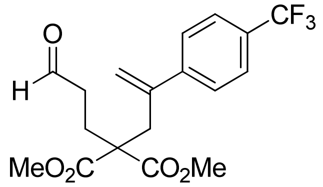

Dimethyl 2-(3-oxopropyl)-2-(2-(4-(trifluoromethyl)phenyl)allyl)malonate (1h)
^{1}H NMR (500 MHz, $CDCl_3$) δ 9.65 (t, J = 1.1 Hz, 1H), 7.57 (d, J = 8.1 Hz, 2H), 7.41 (d, J = 8.1 Hz, 2H), 5.34 (d, J = 1.0 Hz, 1H), 5.24 (d, J = 1.0 Hz, 1H), 3.46 (s, 6H), 3.21 (s, 2H), 2.42 – 2.37 (m, 2H), 2.13 (m, 2H). **^{13}C NMR** (125 MHz, $CDCl_3$) δ 200.36, 170.79, 144.90, 143.06, 129.71 (q, $J_{C\text{-}F}$ = 32.3 Hz, 1C), 127.26, 125.11 (q, $J_{C\text{-}F}$ = 3.6 Hz, 1C), 124.04 (q, $J_{C\text{-}F}$ = 270.4 Hz, 1C), 120.64, 56.28, 52.33, 39.05, 38.61, 24.61. **^{19}F NMR** (376 MHz, $CDCl_3$) δ -62.61.
HRMS (ESI) m/z calcd. for $C_{18}H_{19}F_3O_5Na$ $[M+Na]^+$ 395.1068, found 395.1076.

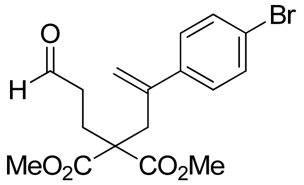

Dimethyl 2-(2-(4-bromophenyl)allyl)-2-(3-oxopropyl)malonate (1i)
^{1}H NMR (400 MHz, $CDCl_3$) δ 9.61 (s, 1H), 7.47 – 7.37 (m, 2H), 7.20 – 7.10 (m, 2H), 5.25 (s, 1H), 5.13 (s, 1H), 3.46 (s, 6H), 3.14 (s, 2H), 2.36 (t, J = 7.6 Hz, 2H), 2.10 (t, J = 7.6 Hz, 2H).
^{13}C NMR (100 MHz, $CDCl_3$) δ 200.43, 170.83, 143.07, 140.17, 131.24, 128.53, 121.63, 119.44, 56.34, 52.36, 39.03, 38.49, 24.54.
HRMS (ESI) m/z calcd. for $C_{17}H_{19}BrO_5Na$ $[M+Na]^+$ 405.0302, found 405.0308.

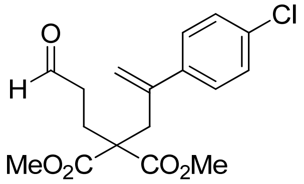

Dimethyl 2-(2-(4-chlorophenyl)allyl)-2-(3-oxopropyl)malonate (1j)
^{1}H NMR (400 MHz, $CDCl_3$) δ 9.61 (t, J = 1.1 Hz, 1H), 7.29 – 7.19 (m, 4H), 5.24 (d, J = 1.3 Hz, 1H), 5.13 (d, J = 0.8 Hz, 1H), 3.47 (s, 6H), 3.14 (s, 2H), 2.39 – 2.32 (m, 2H), 2.13 – 2.05 (m, 2H).
^{13}C NMR (100 MHz, $CDCl_3$) δ 200.44, 170.84, 143.02, 139.70, 133.48, 128.28, 128.19, 119.39, 56.32, 52.35, 39.02, 38.51, 24.51.
HRMS (ESI) m/z calcd. for $C_{17}H_{19}ClO_5Na$ $[M+Na]^+$ 361.0808, found 361.0813.

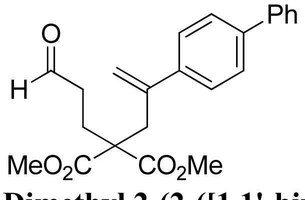

Dimethyl 2-(2-([1,1'-biphenyl]-4-yl)allyl)-2-(3-oxopropyl)malonate (1k)

^{1}**H NMR** (500 MHz, CDCl3) δ 9.61 (t, *J* = 1.1 Hz, 1H), 7.63 – 7.59 (m, 2H), 7.57 (d, *J* = 8.4 Hz, 2H), 7.45 (t, *J* = 8.0 Hz, 2H), 7.40 (d, *J* = 8.4 Hz, 2H), 7.37 – 7.34 (m, 1H), 5.35 (d, *J* = 1.5 Hz, 1H), 5.18 (s, 1H), 3.50 (s, 6H), 3.25 (s, 2H), 2.40 (t, *J* = 8.0 Hz, 2H), 2.24 (t, *J* = 8.0 Hz, 2H).
^{13}**C NMR** (125 MHz, CDCl3) δ 200.63, 171.01, 143.76, 140.46, 140.42, 140.24, 128.87, 127.48, 127.35, 126.95, 126.82, 118.80, 56.52, 52.36, 39.08, 38.47, 24.56.
HRMS (ESI) m/z calcd. for $C_{23}H_{25}O_5$ [M+H]$^+$ 381.1694, found 381.1696.

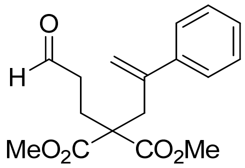

Dimethyl 2-(3-oxopropyl)-2-(2-phenylallyl)malonate (1l)
^{1}**H NMR** (500 MHz, CDCl3) δ 9.57 (t, *J* = 1.5 Hz, 1H), 7.32 – 7.28 (m, 4H), 7.28 – 7.23 (m, 1H), 5.27 (d, *J* = 1.5 Hz, 1H), 5.13 (s, 1H), 3.48 (s, 6H), 3.19 (s, 2H), 2.38 – 2.32 (m, 2H), 2.15 – 2.09 (m, 2H). ^{13}**C NMR** (125 MHz, CDCl3) δ 200.67, 170.98, 144.19, 141.33, 128.19, 127.68, 126.87, 118.79, 56.52, 52.33, 39.03, 38.48, 24.47.
HRMS (ESI) m/z calcd. for $C_{17}H_{21}O_5$ [M+H]$^+$ 305.1381, found 305.1384.

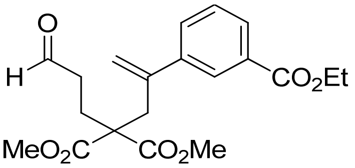

Dimethyl 2-(2-(3-(ethoxycarbonyl)phenyl)allyl)-2-(3-oxopropyl)malonate (1m)
^{1}**H NMR** (400 MHz, CDCl3) δ 9.39 (s, 1H), 7.77 (s, 1H), 7.73 (d, *J* = 7.8 Hz, 1H), 7.30 (d, *J* = 7.8 Hz, 1H), 7.18 (t, *J* = 7.8 Hz, 1H), 5.12 (s, 1H), 5.01 (s, 1H), 4.17 (q, *J* = 7.1 Hz, 2H), 3.25 (s, 6H), 3.03 (s, 2H), 2.18 (t, *J* = 7.7 Hz, 2H), 1.91 (t, *J* = 7.7 Hz, 2H), 1.19 (t, *J* = 7.1 Hz, 3H).
^{13}**C NMR** (100 MHz, CDCl3) δ 200.10, 170.55, 165.91, 143.28, 141.31, 131.13, 130.24, 128.49, 128.15, 127.43, 119.51, 60.82, 56.08, 51.98, 38.65, 37.98, 24.29, 14.08.
HRMS (ESI) m/z calcd. for $C_{20}H_{24}O_7Na$ [M+Na]$^+$ 399.1407, found 399.1414.

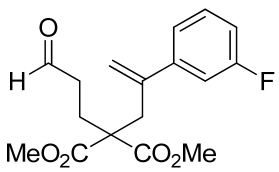

Dimethyl 2-(2-(3-fluorophenyl)allyl)-2-(3-oxopropyl)malonate (1n)
^{1}**H NMR** (400 MHz, CDCl3) δ 9.44 (s, 1H), 7.14 – 7.09 (m, 1H), 6.94 (d, *J* = 7.8 Hz, 1H), 6.87 (d, *J* = 10.1 Hz, 1H), 6.82 – 6.77 (m, 1H), 5.15 (s, 1H), 5.03 (s, 1H), 3.33 (s, 6H), 3.03 (s, 2H), 2.23 (t, *J* = 7.7 Hz, 2H), 1.96 (t, *J* = 7.7 Hz, 2H). ^{13}**C NMR** (100 MHz, CDCl3) δ 200.21, 170.63, 162.41 (d, J_{C-F} = 244 Hz, 1C), 143.52 (d, J_{C-F} = 8 Hz), 143.06 (d, J_{C-F} = 2 Hz), 129.62 (d, J_{C-F} = 8 Hz), 122.48 (d, J_{C-F} = 2 Hz), 119.45, 114.27 (d, J_{C-F} = 22 Hz), 113.60 (d, J_{C-F} = 22 Hz), 56.20, 52.01, 38.71, 38.06, 24.33. ^{19}**F NMR** (376 MHz, CDCl3) δ -113.33.
HRMS (ESI) m/z calcd. for $C_{17}H_{19}FO_5Na$ [M+Na]$^+$ 345.1102, found 345.1108.

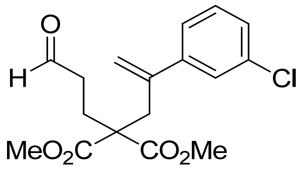

Dimethyl 2-(2-(3-chlorophenyl)allyl)-2-(3-oxopropyl)malonate (1o)
^{1}H NMR (400 MHz, $CDCl_3$) δ 9.62 (s, 1H), 7.27 – 7.26 (m, 1H), 7.25 – 7.21 (m, 2H), 7.20 – 7.15 (m, 1H), 5.29 (d, J = 1.1 Hz, 1H), 5.16 (s, 1H), 3.50 (s, 6H), 3.16 (s, 2H), 2.39 – 2.35 (m, 2H), 2.13 – 2.07 (m, 2H). **^{13}C NMR** (100 MHz, $CDCl_3$) δ 200.39, 170.80, 143.04, 142.93, 134.02, 129.52, 127.72, 126.85, 125.16, 119.86, 56.29, 52.35, 39.02, 38.45, 24.55.
HRMS (ESI) m/z calcd. for $C_{17}H_{19}ClO_5Na$ $[M+Na]^+$ 361.0807, found 361.0813.

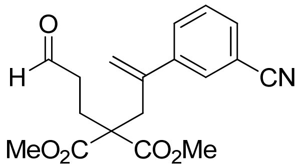

Dimethyl 2-(2-(3-cyanophenyl)allyl)-2-(3-oxopropyl)malonate (1p)
^{1}H NMR (400 MHz, $CDCl_3$) δ 9.63 (s, 1H), 7.58 – 7.48 (m, 3H), 7.41 (t, J = 7.7 Hz, 1H), 5.30 (s, 1H), 5.23 (s, 1H), 3.46 (s, 6H), 3.16 (s, 2H), 2.38 (t, J = 7.7 Hz, 2H), 2.11 – 2.05 (m, 2H).
^{13}C NMR (100 MHz, $CDCl_3$) δ 200.19, 170.64, 142.46, 142.26, 131.31, 131.11, 130.31, 129.13, 120.95, 118.49, 112.36, 56.19, 52.36, 38.98, 38.45, 24.69.
HRMS (ESI) m/z calcd. for $C_{18}H_{19}NO_5Na$ $[M+Na]^+$ 352.1148, found 352.1155.

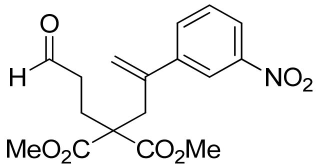

Dimethyl 2-(2-(3-nitrophenyl)allyl)-2-(3-oxopropyl)malonate (1q)
^{1}H NMR (400 MHz, $CDCl_3$) δ 9.63 (s, 1H), 8.12 – 8.08 (m, 2H), 7.62 (d, J = 7.8 Hz, 1H), 7.48 (t, J = 7.8 Hz, 1H), 5.37 (s, 1H), 5.27 (s, 1H), 3.45 (s, 6H), 3.21 (s, 2H), 2.39 (t, J = 7.7 Hz, 2H), 2.08 (t, J = 7.7 Hz, 2H). **^{13}C NMR** (100 MHz, $CDCl_3$) δ 200.25, 170.66, 148.03, 142.85, 142.13, 132.95, 129.29, 122.49, 121.47, 121.29, 56.19, 52.38, 38.95, 38.38, 24.66.
HRMS (ESI) m/z calcd. for $C_{17}H_{19}NO_7Na$ $[M+Na]^+$ 372.1046, found 372.1053.

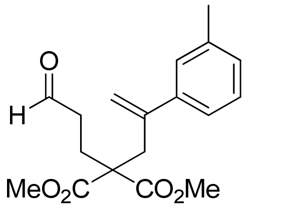

Dimethyl 2-(2-(3-methylphenyl)allyl)-2-(3-oxopropyl)malonate (1r)
^{1}H NMR (400 MHz, $CDCl_3$) δ 9.41 (s, 1H), 7.05 (t, J = 7.5 Hz, 1H), 6.98 – 6.93 (m, 3H), 5.12 (d, J = 0.9 Hz, 1H), 4.99 (s, 1H), 3.34 (s, 6H), 3.07 (s, 2H), 2.23 – 2.19 (m, 5H), 2.01 – 1.87 (m, 2H).
^{13}C NMR (100 MHz, $CDCl_3$) δ 200.30, 170.76, 144.33, 141.20, 137.45, 128.25, 127.96, 127.39, 123.90, 118.20, 56.37, 52.00, 38.76, 38.11, 24.27, 21.16.

HRMS (ESI) m/z calcd. for $C_{18}H_{23}O_5$ $[M+H]^+$ 319.1538, found 319.1540.

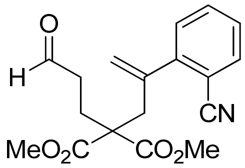

Dimethyl 2-(2-(2-cyanophenyl)allyl)-2-(3-oxopropyl)malonate (1s)
^{1}**H NMR** (400 MHz, $CDCl_3$) δ 9.64 (t, *J* = 1.2 Hz, 1H), 7.65 (dd, *J* = 7.7, 1.2 Hz, 1H), 7.53 (td, *J* = 7.7, 1.6 Hz, 1H), 7.36 (td, *J* = 7.7, 1.2 Hz, 1H), 7.31 (dd, *J* = 7.8, 0.4 Hz, 1H), 5.46 (d, *J* = 0.8 Hz, 1H), 5.36 (d, *J* = 0.8 Hz, 1H), 3.46 (s, 6H), 3.26 (s, 2H), 2.44 – 2.34 (m, 2H), 2.23 – 2.12 (m, 2H). ^{13}**C NMR** (100 MHz, $CDCl_3$) δ 200.43, 170.64, 145.18, 141.13, 133.28, 132.30, 129.05, 127.87, 123.67, 118.11, 111.31, 56.28, 52.40, 39.88, 39.02, 24.86.
HRMS (ESI) m/z calcd. for $C_{18}H_{19}NO_5Na$ $[M+Na]^+$ 352.1149, found 352.1155.

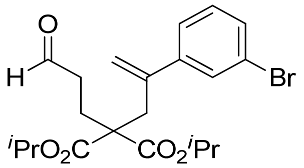

Diisopropyl 2-(2-(3-bromophenyl)allyl)-2-(3-oxopropyl)malonate (1t)
^{1}**H NMR** (400 MHz, $CDCl_3$) δ 9.58 (t, *J* = 1.3 Hz, 1H), 7.45 (t, *J* = 1.8 Hz, 1H), 7.40 (dt, *J* = 8.0, 1.2 Hz,, 1H), 7.25 (dt, *J* = 8.0, 1.2 Hz, 1H), 7.18 (t, *J* = 7.8 Hz, 1H), 5.27 (d, *J* = 1.2 Hz, 1H), 5.16 (d, *J* = 0.8 Hz, 1H), 4.86 – 4.79 (m, 2H), 3.13 (s, 2H), 2.34 – 2.26 (m, 2H), 2.11 – 2.01 (m, 2H), 1.18 (dd, *J* = 6.3, 2.0 Hz, 12H). ^{13}**C NMR** (100 MHz, $CDCl_3$) δ 200.52, 170.05, 144.04, 143.20, 130.65, 129.84, 129.65, 125.61, 122.32, 119.61, 69.36, 56.53, 38.91, 37.64, 24.11, 21.52.
HRMS (ESI) m/z calcd. for $C_{21}H_{28}O_5Br$ $[M+H]^+$ 439.1108, found 439.1115.

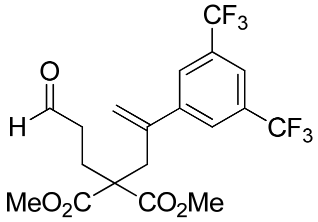

Dimethyl 2-(2-(3,5-bis(trifluoromethyl)phenyl)allyl)-2-(3-oxopropyl)malonate (1u)
^{1}**H NMR** (500 MHz, $CDCl_3$) δ 9.68 (s, 1H), 7.79 (s, 1H), 7.74 (s, 2H), 5.42 (s, 1H), 5.35 (s, 1H), 3.45 (s, 6H), 3.23 (s, 2H), 2.44 (t, *J* = 7.8 Hz, 2H), 2.18 – 2.12 (m, 2H).
^{13}**C NMR** (125 MHz, $CDCl_3$) δ 200.08, 170.58, 143.26, 141.72, 131.54 (q, $J_{C\text{-}F}$ = 33.2 Hz), 126.95, 123.46 (q, $J_{C\text{-}F}$ = 271.2 Hz), 122.17, 121.35 (m), 56.02, 52.34, 39.00, 38.51, 24.75.
^{19}**F NMR** (376 MHz, $CDCl_3$) δ -62.87.
HRMS (ESI) m/z calcd. for $C_{19}H_{18}F_6O_5Na$ $[M+Na]^+$ 463.0942, found 463.0950.

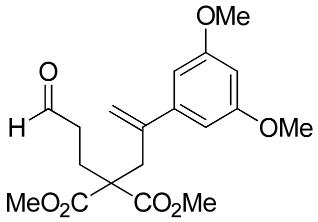

Dimethyl 2-(2-(3,5-dimethoxyphenyl)allyl)-2-(3-oxopropyl)malonate (1v)
^{1}H NMR (500 MHz, $CDCl_3$) δ 9.48 (s, 1H), 6.36 (s, 2H), 6.29 (s, 1H), 5.21 (s, 1H), 5.02 (s, 1H), 3.70 (s, 6H), 3.45 (s, 6H), 3.07 (s, 2H), 2.26 (t, *J* = 7.5 Hz, 2H), 2.02 (t, *J* = 7.5 Hz, 2H).
^{13}C NMR (125 MHz, $CDCl_3$) δ 200.53, 170.89, 160.46, 144.13, 143.31, 118.48, 105.01, 99.44, 56.53, 55.22, 52.21, 38.91, 38.24, 24.32.
HRMS (ESI) m/z calcd. for $C_{19}H_{25}O_7$ $[M+H]^+$ 365.1594, found 365.1595.

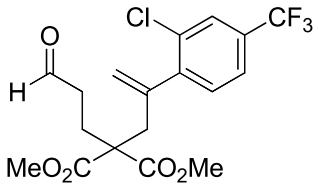

Dimethyl 2-(2-(2-chloro-4-(trifluoromethyl)phenyl)allyl)-2-(3-oxopropyl)malonate (1w)
^{1}H NMR (400 MHz, $CDCl_3$) δ 9.64 (s, 1H), 7.60 (s, 1H), 7.46 (dd, *J* = 8.0, 0.9 Hz, 1H), 7.28 (d, *J* = 8.0 Hz, 1H), 5.38 (s, 1H), 5.20 (d, *J* = 0.9 Hz, 1H), 3.44 (s, 6H), 3.23 (s, 2H), 2.39 (t, *J* = 7.6, 2H), 2.16 (t, *J* = 7.6, 2H). **^{13}C NMR** (100 MHz, $CDCl_3$) δ 200.19, 170.64, 143.90, 141.85, 132.94, 131.44, 130.97 (q, $J_{C\text{-}F}$ = 33.2 Hz, 1C), 126.50 (q, $J_{C\text{-}F}$ = 3.9 Hz, 1C),), 123.37 (q, $J_{C\text{-}F}$ = 3.7 Hz, 1C), 123.16 (q, $J_{C\text{-}F}$ = 270.8 Hz, 1C), 56.21, 52.25, 39.22, 39.02, 24.92. **^{19}F NMR** (376 MHz, $CDCl_3$) δ -62.83. **HRMS** (ESI) m/z calcd. for $C_{18}H_{18}ClF_3O_5Na$ $[M+Na]^+$ 429.0679, found 429.0687.

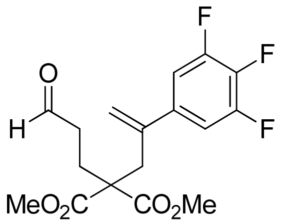

Dimethyl 2-(3-oxopropyl)-2-(2-(3,4,5-trifluorophenyl)allyl)malonate (1x)
^{1}H NMR (500 MHz, $CDCl_3$) δ 9.63 (s, 1H), 6.94 – 6.84 (m, 2H), 5.27 (s, 1H), 5.17 (s, 1H), 3.52 (s, 6H), 3.08 (s, 2H), 2.38 (t, *J* = 7.5 Hz, 2H), 2.05 (t, *J* = 7.5 Hz, 2H); **^{13}C NMR** (125 MHz, $CDCl_3$) δ 200.24, 170.67, 151.76 (ddd, $J_{C\text{-}F}$ = 248.5, 10.1, 4.1 Hz, 1C), 141.43, 139.07 (td, $J_{C\text{-}F}$ = 250.9, 15.4 Hz, 1C), 137.28 (m, 1C), 120.61, 110.88 (dd, *J* = 16.5, 5.0 Hz, 1C), 56.17, 52.39, 38.97, 38.29, 24.60; **^{19}F NMR** (376 MHz, $CDCl_3$) δ -134.23 (d, *J* = 20.7 Hz, 2F), -161.52 (d, *J* = 20.7 Hz, F); **HRMS** (ESI) m/z calcd. for $C_{17}H_{17}F_3O_5Na$ $[M+Na]^+$ 381.0916, found 381.0920.

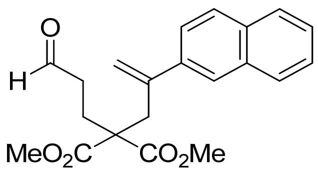

Dimethyl 2-(2-(naphthalen-2-yl)allyl)-2-(3-oxopropyl)malonate (1y)
^{1}H NMR (400 MHz, $CDCl_3$) δ 9.58 (t, *J* = 1.3 Hz, 1H), 7.85 – 7.74 (m, 4H), 7.53 – 7.43 (m, 3H), 5.42 (d, *J* = 1.4 Hz, 1H), 5.24 (d, *J* = 1.0 Hz, 1H), 3.42 (s, 6H), 3.33 (s, 2H), 2.40 – 2.36 (m, 2H), 2.21 – 2.15 (m, 2H). **^{13}C NMR** (100 MHz, $CDCl_3$) δ 200.59, 171.01, 144.06, 138.57, 133.08, 132.78, 128.06, 127.83, 127.57, 126.35, 126.07, 125.44, 125.23, 119.30, 56.58, 52.33, 39.10, 38.56, 24.62. **HRMS** (ESI) m/z calcd. for $C_{21}H_{23}O_5$ $[M+H]^+$ 355.1535, found 355.1540.

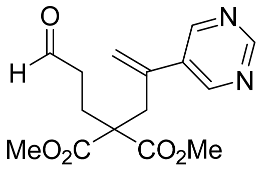

Dimethyl 2-(3-oxopropyl)-2-(2-(pyrimidin-5-yl)allyl)malonate (1z)
^{1}H NMR (500 MHz, $CDCl_3$) δ 9.69 (s, 1H), 9.11 (s, 1H), 8.66 (s, 2H), 5.40 (s, 1H), 5.35 (s, 1H), 3.49 (s, 6H), 3.19 (s, 2H), 2.45 (t, *J* = 7.5 Hz, 2H), 2.14 (t, *J* = 7.5 Hz, 2H).
^{13}C NMR (125 MHz, $CDCl_3$) δ 200.08, 170.52, 157.81, 154.70, 137.96, 134.32, 122.40, 55.98, 52.54, 39.02, 38.44, 24.90. **HRMS** (ESI) m/z calcd. for $C_{15}H_{19}O_5N_2$ $[M+H]^+$ 307.1287, found 307.1288.

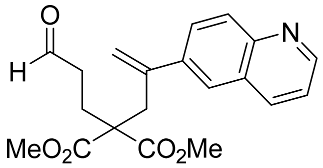

Dimethyl 2-(3-oxopropyl)-2-(2-(quinolin-6-yl)allyl)malonate (1aa)

^{1}H NMR (400 MHz, $CDCl_3$) δ 9.60 (t, *J* = 1.2 Hz, 1H), 8.88 (dd, *J* = 4.0, 1.6 Hz, 1H), 8.14 (dd, *J* = 8.4, 0.8 Hz, 1H), 8.04 (d, *J* = 8.8 Hz, 1H), 7.72 (td, *J* = 8.8, 2.0 Hz,, 2H), 7.40 (dd, *J* = 8.0, 4.0 Hz, 1H), 5.42 (d, *J* = 1.2 Hz, 1H), 5.27 (d, *J* = 0.8 Hz, 1H), 3.39 (s, 6H), 3.30 (s, 2H), 2.43 – 2.35 (m, 2H), 2.19 – 2.13 (m, 2H). **^{13}C NMR** (100 MHz, $CDCl_3$) δ 200.44, 170.90, 150.55, 147.69, 143.41, 139.37, 136.18, 129.21, 128.75, 127.88, 125.35, 121.57, 120.22, 56.41, 52.35, 39.08, 38.60, 24.64. **HRMS** (ESI) m/z calcd. for $C_{20}H_{22}NO_5$ $[M+H]^+$ 356.1492, found 356.1492.

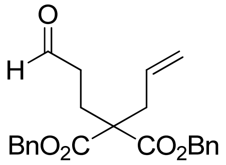

Dibenzyl 2-allyl-2-(3-oxopropyl)malonate (1ab)
^{1}H NMR (400 MHz, $CDCl_3$) δ 9.64 (s, 1H), 7.38 – 7.25 (m, 10H), 5.69 – 5.56 (m, 1H), 5.14 (s, 4H), 5.11 – 5.03 (m, 2H), 2.71 (d, *J* = 7.4 Hz, 2H), 2.44 – 2.36 (m, 2H), 2.27 – 2.20 (m, 2H). **^{13}C NMR** (125 MHz, $CDCl_3$) δ 200.54, 170.45, 135.28, 131.72, 128.60, 128.46, 128.33, 119.74,

67.24, 56.71, 38.89, 37.85, 24.89. **HRMS** (ESI) m/z calcd. for $C_{23}H_{24}O_5Na$ $[M+Na]^+$ 403.1507, found 403.1516.

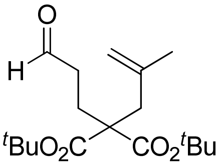

Di-tert-butyl 2-(2-methylallyl)-2-(3-oxopropyl)malonate (1ac)

^{1}H NMR (400 MHz, $CDCl_3$) δ 9.73 (t, *J* = 1.5 Hz, 1H), 4.86 – 4.83 (m, 1H), 4.75 (d, *J* = 0.9 Hz, 1H), 2.64 (s, 2H), 2.44 – 2.33 (m, 2H), 2.17 – 2.07 (m, 2H), 1.69 (s, 3H), 1.45 (s, 18H). **^{13}C NMR** (100 MHz, $CDCl_3$) δ 201.15, 170.40, 140.85, 115.55, 81.83, 56.68, 40.57, 39.14, 27.85, 24.64, 23.29. **HRMS** (ESI) m/z calcd. for $C_{18}H_{30}O_5Na$ $[M+Na]^+$ 349.1978, found 349.1985.

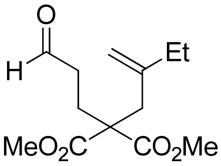

Dimethyl 2-(2-methylenebutyl)-2-(3-oxopropyl)malonate (1ad)

^{1}H NMR (400 MHz, $CDCl_3$) δ 9.73 (s, 1H), 4.90 (d, *J* = 1.4 Hz, 1H), 4.76 (s, 1H), 3.73 (s, 6H), 2.75 (s, 2H), 2.46 (t, *J* = 7.6 Hz, 2H), 2.24 – 2.19 (m, 2H), 1.90 (q, *J* = 7.4 Hz, 2H), 1.01 (t, *J* = 7.4 Hz, 3H). **^{13}C NMR** (100 MHz, $CDCl_3$) δ 200.77, 171.65, 145.68, 113.47, 56.30, 52.55, 39.70, 39.32, 29.07, 25.03, 12.32. **HRMS** (ESI) m/z calcd. for $C_{13}H_{21}O_5$ $[M+H]^+$ 257.1381, found 257.1383.

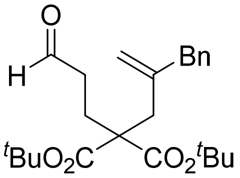

Di-tert-butyl 2-(2-benzylallyl)-2-(3-oxopropyl)malonate (1ae)

^{1}H NMR (400 MHz, $CDCl_3$) δ 9.66 (s, 1H), 7.32 – 7.24 (m, 2H), 7.23 – 7.13 (m, 3H), 4.91 (s, 1H), 4.83 (d, *J* = 1.2 Hz, 1H), 3.29 (s, 2H), 2.61 (s, 2H), 2.29 – 2.14 (m, 4H), 1.45 (s, 18H). **^{13}C NMR** (100 MHz, $CDCl_3$) δ 200.88, 170.32, 143.88, 139.14, 129.14, 128.37, 126.25, 115.83, 81.83, 56.96, 43.86, 39.08, 37.56, 27.85, 24.54. **HRMS** (ESI) m/z calcd. for $C_{24}H_{35}O_5$ $[M+H]^+$ 403.2477, found 403.2479.

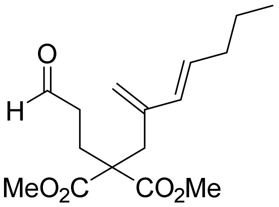

Dimethyl (E)-2-(2-methylenehept-3-en-1-yl)-2-(3-oxopropyl)malonate (1af)

^{1}H NMR (400 MHz, $CDCl_3$) δ 9.71 (t, *J* = 1.2 Hz, 1H), 5.98 (d, *J* = 16.0 Hz, 1H), 5.73 (dt, *J* = 16.0, 6.8 Hz, 1H), 5.08 (s, 1H), 4.80 (s, 1H), 3.71 (s, 6H), 2.87 (s, 2H), 2.48 – 2.44 (m, 2H), 2.22 – 2.17 (m, 2H), 2.09 – 2.03 (m, 2H), 1.41 (q, *J* = 7.6 Hz, 2H), 0.91 – 0.87 (t, *J* = 7.6 Hz, 4H). **^{13}C NMR** (126 MHz, $CDCl_3$) δ 200.79, 171.40, 140.81, 132.10, 131.10, 116.71, 56.86, 52.51, 39.49, 35.21, 34.96, 25.06, 22.45, 13.80. **HRMS** (ESI) m/z calcd. for $C_{16}H_{25}O_5$ $[M+H]^+$ 297.1696, found 297.1697.

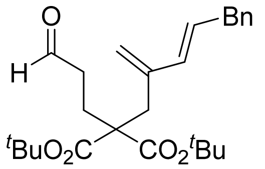

Di-tert-butyl (E)-2-(2-methylene-5-phenylpent-3-en-1-yl)-2-(3-oxopropyl)malonate (1ag)

^{1}H NMR (400 MHz, $CDCl_3$) δ 9.61 (t, *J* = 1.6 Hz, 1H), 7.34 – 7.29 (m, 2H), 7.25 – 7.18 (m, 3H), 6.10 (dq, *J* = 15.6, 1.2 Hz, 1H), 5.93 (dt, *J* = 15.6, 6.8 Hz, 1H), 5.16 – 5.10 (t, *J* = 0.8 Hz, 1H), 4.94 (d, *J* = 1.2 Hz, 1H), 3.43 (dd, *J* = 6.8, 1.2 Hz, 2H), 2.79 (d, *J* = 1.2 Hz, 2H), 2.40 – 2.32 (m, 2H), 2.18 – 2.10 (m, 2H), 1.47 (s, 18H). **^{13}C NMR** (100 MHz, $CDCl_3$) δ 201.11, 170.24, 140.84, 139.97, 133.83, 129.22, 128.62, 128.51, 126.18, 116.82, 81.89, 57.61, 39.41, 39.17, 34.14, 27.91, 24.51. **HRMS** (ESI) m/z calcd. for $C_{26}H_{36}O_5Na$ $[M+Na]^+$ 451.2446, found 451.2455.

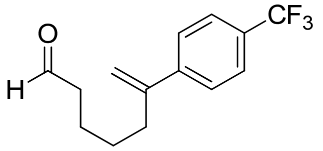

6-(4-(Trifluoromethyl)phenyl)hept-6-enal (1ah)

^{1}H NMR (400 MHz, $CDCl_3$) δ 9.75 (t, *J* = 1.6 Hz, 1H), 7.59 (d, *J* = 8.0 Hz, 2H), 7.50 (d, *J* = 8.0 Hz, 2H), 5.35 (s, 1H), 5.18 (d, *J* = 1.2 Hz, 1H), 2.56 (t, *J* = 7.6 Hz, 2H), 2.44 (td, *J* = 7.2, 1.6 Hz, 2H), 1.71–1.64 (m, 2H), 1.53–1.45 (m, 2H). **^{13}C NMR** (100 MHz, $CDCl_3$) δ 202.32, 146.92, 144.73, 129.36 (q, $J_{C\text{-}F}$ = 32.2 Hz), 126.41, 125.73 (q, $J_{C\text{-}F}$ = 3.6 Hz), 124.44 (q, $J_{C\text{-}F}$ = 270.2 Hz), 114.51, 43.60, 34.89, 27.46, 21.53. **^{19}F NMR** (376 MHz, $CDCl_3$) δ -62.43. **HRMS** (ESI) m/z calcd. for $C_{14}H_{16}F_3O$ $[M+H]^+$ 257.1142, found 257.1148.

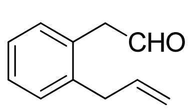

2-(2-Allylphenyl)acetaldehyde (1ai)

^{1}H NMR (500 MHz, $CDCl_3$) δ 9.73 (t, *J* = 2.5 Hz, 1H), 7.34 – 7.25 (m, 3H), 7.22– 7.21 (m, 1H), 6.01– 5.93 (m, 1H), 5.13 (dq, *J* = 10.0, 1.5 Hz, 1H), 4.99 (dq, *J* = 17.0, 1.5 Hz, 1H), 3.75 (d, *J* = 2.2 Hz, 2H), 3.41 (d, *J* = 6.1 Hz, 2H). **^{13}C NMR** (125 MHz, $CDCl_3$) δ 199.62, 138.73, 136.51, 130.95, 130.37, 128.50, 127.98, 127.04, 116.40, 48.07, 37.51. **HRMS** (ESI) m/z calcd. for $C_{11}H_{13}O$ $[M+H]^+$ 161.0959, found 161.0960.

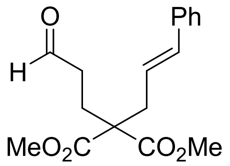

Dimethyl 2-cinnamyl-2-(3-oxopropyl)malonate (1aj)

^{1}H NMR (400 MHz, $CDCl_3$) δ 9.75 (s, 1H), 7.37 – 7.28 (m, 4H), 7.27 – 7.21 (m, 1H), 6.47 (d, *J* = 15.7 Hz, 1H), 6.11 – 5.98 (m, 1H), 3.76 (s, 6H), 2.82 (dd, *J* = 7.5, 1.0 Hz, 2H), 2.55 (t, *J* = 7.6 Hz, 2H), 2.29 – 2.25 (m, 2H). **^{13}C NMR** (125 MHz, $CDCl_3$) δ 200.67, 171.22, 136.84, 134.37, 128.56, 127.61, 126.28, 123.34, 57.12, 52.64, 39.22, 37.54, 25.38. **HRMS** (ESI) m/z calcd. for $C_{17}H_{21}O_5$ $[M+H]^+$ 305.1379, found 305.1384.

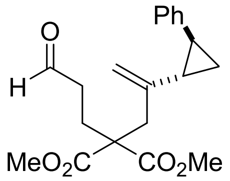

Dimethyl 2-(3-oxopropyl)-2-(2-(2-phenylcyclopropyl)allyl)malonate (1ak)

^{1}H NMR (400 MHz, $CDCl_3$) δ 9.54 (t, *J* = 1.2 Hz, 1H), 7.25 (t, *J* = 7.6 Hz, 2H), 7.17 – 7.11 (m, 1H), 7.06 – 6.99 (m, 2H), 4.82 (d, *J* = 0.8 Hz, 1H), 4.75 (d, *J* = 0.8 Hz, 1H), 3.57 (s, 3H), 3.51 (s, 3H), 2.87 (s, 2H), 2.42 – 2.35 (m, 2H), 2.32 – 2.15 (m, 2H), 1.84 (dt, *J* = 8.8, 5.2 Hz, 1H), 1.42 – 1.34 (m, 1H), 1.25 – 1.21 (m, 1H), 1.12 (dt, *J* = 8.8, 5.2 Hz, 1H).
^{13}C NMR (100 MHz, $CDCl_3$) δ 200.70, 171.47, 171.28, 144.55, 142.40, 128.38, 125.75, 125.42, 112.20, 56.54, 52.50, 52.40, 41.06, 39.21, 28.67, 26.88, 25.06, 17.13. **HRMS** (ESI) m/z calcd. for $C_{20}H_{25}O_5$ $[M+H]^+$ 345.1692, found 345.1697.

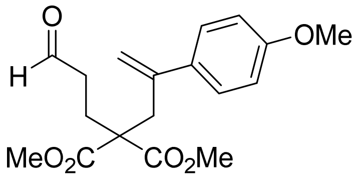

Dimethyl 2-(2-(4-methoxyphenyl)allyl)-2-(3-oxopropyl)malonate (1al)

^{1}H NMR (400 MHz, $CDCl_3$) δ 9.60 (d, *J* = 0.8 Hz, 1H), 7.23 (d, *J* = 8.0 Hz, 2H), 6.84 (d, *J* = 8.0 Hz, 2H), 5.20 (s, 1H), 5.05 (s, 1H), 3.80 (s, 3H), 3.50 (s, 6H), 3.16 (s, 2H), 2.36 (t, *J* = 8.0 Hz, 2H), 2.14 – 2.10 (m, 2H). **^{13}C NMR** (100 MHz, $CDCl_3$) δ 200.71, 171.04, 159.20, 143.53, 133.73, 127.98, 117.52, 113.50, 56.52, 55.28, 52.37, 39.07, 38.49, 24.47. **HRMS** (ESI) m/z calcd. for $C_{18}H_{23}O_6$ $[M+H]^+$ 335.1485, found 335.1489.

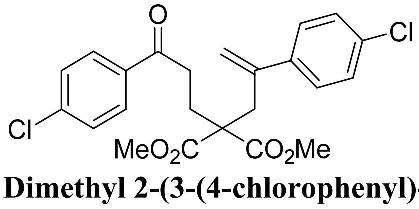

Dimethyl 2-(3-(4-chlorophenyl)-3-oxopropyl)-2-(2-(4-chlorophenyl)allyl)malonate (1am)

^{1}H NMR (400 MHz, CDCl$_3$) δ 7.81 (d, *J* = 8.4 Hz, 2H), 7.40 (d, *J* = 8.4 Hz, 2H), 5.25 (d, *J* = 1.6 Hz, 1H), 5.16 (d, *J* = 1.6 Hz, 1H), 3.47 (s, 6H), 3.21 (s, 2H), 2.87 (t, *J* = 7.6 Hz, 2H), 2.22 (dd, *J* = 8.5, 6.8 Hz, 2H). **^{13}C NMR** (100 MHz, CDCl$_3$) δ 197.41, 171.04, 143.05, 139.82, 139.50, 134.88, 133.43, 129.41, 128.89, 128.26, 128.21, 119.44, 56.61, 52.33, 39.10, 33.76, 26.85. **HRMS** (ESI) m/z calcd. for $C_{23}H_{23}O_5Cl_2$ [M+H]$^+$ 449.0926, found 449.0917.

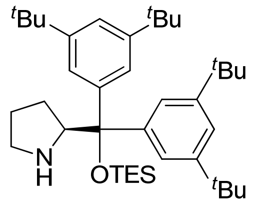

A12, **(*S*)-2-(bis(3,5-di-tert-butylphenyl)((triethylsilyl)oxy)methyl)pyrrolidine**
^{1}H NMR (400 MHz, CDCl$_3$) δ 7.33 (d, *J* = 2.0 Hz, 2H), 7.29 (t, *J* = 1.6 Hz, 2H), 7.25 (d, *J* = 1.6 Hz, 2H), 4.22 (dd, *J* = 7.6, 6.0 Hz, 1H), 2.72 – 2.66 (m, 1H), 2.32 – 2.27 (m, 1H), 1.80 – 1.74 (m, 1H), 1.70 – 1.62 (m, 1H), 1.53 – 1.43 (m, 1H), 1.29 (s, 18H), 1.28 (s, 18H), 1.04 – 0.92 (m, 1H), 0.85 (t, *J* = 8.0 Hz, 9H), 0.29 (q, *J* = 7.6 Hz, 6H). **^{13}C NMR** (125 MHz, CDCl$_3$) δ 149.16, 148.85, 143.66, 143.23, 123.71, 123.41, 120.47, 120.22, 83.97, 65.83, 46.57, 34.83, 31.50, 31.48, 27.43, 25.01, 7.32, 6.22. **HRMS** (ESI) m/z calcd. for $C_{39}H_{66}NOSi$ [M+H]$^+$ 592.4918, found 592.4908.

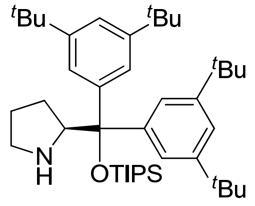

A14, (*S*)-2-(bis(3,5-di-tert-butylphenyl)((triisopropylsilyl)oxy)methyl)pyrrolidine
^{1}H NMR (500 MHz, CDCl$_3$) δ 7.31 (s, 2H), 7.29 (d, *J* = 1.5 Hz, 2H), 7.26 (d, *J* = 1.0 Hz, 2H), 4.37 (dd, *J* = 8.0, 5.5 Hz, 1H), 2.64 (dd, *J* = 14.5, 7.5 Hz, 1H), 2.10 (dd, *J* = 15.5, 7.5 Hz, 1H), 1.92 – 1.86 (m, 1H), 1.74 – 1.67 (m, 1H), 1.46 – 1.40 (m, 1H), 1.29 (s, 18H), 1.28 (s, 18H), 0.95 – 0.88 (m, 21H), 0.86 – 0.78 (m, 1H). **^{13}C NMR** (125 MHz, CDCl$_3$) δ 149.04, 148.61, 143.74, 143.13, 124.17, 124.04, 120.67, 120.22, 84.77, 65.61, 46.44, 34.81, 31.48, 31.45, 27.58, 24.93, 18.55, 17.74, 13.83, 12.32. **HRMS** (ESI) m/z calcd. for $C_{42}H_{72}NOSi$ [M+H]$^+$ 634.5390, found 634.5378.

General procedure for racemic radical [2+1] cycloaddition toward bicyclo[3.1.0]hexenes

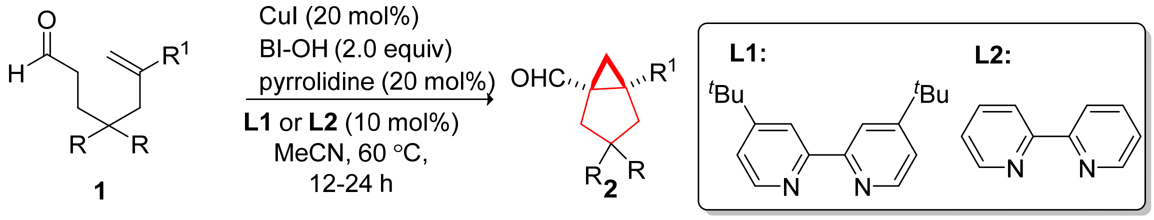

To a flame-dried Schlenk tube equipped with a magnetic stir bar were added **1** (0.2 mmol), CuI (7.6 mg, 20 mol%), Ligand (10 mol%) and BI-OH (108 mg, 0.4 mmol). The tube was evacuated and backfilled with argon for three times. Pyrrolidine (3.3 μL, 20 mol%) and freshly degassed acetonitrile (2.0 mL) was added *via* syringe. The tube was stirred at 60 °C for 12-24 h until TLC monitored the full completion of starting material. After completion, solvent was removed under reduced pressure, and the residue was diluted with ethyl acetate (15 mL), washed with saturated $NaHCO_3$ solution, then washed with brine, dried with $MgSO_4$, filtered and concentrated. Flash chromatography (petroleum ether/ ethyl acetate =10/1-5/1) gave the corresponding products **2**.

General procedure for asymmetric radical [2+1] cycloaddition toward bicyclo[3.1.0]hexenes (Conditions A)

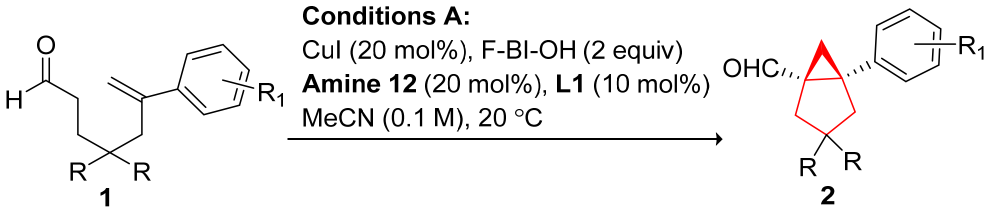

To a flame-dried Schlenk tube equipped with a magnetic stir bar were added **1** (0.1 mmol), CuI (3.8 mg 20 mol%), **L1** (2.6 mg, 10 mol%), F-BI-OH (58 mg, 0.2 mmol) and **Amine 12** (12.0 mg, 20 mol%). The tube was evacuated and backfilled with argon for three times, the freshly degassed dry acetonitrile (1.0 mL) was added *via* syringe. The tube was stirred at 20 °C for 48 hours. After completion, solvent was removed under reduced pressure, and the residue was diluted with ethyl acetate (15 mL), washed with saturated $NaHCO_3$ solution, then washed with brine, dried with $MgSO_4$, filtered and concentrated. Flash chromatography (petroleum ether/ ethyl acetate =10/1-5/1) gave the corresponding products **2**.

General procedure for asymmetric radical [2+1] cycloaddition toward bicyclo[3.1.0]hexenes (Conditions B)

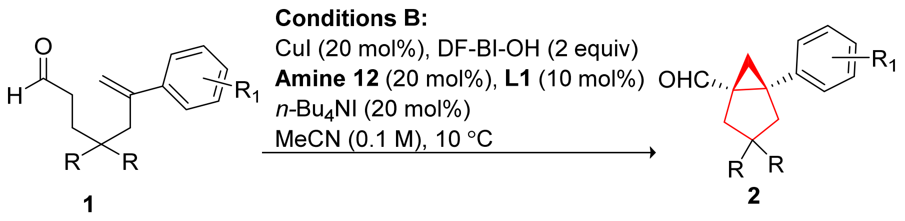

To a flame-dried Schlenk tube equipped with a magnetic stir bar were added **1** (0.1 mmol), CuI (3.8 mg 20 mol%), **L1** (2.6 mg, 10 mol%), DF-BI-OH (60 mg, 0.2 mmol), *n*-Bu_4NI (7.4 mg, 20 mol%) and **Amine 12** (12.0 mg, 20 mol%). The tube was evacuated and backfilled with argon for three times, the freshly degassed dry acetonitrile (1.0 mL) was added *via* syringe. The tube was stirred at 10 °C for 72 hours. After completion, solvent was removed under reduced pressure, and the residue was diluted with ethyl acetate (15 mL), washed with saturated $NaHCO_3$ solution, then washed with brine, dried with $MgSO_4$, filtered and concentrated. Flash chromatography (petroleum ether/ ethyl acetate =10/1-5/1) gave the corresponding products **2**.

General procedure for asymmetric radical [2+1] cycloaddition toward bicyclo[3.1.0]hexenes (Conditions C)

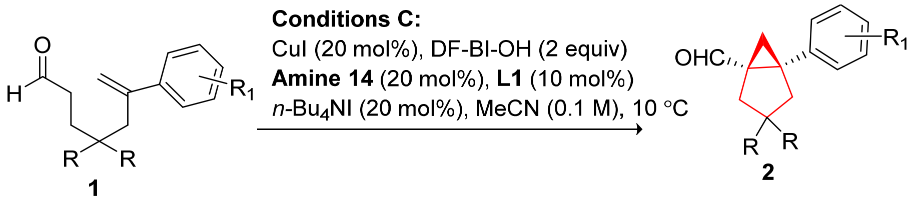

To a flame-dried Schlenk tube equipped with a magnetic stir bar were added **1** (0.1 mmol), CuI (3.8 mg 20 mol%), **L1** (2.6 mg, 10 mol%), DF-BI-OH (60 mg, 0.2 mmol), *n*-Bu_4NI (7.4 mg, 20 mol%) and **Amine 14** (12.8 mg, 20 mol%). The tube was evacuated and backfilled with argon for three times, the freshly degassed dry acetonitrile (1.0 mL) was added *via* syringe. The tube was stirred at 10 °C for 72 hours. After completion, solvent was removed under reduced pressure, and the residue was diluted with saturated $NaHCO_3$ solution, then extracted with ethyl acetate. The organic layer was washed with brine, dried with $MgSO_4$, filtered and concentrated. Flash chromatography (petroleum ether/ ethyl acetate =10/1-5/1) gave the corresponding products **2**.

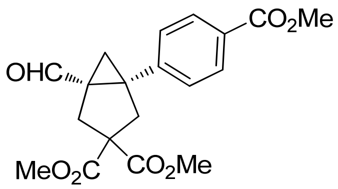

Dimethyl 1-formyl-5-(4-(methoxycarbonyl)phenyl)bicyclo[3.1.0]hexane-3,3-dicarboxylate 2a, 78% yield, colorless oil, analytical TLC (silica gel 60), 25% EtOAc in *n*-hexane, R_f = 0.45; **^{1}H NMR** (500 MHz, $CDCl_3$) δ 8.54 (s, 1H), 7.99 (d, J = 8.4 Hz, 2H), 7.42 – 7.38 (m, 2H), 3.91 (s, 3H), 3.79 (s, 3H), 3.76 (s, 3H), 3.20 (d, J = 14.5 Hz, 1H), 2.97 (d, J = 14.5 Hz, 1H), 2.89 (dd,

J = 14.5, 1.5 Hz, 1H), 2.80 (d, J = 14.5 Hz, 1H), 2.05 (d, J = 6.5 Hz, 1H), 1.49 (d, J = 6.5 Hz, 1H). **^{13}C NMR** (100 MHz, $CDCl_3$) δ 198.73, 172.52, 171.21, 166.54, 143.42, 130.12, 129.55, 129.33, 58.06, 53.37, 53.28, 52.20, 45.54, 44.44, 43.79, 34.37, 21.96.
HRMS (ESI) m/z calcd. for $C_{19}H_{20}O_7Na$ $[M+Na]^+$ 383.1094, found 383.1101.
HPLC analysis: Chiralcel IA, *i*-PrOH/*n*-hexane = 90/10, flow rate 1.0 mL/min. λ = 254 nm, t(minor) = 18.28 min, t(major) = 20.3 min, 95:5 er.

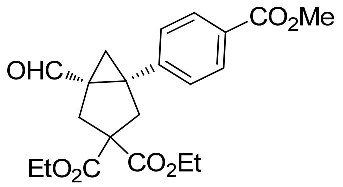

Diethyl 1-formyl-5-(4-(methoxycarbonyl)phenyl)bicyclo[3.1.0]hexane-3,3-dicarboxylate
2b, 82% yield, yellow oil, analytical TLC (silica gel 60), 25% EtOAc in *n*-hexane, R_f = 0.43;
^{1}H NMR (400 MHz, $CDCl_3$) δ 8.55 (s, 1H), 8.00 (d, J = 8.4 Hz, 2H), 7.41 (d, J = 8.4 Hz, 2H), 4.32 – 4.16 (m, 4H), 3.92 (s, 3H), 3.19 (d, J = 14.4 Hz, 1H), 2.91 (dt, J = 14.5, 7.8 Hz, 2H), 2.97 – 2.87 (m, 1H), 2.05 (d, J = 6.4 Hz, 1H), 1.53 (d, J = 6.4 Hz, 1H), 1.33–1.25 (m, 6H).
^{13}C NMR (100 MHz, $CDCl_3$) δ 198.85, 172.09, 170.77, 166.57, 143.58, 130.11, 129.51, 129.34, 62.25, 62.13, 58.25, 52.20, 45.68, 44.55, 43.73, 34.25, 22.08, 14.02, 13.99.
HRMS (ESI) m/z calcd. for $C_{21}H_{24}O_7Na$ $[M+Na]^+$ 411.1401, found 411.1412.
HPLC analysis: Chiralcel IA, *i*-PrOH/*n*-hexane = 90/10, flow rate 1.0 mL/min. λ = 230 nm, t(minor) = 15.03 min, t(major) = 16.89 min, 92.5:7.5 er.

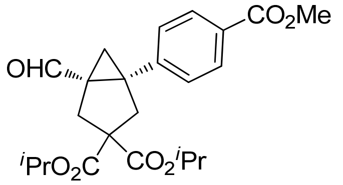

Diisopropyl-1-formyl-5-(4-(methoxycarbonyl)phenyl)bicyclo[3.1.0]hexane-3,3-dicarboxylate
2c, 85% yield, light-yellow oil, analytical TLC (silica gel 60), 20% EtOAc in *n*-hexane, R_f = 0.4;
^{1}H NMR (500 MHz, $CDCl_3$) δ 8.55 (s, 1H), 8.01 (d, J = 8.3 Hz, 2H), 7.42 (d, J = 8.3 Hz, 2H), 5.12–5.04 (m, 2H), 3.93 (s, 3H), 3.16 (d, J = 14.5 Hz, 1H), 2.90 (q, J = 14.4 Hz, 2H), 2.76 (d, J = 14.5 Hz, 1H), 2.05 (d, J = 6.3 Hz, 1H), 1.55 (d, J = 6.3 Hz, 1H), 1.31–1.25 (m, 9H), 1.24 (d, J = 6.3 Hz, 3H). **^{13}C NMR** (125 MHz, $CDCl_3$) δ 198.98, 171.61, 170.30, 166.61, 143.70, 130.11, 129.47, 129.36, 69.84, 69.64, 58.44, 52.21, 45.82, 44.66, 43.74, 34.18, 22.24, 21.53, 21.50, 21.49.
HRMS (ESI) m/z calcd. for $C_{23}H_{28}O_7Na$ $[M+Na]^+$ 439.1714, found 439.1720.
HPLC analysis: Chiralcel OD-H, *i*-PrOH/*n*-hexane = 90/10, flow rate 1.0 mL/min. λ = 230 nm, t(minor) = 8.31 min, t(major) = 9.21 min, 92.5:7.5 er.

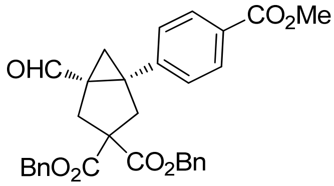

Dibenzyl 1-formyl-5-(4-(methoxycarbonyl)phenyl)bicyclo[3.1.0]hexane-3,3-dicarboxylate
2d, 70% yield, colorless oil, analytical TLC (silica gel 60), 20% EtOAc in *n*-hexane, $R_f = 0.42$;
^{1}H NMR (400 MHz, $CDCl_3$) δ 8.54 (s, 1H), 7.99 (d, *J* = 8.2 Hz, 2H), 7.37 (d, *J* = 8.3 Hz, 2H), 7.36 – 7.33 (m, 6H), 7.27 – 7.24 (m, 3H), 5.16 (d, *J* = 1.9 Hz, 2H), 5.13 (s, 2H), 3.93 (s, 3H), 3.24 (d, *J* = 14.4 Hz, 1H), 3.00 (d, *J* = 14.4 Hz, 1H), 2.90 (d, *J* = 14.5 Hz, 1H), 2.82 (d, *J* = 14.5 Hz, 1H), 2.02 (d, *J* = 6.5 Hz, 1H), 1.46 (d, *J* = 6.5 Hz, 1H). **^{13}C NMR** (100 MHz, $CDCl_3$) δ 198.61, 171.69, 170.40, 166.55, 143.40, 135.03, 134.98, 130.11, 129.57, 129.31, 128.63, 128.62, 128.54, 128.49, 128.26, 128.06, 67.84, 58.56, 52.17, 45.67, 44.54, 43.84, 34.40, 22.17.
HRMS (ESI) m/z calcd. for $C_{31}H_{28}O_7Na$ $[M+Na]^+$ 535.1718, found 535.1727.
HPLC analysis: Chiralcel IA, *i*-PrOH/*n*-hexane = 90/10, flow rate 1.0 mL/min. λ = 230 nm, t(minor) = 17.09 min, t(major) = 19.84 min, 93.5:6.5 er.

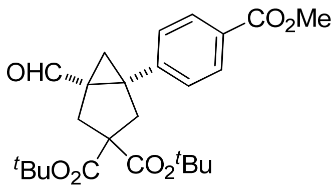

Di-tert-butyl 1-formyl-5-(4-(methoxycarbonyl)phenyl)bicyclo[3.1.0]hexane-3,3 dicarboxyl ate
2e, 35% yield (80% conversion), colorless oil, analytical TLC (silica gel 60), 20% EtOAc in *n*-hexane, $R_f = 0.33$; **^{1}H NMR** (500 MHz, $CDCl_3$) δ 8.55 (s, 1H), 8.00 (d, *J* = 8.2 Hz, 2H), 7.42 (d, *J* = 8.3 Hz, 2H), 3.92 (s, 3H), 3.08 (d, *J* = 14.4 Hz, 1H), 2.83 (s, 2H), 2.68 (d, *J* = 14.4 Hz, 1H), 2.03 (d, *J* = 6.2 Hz, 1H), 1.56 (d, *J* = 6.2 Hz, 1H), 1.48 (d, *J* = 9.1 Hz, 18H).
^{13}C NMR (125 MHz, $CDCl_3$) δ 199.16, 171.25, 169.86, 166.63, 143.91, 130.06, 129.39, 129.36, 82.28, 81.97, 59.72, 52.19, 45.89, 44.71, 43.66, 34.07, 27.81, 22.33.
HRMS (ESI) m/z calcd. for $C_{25}H_{32}O_7Na$ $[M+Na]^+$ 467.2028, found 467.2040.

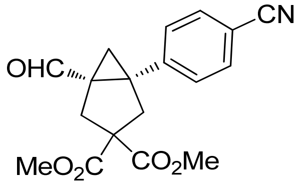

Dimethyl 1-(4-cyanophenyl)-5-formylbicyclo[3.1.0]hexane-3,3-dicarboxylate
2f, 80% yield, yellow oil, analytical TLC (silica gel 60), 25% EtOAc in *n*-hexane, $R_f = 0.25$;
^{1}H NMR (500 MHz, $CDCl_3$) δ 8.62 (s, 1H), 7.64 (d, *J* = 7.6 Hz, 2H), 7.45 (d, *J* = 7.8 Hz, 2H), 3.80 (s, 3H), 3.78 (s, 3H), 3.21 (d, *J* = 14.5 Hz, 1H), 2.97 (d, *J* = 14.5 Hz, 1H), 2.87 (d, *J* = 14.5 Hz, 1H), 2.82 (d, *J* = 14.5 Hz, 1H), 2.05 (d, *J* = 6.6 Hz, 1H), 1.51 (d, *J* = 6.5 Hz, 1H).
^{13}C NMR (125 MHz, $CDCl_3$) δ 198.22, 172.35, 171.12, 143.68, 132.68, 130.16, 118.38, 111.68, 58.26, 53.47, 53.37, 45.82, 44.56, 43.74, 34.52, 22.10.
HRMS (ESI) m/z calcd. for $C_{18}H_{17}NO_5Na$ $[M+Na]^+$ 350.0997, found 350.0999.

HPLC analysis: Chiralcel AD-H, *i*-PrOH/*n*-hexane = 80/20, flow rate 1.0 mL/min. λ = 230 nm, t(major) = 12.58 min, t(minor) = 15.27 min, 91.5:8.5 er.

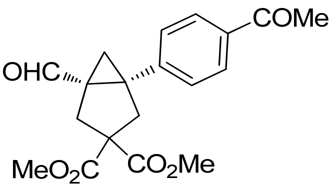

Dimethyl 1-(4-acetylphenyl)-5-formylbicyclo[3.1.0]hexane-3,3-dicarboxylate
2g, 80% yield, yellow oil, analytical TLC (silica gel 60), 25% EtOAc in *n*-hexane, R_f = 0.25;
^{1}H NMR (400 MHz, $CDCl_3$) δ 8.58 (s, 1H), 7.93 (d, *J* = 8.4 Hz, 2H), 7.43 (d, *J* = 8.3 Hz, 2H), 3.81 (s, 3H), 3.78 (s, 3H), 3.22 (d, *J* = 14.4 Hz, 1H), 2.98 (d, *J* = 14.4 Hz, 1H), 2.91 (dd, *J* = 14.4, 1.4 Hz, 1H), 2.82 (d, *J* = 14.4 Hz, 1H), 2.60 (s, 3H), 2.06 (d, *J* = 6.4 Hz, 1H), 1.51 (d, *J* = 6.4 Hz, 1H). **^{13}C NMR** (100 MHz, $CDCl_3$) δ 198.55, 197.33, 172.47, 171.22, 143.61, 136.50, 129.52, 128.87, 58.26, 53.33, 53.24, 45.66, 44.55, 43.84, 34.51, 26.58, 22.10.
HRMS (ESI) m/z calcd. for $C_{19}H_{20}O_6$ $[M+H]^+$ 345.1324, found 345.1332.
HPLC analysis: Chiralcel OD-H, *i*-PrOH/*n*-hexane = 80/20, flow rate 1.0 mL/min. λ = 230 nm, t(major) = 16.17 min, t(minor) = 18.64 min, 93.5:6.5 er.

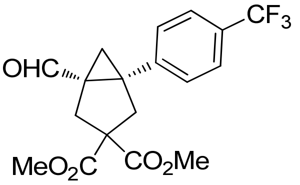

Dimethyl 1-formyl-5-(4-(trifluoromethyl)phenyl)bicyclo[3.1.0]hexane-3,3-dicarboxylate
2h, 85% yield, yellow oil, analytical TLC (silica gel 60), 20% EtOAc in *n*-hexane, R_f = 0.55;
^{1}H NMR (500 MHz, $CDCl_3$) δ 8.58 (s, 1H), 7.60 (d, *J* = 8.2 Hz, 2H), 7.46 (d, *J* = 8.1 Hz, 2H), 3.81 (s, 3H), 3.77 (s, 3H), 3.22 (d, *J* = 14.5 Hz, 1H), 2.98 (d, *J* = 14.4 Hz, 1H), 2.88 (dd, *J* = 14.5, 1.2 Hz, 1H), 2.82 (d, *J* = 14.5 Hz, 1H), 2.05 (d, *J* = 6.5 Hz, 1H), 1.51 (d, *J* = 6.5 Hz, 1H).
^{13}C NMR (125 MHz, $CDCl_3$) δ 198.62, 172.48, 171.20, 142.41, 130.05 (q, J_{C-F} = 32.4 Hz), 129.75, 125.85 (q, J_{C-F} = 3.6 Hz), 123.89 (q, J_{C-F} = 270.5 Hz), 58.12, 53.40, 53.31, 45.43, 44.32, 43.92, 34.42, 22.03. **^{19}F NMR** (376 MHz, $CDCl_3$) δ -62.66.
HRMS (ESI) m/z calcd. for $C_{18}H_{17}F_3O_5Na$ $[M+Na]^+$ 393.0921, found 393.0921.
HPLC analysis: Chiralcel OD-H, *i*-PrOH/*n*-hexane = 80/20, flow rate 1.0 mL/min. λ = 230 nm, t(major) = 6.23 min, t(minor) = 7.47 min, 94.5:5.5 er.

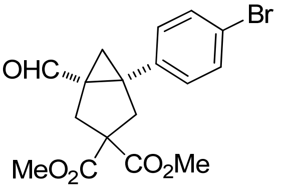

Dimethyl 1-formyl-5-(4-bromophenyl)bicyclo[3.1.0]hexane-3,3-dicarboxylate
2i, 75% yield, yellowish oil, analytical TLC (silica gel 60), 25% EtOAc in *n*-hexane, R_f = 0.42;

^{1}H NMR (500 MHz, $CDCl_3$) δ 8.57 (s, 1H), 7.46 (d, *J* = 8.3 Hz, 2H), 7.21 (d, *J* = 8.4 Hz, 2H), 3.80 (s, 3H), 3.77 (s, 3H), 3.18 (d, *J* = 14.5 Hz, 1H), 2.95 (d, *J* = 14.5 Hz, 1H), 2.84 (d, *J* = 14.5 Hz, 1H), 2.79 (d, *J* = 14.5 Hz, 1H), 1.99 (d, *J* = 6.4 Hz, 1H), 1.45 (d, *J* = 6.4 Hz, 1H).
^{13}C NMR (100 MHz, $CDCl_3$) δ 198.89, 172.53, 171.26, 137.42, 132.00, 130.96, 121.69, 58.04, 53.32, 53.23, 45.19, 44.18, 43.99, 34.39, 22.05.
HRMS (ESI) m/z calcd. for $C_{17}H_{17}BrO_5Na$ $[M+Na]^+$ 403.0151, found 403.0152.

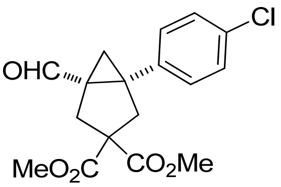

Dimethyl 1-(4-chlorophenyl)-5-formylbicyclo[3.1.0]hexane-3,3-dicarboxylate
2j, 60% yield, light-yellow oil, analytical TLC (silica gel 60), 25% EtOAc in *n*-hexane, R_f = 0.5;
^{1}H NMR (400 MHz, $CDCl_3$) δ 8.55 (s, 1H), 7.31 – 7.24 (m, 4H), 3.79 (s, 3H), 3.76 (s, 3H), 3.18 (d, *J* = 14.4 Hz, 1H), 2.94 (d, *J* = 14.4 Hz, 1H), 2.83 (d, *J* = 14.4 Hz, 1H), 2.78 (d, *J* = 14.4 Hz, 1H), 1.99 (d, *J* = 6.5 Hz, 1H), 1.45 (d, *J* = 6.5 Hz, 1H).
^{13}C NMR (100 MHz, $CDCl_3$) δ 199.01, 172.55, 171.27, 136.89, 133.56, 130.64, 129.03, 57.97, 53.34, 53.26, 45.18, 44.11, 44.00, 34.34, 22.06.
HRMS (ESI) m/z calcd. for $C_{17}H_{17}ClO_5Na$ $[M+Na]^+$ 359.0656, found 359.0657.

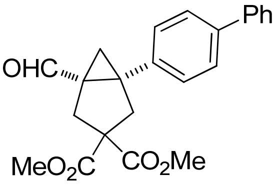

Dimethyl 1-([1,1'-biphenyl]-4-yl)-5-formylbicyclo[3.1.0]hexane-3,3-dicarboxylate
2k, 45% yield, yellow oil, analytical TLC (silica gel 60), 20% EtOAc in *n*-hexane, R_f = 0.55;
^{1}H NMR (400 MHz, $CDCl_3$) δ 8.61 (s, 1H), 7.61 – 7.53 (m, 4H), 7.49 – 7.34 (m, 5H), 3.82 (s, 3H), 3.79 (s, 3H), 3.24 (d, *J* = 14.0 Hz, 1H), 3.01 (d, *J* = 14.4 Hz, 1H), 2.94 (dd, *J* = 14.4, 1.6 Hz, 1H), 2.82 (d, *J* = 14.4 Hz, 1H), 2.08 (d, *J* = 6.4 Hz, 1H), 1.50 (d, *J* = 6.4 Hz, 1H).
^{13}C NMR (125 MHz, $CDCl_3$) δ 199.57, 172.73, 171.39, 140.65, 140.42, 137.33, 129.68, 128.84, 127.55, 127.50, 127.07, 57.95, 53.34, 53.26, 45.21, 44.49, 44.06, 34.33, 22.08.
HRMS (ESI) m/z calcd. for $C_{23}H_{23}O_5$ $[M+H]^+$ 379.1537, found 379.1540.

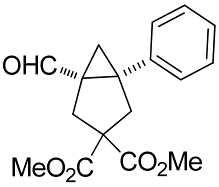

Dimethyl 1-formyl-5-phenylbicyclo[3.1.0]hexane-3,3-dicarboxylate (2l)
2l, 55% yield, colorless oil, analytical TLC (silica gel 60), 20% EtOAc in *n*-hexane, R_f = 0.42;

^{1}**H NMR** (500 MHz, $CDCl_3$) δ 8.54 (s, 1H), 7.36 – 7.31 (m, 4H), 7.30 – 7.26 (m, 1H), 3.80 (s, 3H), 3.77 (s, 3H), 3.20 (dd, *J* = 14.5, 1.0 Hz, 1H), 2.98 (d, *J* = 14.5 Hz, 1H), 2.89 (dd, *J* = 14.5, 1.5 Hz, 1H), 2.80 (d, *J* = 14.5 Hz, 1H), 2.04 (dt, *J* = 6.5, 1.5 Hz, 1H), 1.46 (d, *J* = 6.5 Hz, 1H).
^{13}**C NMR** (125 MHz, $CDCl_3$) δ 199.55, 172.73, 171.38, 138.38, 129.28, 128.84, 127.71, 57.89, 53.31, 53.24, 45.04, 44.74, 44.14, 34.30, 21.97.
HRMS (ESI) m/z calcd. for $C_{17}H_{18}O_5Na$ $[M+Na]^+$ 325.1043, found 325.1046.

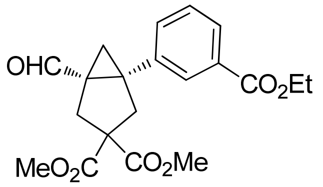

Dimethyl 1-(3-(ethoxycarbonyl)phenyl)-5-formylbicyclo[3.1.0]hexane-3,3-dicarboxylate
2m, 82% yield, yellow solid, analytical TLC (silica gel 60), 25% EtOAc in *n*-hexane, R_f = 0.30;
^{1}**H NMR** (500 MHz, $CDCl_3$) δ 8.56 (s, 1H), 7.99 (s, 1H), 7.95 (d, *J* = 7.7 Hz, 1H), 7.50 (d, *J* = 7.7 Hz, 1H), 7.40 (t, *J* = 7.7 Hz, 1H), 4.39 (q, *J* = 7.1 Hz, 2H), 3.79 (s, 3H), 3.76 (s, 3H), 3.20 (d, *J* = 14.4 Hz, 1H), 2.97 (d, *J* = 14.4 Hz, 1H), 2.87 (d, *J* = 14.5 Hz, 1H), 2.80 (d, *J* = 14.4 Hz, 1H), 2.06 (d, *J* = 6.4 Hz, 1H), 1.48 (d, *J* = 6.5 Hz, 1H), 1.41 (t, *J* = 7.1 Hz, 3H).
^{13}**C NMR** (125 MHz, $CDCl_3$) δ 198.95, 172.53, 171.20, 166.14, 138.76, 133.80, 131.15, 130.21, 128.97, 128.93, 61.23, 58.01, 53.35, 53.28, 45.19, 44.44, 43.90, 34.34, 22.05, 14.35.
HRMS (ESI) m/z calcd. for $C_{20}H_{22}O_7Na$ $[M+Na]^+$ 397.1252, found 397.1258.
HPLC analysis: Chiralcel AS-H, *i*-PrOH/*n*-hexane = 95/5, flow rate 0.8 mL/min. λ = 230 nm, t(major) = 40.79 min, t(minor) = 46.21 min, 93:7 er.

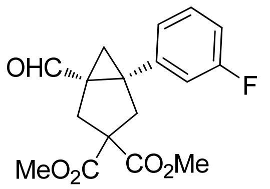

Dimethyl 1-(3-fluorophenyl)-5-formylbicyclo[3.1.0]hexane-3,3-dicarboxylate
2n, 85% yield, colorless oil, analytical TLC (silica gel 60), 20% EtOAc in *n*-hexane, R_f = 0.54;
^{1}**H NMR** (500 MHz, $CDCl_3$) δ 8.57 (s, 1H), 7.33 – 7.28 (m, 1H), 7.11 (ddd, *J* = 7.7, 1.5, 0.9 Hz, 1H), 7.06 – 7.02 (m, 1H), 7.00 – 6.94 (m, 1H), 3.80 (s, 3H), 3.77 (s, 3H), 3.19 (d, *J* = 14.5 Hz, 1H), 2.97 (d, *J* = 14.4 Hz, 1H), 2.87 (dd, *J* = 14.4, 1.5 Hz, 1H), 2.79 (d, *J* = 14.5 Hz, 1H), 2.02 (dt, *J* = 6.4, 1.3 Hz, 1H), 1.46 (d, *J* = 6.5 Hz, 1H).
^{13}**C NMR** (125 MHz, $CDCl_3$) δ 198.95, 172.56, 171.24, 162.83 (d, J_{C-F} = 246.2 Hz), 140.86 (d, J_{C-F} = 7.3 Hz), 130.46 (d, J_{C-F} = 2.9 Hz), 124.93 (d, J_{C-F} = 2.9 Hz), 116.32 (d, J_{C-F} = 21.4 Hz), 114.81 (d, J_{C-F} = 21.4 Hz), 57.98, 53.37, 53.28, 45.36, 44.20 (d, J_{C-F} = 2.0 Hz), 43.91, 34.31, 22.04. ^{19}**F NMR** (376 MHz, $CDCl_3$) δ -112.19. **HRMS** (ESI) m/z calcd. for $C_{17}H_{17}FO_5Na$ $[M+Na]^+$ 343.0948, found 343.0952.
HPLC analysis: Chiralcel AS-H, *i*-PrOH/*n*-hexane = 80/20, flow rate 1.0 mL/min. λ = 230 nm, t(major) = 10.79 min, t(minor) = 13.02 min, 93.5:6.5 er.

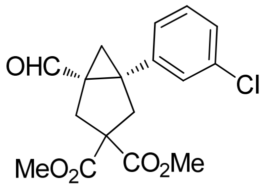

Dimethyl 1-(3-chlorophenyl)-5-formylbicyclo[3.1.0]hexane-3,3-dicarboxylate
2o, 80% yield, colorless oil, analytical TLC (silica gel 60), 25% EtOAc in *n*-hexane, $R_f = 0.45$;
^{1}H NMR (400 MHz, $CDCl_3$) δ 8.58 (s, 1H), 7.35 – 7.17 (m, 4H), 3.80 (s, 3H), 3.77 (s, 3H), 3.19 (d, *J* = 14.4 Hz, 1H), 2.96 (d, *J* = 14.4 Hz, 1H), 2.85 (d, *J* = 14.4 Hz, 1H), 2.79 (d, *J* = 14.4 Hz, 1H), 2.01 (d, *J* = 6.4 Hz, 1H), 1.46 (d, *J* = 6.4 Hz, 1H).
^{13}C NMR (100 MHz, $CDCl_3$) δ 198.85, 172.52, 171.21, 140.43, 134.65, 130.15, 129.43, 128.00, 127.51, 57.97, 53.36, 53.27, 45.27, 44.16, 43.92, 34.32, 21.94.
HRMS (ESI) m/z calcd. for $C_{17}H_{17}ClO_5$ $[M]^+$ 336.0754, found 336.0759.
HPLC analysis: Chiralcel IA, *i*-PrOH/*n*-hexane = 95/5, flow rate 0.8 mL/min. λ = 214 nm, t(minor) = 13.51 min, t(major) = 16.32 min, 90.5:9.5 er.

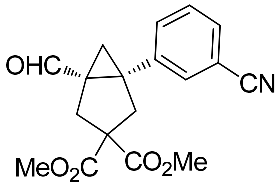

Dimethyl 1-(3-cynophenyl)-5-formylbicyclo[3.1.0]hexane-3,3-dicarboxylate
2p, 82% yield, colorless oil, analytical TLC (silica gel 60), 30% EtOAc in *n*-hexane, $R_f = 0.25$;
^{1}H NMR (400 MHz, $CDCl_3$) δ 8.66 (s, 1H), 7.67 – 7.63 (m, 1H), 7.60 – 7.54 (m, 2H), 7.46 (t, *J* = 7.8 Hz, 1H), 3.81 (s, 3H), 3.79 (s, 3H), 3.22 (d, *J* = 14.4 Hz, 1H), 2.98 (d, *J* = 14.4 Hz, 1H), 2.89 – 2.80 (m, 2H), 2.04 (d, *J* = 6.5 Hz, 1H), 1.51 (d, *J* = 6.5 Hz, 1H).
^{13}C NMR (100 MHz, $CDCl_3$) δ 198.12, 172.29, 171.11, 140.01, 133.82, 132.84, 131.39, 129.77, 118.21, 113.20, 58.33, 53.39, 53.29, 45.48, 44.22, 43.93, 34.60, 22.10.
HRMS (ESI) m/z calcd. for $C_{18}H_{17}NO_5Na$ $[M+Na]^+$ 350.0997, found 350.0999.
HPLC analysis: Chiralcel AD-H, *i*-PrOH/*n*-hexane = 80/20, flow rate 1.0 mL/min. λ = 214 nm, t(minor) = 10.2 min, t(major) = 11.93 min, 95:5 er.

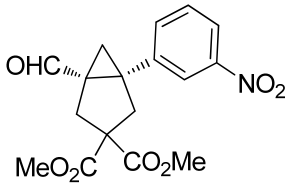

Dimethyl 1-(3-nitrophenyl)-5-formylbicyclo[3.1.0]hexane-3,3-dicarboxylate
2q, 90% yield, colorless oil, analytical TLC (silica gel 60), 30% EtOAc in *n*-hexane, $R_f = 0.2$;
^{1}H NMR (500 MHz, $CDCl_3$) δ 8.69 (s, 1H), 8.20 (t, *J* = 1.8 Hz, 1H), 8.15 (dd, *J* = 8.2, 1.3 Hz, 1H), 7.66 (d, *J* = 7.7 Hz, 1H), 7.53 (t, *J* = 7.9 Hz, 1H), 3.81 (s, 3H), 3.79 (s, 3H), 3.23 (d, *J* = 14.5 Hz, 1H), 3.00 (d, *J* = 14.5 Hz, 1H), 2.90 (dd, *J* = 14.5, 1.4 Hz, 1H), 2.84 (d, *J* = 14.5 Hz, 1H), 2.11 (d, *J* = 6.5 Hz, 1H), 1.54 (d, *J* = 6.5 Hz, 1H).
^{13}C NMR (125 MHz, $CDCl_3$) δ 198.21, 172.28, 171.09, 148.48, 140.49, 135.55, 129.96, 124.22, 122.85, 58.36, 53.48, 53.38, 45.67, 44.34, 43.82, 34.59, 22.36.
HRMS (ESI) m/z calcd. for $C_{17}H_{17}NO_7Na$ $[M+Na]^+$ 370.0892, found 370.0897.

HPLC analysis: Chiralcel OD-H, *i*-PrOH/*n*-hexane = 80/20, flow rate 1.0 mL/min. λ = 254 nm, t(major) = 16.26 min, t(minor) = 18.21 min, 92:8 er.

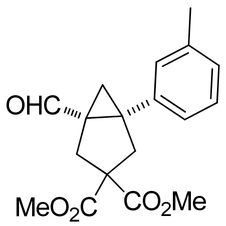

Dimethyl 1-(3-methylphenyl)-5-formylbicyclo[3.1.0]hexane-3,3-dicarboxylate
2r, 42% yield, colorless oil, analytical TLC (silica gel 60), 20% EtOAc in *n*-hexane, R_f = 0.42;
^{1}**H NMR** (400 MHz, $CDCl_3$) δ 8.54 (s, 1H), 7.22 (t, *J* = 7.5 Hz, 1H), 7.14 – 7.08 (m, 3H), 3.80 (s, 3H), 3.77 (s, 3H), 3.20 (dd, *J* = 14.4, 1.0 Hz, 1H), 2.96 (d, *J* = 14.4 Hz, 1H), 2.88 (dd, *J* = 14.4, 1.5 Hz, 1H), 2.79 (d, *J* = 14.4 Hz, 1H), 2.35 (s, 3H), 2.01 (d, *J* = 6.4 Hz, 1H), 1.44 (d, *J* = 6.4 Hz, 1H). ^{13}**C NMR** (125 MHz, $CDCl_3$) δ 199.64, 172.75, 171.41, 138.56, 138.32, 129.94, 128.70, 128.47, 126.29, 57.86, 53.26, 53.19, 44.98, 44.68, 44.16, 34.30, 21.94, 21.35.
HRMS (ESI) m/z calcd. for $C_{18}H_{22}O_5Na$ $[M+Na]^+$ 339.1200, found 339.1203.

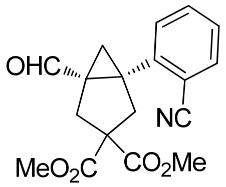

Dimethyl 1-(2-cynophenyl)-5-formylbicyclo[3.1.0]hexane-3,3-dicarboxylate
2s, 70% yield, colorless oil, analytical TLC (silica gel 60), 30% EtOAc in *n*-hexane, R_f = 0.25;
^{1}**H NMR** (400 MHz, $CDCl_3$) δ 9.05 (s, 1H), 7.64 (dd, *J* = 7.7, 0.8 Hz, 1H), 7.57 (td, *J* = 7.7, 1.2 Hz, 1H), 7.42 – 7.37 (m, 2H), 3.80 (s, 3H), 3.76 (s, 3H), 3.34 (d, *J* = 14.2 Hz, 1H), 2.99 – 2.89 (m, 3H), 1.99 (d, *J* = 6.3 Hz, 1H), 1.58 (d, *J* = 6.3 Hz, 1H). ^{13}**C NMR** (100 MHz, $CDCl_3$) δ 197.84, 172.44, 170.64, 142.76, 133.34, 133.19, 131.31, 128.33, 117.52, 113.75, 58.50, 53.35, 45.33, 44.26, 42.80, 34.80, 22.86.
HRMS (ESI) m/z calcd. for $C_{18}H_{17}NO_5Na$ $[M+Na]^+$ 350.0992, found 350.0999.
HPLC analysis: Chiralcel AD-H, *i*-PrOH/*n*-hexane = 80/20, flow rate 1.0 mL/min. λ = 210 nm, t(minor) = 10.28 min, t(major) = 15.0 min, 93:7 er.

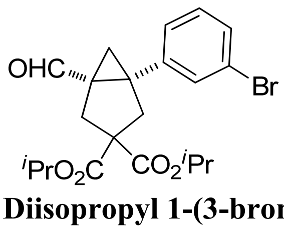

Diisopropyl 1-(3-bromophenyl)-5-formylbicyclo[3.1.0]hexane-3,3-dicarboxylate
2t, 81% yield, colorless oil, analytical TLC (silica gel 60), 20% EtOAc in *n*-hexane, R_f = 0.4;
^{1}**H NMR** (500 MHz, $CDCl_3$) δ 8.59 (s, 1H), 7.50 (s, 1H), 7.41 (d, *J* = 7.9 Hz, 1H), 7.27 (d, *J* = 7.8 Hz, 1H), 7.21 (t, *J* = 7.8 Hz, 1H), 5.12 – 5.04 (m, 2H), 3.14 (d, *J* = 14.4 Hz, 1H), 2.90 (d, *J* = 14.4 Hz, 1H), 2.82 (d, *J* = 14.4 Hz, 1H), 2.74 (d, *J* = 14.5 Hz, 1H), 2.00 (d, *J* = 6.3 Hz, 1H), 1.51 (d, *J* = 6.3 Hz, 1H), 1.28 (d, *J* = 6.0 Hz, 3H), 1.27 (d, *J* = 6.0 Hz, 6H), 1.24 (d, *J* = 6.3 Hz, 3H).

^{13}C NMR (100 MHz, $CDCl_3$) δ 199.07, 171.59, 170.28, 140.96, 132.32, 130.85, 130.38, 128.04, 122.79, 69.82, 69.62, 58.31, 45.53, 44.33, 43.88, 34.11, 22.21, 21.52, 21.50.
HRMS (ESI) m/z calcd. for $C_{21}H_{25}O_5BrNa$ $[M+Na]^+$ 459.0771, found 459.0778.

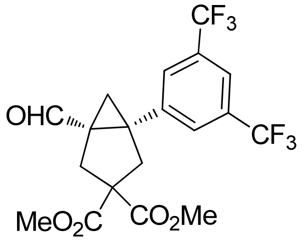

Dimethyl 1-(3,5-bis(trifluoromethyl)phenyl)-5-formylbicyclo[3.1.0]hexane-3,3-dicarboxylate. 2u, 72% yield, white solid, analytical TLC (silica gel 60), 15% EtOAc in *n*-hexane, R_f = 0.40; **^{1}H NMR** (400 MHz, $CDCl_3$) δ 8.73 (s, 1H), 7.80 (s, 1H), 7.76 (s, 2H), 3.81 (s, 3H), 3.79 (s, 3H), 3.26 (dd, *J* = 14.5, 0.7 Hz, 1H), 3.01 (d, *J* = 14.5 Hz, 1H), 2.89 – 2.82 (m, 2H), 2.09 (d, *J* = 6.5 Hz, 1H), 1.56 (d, *J* = 6.5 Hz, 1H). **^{13}C NMR** (100 MHz, $CDCl_3$) δ 197.71, 172.15, 170.99, 141.04, 132.32 (q, $J_{C\text{-}F}$ = 33.4 Hz, 1C), 129.52, 123.35 (q, $J_{C\text{-}F}$ = 271.2 Hz, 1C), 121.86 (m, 1C), 58.47, 53.45, 53.36, 45.74, 44.19, 43.83, 34.64, 22.47. **^{19}F NMR** (376 MHz, $CDCl_3$) δ -62.87.
HRMS (ESI) m/z calcd. for $C_{19}H_{16}F_6O_5Na$ $[M+Na]^+$ 461.0791, found 461.0794.
HPLC analysis: Chiralcel AS-H, *i*-PrOH/*n*-hexane = 95/5, flow rate 0.8 mL/min. λ = 230 nm, t(minor) = 7.29 min, t(major) = 8.27 min, 89.5:10.5 er.

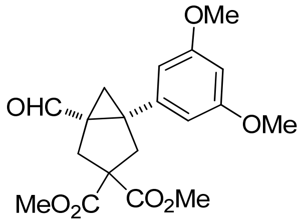

Dimethyl 1-(3,5-dimethoxyphenyl)-5-formylbicyclo[3.1.0]hexane-3,3-dicarboxylate
2v, 62% yield, yellowish oil, analytical TLC (silica gel 60), 30 % EtOAc in *n*-hexane, R_f = 0.28; **^{1}H NMR** (500 MHz, $CDCl_3$) δ 8.56 (s, 1H), 6.46 (d, *J* = 2.2 Hz, 2H), 6.37 (t, *J* = 2.2 Hz, 1H), 3.80 (s, 3H), 3.79 (s, 6H), 3.77 (s, 3H), 3.18 (d, *J* = 14.0 Hz, 1H), 2.96 (d, *J* = 14.4 Hz, 1H), 2.87 (dd, *J* = 14.4, 1.2 Hz, 1H), 2.76 (d, *J* = 14.5 Hz, 1H), 2.00 (d, *J* = 6.4 Hz, 1H), 1.42 (d, *J* = 6.4 Hz, 1H). **^{13}C NMR** (125 MHz, $CDCl_3$) δ 199.40, 172.69, 171.34, 161.04, 140.74, 107.35, 99.51, 57.93, 55.40, 53.27, 53.21, 45.17, 44.69, 43.97, 34.25, 22.24.
HRMS (ESI) m/z calcd. for $C_{19}H_{23}O_7$ $[M+H]^+$ 363.1436, found 363.1438.

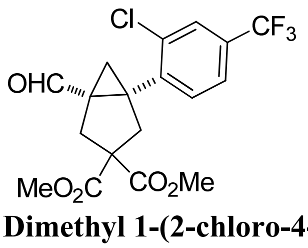

Dimethyl 1-(2-chloro-4-(trifluoromethyl)phenyl)-5-formylbicyclo[3.1.0]hexane-3,3-dicarboxylate

2w, 70% yield, colorless oil, analytical TLC (silica gel 60), 20% EtOAc in *n*-hexane, R_f = 0.45; **^{1}H NMR** (500 MHz, $CDCl_3$) δ 8.91 (s, 1H), 7.64 (s, 1H), 7.54 (s, 2H), 3.81 (s, 3H), 3.77 (s, 3H), 3.33 (d, *J* = 14.3 Hz, 1H), 2.96 – 2.81 (m, 3H), 1.88 (d, *J* = 6.1 Hz, 1H), 1.54 (d, *J* = 6.0 Hz, 1H). **^{13}C NMR** (125 MHz, $CDCl_3$) δ 197.45, 172.69, 170.84, 140.15, 135.53, 132.83, 131.69 (q, J_{C-F} = 33 Hz), 127.09 (q, J_{C-F} = 3.7 Hz), 124.14 (q, J_{C-F} = 3.6 Hz), 123.23 (q, J_{C-F} = 270.5 Hz), 58.28, 53.37, 53.36, 45.18, 43.40, 40.60, 34.61.
^{19}F NMR (376 MHz, $CDCl_3$) δ -62.85. **HRMS** (ESI) m/z calcd. for $C_{18}H_{16}ClF_3O_5Na$ $[M+Na]^+$ 427.0529, found 427.0531.

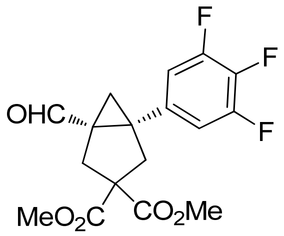

Dimethyl 1-formyl-5-(3,4,5-trifluorophenyl)bicyclo[3.1.0]hexane-3,3-dicarboxylate
2x, 80% yield, white solid, analytical TLC (silica gel 60), 20% EtOAc in *n*-hexane, R_f = 0.48; **^{1}H NMR** (400 MHz, $CDCl_3$) δ 8.68 (s, 1H), 6.98 – 6.93 (m, 2H), 3.80 (s, 3H), 3.78 (s, 3H), 3.18 (d, *J* = 14.5 Hz, 1H), 2.95 (d, *J* = 14.5 Hz, 1H), 2.85 – 2.76 (m, 2H), 2.02 – 1.96 (m, 1H), 1.46 (d, *J* = 6.5 Hz, 1H); **^{13}C NMR** (125 MHz, $CDCl_3$) δ 198.19, 172.29, 171.10, 151.26 (ddd, J_{C-F} = 250.1, 9.9, 4.1 Hz, 1C), 139.25 (td, J_{C-F} = 251.3, 15.3 Hz, 1C), 134.71–134.56 (m, 1C), 113.52 (dd, *J* = 16.3, 5.0 Hz, 1C), 58.17, 53.46, 53.35, 45.69, 43.88, 43.76, 34.45, 22.38; **^{19}F NMR** (376 MHz, $CDCl_3$) δ -132.79 (d, *J* = 20.7 Hz, 2F), -160.52 (t, *J* = 20.7 Hz, F); **HRMS** (ESI) m/z calcd. for $C_{17}H_{17}F_3O_5Na$ $[M+Na]^+$ 379.0763, found 379.0764.
HPLC analysis: Chiralcel AS-H, *i*-PrOH/*n*-hexane = 80/20, flow rate 1.0 mL/min. λ = 230 nm, t(minor) = 7.34 min, t(major) = 8.47 min, 92.5:7.5 er.

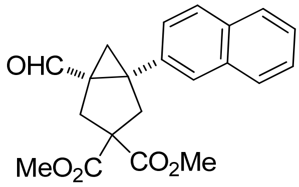

Dimethyl 1-formyl-5-(naphthalen-2-yl)bicyclo[3.1.0]hexane-3,3-dicarboxylate
2y, 50% yield, colorless oil, analytical TLC (silica gel 60), 20% EtOAc in *n*-hexane, R_f = 0.4; **^{1}H NMR** (400 MHz, $CDCl_3$) δ 8.55 (s, 1H), 7.84 – 7.81 (m, 4H), 7.55 – 7.46 (m, 2H), 7.43 (dd, *J* = 8.5, 1.7 Hz, 1H), 3.83 (s, 3H), 3.79 (s, 3H), 3.27 (d, *J* = 14.4 Hz, 1H), 3.06 – 2.97 (m, 2H), 2.85 (d, *J* = 14.5 Hz, 1H), 2.17 (d, *J* = 6.4 Hz, 1H), 1.55 (d, *J* = 6.4 Hz, 1H).
^{13}C NMR (100 MHz, $CDCl_3$) δ 199.41, 172.74, 171.42, 135.80, 133.28, 132.71, 128.78, 128.18, 127.74, 127.72, 126.93, 126.55, 126.32, 58.02, 53.33, 53.25, 45.29, 44.91, 44.00, 34.40, 22.16.
HRMS (ESI) m/z calcd. for $C_{21}H_{20}O_5Na$ $[M+Na]^+$ 375.1200, found 375.1202.

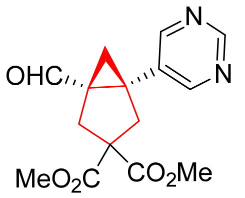

Dimethyl-1-formyl-5-(pyrimidin-5-yl)bicyclo[3.1.0]hexane-3,3-dicarboxylate
2z, 66% yield, yellowish solid, analytical TLC (silica gel 60), 25% EtOAc in *n*-hexane, R_f = 0.2; **^{1}H NMR** (400 MHz, $CDCl_3$) δ 9.15 (s, 1H), 8.87 (s, 1H), 8.71 (s, 2H), 3.81 (s, 3H), 3.79 (s, 3H), 3.25 (dd, *J* = 14.4, 1.2 Hz, 1H), 2.99 (d, *J* = 14.4 Hz, 1H), 2.90 – 2.84 (m, 2H), 2.07 (dt, *J* = 6.4, 1.6 Hz, 1H), 1.54 (d, *J* = 6.4 Hz, 1H). **^{13}C NMR** (100 MHz, $CDCl_3$) δ 197.53, 172.05, 170.93, 157.81, 157.73, 132.14, 132.04, 132.02, 128.56, 58.69, 53.52, 53.40, 44.98, 43.40, 40.53, 34.80, 21.83. **HRMS** (ESI) m/z calcd. for $C_{15}H_{17}O_5N_2$ $[M+H]^+$ 305.1129, found 305.1132.
HPLC condition: Chiralcel OD-H, *i*-PrOH/*n*-hexane = 80/20, flow rate 1.0 mL/min. λ = 210 nm, t(major) = 36.43 min, t(minor) = 39.51 min, 94.5:5.5 er.

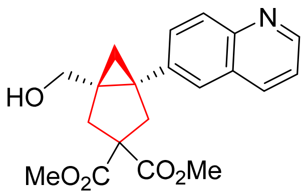

Dimethyl-1-(hydroxymethyl)-5-(quinolin-6-yl)bicyclo[3.1.0]hexane-3,3-dicarboxylate

2aa-OH, **2aa** was reduced to its alcohol product to confirm its NMR structure, while HPLC was measured for **2aa**. 55% yield for two steps, yellowish oil, analytical TLC (silica gel 60), 30% EtOAc in *n*-hexane, R_f = 0.2; **^{1}H NMR** (400 MHz, $CDCl_3$) δ 8.90 (dd, *J* = 4.0, 1.2 Hz, 1H), 8.13 (d, *J* = 7.6 Hz, 1H), 8.07 (d, *J* = 9.2 Hz, 1H), 7.76 (dd, *J* = 7.2, 2.0 Hz, 2H), 7.41 (dd, *J* = 8.4, 4.4 Hz, 1H), 3.81 (s, 3H), 3.79 (s, 3H), 3.50 (d, *J* = 12.0 Hz, 1H), 3.34 (d, *J* = 12.0 Hz, 1H), 3.02 2.86 (m, 3H), 1.73 (s, 1H), 1.19 (d, *J* = 6.2 Hz, 1H), 0.90 (d, *J* = 6.2 Hz, 1H).
^{13}C NMR (125 MHz, $CDCl_3$) δ 173.04, 172.32, 150.29, 147.33, 138.84, 135.87, 131.09, 129.63, 128.26, 127.91, 121.40, 65.06, 58.59, 53.26, 53.09, 44.42, 38.60, 37.76, 36.93, 18.18.
HRMS (ESI) m/z calcd. for $C_{20}H_{22}NO_5$ $[M+H]^+$ 356.1495, found 356.1492.
HPLC condition for 2aa: Chiralcel AS-H, *i*-PrOH/*n*-hexane = 80/20, flow rate 1.0 mL/min. λ = 230 nm, t(major) = 23.94 min, t(minor) = 28.15 min, 94.9:5.1 er.

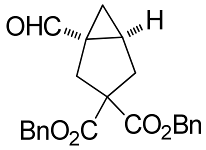

Dibenzyl 1-formylbicyclo[3.1.0]hexane-3,3-dicarboxylate
2ab, 55% yield, colorless oil, analytical TLC (silica gel 60), 20% EtOAc in *n*-hexane, R_f = 0.32; **^{1}H NMR** (400 MHz, $CDCl_3$) δ 8.90 (s, 1H), 7.34 –7.30 (m, 6H), 7.27 – 7.24 (m, 4H), 5.11 (d, *J*

= 6.4 Hz, 4H), 2.99 (dd, J = 14.3, 1.0 Hz, 1H), 2.67 – 2.54 (m, 3H), 2.08 – 2.03 (m, 1H), 1.46 – 1.41 (m, 1H), 1.04 (t, J = 5.8 Hz, 1H).
^{13}C NMR (100 MHz, $CDCl_3$) δ 198.74, 171.84, 170.50, 135.15, 135.09, 128.59, 128.46, 128.42, 128.20, 128.06, 67.63, 60.53, 41.82, 34.87, 33.18, 28.25, 19.19.
HRMS (ESI) m/z calcd. for $C_{23}H_{22}O_5Na$ $[M+Na]^+$ 401.1355, found 401.1359.

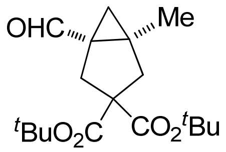

Di-tert-butyl 1-formyl-5-methylbicyclo[3.1.0]hexane-3,3-dicarboxylate
2ac, 60% yield, yellow solid, analytical TLC (silica gel 60), 20% EtOAc in *n*-hexane, R_f = 0.38; **^{1}H NMR** (400 MHz, $CDCl_3$) δ 9.21 (s, 1H), 2.87 (dd, J = 14.1, 1.2 Hz, 1H), 2.56 (d, J = 6.3 Hz, 1H), 2.53 (d, J = 6.3 Hz, 1H), 2.39 (dd, J = 14.0, 1.2 Hz, 1H), 1.45 (d, J = 3.6 Hz, 19H), 1.37 (s, 3H), 1.16 (d, J = 5.8 Hz, 1H). **^{13}C NMR** (100 MHz, $CDCl_3$) δ 200.72, 171.30, 170.17, 81.89, 81.61, 59.78, 43.88, 41.92, 38.93, 34.58, 27.77, 25.64, 18.43.
HRMS (ESI) m/z calcd. for $C_{18}H_{28}O_5Na$ $[M+Na]^+$ 347.1827, found 347.1829.

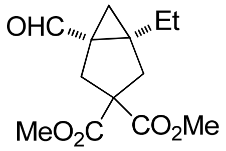

Dimethyl 1-ethyl-5-formylbicyclo[3.1.0]hexane-3,3-dicarboxylate
2ad, 66% yield, colorless oil, analytical TLC (silica gel 60), 20 % EtOAc in *n*-hexane, R_f = 0.34; **^{1}H NMR** (500 MHz, $CDCl_3$) δ 9.22 (s, 1H), 3.75 (s, 3H), 3.74 (s, 3H), 3.00 (d, J = 14.2 Hz, 1H), 2.66 (dd, J = 14.1, 9.8 Hz, 2H), 2.54 (dd, J = 14.0, 1.2 Hz, 1H), 1.66 (tt, J = 14.4, 7.0 Hz, 2H), 1.49 (d, J = 6.0 Hz, 1H), 1.09 (d, J = 6.0 Hz, 1H), 1.00 (t, J = 7.4 Hz, 3H).
^{13}C NMR (125 MHz, $CDCl_3$) δ 200.21, 172.65, 171.59, 58.13, 53.20, 53.11, 44.48, 44.03, 39.40, 35.15, 25.82, 25.05, 12.06. **HRMS** (ESI) m/z calcd. for $C_{13}H_{19}O_5$ $[M+H]^+$ 255.1224, found 255.1227.

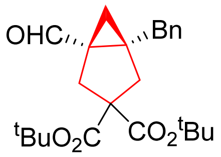

Di-tert-butyl-1-benzyl-5-formylbicyclo[3.1.0]hexane-3,3-dicarboxylate
2ae, 62% yield, colorless oil, analytical TLC (silica gel 60), 15 % EtOAc in *n*-hexane, R_f = 0.3; **^{1}H NMR** (500 MHz, $CDCl_3$) δ 9.41 (s, 1H), 7.33 (t, J = 7.4 Hz, 2H), 7.27 – 7.19 (m, 3H), 3.03 – 2.90 (m, 3H), 2.60 (d, J = 14.0 Hz, 1H), 1.73 (d, J = 5.5 Hz, 1H), 1.47 – 1.37 (m, 18H), 1.29 (d, J = 5.5 Hz, 1H). **^{13}C NMR** (100 MHz, $CDCl_3$) δ 200.45, 171.21, 170.12, 138.87, 128.72, 128.58, 126.51, 81.96, 81.69, 59.99, 43.96, 43.82, 40.02, 38.48, 35.18, 27.76, 25.33. **HRMS** (ESI) m/z calcd. for $C_{24}H_{32}O_5Na$ [M+ Na]$^+$ 423.2140, found 423.2142.

HPLC condition: Chiralcel AS-H, *i*-PrOH/*n*-hexane = 97/3, flow rate 0.6 mL/min. λ = 214 nm, t(major) = 11.07 min, t(minor) = 13.54 min, 91.6:8.4 er.

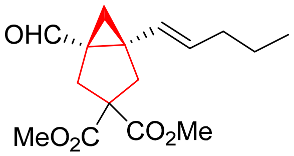

Dimethyl-1-formyl-5-((E)-pent-1-en-1-yl)bicyclo[3.1.0]hexane-3,3-dicarboxylate
2af, 55% yield, colorless oil, analytical TLC (silica gel 60), 20 % EtOAc in *n*-hexane, R_f = 0.4; **^{1}H NMR** (400 MHz, $CDCl_3$) δ 9.04 (s, 1H), 5.68 (dt, *J* = 15.6, 6.8 Hz, 1H), 5.48 (d, *J* = 15.6 Hz, 1H), 3.77 (s, 3H), 3.75 (s, 3H), 2.99 (dd, *J* = 14.4, 1.2 Hz, 1H), 2.76 (dd, *J* = 14.4, 1.2 Hz, 1H), 2.71 (d, *J* = 12.0 Hz, 1H), 2.67 (d, *J* = 12.0 Hz, 1H), 2.08 – 1.97 (m, 2H), 1.83 (d, *J* = 6.4 Hz, 1H), 1.39 (q, *J* = 7.2 Hz, 2H), 1.25 (d, *J* = 6.4 Hz, 1H), 0.89 (t, *J* = 6.4 Hz, 3H). **^{13}C NMR** (100 MHz, $CDCl_3$) δ 199.85, 172.59, 171.40, 132.98, 127.53, 58.00, 53.23, 53.12, 45.64, 43.28, 39.47, 34.49, 34.24, 23.10, 22.40, 13.61. **HRMS** (ESI) m/z calcd. for $C_{16}H_{23}O_5$ $[M+H]^+$ 295.1539, found 295.1540.

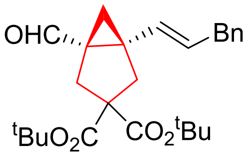

Di-tert-butyl-1-formyl-5-((E)-3-phenylprop-1-en-1-yl)bicyclo[3.1.0]hexane-3,3dicarboxylate
2ag, 60% yield, colorless oil, analytical TLC (silica gel 60), 20 % EtOAc in *n*-hexane, R_f = 0.44; **^{1}H NMR** (400 MHz, $CDCl_3$) δ 9.07 (s, 1H), 7.34 – 7.29 (m, 2H), 7.25 – 7.20 (m, 1H), 7.19 – 7.13 (m, 2H), 5.84 (dt, *J* = 15.2, 6.8 Hz, 1H), 5.57 (dt, *J* = 15.3, 1.2 Hz, 1H), 3.38 (d, *J* = 7.2 Hz, 2H), 2.85 (dd, *J* = 14.0, 1.2 Hz, 1H), 2.72 (dd, *J* = 14.0, 1.6 Hz, 1H), 2.61 (d, *J* = 14.0 Hz, 1H), 2.52 (d, *J* = 14.0 Hz, 1H), 1.81 (dt, *J* = 6.0, 1.2 Hz, 1H), 1.46 (s, 9H), 1.45 (s, 9H), 1.35 (d, *J* = 6.4 Hz, 1H). **^{13}C NMR** (100 MHz, $CDCl_3$) δ 200.03, 171.23, 169.92, 139.90, 131.17, 129.23, 128.52, 126.21, 82.05, 81.77, 59.59, 46.14, 43.46, 38.89, 33.92, 27.78, 23.58. **HRMS** (ESI) m/z calcd. for $C_{26}H_{34}O_5Na$ $[M+Na]^+$ 449.2293, found 449.2298.

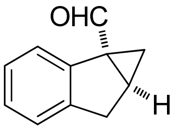

1,1a,6,6a-tetrahydrocyclopropa[a]indene-1a-carbaldehyde
2ah, 50% yield, colorless oil, analytical TLC (silica gel 60), 20 % EtOAc in *n*-hexane, R_f = 0.42; **^{1}H NMR** (400 MHz, $CDCl_3$) δ 9.48 (s, 1H), 7.68 (d, *J* = 7.1 Hz, 1H), 7.26 – 7.17 (m, 3H), 3.31 (dd, *J* = 16.9, 6.9 Hz, 1H), 3.03 (d, *J* = 16.9 Hz, 1H), 2.64 – 2.59 (m, 1H), 2.10 (dd, *J* = 8.6, 4.6 Hz, 1H), 1.04 (t, *J* = 5.2 Hz, 1H). **^{13}C NMR** (101 MHz, $CDCl_3$) δ 198.44, 142.35, 140.59, 126.91, 126.52, 125.68, 124.58, 46.31, 34.14, 29.83, 26.38.
HRMS (ESI) m/z calcd. for $C_{11}H_{11}O$ $[M+H]^+$ 159.0803, found 159.0804.

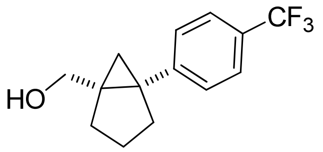

(5-(4-(trifluoromethyl)phenyl)bicyclo[3.1.0]hexan-1-yl)methanol

2ai, 45% yield for two steps, colorless oil, analytical TLC (silica gel 60), 20% EtOAc in *n*-hexane, R_f = 0.2; **^{1}H NMR** (500 MHz, $CDCl_3$) δ 7.57 (d, *J* = 8.0 Hz, 2H), 7.44 (d, *J* = 8.0 Hz, 2H), 3.42 (d, *J* = 1.0 Hz, 2H), 2.19 – 2.09 (m, 2H), 2.06 (dd, *J* = 12.5, 8.0 Hz, 1H), 2.00 (dd, *J* = 12.5, 8.0 Hz, 1H), 1.81 (dt, *J* = 13.5, 8.0 Hz, 1H), 1.42 – 1.33 (m, 1H), 1.03 (d, *J* = 5.5 Hz, 1H), 0.96 (d, *J* = 5.5 Hz, 1H). **^{13}C NMR** (125 MHz, $CDCl_3$) δ 146.60, 129.45, 128.42 (q, $J_{C\text{-}F}$ = 32.2 Hz), 125.27 (q, $J_{C\text{-}F}$ = 3.7 Hz), 124.27 (q, $J_{C\text{-}F}$ = 270.2 Hz), 65.95, 37.48, 36.84, 36.53, 30.32, 20.71, 14.98. **^{19}F NMR** (376 MHz, $CDCl_3$) δ -62.36; **HRMS** (ESI) m/z calcd. for $C_{14}H_{14}F_3$ $[M-H_2O]^+$ 239.1039, found 239.1042.

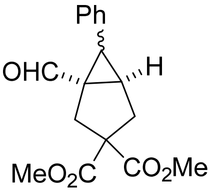

Dimethyl-1-formyl-6-phenylbicyclo[3.1.0]hexane-3,3-dicarboxylate

2aj, **^{1}H NMR** (400 MHz, $CDCl_3$) δ 9.21 (s, 1H), 7.39 – 7.36 (m, 2H), 7.32 – 7.29 (m, 1H), 7.17 (d, *J* = 8.0 Hz, 2H), 3.73 (s, 3H), 3.39 (s, 3H), 3.26 (d, *J* = 9.2 Hz, 1H), 3.18 (d, *J* = 14.8 Hz, 1H), 2.77 – 2.69 (m, 1H), 2.66 – 2.58 (m, 1H), 2.31 (dd, *J* = 14.3, 2.9 Hz, 1H), 2.22 (d, *J* = 14.9 Hz, 1H). **^{13}C NMR** (100 MHz, $CDCl_3$) δ 199.25, 172.30, 169.64, 131.97, 131.06, 128.89, 127.56, 67.34, 53.15, 52.51, 48.92, 39.42, 36.05, 32.19, 31.00.

2ag', **^{1}H NMR** (400 MHz, $CDCl_3$) δ 8.72 (s, 1H), 7.35 – 7.24 (m, 5H), 3.78 (s, 3H), 3.75 (s, 3H), 3.09 (d, *J* = 14.8 Hz, 1H), 2.91 – 2.84 (m, 2H), 2.76 (d, *J* = 4.8 Hz, 1H), 2.71 (d, *J* = 14.8 Hz, 1H), 2.67 – 2.62 (m, 1H). **^{13}C NMR** (100 MHz, $CDCl_3$) δ 198.80, 172.59, 171.44, 134.52, 128.99, 128.70, 127.35, 61.28, 53.21, 53.15, 47.90, 38.96, 35.67, 34.57, 32.71.

HRMS (ESI) m/z calcd. for $C_{17}H_{19}O_5$ $[M+H]^+$ 303.1225, found 303.1227.

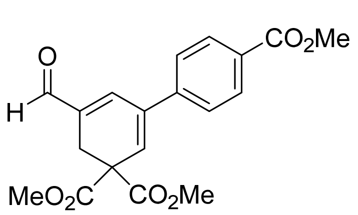

Trimethyl 5-formyl-[1,1'-biphenyl]-3,3,4'(4H)-tricarboxylate

3a, **^{1}H NMR** (500 MHz, $CDCl_3$) δ 9.68 (s, 1H), 8.10 (d, *J* = 8.4 Hz, 2H), 7.56 (d, *J* = 8.4 Hz, 2H), 7.15 (d, *J* = 1.0 Hz, 1H), 6.71 (d, *J* = 1.0 Hz, 1H), 3.96 (s, 3H), 3.80 (s, 6H), 3.20 (d, *J* = 1.5 Hz, 2H). **^{13}C NMR** (125 MHz, $CDCl_3$) δ 191.53, 169.57, 166.63, 141.88, 140.27, 137.72, 136.54, 130.16, 128.54, 126.00, 124.72, 55.05, 53.48, 52.32, 25.42. **HRMS** (ESI) m/z calcd. for $C_{19}H_{19}O_7$ $[M+H]^+$ 359.1124, found 359.1125.

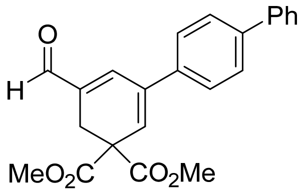

Dimethyl 5-formyl-[1,1':4',1''-terphenyl]-3,3(4H)-dicarboxylate

3k, ^{1}H NMR (500 MHz, $CDCl_3$) δ 9.70 (s, 1H), 7.68 (d, *J* = 8.5 Hz, 2H), 7.66 – 7.63 (m, 2H), 7.58 (d, *J* = 8.5 Hz, 2H), 7.49 (t, *J* = 8.0 Hz, 2H), 7.41 (d, *J* = 6.0 Hz, 1H), 7.21 (d, *J* = 1.0 Hz, 1H), 6.69 (d, *J* = 1.0 Hz, 1H), 3.80 (s, 6H), 3.21 (d, *J* = 1.5 Hz, 2H). **^{13}C NMR** (125 MHz, $CDCl_3$) δ 191.62, 169.82, 141.52, 140.99, 140.27, 137.50, 136.77, 136.42, 128.92, 127.69, 127.54, 127.06, 126.81, 126.39, 55.06, 53.39, 25.51. **HRMS** (ESI) m/z calcd. for $C_{23}H_{21}O_5$ $[M+H]^+$ 377.1383, found 377.1384.

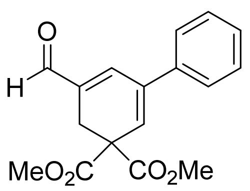

Dimethyl 5-formyl-[1,1'-biphenyl]-3,3(4H)-dicarboxylate

3l, ^{1}H NMR (500 MHz, $CDCl_3$) δ 9.67 (s, 1H), 7.50 – 7.49 (m, 2H), 7.46 – 7.40 (m, 3H), 7.16 (q, *J* = 1.5 Hz, 1H), 6.63 (d, *J* = 1.3 Hz, 1H), 3.79 (s, 6H), 3.20 (d, *J* = 2.0 Hz, 2H). **^{13}C NMR** (125 MHz, $CDCl_3$) δ 191.61, 169.82, 141.17, 137.62, 137.43, 137.24, 128.87, 128.65, 126.96, 125.99, 55.03, 53.36, 25.48. **HRMS** (ESI) m/z calcd. for $C_{17}H_{17}O_5$ $[M+H]^+$ 301.1069, found 301.1071.

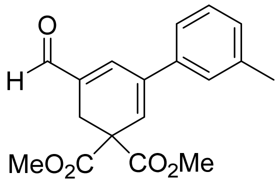

Dimethyl 5-formyl-3'-methyl-[1,1'-biphenyl]-3,3(4H)-dicarboxylate

3r, ^{1}H NMR (500 MHz, $CDCl_3$) δ 9.65 (s, 1H), 7.31 – 7.27 (m, 3H), 7.20 (d, 1H), 7.13 (d, *J* = 7.0 Hz, 1H), 6.59 (d, *J* = 1.5 Hz, 1H), 3.77 (s, 7H), 3.17 (d, *J* = 1.6 Hz, 2H), 2.40 (s, 3H). **^{13}C NMR** (125 MHz, $CDCl_3$) δ 191.65, 169.86, 141.37, 138.60, 137.58, 137.33, 137.31, 129.40, 128.76, 126.78, 126.67, 123.07, 77.29, 77.03, 76.78, 55.02, 53.35, 25.48, 21.48. **HRMS** (ESI) m/z calcd. for $C_{18}H_{19}O_5$ $[M+H]^+$ 315.1224, found 315.1227.

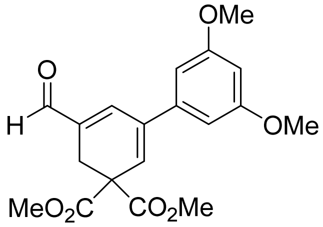

Dimethyl 5-formyl-3',5'-dimethoxy-[1,1'-biphenyl]-3,3(4H)-dicarboxylate

3v, ^{1}H NMR (500 MHz, $CDCl_3$) δ 9.66 (s, 1H), 7.10 (d, *J* = 1.5 Hz, 1H), 6.62 – 6.60 (m, 3H), 6.50 (s, 1H), 3.86 (s, 6H), 3.79 (s, 6H), 3.18 (d, *J* = 1.5 Hz, 2H). **^{13}C NMR** (125 MHz, $CDCl_3$) δ 191.60, 169.78, 161.15, 141.16, 139.75, 137.33, 137.27, 127.19, 104.37, 100.34, 55.53, 54.99, 53.38, 25.49. **HRMS** (ESI) m/z calcd. for $C_{19}H_{21}O_7$ $[M+H]^+$ 361.1284, found 361.1282.

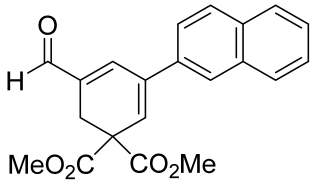

Dimethyl 5-formyl-3-(naphthalen-2-yl)cyclohexa-2,4-diene-1,1-dicarboxylate

3y, ^{1}H NMR (500 MHz, $CDCl_3$) δ 9.73 (s, 1H), 7.94 – 7.87 (m, 4H), 7.63 (dd, *J* = 8.5, 2.0 Hz, 1H), 7.57 – 7.51 (m, 2H), 7.31 (q, *J* = 1.5 Hz, 1H), 6.77 (d, *J* = 1.5 Hz, 1H), 3.81 (s, 6H), 3.24 (d, *J* = 1.5 Hz, 2H). **^{13}C NMR** (126 MHz, $CDCl_3$) δ 191.67, 169.85, 141.14, 137.55, 137.13, 134.80, 133.28, 133.20, 128.72, 128.24, 127.72, 127.28, 126.71, 126.62, 125.05, 123.80, 55.13, 53.41, 25.55. **HRMS** (ESI) m/z calcd. for $C_{21}H_{19}O_5$ $[M+H]^+$ 351.1230, found 351.1227.

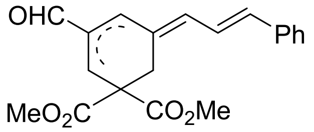

3ak + **3ak'**, **^{1}H NMR** (500 MHz, $CDCl_3$) δ 9.64 (s, 1H), 9.54 (s, 1H), 7.56 – 7.49 (m, 4H), 7.41–7.36 (m, 4H), 7.35 – 7.29 (m, 2H), 7.19 (dd, *J* = 15.0, 11.5 Hz, 1H), 6.98 (s, 1H), 6.84 (d, *J* = 15.5 Hz, 1H), 6.77 (d, *J* = 15.5 Hz, 1H), 6.63 (d, *J* = 11.5 Hz, 1H), 6.50 (d, *J* = 11.5 Hz, 1H), 3.74 (s, 6H), 3.73 (s, 6H), 3.18 (s, 2H), 3.04 (s, 2H), 2.98 (s, 2H), 2.97 (s, 2H). **^{13}C NMR** (125 MHz, $CDCl_3$) δ 192.44, 191.96, 170.83, 170.75, 147.43, 139.84, 138.71, 137.75, 137.15, 136.85, 136.60, 136.28, 135.91, 132.07, 130.32, 128.83, 128.63, 127.12, 126.92, 123.50, 122.61, 53.30, 53.15, 53.07, 53.02, 37.32, 31.44, 29.72, 27.84. **HRMS** (ESI) m/z calcd. for $C_{20}H_{21}O_5$ $[M+H]^+$ 341.1379, found 341.1384.

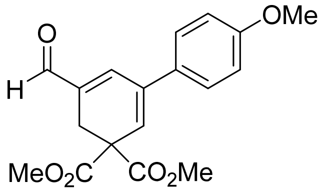

Dimethyl 5-formyl-4'-methoxy-[1,1'-biphenyl]-3,3(4H)-dicarboxylate

3al, ^{1}H NMR (400 MHz, $CDCl_3$) δ 9.66 (s, 1H), 7.46 – 7.40 (m, 2H), 7.14 (d, *J* = 1.2 Hz, 1H), 6.99 – 6.93 (m, 2H), 6.54 (d, *J* = 1.2 Hz, 1H), 3.86 (s, 3H), 3.78 (s, 6H), 3.18 (d, *J* = 1.2 Hz, 2H). **^{13}C NMR** (100 MHz, $CDCl_3$) δ 191.67, 169.94, 160.00, 141.38, 137.31, 136.55, 130.03, 127.19,

125.22, 114.21, 55.38, 54.99, 53.30, 25.49. **HRMS** (ESI) m/z calcd. for $C_{18}H_{19}O_6$ [M+H]$^+$ 331.1176, found 331.1174.

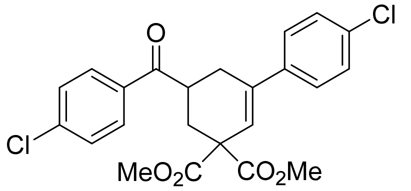

Dimethyl 4'-chloro-5-(4-chlorobenzoyl)-5,6-dihydro-[1,1'-biphenyl]-3,3(4H)-dicarboxylate

3am, **^{1}H NMR** (400 MHz, $CDCl_3$) δ 8.20 – 8.13 (m, 2H), 7.55 – 7.48 (m, 2H), 7.42 – 7.37 (m, 2H), 7.35 – 7.30 (m, 2H), 6.35 – 6.28 (m, 1H), 3.98 – 3.89 (m, 1H), 3.84 (s, 3H), 3.74 (s, 3H), 2.89 (ddd, *J* = 17.6, 11.2, 2.4 Hz, 1H), 2.84 – 2.79 (m, 1H), 2.55 – 2.46 (m, 1H), 1.79 (t, *J* = 13.2 Hz, 1H). **^{13}C NMR** (100 MHz, $CDCl_3$) δ 200.25, 171.02, 170.53, 139.93, 138.98, 138.85, 133.88, 130.22, 129.19, 128.58, 127.00, 120.31, 55.76, 53.19, 53.14, 39.59, 31.79, 28.91.
HRMS (ESI) m/z calcd. for $C_{23}H_{21}O_5Cl_2$ [M+H]$^+$ 447.0769, found 447.0761.

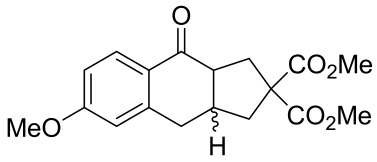

Dimethyl 7-methoxy-4-oxo-1,3,3a,4,9,9a-hexahydro-2H-cyclopenta[b]naphthalene-2,2-dicarboxylate

3an, ^{1}H NMR (500 MHz, $CDCl_3$) δ 7.99 (d, *J* = 8.5 Hz, 1H), 6.85 (dd, *J* = 8.5, 2.5 Hz, 1H), 6.68 (d, *J* = 1.5 Hz, 1H), 3.87 (s, 3H), 3.78 (s, 3H), 3.70 (s, 3H), 3.08 (dd, *J* = 16.0, 5.0 Hz, 1H), 2.97 (q, *J* = 7.5 Hz, 1H), 2.91 – 2.78 (m, 3H), 2.76 (dd, *J* = 8.0, 4.5 Hz, 1H), 2.49 (dd, *J* = 14.0, 6.5 Hz, 1H), 2.17 (dd, *J* = 14.0, 7.5 Hz, 1H). **^{13}C NMR** (125 MHz, $CDCl_3$) δ 197.13, 172.82, 172.35, 164.01, 144.13, 130.02, 125.22, 113.38, 112.98, 58.95, 55.46, 52.99, 52.89, 49.25, 39.08, 38.07, 36.35, 20.63. **HRMS** (ESI) m/z calcd. for $C_{18}H_{21}O_6$ [M+H]$^+$ 333.1329, found 331.1333.

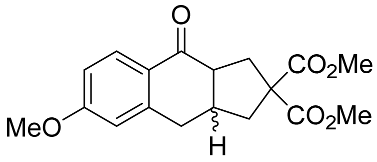

Dimethyl 7-methoxy-4-oxo-1,3,3a,4,9,9a-hexahydro-2H-cyclopenta[b]naphthalene-2,2-dicarboxylate

3an' ^{1}H NMR (500 MHz, $CDCl_3$) δ 8.02 (d, *J* = 8.5 Hz, 1H), 6.85 (d, *J* = 8.5 Hz, 1H), 6.74 (s, 1H), 3.88 (s, 3H), 3.78 (s, 3H), 3.75 (s, 3H), 3.17 (dd, *J* = 16.0, 4.0 Hz, 1H), 2.91 – 2.80 (m, 3H), 2.66 – 2.58 (m, 1H), 2.39 (dd, *J* = 14.0, 11.0 Hz, 1H), 2.36 – 2.28 (m, 1H), 1.99 (t, *J* = 12.0 Hz, 1H). **^{13}C NMR** (125 MHz, $CDCl_3$) δ 196.95, 172.84, 172.65, 163.49, 145.98, 129.50, 128.43, 113.34, 112.92, 55.47, 54.07, 52.96, 52.91, 43.21, 40.52, 36.18, 33.73, 29.33. **HRMS** (ESI) m/z calcd. for $C_{18}H_{21}O_6$ [M+H]$^+$ 333.1326, found 331.1330.

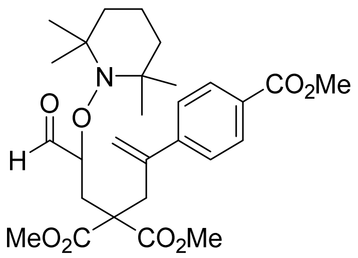

Dimethyl 2-(2-(4-(methoxycarbonyl)phenyl)allyl)-2-(3-oxo-2-((2,2,6,6-tetramethylpiperidin-1-yl)oxy)propyl)malonate

9a, **^{1}H NMR** (400 MHz, $CDCl_3$) δ 9.85 (d, *J* = 4.5 Hz, 1H), 7.98 (d, *J* = 8.4 Hz, 2H), 7.37 (d, *J* = 8.4 Hz, 2H), 5.39 (s, 1H), 5.33 (s, 1H), 4.36 – 4.32 (m, 1H), 3.93 (s, 3H), 3.50 (s, 3H), 3.37 (s, 3H), 3.36 (d, *J* = 14.4 Hz, 1H), 3.27 (d, *J* = 14.4 Hz, 1H), 2.37 (dd, *J* = 15.2, 8.0 Hz, 1H), 2.23 (dd, *J* = 15.2, 4.4 Hz, 1H), 1.55 – 1.39 (m, 4H), 1.38 – 1.26 (m, 2H), 1.22 – 1.07 (m, 12H). **^{13}C NMR** (100 MHz, $CDCl_3$) δ 201.80, 170.65, 170.59, 166.76, 145.98, 143.23, 129.42, 129.16, 126.96, 120.92, 83.13, 61.44, 59.99, 54.78, 52.48, 52.33, 52.12, 40.16, 39.84, 37.60, 34.02, 33.95, 32.46, 20.65, 20.54, 17.01. **HRMS** (ESI) m/z calcd. for $C_{28}H_{40}NO_8$ $[M+H]^+$ 391.1754, found 391.1751.

DIVERSE SYNTHETIC APPLICATION

Synthesis of compound 4a:

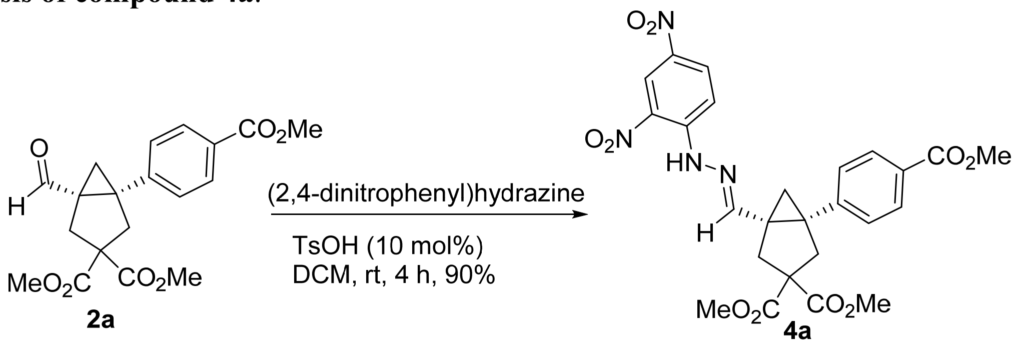

To a flame-dried flask equipped with a magnetic stir bar were added **2a** (53 mg, 0.147 mmol), (2,4-dinitrophenyl)hydrazine (30 mg, 1.0 equiv), *p*-toluenesulfonic acid (2.6 mg, 10 mol%) and DCM (2.0 mL). The resulting mixture was stirred at rt for 4 hours. After completion, the mixture was diluted with water, then extracted with ethyl acetate. The organic layer was washed with brine, dried with $MgSO_4$, filtered and concentrated. Flash chromotagraphy (petroleum ether/ ethyl acetate =8/1-4/1) gave the corresponding product **4a** as a yellow solid in (71 mg, 90% yield).

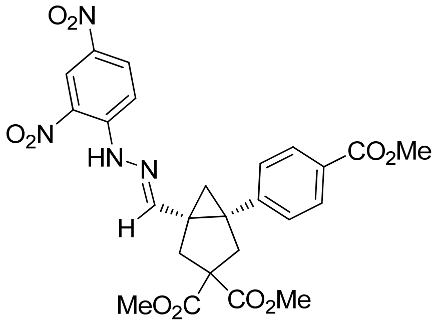

(*1S,5S*)-dimethyl 1-((E)-(2-(2,4-dinitrophenyl)hydrazono)methyl)-5-(4 (methoxycarbonyl) phenyl)bicyclo[3.1.0]hexane-3,3-dicarboxylate

4a, **^{1}H NMR** (400 MHz, $CDCl_3$) δ 10.90 (s, 1H), 9.04 (d, *J* = 2.4 Hz, 1H), 8.28 (dd, *J* = 9.6, 2.4 Hz, 1H), 7.99 (d, *J* = 8.0 Hz, 2H), 7.87 (d, *J* = 9.6 Hz, 1H), 7.40 (d, *J* = 8.4 Hz, 2H), 6.77 (s, 1H), 3.90 (s, 3H), 3.83 (s, 3H), 3.81 (s, 3H), 3.26 (d, *J* = 14.0 Hz, 1H), 3.07 (d, *J* = 14.4 Hz, 1H), 3.01 (d, *J* = 14.0 Hz, 1H), 2.90 (dd, *J* = 14.0, 0.8 Hz, 1H), 1.67 (d, *J* = 6.4 Hz, 1H), 1.40 (d, *J* = 6.4 Hz, 1H). **^{13}C NMR** (100 MHz, $CDCl_3$) δ 172.57, 171.57, 166.54, 151.87, 144.61, 144.15, 137.82, 130.12, 129.90, 129.43, 129.38, 128.71, 123.43, 116.48, 58.11, 53.45, 53.29, 52.19, 43.88, 42.22, 37.71, 37.12, 21.66. **HRMS** (ESI) m/z calcd. for $C_{25}H_{25}N_4O_{10}$ 541.1578, found 541.1565.
HPLC analysis: Chiralcel AD-H, *i*-PrOH/*n*-hexane = 80/20, flow rate 1.0 mL/min. λ = 230 nm, t(minor) = 20.07 min, t(major) = 26.41 min, 95.5:4.5 er.

Synthesis of compound 5a:[14]

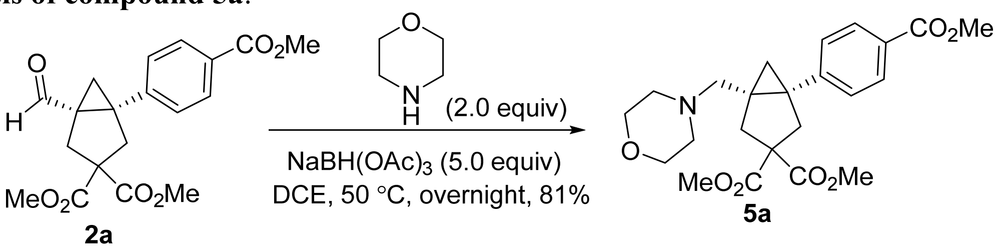

To a flame-dried flask equipped with a magnetic stir bar were added **2a** (42 mg, 0.117 mmol), morpholine (21 *μL*, 2.0 equiv), and DCE (3.0 mL). To the stirred solution, $NaBH(OAc)_3$ (125 mg, 5.0 equiv) was added and the resulting mixture was stirred at 50 °C overnight. After completion, the mixture was treated with saturated $NaHCO_3$ solution, then extracted with ethyl acetate. The organic layer was washed with brine, dried with $MgSO_4$, filtered and concentrated. Flash chromotagraphy (petroleum ether/ ethyl acetate =10/1-5/1) gave the product **5a** (41 mg, 81% yield).

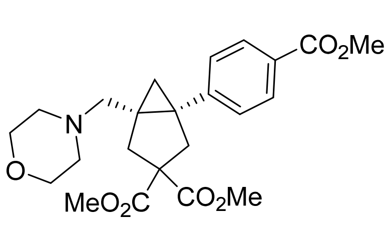

Dimethyl-1-(4-(methoxycarbonyl)phenyl)-5-(morpholinomethyl)bicyclo[3.1.0]hexane-3,3-dicarboxylate

5a, 81% yield; **^{1}H NMR** (500 MHz, $CDCl_3$) δ 7.99 (d, *J* = 8.5 Hz, 2H), 7.34 (d, *J* = 8.5 Hz, 2H), 3.92 (s, 3H), 3.79 (s, 3H), 3.76 (s, 3H), 3.72 – 3.60 (m, 4H), 2.95 – 2.83 (m, 3H), 2.61 (d, *J* = 13.0 Hz, 2H), 2.43 – 2.41 (m, 2H), 2.30 – 2.18 (m, 2H), 1.42 (d, *J* = 12.5 Hz, 1H), 1.08 (d, *J* = 6.0 Hz, 1H), 0.89 (d, *J* = 6.0 Hz, 1H). **^{13}C NMR** (125 MHz, $CDCl_3$) δ 173.02, 172.21, 166.94, 145.75, 129.68, 129.16, 128.49, 66.85, 63.11, 58.59, 53.79, 53.19, 53.05, 52.11, 43.95, 41.24, 35.51, 32.56, 20.57.
HRMS (ESI) m/z calcd. for $C_{23}H_{30}NO_7$ $[M+H]^+$ 432.2017, found 432.2011.
HPLC analysis: Chiralcel OD-3, *i*-PrOH/*n*-hexane = 95/5, flow rate 0.6 mL/min. λ = 254 nm, t(minor) = 20.87 min, t(major) = 23.42 min, 94:6 er.

Synthesis of compound 6a:

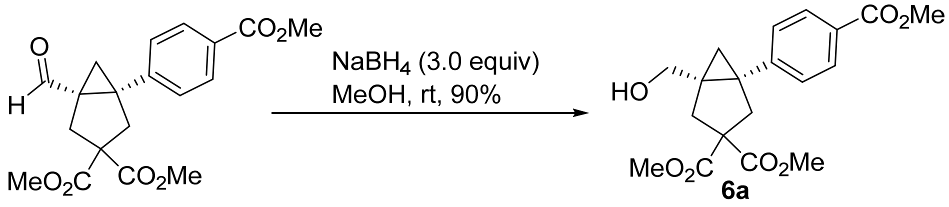

To a flame-dried flask equipped with a magnetic stir bar were added **2a** (20 mg, 0.056 mmol) and MeOH (1.5 mL). To the stirred solution, $NaBH_4$ (6.4 mg, 3.0 equiv) was added and the resulting mixture was stirred at rt for 1 h. After completion, the mixture was treated with saturated $NaHCO_3$ solution, then extracted with ethyl acetate. The organic layer was washed with brine, dried with $MgSO_4$, filtered and concentrated. Flash chromotagraphy (petroleum ether/ ethyl acetate =5/1-2/1) gave the product **6a** (18.5 mg, 90% yield).

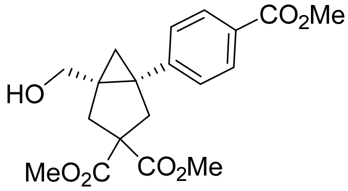

Dimethyl-1-(hydroxymethyl)-5-(4-(methoxycarbonyl)phenyl)bicyclo[3.1.0]hexane-3,3-dicarboxylate
6a, 90% yield; **^{1}H NMR** (500 MHz, $CDCl_3$) δ 8.00 (d, *J* = 8.3 Hz, 2H), 7.42 (d, *J* = 8.3 Hz, 2H), 3.93 (s, 3H), 3.79 (s, 3H), 3.77 (s, 3H), 3.45 (d, *J* = 12.0 Hz, 1H), 3.30 (d, *J* = 12.0 Hz, 1H), 2.94 (d, *J* = 14.0 Hz, 1H), 2.91 – 2.81 (m, 3H), 1.10 (d, *J* = 6.0 Hz, 1H), 0.84 (d, *J* = 6.0 Hz, 1H).
^{13}C NMR (125 MHz, $CDCl_3$) δ 172.94, 172.24, 166.89, 145.72, 129.86, 129.30, 128.69, 65.06, 58.56, 53.24, 53.08, 52.14, 44.25, 38.55, 37.63, 37.11, 18.17.
HRMS (ESI) m/z calcd. for $C_{19}H_{21}O_6$ $[M-OH]^+$ 345.1333, found 345.1329.
HPLC analysis: Chiralcel AD-3, *i*-PrOH/*n*-hexane = 80/20, flow rate 0.8 mL/min. λ = 254 nm, t(minor) = 13.89 min, t(major) = 20.35 min, 94:6 er.

Synthesis of compound 7a:

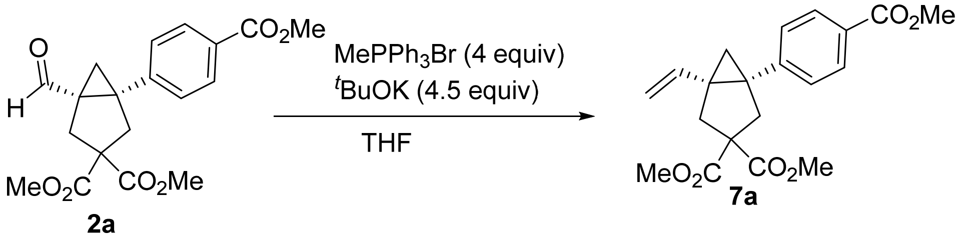

To a flame-dried flask equipped with a magnetic stir bar were added methyltriphenylphosphonium bromide (150 mg, 0.42 mmol) and dry THF (3.0 mL). To the stirred solution, *ᵗ*BuOK (52 mg, 3.3 equiv) was added and the resulting mixture was stirred at rt for 1 h. Then **2a** (50 mg, 0.14 mmol) in dry THF (1.0 mL) was added to the above solution and the resultant reaction mixture was stirred at reflux overnight. The reaction mixture was cooled to rt and treated with water, then extracted with ethyl acetate. The organic layer was washed with brine, dried with $MgSO_4$, filtered and concentrated. Flash chromotagraphy (petroleum ether/ ethyl acetate =10/1-5/1) recovered 23 mg **2a** and gave the product **7a** (24 mg, 90% yield based on recovered starting material).

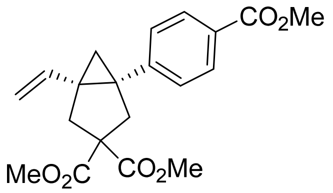

Dimethyl-1-(4-(methoxycarbonyl)phenyl)-5-vinylbicyclo[3.1.0]hexane-3,3-dicarboxylate
7a, 90% yield BRSM; **^{1}H NMR** (500 MHz, $CDCl_3$) δ 8.01 – 7.95 (m, 2H), 7.36 – 7.31 (m, 2H), 5.15 (d, *J* = 10.3 Hz, 0.3 H), 5.12 (d, *J* = 10.3 Hz, 0.7H), 5.05 (dd, *J* = 17.3, 1.6 Hz, 1H), 4.93 (dd, *J* = 10.3, 1.6 Hz, 1H), 3.92 (s, 3H), 3.81 (s, 3H), 3.77 (s, 3H), 3.00 (d, *J* = 14.0 Hz, 1H), 2.89 (d, *J* = 14.0 Hz, 1H), 2.85 (d, *J* = 14.0 Hz, 1H), 2.78 (dd, *J* = 14.0, 1.6 Hz, 1H), 1.23 (d, *J* = 6.5 Hz, 1H), 1.06 (d, *J* = 6.5 Hz, 1H). **^{13}C NMR** (125 MHz, $CDCl_3$) δ 172.92, 172.05, 166.97, 145.80, 139.10, 129.65, 129.57, 128.51, 113.11, 77.30, 77.04, 76.79, 58.04, 53.26, 53.10, 52.09, 43.85, 39.93, 38.27, 37.08, 20.83.
HRMS (ESI) m/z calcd. for $C_{20}H_{23}O_6$ $[M+H]^+$ 359.1489, found 359.1483.
HPLC analysis: Chiralcel OD-3, *i*-PrOH/*n*-hexane = 98/2, flow rate 0.3 mL/min. λ = 254 nm, t(major)= 33.99 min, t(minor) = 38.12 min, 94.5:5.5 er.

Synthesis of compound 8a:[15]

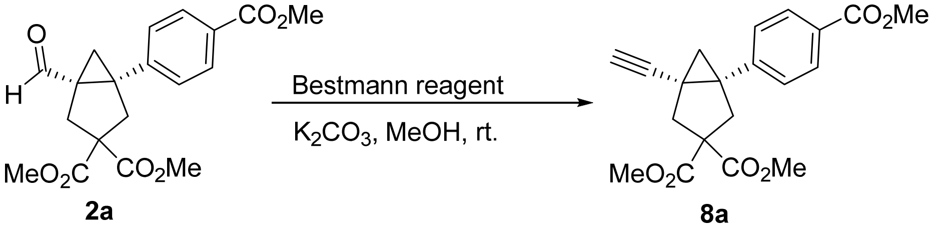

To a flame-dried flask equipped with a magnetic stir bar were added **2a** (28 mg, 0.078 mmol), K_2CO_3 (33 mg, 3.0 equiv) and MeOH (2.0 mL). To the stirred solution, Bestmann reagent

(dimethyl(acetyldiazomethyl)phosphonate, 24 μL, 2.0 equiv) was added and the resulting mixture was stirred at rt for 6 h. After completion, the mixture was treated with saturated $NaHCO_3$ solution, then extracted with ethyl acetate. The organic layer was washed with brine, dried with $MgSO_4$, filtered and concentrated. Flash chromotagraphy (petroleum ether/ ethyl acetate = 10/1-6/1) gave the product **8a** (24 mg, 85% yield).

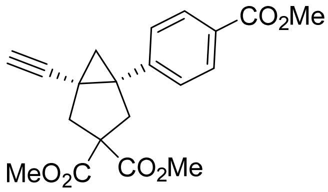

Dimethyl-1-ethynyl-5-(4-(methoxycarbonyl)phenyl)bicyclo[3.1.0]hexane-3,3-dicarboxylate 8a, 85% yield; ^{1}**H NMR** (400 MHz, $CDCl_3$) δ 8.07 – 7.98 (m, 2H), 7.49 – 7.43 (m, 2H), 3.92 (s, 3H), 3.80 (s, 3H), 3.76 (s, 3H), 3.05 (d, J = 14.0 Hz, 1H), 2.98 (d, J = 14.0 Hz, 1H), 2.86 – 2.83 (m, 2H), 1.97 (s, 1H), 1.40 (d, J = 6.2 Hz, 1H), 1.15 (d, J = 6.2 Hz, 1H). ^{13}**C NMR** (100 MHz, $CDCl_3$) δ 172.52, 171.29, 166.97, 144.80, 129.59, 128.78, 128.72, 83.92, 69.72, 58.22, 53.33, 53.20, 52.08, 42.39, 40.90, 39.92, 25.84, 23.13.

HRMS (ESI) m/z calcd. for $C_{20}H_{21}O_6$ $[M+H]^+$ 357.1333, found 357.1327.

HPLC analysis: Chiralcel AD-3, *i*-PrOH/*n*-hexane = 90/10, flow rate 0.8 mL/min. λ = 254 nm, t(minor) = 15.96 min, t(major) = 17.79 min, 94.5:5.5 er.

Mechanism Studies

Control Experiments on the Radical Pathway:

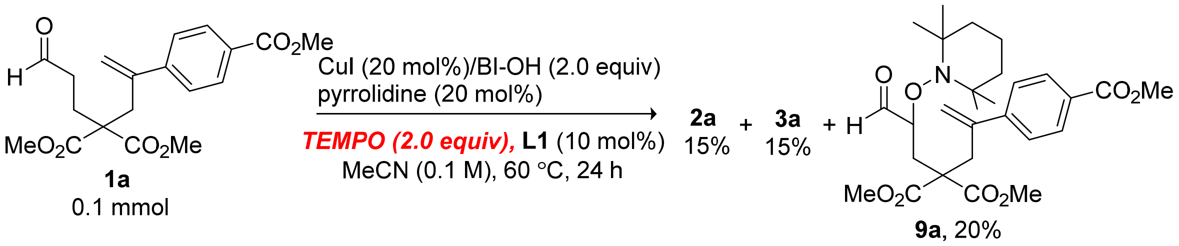

To a flame-dried Schlenk tube equipped with a magnetic stir bar were added **1a** (0.1 mmol), CuI (3.8 mg, 20 mol%), **L1** (2.7 mg, 10 mol%), BI-OH (108 mg, 0.2 mmol) and TEMPO (32 mg, 0.2 mmol). The tube was evacuated and backfilled with argon for three times. Pyrrolidine (1.7 μL, 20 mol%) and freshly degassed acetonitrile (1.0 mL) was added *via* syringe. The tube was stirred at 60 °C for 12h. After completion, solvent was removed under reduced pressure, and the reaction mixture was checked by crude ^{1}H NMR, which detects the formation of **2a** (15%) and **3a** (15%) and **9a** (20%).

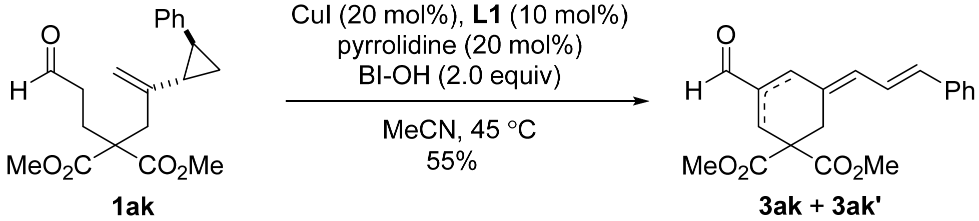

To a flame-dried Schlenk tube equipped with a magnetic stir bar were added **1ak** (0.1 mmol), CuI (3.8 mg, 20 mol%), **L1** (2.7 mg, 10 mol%) and BI-OH (108 mg, 0.2 mmol). The tube was evacuated and backfilled with argon for three times. Pyrrolidine (2.6 μL, 30 mol%) and freshly degassed acetonitrile (1.0 mL) was added *via* syringe. The tube was stirred at 45 °C for 24 h. After completion, solvent was removed under reduced pressure. Flash chromotagraphy (petroleum ether/ ethyl acetate =20/1-10/1) gave a mixture of **3ak** and **3ak'**.

Different Cyclization Preferences for Aryl-Substituted Germinal Alkene and Mono-Substituted Terminal Alkene:

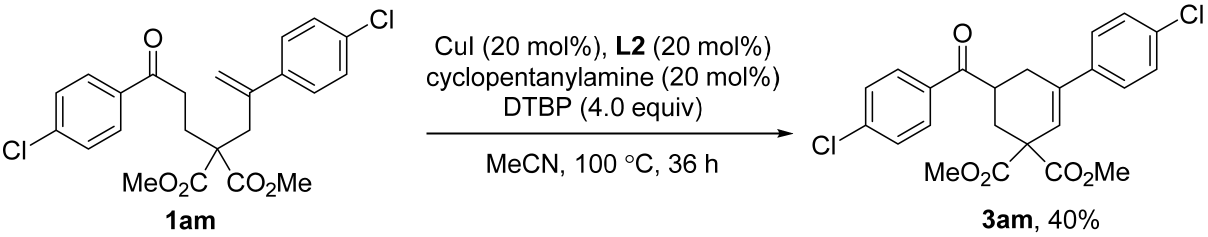

To a flame-dried Schlenk tube equipped with a magnetic stir bar were added **1am** (0.2 mmol), CuI (7.6 mg, 20 mol%) and **L2** (6.3 mg, 20 mol%). The tube was evacuated and backfilled with argon for three times. Cyclopentanylamine (2.0 μL, 20 mol%), DTBP (300 μL,) and freshly degassed acetonitrile (2.0 mL) was added *via* syringe. The tube was stirred at 100 °C for 36 h. After completion, solvent was removed under reduced pressure. Flash chromotagraphy (petroleum ether/ ethyl acetate =20/1-10/1) gave **3am** in 40% yield.

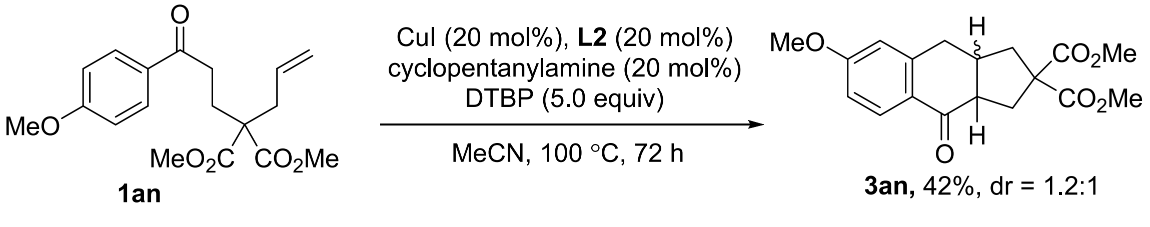

To a flame-dried Schlenk tube equipped with a magnetic stir bar were added **1an** (0.2 mmol), CuI (7.6 mg, 20 mol%) and **L2** (6.3 mg, 20 mol%). The tube was evacuated and backfilled with argon for three times. Cyclopentanylamine (4.0 μL, 20 mol%), DTBP (200 μL, 5.0 equiv.) and freshly degassed acetonitrile (2.0 mL) was added *via* syringe. The tube was stirred at 100 °C for

72 h. After completion, solvent was removed under reduced pressure. Flash chromotagraphy (petroleum ether/ ethyl acetate =20/1-10/1) gave **3an** in 42% yield with 1.2:1 ratio.

Control Experiments on the Catalyst and the Oxidant:

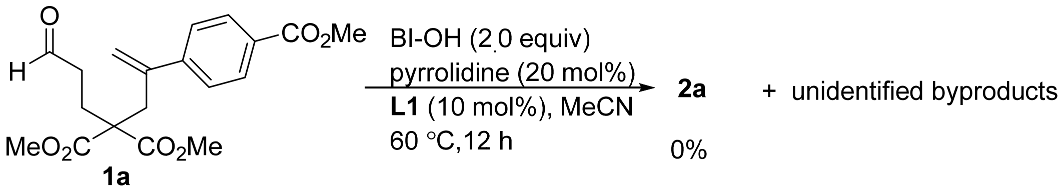

To a flame-dried Schlenk tube equipped with a magnetic stir bar were added **1a** (0.1 mmol), **L1** (2.7 mg, 10 mol%) and BI-OH (108 mg, 0.2 mmol). The tube was evacuated and backfilled with argon for three times. Pyrrolidine (1.7 μL, 20 mol%) and freshly degassed acetonitrile (1.0 mL) was added *via* syringe. The tube was stirred at 60 °C for 12 h. After that, solvent was removed under reduced pressure, and the reaction mixture was checked by crude ^{1}H NMR, which detects the complete assumption of **1a**, while no formation of **2a**.

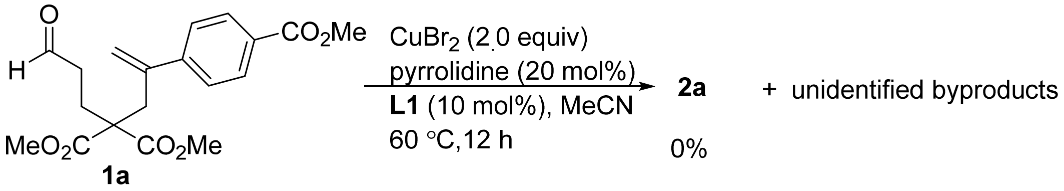

To a flame-dried Schlenk tube equipped with a magnetic stir bar were added **1a** (0.1 mmol), **L1** (2.7 mg, 10 mol%) and $CuBr_2$ (45 mg, 0.2 mmol). The tube was evacuated and backfilled with argon for three times. Pyrrolidine (1.7 μL, 20 mol%) and freshly degassed acetonitrile (1.0 mL) was added *via* syringe. The tube was stirred at 60 °C for 12h. After that, solvent was removed under reduced pressure, and the reaction mixture was checked by crude ^{1}H NMR, which detects the complete assumption of **1a**, while no formation of **2a**.

Supplementary References:

1 Lin, Q. *et al.* Enantioselective synthesis of Janus kinase inhibitor INCB018424 via an organocatalytic aza-Michael reaction. *Org. Lett.* **11**, 1999–2002 (2009).

2 Stiller, J. *et al.* Enantioselective α- and γ-alkylation of α,β-unsaturated aldehydes using dienamine activation. *Org. Lett.* **13**, 70–73 (2011).

3 Kraszkiewicz, L. & Skulski, L. Optimized syntheses of iodylarenes from iodoarenes, with sodium periodate as the oxidant. Part II. *Arkivoc*, 120–125 (2003).

4 Ferrer, C., Raducan, M., Nevado, C., Claverie, C. K. & Echavarren, A. M. Missing cyclization pathways and new rearrangements unveiled in the gold(I) and platinum(II)-catalyzed cyclization of 1,6-enynes. *Tetrahedron* **63**, 6306–6316 (2007).

5 Erkkilä, A. & Pihko, P. M. Mild organocatalytic α-methylenation of aldehydes. *J. Org. Chem.* **71**, 2538–2541 (2006).

6 Ma, W., Fang, J., Ren, J. & Wang, Z. Lewis acid catalyzed formal intramolecular [3 + 3] cross-cycloaddition of cyclopropane 1,1-diesters for construction of benzobicyclo[2.2.2]octane skeletons. *Org. Lett.* **17**, 4180–4183 (2015).

7 Ikeda, H. *et al.* Evidence for significant through-space and through-bond electronic coupling in the 1,4-diphenylcyclohexane-1,4-diyl radical cation gained by absorption spectroscopy and DFT calculations. *Chem. Eur. J.* **13**, 9207–9215 (2007).

8 Ohmura, T., Masuda, K., Takase, I. & Suginome, M. Palladium-catalyzed silylene-1,3-diene [4 + 1] cycloaddition with use of (aminosilyl)boronic esters as synthetic equivalents of silylene. *J. Am. Chem. Soc.* **131**, 16624–16625 (2009).

9 Campaña, A. G. *et al.* Sodium tetramethoxyborate: an efficient catalyst for Michael additions of stabilized carbon nucleophiles. *J. Org. Chem.* **72**, 8127–8130 (2007).

10 Xiao, Y.-P., Liu, X.-Y. & Che, C.-M. Efficient gold(I)-catalyzed direct intramolecular hydroalkylation of unactivated alkenes with α-ketones. *Angew. Chem. Int. Ed.* **50**, 4937–4941 (2011).

11 Lin, S., Song, C.-X., Cai, G.-X., Wang, W.-H. & Shi, Z.-J. Intra/Intermolecular direct allylic alkylation via Pd(II)-catalyzed allylic C−H activation. *J. Am. Chem. Soc.* **130**, 12901–12903 (2008).

12 Karila, D., Leman, L. & Dodd, R. H. Copper-catalyzed iminoiodane-mediated aminolactonization of olefins: application to the synthesis of 5,5-disubstituted butyrolactones. *Org. Lett.* **13**, 5830–5833 (2011).

13 Cheng, Y.-F., Dong, X.-Y., Gu, Q.-S., Yu, Z.-L. & Liu, X.-Y. Achiral pyridine ligand-enabled enantioselective radical oxytrifluoromethylation of alkenes with alcohols. *Angew. Chem. Int. Ed.* **56**, 8883–8886 (2017).

14 Abdel-Magid, A. F., Carson, K. G., Harris, B. D., Maryanoff, C. A. & Shah, R. D. Reductive amination of aldehydes and ketones with sodium triacetoxyborohydride. Studies on direct and indirect reductive amination procedures. *J. Org. Chem.* **61**, 3849–3862 (1996).

15 Roth, G. J., Liepold, B., Muller, S. G. & Bestmann, H. J. Further improvements of the synthesis of alkynes from aldehydes. *Synthesis*, 59–62 (2004).
